# Identification of Potential miRNA-mRNA Regulatory Network Contributing to Hypertrophic Cardiomyopathy (HCM)

**DOI:** 10.3389/fcvm.2021.660372

**Published:** 2021-05-31

**Authors:** Lin Wang, Fengmin Lu, Jing Xu

**Affiliations:** Cardiology Department, Tianjin Chest Hospital, Tianjin, China

**Keywords:** hypertrophic cardiomyopathy, miRNA-mRNA network, go, KEGG, logistic model

## Abstract

**Background:** Hypertrophic cardiomyopathy (HCM) is a myocardial disease with unidentified pathogenesis. Increasing evidence indicated the potential role of microRNA (miRNA)-mRNA regulatory network in disease development. This study aimed to explore the miRNA-mRNA axis in HCM.

**Methods:** The miRNA and mRNA expression profiles obtained from the Gene Expression Omnibus (GEO) database were used to identify differentially expressed miRNAs (DEMs) and genes (DEGs) between HCM and normal samples. Target genes of DEMs were determined by miRTarBase. Gene ontology (GO) annotation and Kyoto Encyclopedia of Genes and Genomes (KEGG) pathway analysis were conducted to identify biological functions of the DEGs and DEMs. miRNA-mRNA regulatory network was constructed to identify the hub genes and miRNAs. Logistic regression model for HCM prediction was established basing on the network.

**Results:** A total of 224 upregulated and 366 downregulated DEGs and 10 upregulated and 14 downregulated DEMs were determined. We identified 384 DEM-targeted genes, and 20 of them were overlapped with the DEGs. The enriched functions include extracellular structure organization, organ growth, and phagosome and melanoma pathways. The four miRNAs and three mRNAs, including hsa-miR-373, hsa-miR-371-3p, hsa-miR-34b, hsa-miR-452, ARHGDIA, SEC61A1, and MYC, were identified through miRNA-mRNA regulatory network to construct the logistic regression model. The area under curve (AUC) values over 0.9 suggested the good performance of the model.

**Conclusion:** The potential miRNA-mRNA regulatory network and established logistic regression model in our study may provide promising diagnostic methods for HCM.

## Highlights

• hsa-miR-373, hsa-miR-371-3p, hsa-miR-34b, hsa-miR-452, ARHGDIA, SEC61A1, and MYC were potential pathological factors for HCM.
• The logistic model based on the miRNA-mRNA regulatory network could effectively distinguish HCM samples from normal samples.

## Introduction

Hypertrophic cardiomyopathy (HCM), a common but complex cardiovascular disorder which was found over 50 years ago, is featured by uncertain asymmetry left ventricular (LV) thickening in the absence of other cardiac, metabolic, or systemic diseases (1, 2). HCM often occurs in perioperative period, causing shrunken ventricular cavity, blocked blood filling, and reduced left ventricular pressure during diastole and is closely associated with the risk of sudden death and heart failure (3). The phenotypes of HCM contain obstructive hypertrophic cardiomyopathy (HOCM) and nonobstructive hypertrophic cardiomyopathy (NHCM), while the genotypes are variable (1). Accurate diagnosis of HCM is crucial for the perioperative management of patients and their survival (4). Echocardiography and magnetic resonance imaging (MRI) assessment of left ventricular function are the most common diagnostic methods of HCM in clinical practice, which are solely based on the clinical criteria (5, 6). Moreover, previous epidemiologic studies suggested that the diagnostic rate of echocardiography was notably lower than that of combination of echocardiography and genetic diagnosis (7, 8). Hence, the exploration of genetic diagnostic methods for HCM is clinically significant.

MicroRNAs (miRNAs) are highly conserved endogenous single-strand RNAs, consisting of 20–25 nucleotides that do not translate to any proteins (9). MiRNAs function through interacting with 3′ untranslated region (3′-UTR) of the target mRNAs, disrupting the translation or facilitating degradation (10). In the past decades, the functions of miRNAs were widely studied in various diseases, particularly in cardiovascular diseases, which could cause the major alteration in cellular behaviors such as cell proliferation, migration, and apoptosis (11). Increasing evidences are suggesting the important role of miRNAs in HCM pathogenesis. For example, miR-20a-5p was highly expressed in HCM tissues compared with the control tissues and probably interacted with MFN2 to promote cardiomyocyte hypertrophy (12). A previous microarray analysis on human serum revealed several circulating miRNAs, including miR-18a-5p, miR-30d-5p, miR-21-5p, miR-193-5p, miR-10b-5p, miR-15a-5p, miR-296-5p, and miR-29a-3p, were potential diagnostic biomarkers for diffuse myocardial fibrosis during HCM (13). Moreover, Prestes et al. (14) reported a validated miRNA-mRNA network composed of two upregulated miRNAs, miR-34a-5p and miR-17-5p, and five targeted genes including Pkp2, Rbm20, Ryr2, Tpm1, and Vcl in hypertrophic heart rat. Yet so far, no systemic and detailed analysis of miRNA-mRNA regulatory network was conducted to improve the diagnostic method of HCM.

In this study, we analyzed the differentially expressed genes and miRNAs in HCM samples compared with the normal group, trying to elucidate the potential miRNA-mRNA regulatory network during HCM and identify its diagnostic role.

## Materials and Methods

### Data Collection

We downloaded the mRNA expression dataset GSE36961 and miRNA expression dataset GSE36946 from Gene Expression Omnibus (GEO, https://www.ncbi.nlm.nih.gov/geo/) database. The GSE36961 dataset consisted of 106 heart samples from hypertrophic cardiomyopathy (HCM) patients and 39 samples from healthy hearts as control and was detected by Illumina Human HT-12 V3.0 expression beadchip platform. The miRNA expression dataset GSE36946 contained 127 heart samples, including 107 HCM samples and 20 samples from healthy donors, and was detected by Illumina Human v2 MicroRNA expression beadchip platform.

### Identification of Differentially Expressed Genes

The raw data of GSE36961 and GSE36946 were normalized by robust multiarray (RMA) method and standardization by log_2_ transformation. We then applied *limma* package (15) to screen differentially expressed genes (DEGs) and differentially expressed miRNAs (DEMs) between HCM samples and the control group. The |log_2_FC| ≥ 1 and *p* < 0.05 were considered the criteria for screening.

### Prediction of miRNA Targets

The targets of screened DEMs were identified *via* miRTarBase database (Release 7.0: Sept. 15, 2017 mirtarbase.mbc.nctu.edu.tw).

### Functional Enrichment Analysis

We performed gene ontology (GO) analysis, which includes biological process, molecular function and cellular component, and Kyoto Encyclopedia of Genes and Genomes (KEGG) pathway enrichment analysis to determine the biological functions of the DEGs and DEMs. Adjusted *P* < 0.05 was considered the threshold value.

### miRNA-mRNA Interaction Network Analysis

We calculated the correlation between the DEGs and DEMs and screened out the significantly negatively correlated miRNA-mRNA pairs (*P* < 0.05) basing on the hypothesis that miRNA negatively regulated the target mRNA. The visualization of miRNA-mRNA regulatory network was realized by Cytoscape software (16) (version 3.7.2). The hub genes in the interaction network were determined by the cytoHubba plug-in of Cytoscape software, basing on the maximum neighborhood component (MNC) algorithm.

### Logistic Regression Model

Logistic regression is a common method adopted in predicting classification basing on a set of variables. In this study, we classified all samples into two sets, namely the normal control samples and HCM samples, adopted the expression of miRNA/mRNA as continuous predictor variable by *glm* package in R software and sample type as categorical response value to construct the logistic regression model. We then classified the samples into the training set and validation set to establish and testify this model.

## Results

### Differentially Expressed mRNAs and miRNAs in HCM

**Figure 1:**
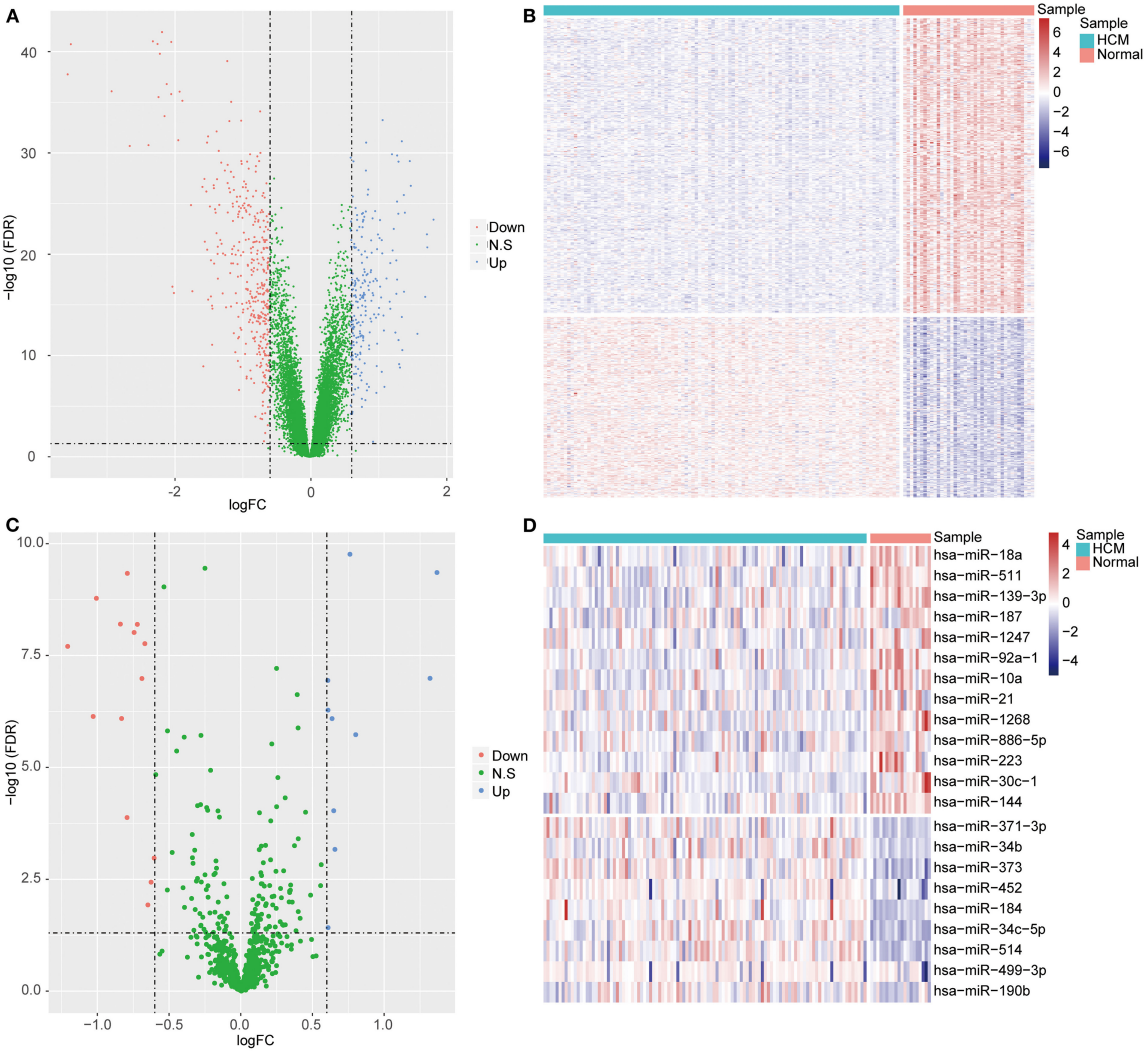
Differentially expressed mRNAs (DEGs) and miRNAs (DEMs) between HCM samples and healthy controls. **(A)** Volcano plot of DEGs in GSE36961. The *x* axis represents log_2_FC of differential expression values, the *y* axis represents –log10 (FDR), blue dots represent upregulated mRNAs, and red dots represent downregulated mRNAs. **(B)** Heatmap of DEGs in GSE36961. The *x* axis represents samples, and the *y* axis represents genes. **(C,D)** The volcano plot **(C)** and heatmap **(D)** of DEMs from GSE36946 dataset.

We carried out standardization on the raw data from GSE36961 (Supplementary Figure 1A) and GSE36946 (Supplementary Figure 1B) datasets and found little biases across samples, which indicated the elimination of expression intensity alteration caused by experimental technique. The following analysis on GSE36961 identified 590 DEGs in HCM samples compared with the control samples, including 224 upregulated ones and 366 downregulated ones (Figures 1A,B). A total of 24 DEMs between HCM samples and the control samples were obtained from GSE36946 dataset, comprising 10 upregulated miRNAs and 14 downregulated miRNAs (Figures 1C,D). Moreover, we identified 384 target mRNAs of the 24 DEMs, among which 20 mRNAs were overlapped with the DEGs from the GSE36961 dataset (Supplementary Figure 2A).

### Functional Enrichment Analysis of the DEGs and DEMs

**Figure 2:**
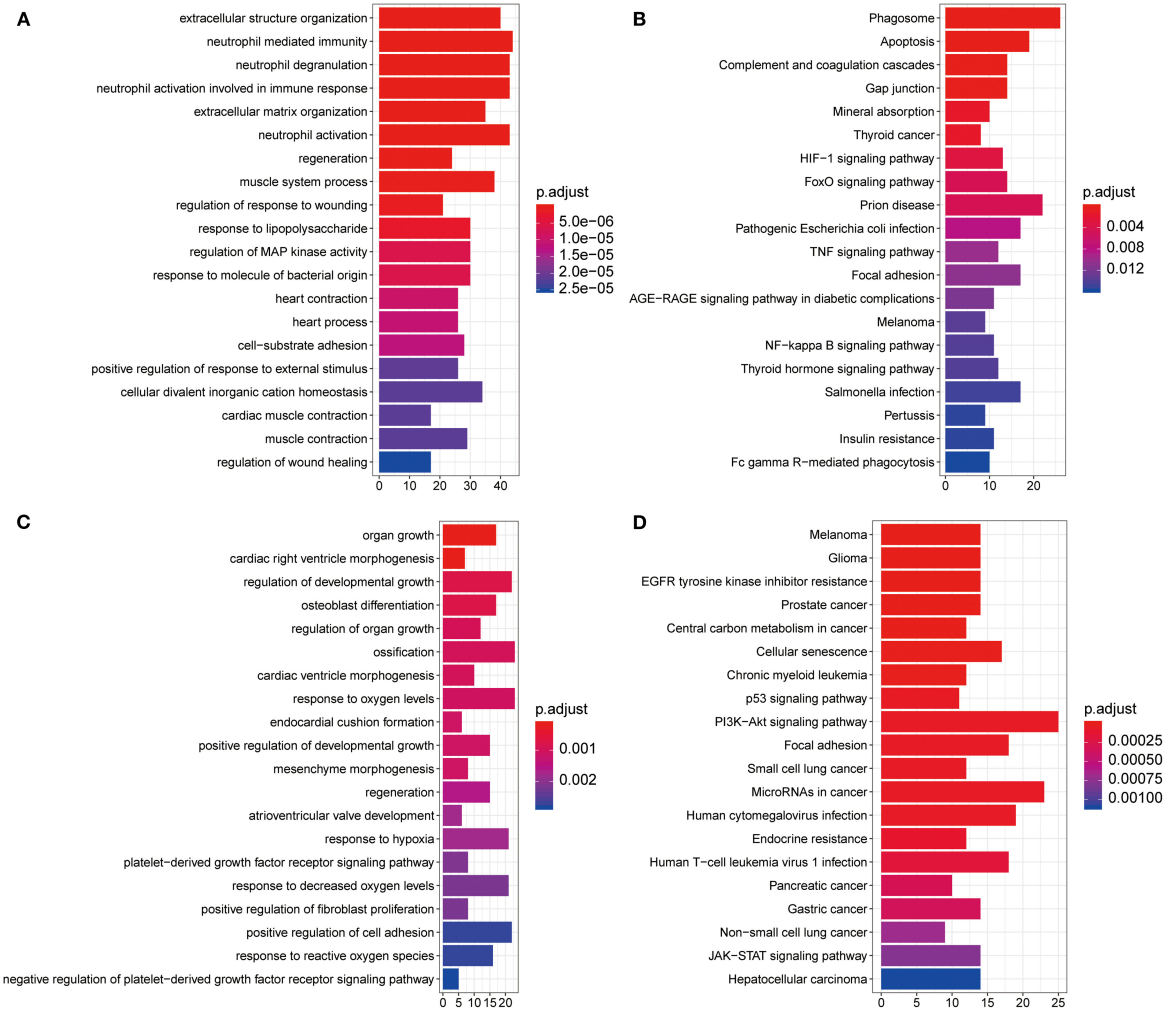
Gene Ontology (GO) and Kyoto Encyclopedia of Genes and Genomes (KEGG) pathway enrichment analysis. **(A)** Histogram of the top 20 most significantly enriched GO terms from DEGs in GSE36961. The *x* axis shows number of mRNAs, and the *y* axis shows the name of GO terms. **(B)** The top 20 most significantly enriched KEGG pathways of DEGs. The *x* axis shows number of mRNAs, and the *y* axis shows the name of KEGG pathways. **(C,D)** Histogram of the top 20 most significantly enriched GO terms **(C)** and KEGG pathways **(D)** from DEMs in GSE36946. DEGs, differentially expressed genes; DEMs, differently expressed miRNAs.

To evaluate the potential biological function of the 590 DEGs and 24 DEMs summarized above, we performed GO analysis and KEGG pathway enrichment analysis. A total of 826 GO terms and 55 KEGG pathways were identified for the DEGs (Supplementary Table 1). The top 20 GO terms are shown in Figure 2A, including extracellular structure organization and neutrophil mediated immunity. The top 20 KEGG pathways mainly contained phagosome and apoptosis (Figure 2B). As for the target genes of the 24 DEMs, 322 enriched GO terms, and 55 KEGG pathways were obtained (Supplementary Table 2). The top 20 GO terms including organ growth and top 20 KEGG pathways including melanoma were presented in Figures 2C,D, separately. Moreover, we found 96 overlapped GO terms (Supplementary Figure 2B) and 24 overlapped KEGG pathways (Supplementary Figure 2C) between the two datasets, such as regeneration and positive regulation of reactive oxygen species metabolic process in GO category and melanoma and central carbon metabolism in cancer in KEGG pathway (Supplementary Table 3).

### Identification of miRNA-mRNA Regulatory Network in HCM

**Figure 3:**
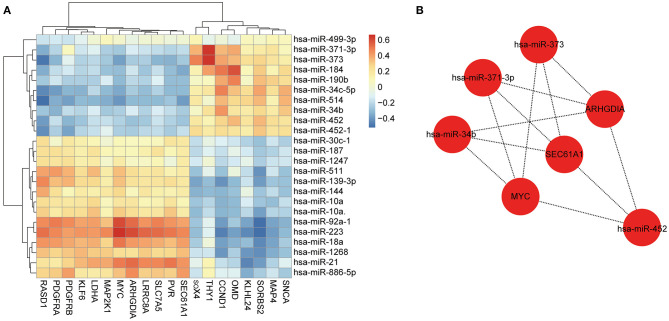
Construction of miRNA-mRNA regulatory network. **(A)** Heatmap of correlation between miRNAs and mRNAs. Red represents positive correlation, and blue represents negative correlation. **(B)** Constructed miRNA-mRNA regulatory network with four miRNAs and three genes, basing on MNC algorithm.

We assessed the correlation between the 24 DEMs and 20 overlapped mRNAs (Figure 3A), identified 225 notably negatively regulated miRNA-mRNA pairs (Supplementary Table 4), and constructed a regulatory network with 44 nodes and 225 lines (Supplementary Figure 3). Subsequently, we scored each node in the network by using MNC algorithm and screened out 10 nodes with high degree, among which seven nodes were able to constitute an effective regulatory network. As shown in Figure 3B, this network contained four miRNAs and three mRNAs, namely hsa-miR-373 (fold change: 2.574234354), hsa-miR-371-3p (fold change: 1.738309775), hsa-miR-34b (fold change: 1.563072937), hsa-miR-452 (fold change: 1.520314608), ARHGDIA (fold change: 1.5385129), SEC61A1 (fold change: 1.5979385), and MYC (fold change: 2.8495458). Given that these seven miRNAs and genes were differentially expressed between the HCM samples and control samples and the Cytoscape software and cytoHubba plug-in with MNC algorithm were important approaches for visualization of miRNA-mRNA regulatory network and identification of hub biomarkers from complex interactome (17, 18), it was speculated that the seven nodes were potential biomarkers associated with HCM. However, our study was only based on the negative correlation between miRNAs and their targeted mRNAs, which was relatively limited.

### Logistic Regression Model for Prediction of HCM

**Figure 4:**
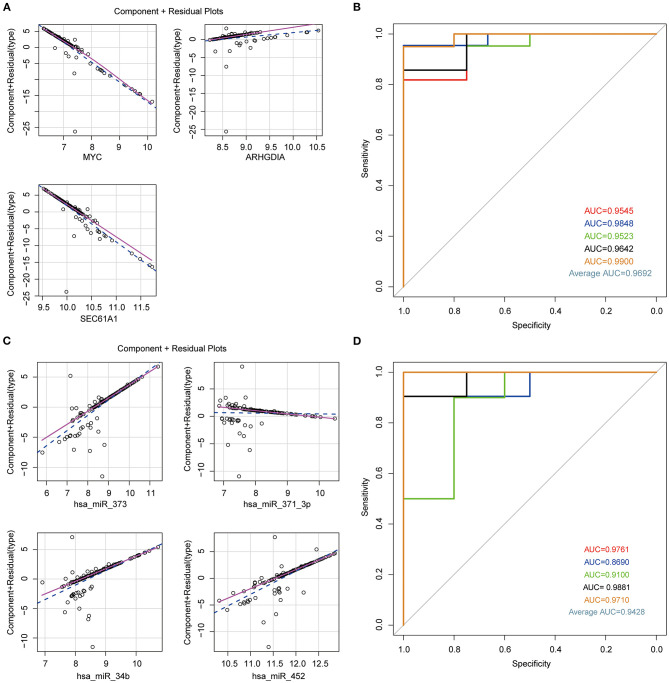
Establishment of logistic regression model. **(A)** Component plus residual plot of ARHGDIA, SEC61A1, and MYC in the established model. Linear correlation between independent variables and dependent variables indicated the feasibility. **(B)** The ROC curve of predicted outcomes of GSE36961 by logistic regression model. The *x* axis represents false-positive rate (FPR), and the *y* axis represents true-positive rate (TPR). **(C)** Component plus residual plot of hsa-miR-373, hsa-miR-371-3p, hsa-miR-34b, and hsa-miR-452 in the established model. **(D)** The ROC curve of predicted outcomes of GSE36946 by logistic regression model.

In order to confirm the diagnostic role of above biomarkers in HCM, we constructed logistic models in GSE36946 and GSE36961 datasets basing on these four miRNAs and three mRNAs, separately. We used the miRNAs and mRNAs as variable and testified the normal distribution of data, which had no influential points (Supplementary Figures 4A–D). The linear correlation between independent and dependent variables suggested the rational application of the three mRNAs and four miRNAs in the model (Figures 4A,C). Furthermore, we applied five-fold crossvalidation to assess the reliability of established models as follows, we first randomly divided the samples into five portions, adopted four portions as the training set to construct logistic regression model and the left one to validate the model, and then repeated this process for five times to reduce the error and enhance the sensitivity of the models. The AUC values of the five models in mRNA dataset GSE36961 were 0.9545, 0.9848 0.9523, 0.9642, and 0.9900, suggesting the good explanatory power of the model (Figure 4B). Similarly, the AUC values of the miRNA dataset GSE36946 were 0.9761, 0.8690, 0.9100, 0.9881, and 0.9710 (Figure 4D). Taken together, the logistic regression model established basing on the four miRNAs or the three mRNAs could effectively identify sample type (HCM/healthy control), and hsa-miR-373, hsa-miR-371-3p, hsa-miR-34b, hsa-miR-452, ARHGDIA, SEC61A1, and MYC were potential targets for HCM study.

## Discussion

Despite the significant advances in the knowledge of HCM, its pathogenesis is still unclear (19). Genetic studies revealed that gene mutations associated with sarcomere, such as β-myosin heavy chain and myosin-binding protein C genes, were closely involved in HCM (1, 20). However, the correlation between genotype and phenotype is highly variable, which makes the diagnosis solely by sarcomere variants unreliable in clinical practice (21). To explore novel diagnostic biomarkers, we evaluated the DEGs and DEMs between the HCM samples and healthy controls from GEO database and conducted functional enrichment analysis. We found GO terms such as extracellular structure organization, regeneration, regulation of wound healing, organ growth, response to hypoxia, and platelet-derived growth factor receptor (PDGFR) signaling pathway in top 20 DEG- and DEM-enriched GO terms. Myocardial fibrosis is frequently seen in HCM, manifested by diverse levels of replacement fibrosis and interstitial fibrosis, and is closely associated with the degree of HCM (22, 23). The fibrosis in HCM is manifested by disorganized collagen, a principal component of extracellular structure organization, and is commonly seen in the process of wound healing (23). Dysfunction of coronary microvasculature is also a major change in heart during HCM and myocardial fibrosis (24, 25). PDGFR is widely studied in multiple pathological processes such as cancer, fibrosis, and atherosclerosis and functions through promoting cell proliferation, angiogenesis, and many other cell behaviors; therefore, it is regarded as an ideal candidate for drug development (26, 27). Moreover, PDGFR was previously reported to regulate proliferation and deposition of collagen in cardiac fibroblasts, as well as hypertrophy of myocardium (28). Mirtschink et al. (29) recently suggested that inhibiting hypoxia signaling could improve heart failure-caused death. Besides, hypoxia signaling frequently participates in the regulation of fibrosis and vascular remodeling in various diseases (30, 31).

In our work, a regulatory network consisting of four miRNAs (hsa-miR-373, hsa-miR-371-3p, hsa-miR-34b, and hsa-miR-452) and three mRNAs (ARHGDIA, SEC61A1, MYC) was identified to be involved in the development of HCM. The logistic regression models based on these hub genes or miRNAs exhibited excellent efficacy in the diagnosis of HCM, which was manifested by the high AUC values. These results indicated the potential diagnostic role of this network in HCM. Among these miRNAs, circulating hsa-miR-373 was previously revealed as a potential biomarker for diffuse myocardial fibrosis in HCM (13). hsa-miR-34b belongs to the miR-34 family, whose expression level is increased in the cardiac tissues of patients with heart disease (32). The silence of miR-34 family members exerts a protective role in heart against pathological cardiac remodeling, and hsa-miR-34a silencing even improves the cardiac dysfunction in moderate HCM (33, 34). hsa-miR-371-3p is a promising signature in cancer (35). Besides, Cakmak et al. (36) indicated the reduced expression of hsa-miR-371-3p in chronic congestive heart failure, one of the cardiovascular disorders, suggesting the implication of hsa-miR-371-3p in heart function. hsa-miR-452 is also associated with various cancers. For example, hsa-miR-452 is considered a potential biomarker for the diagnosis and stratification of bladder cancer (37), and its upregulation suppresses the metastasis of nonsmall cell lung cancer (38). However, the function of hsa-miR-371-3p and hsa-miR-452 has not been clarified in HCM, yet the regulatory network demonstrated the same targets of them as of hsa-miR-373, namely ARHGDIA, SEC61A1, and MYC. MYC is a well-known transcription factor and was shown to regulate cell cycle, angiogenesis, and oxidative stress during cardiomyogenesis (39). Increasing studies have suggested elevated level of MYC in HCM, consequently promoting cardiomyopathy and heart failure (40). ARHGDIA, also known as Rho GDP dissociation inhibitor α, is in complex with Rho GTPases (41); meanwhile, it is known that activated Rho signaling regulates growth of hypertrophic cardiac muscle cells and promotes cardiac myocyte hypertrophy (42, 43). Besides, SEC61A1 is able to govern endoplasmic reticulum (ER) function and calcium translocation, affecting fibrotic responses in cardiac fibroblasts (44). These previous studies supported the practical application of this miRNA-mRNA regulatory network for diagnosis of HCM. However, there are still some limitations in our study. (1) Although the established logistic regression models based on the four miRNAs or three mRNAs exhibited good performance in distinguishing the HCM samples from the healthy controls, it would be more applicable to integrate these two models into one in the future for their pervasive applications in clinical diagnosis. (2) Our study was only based on the negative correlation between miRNAs and their targeted mRNAs, which was restrictive and limited. (3) The miRNA-target interactions were predicted by the miRTarBase database, lacking of experimental validation. Further experimental investigation will be helpful to further verify their interactions.

## Conclusions

To summarize, our present bioinformatic analysis revealed a potential miRNA-mRNA regulatory network consisting of hsa-miR-373, hsa-miR-371-3p, hsa-miR-34b, hsa-miR-452, ARHGDIA, SEC61A1, and MYC. The identified miRNAs and mRNAs have the potential to be used as biomarkers in HCM and might provide novel insight into diagnosis and clinical therapy. However, more studies are needed for their pervasive applications in clinical diagnosis of HCM.

## Data Availability Statement

Publicly available datasets were analyzed in this study. This data can be found here: Gene Expression Omnibus; GSE36961, GSE36946.

## Author Contributions

LW designed this research. FL collected data. JX analyzed data. FL and JX wrote the manuscript. All authors read and approved the final manuscript.

## Conflict of Interest

The authors declare that the research was conducted in the absence of any commercial or financial relationships that could be construed as a potential conflict of interest.

## Supplementary Material

Supplementary Figure 1 Standardization of GEO data. The distribution of standardized samples from GSE36961 **(A)** and GSE36946 **(B)** dataset. The *x* axis represents samples, and the *y* axis represents expression values of mRNAs or miRNAs.

Supplementary Figure 2 Venn diagram of overlapped terms. Venn diagram of overlapped genes **(A)**, GO terms **(B)**, and KEGG pathways **(C)** between DEM-targeted genes in GSE36946 and DEGs in GSE36961.

Supplementary Figure 3 miRNA-mRNA regulatory network. Each dot represents one miRNA or mRNA, and each line represents one miRNA-mRNA pair.

Supplementary Figure 4 Diagnostic plot to detect the accuracy of logistic regression model. **(A)** Normal Q–Q plot of logistic model basing on ARHGDIA, SEC61A1, and MYC. Large deviation of dots from the 45° line suggests violation of normal distribution. **(B)** Plot of residuals vs. leverage. The dotted red line represents COOK's distance, which indicates influential point with values larger than 0.5 and would affect the accuracy of the logistic regression model. **(C,D)** Normal Q–Q plot **(C)** and residuals vs. leverage plot **(D)** of logistic model basing on hsa-miR-373, hsa-miR-371-3p, hsa-miR-34b, and hsa-miR-452.

Supplementary Table 1 Functional enrichment of 590 differentially expressed genes (DEGs).

Supplementary Table 2 Functional enrichment of targeted genes of 24 differentially expressed miRNAs (DEMs).

Supplementary Table 3 Overlapped GO terms and KEGG pathways in Supplementary Tables 1, 2.

Supplementary Table 4 Negatively regulated miRNA-mRNA pairs of 24 DEMs and 20 overlapped mRNAs.
