# Supplementary figures and images for: Identification of Potential miRNA-mRNA Regulatory Network Contributing to Hypertrophic Cardiomyopathy (HCM)
Source: Front Cardiovasc Med. 2021 May 31;8:660372. doi: 10.3389/fcvm.2021.660372 (PMC8200816; doi:10.3389/fcvm.2021.660372)

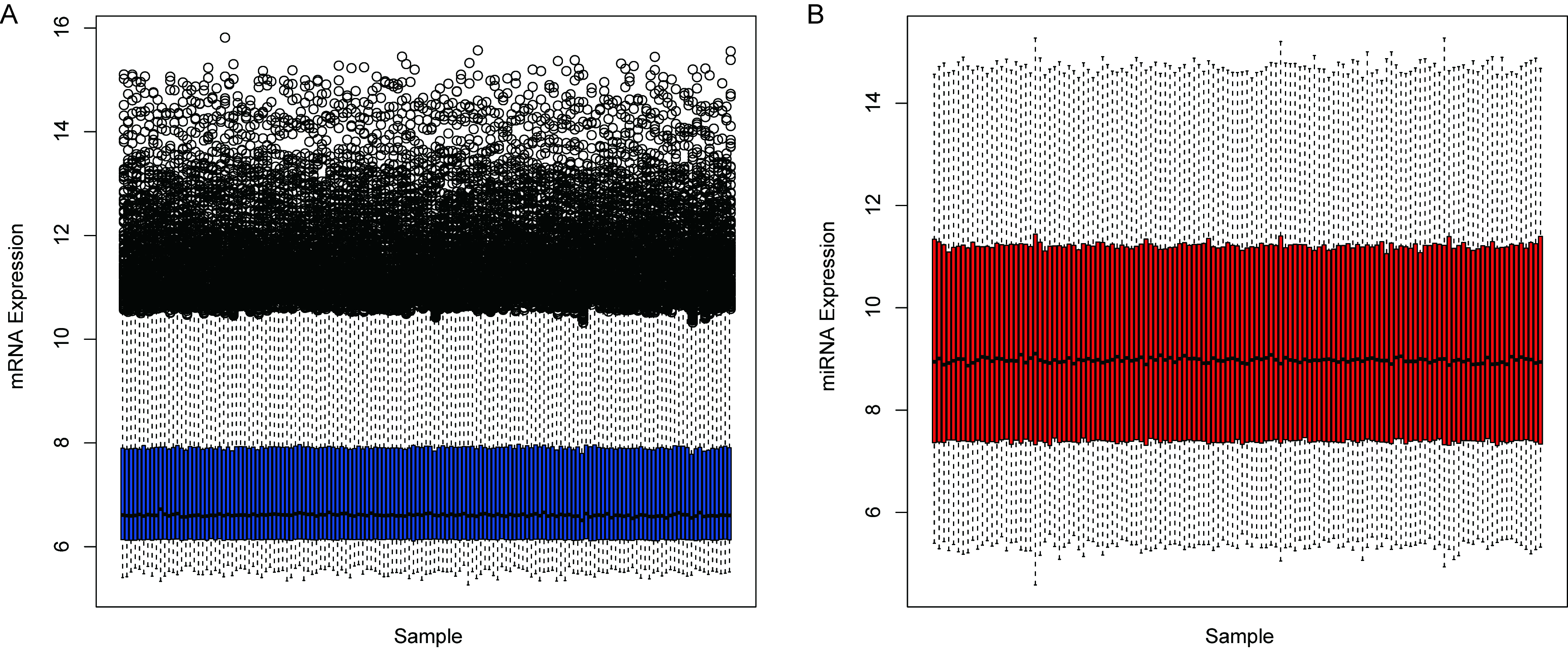

Supplement: Supplementary Figure 1 — Standardization of GEO data. The distribution of standardized samples from GSE36961 (A) and GSE36946 (B) dataset. The x axis represents samples, and the y axis represents expression values of mRNAs or miRNAs. [file Image_1.TIF]

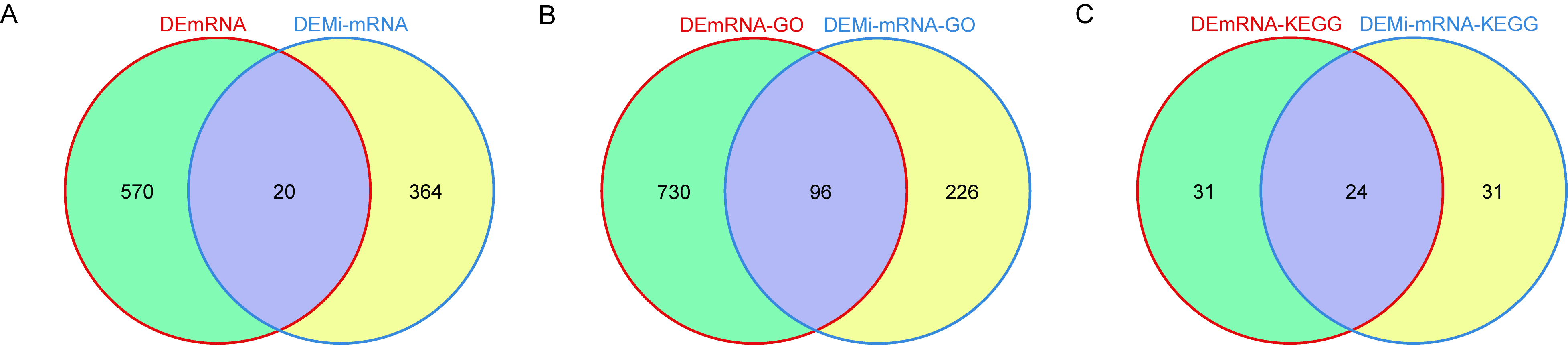

Supplement: Supplementary Figure 2 — Venn diagram of overlapped terms. Venn diagram of overlapped genes (A), GO terms (B), and KEGG pathways (C) between DEM-targeted genes in GSE36946 and DEGs in GSE36961. [file Image_2.TIF]

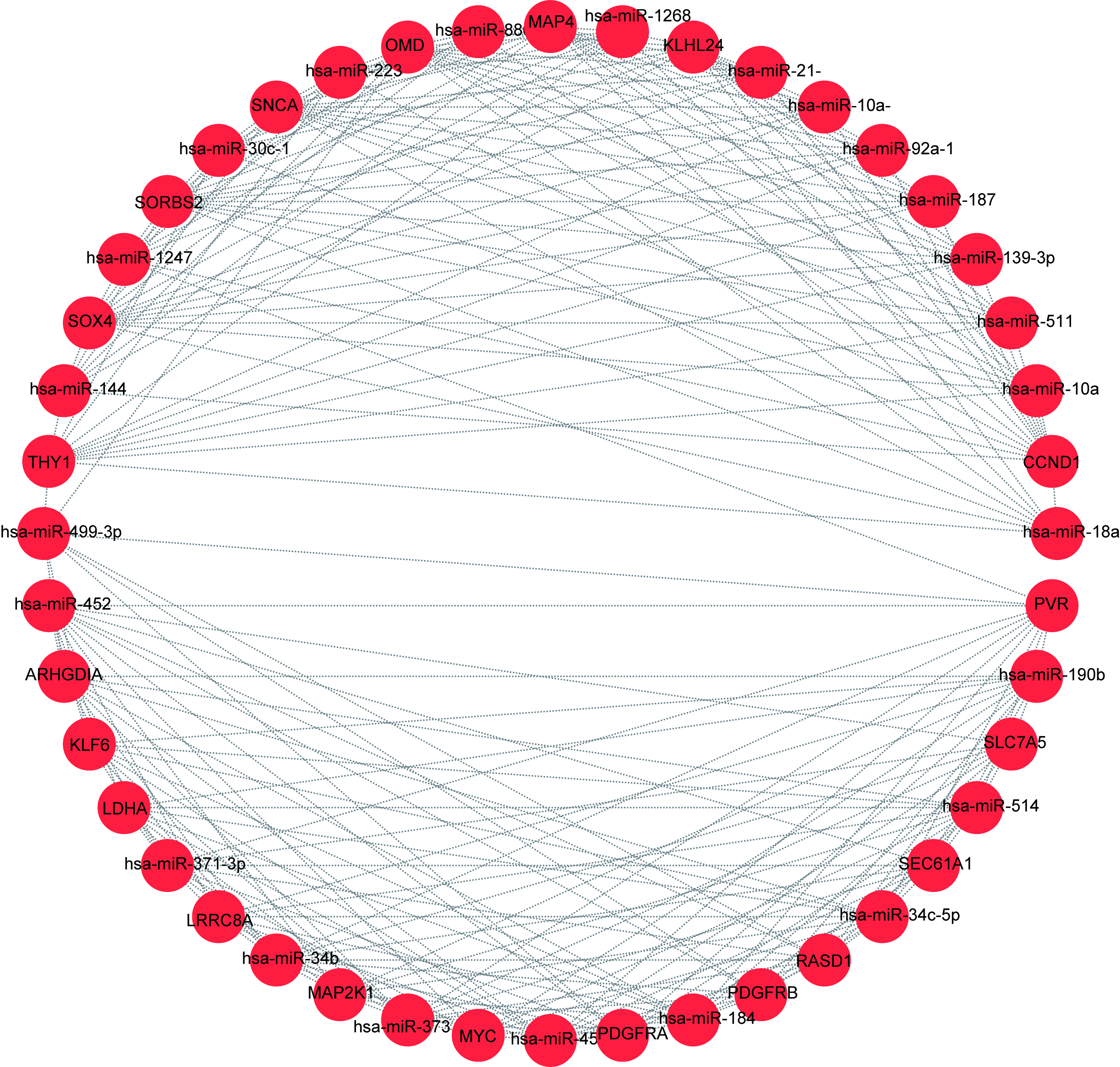

Supplement: Supplementary Figure 3 — miRNA-mRNA regulatory network. Each dot represents one miRNA or mRNA, and each line represents one miRNA-mRNA pair. [file Image_3.TIF]

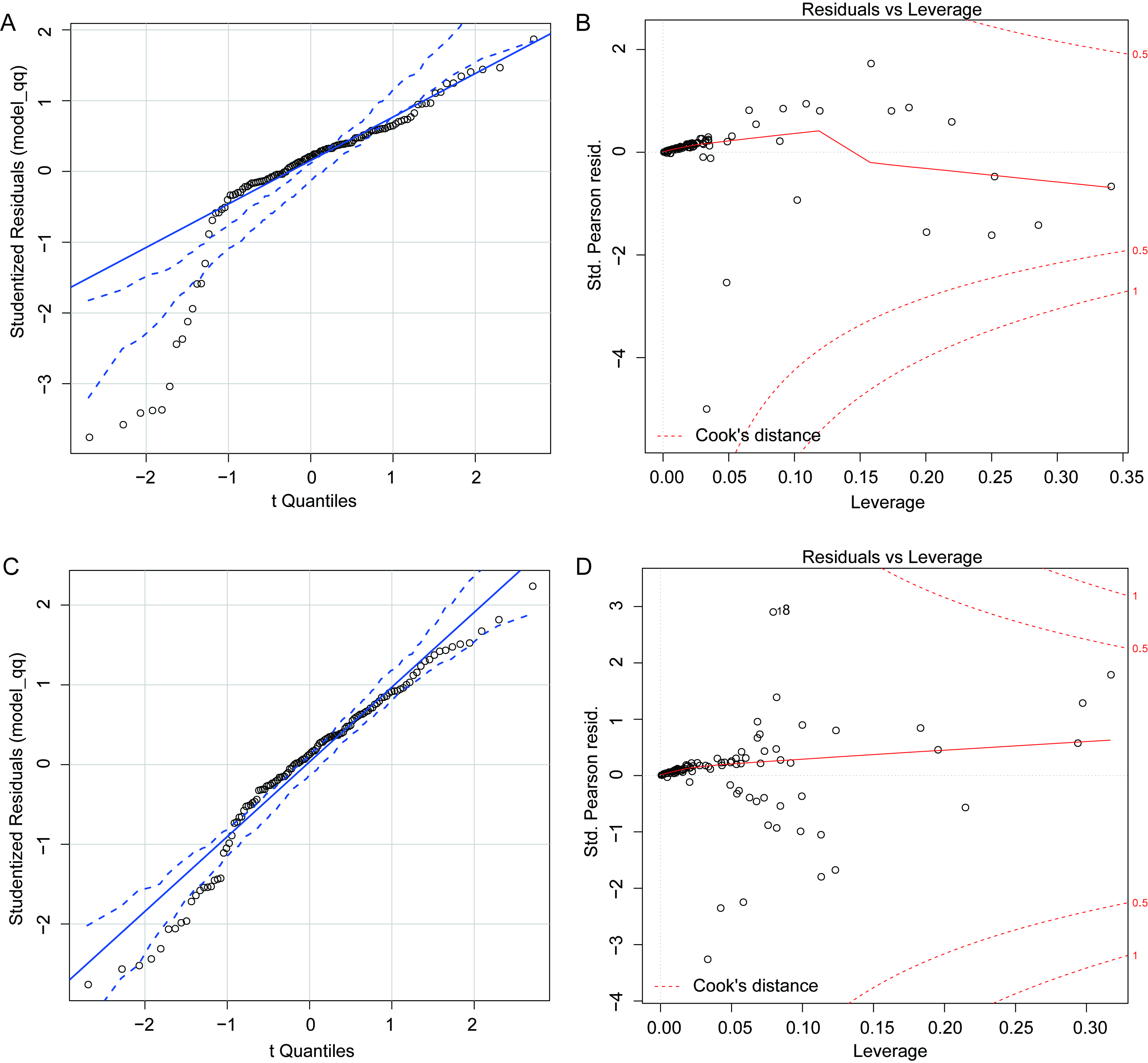

Supplement: Supplementary Figure 4 — Diagnostic plot to detect the accuracy of logistic regression model. (A) Normal Q–Q plot of logistic model basing on ARHGDIA, SEC61A1, and MYC. Large deviation of dots from the 45° line suggests violation of normal distribution. (B) Plot of residuals vs. leverage. The dotted red line represents COOK's distance, which indicates influential point with values larger than 0.5 and would affect the accuracy of the logistic regression model. (C,D) Normal Q–Q plot (C) and residuals vs. leverage plot (D) of logistic model basing on hsa-miR-373, hsa-miR-371-3p, hsa-miR-34b, and hsa-miR-452. [file Image_4.TIF]
