# Supplementary material for: Identification of Potential miRNA-mRNA Regulatory Network Contributing to Hypertrophic Cardiomyopathy (HCM)
Source: Front Cardiovasc Med. 2021 May 31;8:660372. doi: 10.3389/fcvm.2021.660372 (PMC8200816; doi:10.3389/fcvm.2021.660372)
Supplement: Supplementary Table 1 — Functional enrichment of 590 differentially expressed genes (DEGs). [file Table_1.DOCX]

Table S1 Functional enrichment of 590 differentially expressed genes (DEGs).

GO terms

| ONTOLOGY | ID | Description | GeneRatio | BgRatio | pvalue | p.adjust | qvalue | geneID | Count |
| --- | --- | --- | --- | --- | --- | --- | --- | --- | --- |
| BP | GO:0043062 | extracellular structure organization | 40/473 | 395/17653 | 5.01E-13 | 2.26E-09 | 1.65E-09 | ITGB2/SMOC2/LCP1/CAPN1/PLTP/BCL3/PHLDB2/SERPINH1/COL6A2/HTRA1/COL16A1/LUM/PLA2G2A/ECM2/SERPINE1/THBS4/ADAMTS9/FMOD/SPP1/APOA1/OLFML2A/ITGA5/CTSK/COL6A3/ANXA2/CRISPLD2/MFAP4/CTSG/TIMP1/APP/LIPG/SULF1/CD44/CYR61/TNC/COMP/THBS1/CYP1B1/POSTN/APOE | 40 |
| BP | GO:0002446 | neutrophil mediated immunity | 44/473 | 500/17653 | 4.21E-12 | 8.09E-09 | 5.90E-09 | SERPINA3/S100A9/PRKCD/FPR1/FCER1G/S100A8/ALOX5/CTSC/ITGB2/MGST1/CYBA/DOCK2/CD14/CD68/VAMP8/CAPN1/MVP/TUBB/FCGR2A/FGR/TYROBP/DDOST/GSDMD/CYBB/TMBIM1/ASAH1/PLAU/CD59/RNASE2/FCN1/CFD/S100A11/S100A12/CAP1/HSPA6/SERPINB1/BST2/ANXA2/CRISPLD2/CTSG/IL6/LYZ/SLC2A3/CD44 | 44 |
| BP | GO:0043312 | neutrophil degranulation | 43/473 | 485/17653 | 5.85E-12 | 8.09E-09 | 5.90E-09 | SERPINA3/S100A9/PRKCD/FPR1/FCER1G/S100A8/ALOX5/CTSC/ITGB2/MGST1/CYBA/DOCK2/CD14/CD68/VAMP8/CAPN1/MVP/TUBB/FCGR2A/FGR/TYROBP/DDOST/GSDMD/CYBB/TMBIM1/ASAH1/PLAU/CD59/RNASE2/FCN1/CFD/S100A11/S100A12/CAP1/HSPA6/SERPINB1/BST2/ANXA2/CRISPLD2/CTSG/LYZ/SLC2A3/CD44 | 43 |
| BP | GO:0002283 | neutrophil activation involved in immune response | 43/473 | 488/17653 | 7.17E-12 | 8.09E-09 | 5.90E-09 | SERPINA3/S100A9/PRKCD/FPR1/FCER1G/S100A8/ALOX5/CTSC/ITGB2/MGST1/CYBA/DOCK2/CD14/CD68/VAMP8/CAPN1/MVP/TUBB/FCGR2A/FGR/TYROBP/DDOST/GSDMD/CYBB/TMBIM1/ASAH1/PLAU/CD59/RNASE2/FCN1/CFD/S100A11/S100A12/CAP1/HSPA6/SERPINB1/BST2/ANXA2/CRISPLD2/CTSG/LYZ/SLC2A3/CD44 | 43 |
| BP | GO:0030198 | extracellular matrix organization | 35/473 | 341/17653 | 1.04E-11 | 9.35E-09 | 6.82E-09 | ITGB2/SMOC2/LCP1/CAPN1/BCL3/PHLDB2/SERPINH1/COL6A2/HTRA1/COL16A1/LUM/ECM2/SERPINE1/THBS4/ADAMTS9/FMOD/SPP1/OLFML2A/ITGA5/CTSK/COL6A3/ANXA2/CRISPLD2/MFAP4/CTSG/TIMP1/APP/SULF1/CD44/CYR61/TNC/COMP/THBS1/CYP1B1/POSTN | 35 |
| BP | GO:0042119 | neutrophil activation | 43/473 | 498/17653 | 1.40E-11 | 1.05E-08 | 7.66E-09 | SERPINA3/S100A9/PRKCD/FPR1/FCER1G/S100A8/ALOX5/CTSC/ITGB2/MGST1/CYBA/DOCK2/CD14/CD68/VAMP8/CAPN1/MVP/TUBB/FCGR2A/FGR/TYROBP/DDOST/GSDMD/CYBB/TMBIM1/ASAH1/PLAU/CD59/RNASE2/FCN1/CFD/S100A11/S100A12/CAP1/HSPA6/SERPINB1/BST2/ANXA2/CRISPLD2/CTSG/LYZ/SLC2A3/CD44 | 43 |
| BP | GO:0031099 | regeneration | 24/473 | 186/17653 | 1.91E-10 | 1.23E-07 | 8.99E-08 | JAK2/LCP1/NFKBIA/NNMT/CEBPB/CCND1/RGMA/KLF4/MAP2K1/TGFBR3/MATN2/PRRX1/SPP1/APOA1/GJA1/SOX15/CDKN1A/HAMP/MUSTN1/TNC/PTPN3/THY1/POSTN/APOE | 24 |
| BP | GO:0003012 | muscle system process | 38/473 | 450/17653 | 4.31E-10 | 2.43E-07 | 1.77E-07 | MYH6/SCN2B/SORBS2/LMOD2/CYBA/ACE2/SORBS1/PDE5A/PLCE1/EDNRA/ERRFI1/ENO1/ATP1A2/GATM/ATP1A1/AIF1/TNNT1/KLF4/ATP2A2/KCNA5/LMCD1/PDE4D/SSPN/KCNJ2/GJA1/RGS2/TPM3/RYR2/CASQ1/HAMP/SULF1/HEY2/MYL7/TNNI3K/CNN1/MYOT/FGF12/NPPA | 38 |
| BP | GO:1903034 | regulation of response to wounding | 21/473 | 161/17653 | 2.16E-09 | 1.08E-06 | 7.92E-07 | S100A9/PRKCD/FCER1G/PROS1/PLEK/C1QTNF1/PHLDB2/RGMA/SERPINE1/PLAU/PRKCQ/KLF4/PDGFRA/MAP2K1/SPP1/GJA1/SOX15/ANXA2/HBEGF/THBS1/APOE | 21 |
| BP | GO:0032496 | response to lipopolysaccharide | 30/473 | 327/17653 | 4.71E-09 | 2.12E-06 | 1.55E-06 | ZFP36/S100A8/JAK2/HCK/MGST1/NFKBIA/CEBPB/CD14/HAVCR2/IRAK3/LTBR/TNFRSF1A/HMGB2/SERPINE1/CCL2/LY96/CXCL2/TIMP4/SNCA/PDE4D/TRIB1/GJA1/ALPL/FOS/CTSG/LITAF/IL6/JUNB/HAMP/PENK | 30 |
| BP | GO:0043405 | regulation of MAP kinase activity | 30/473 | 344/17653 | 1.52E-08 | 6.09E-06 | 4.44E-06 | MAP3K6/PRKCD/FPR1/JAK2/FGD2/MAP3K8/PDE5A/PLCE1/DUSP1/IRAK3/SFRP1/RGS4/GRM1/HGS/MDFIC/TPD52L1/S100A12/PDGFRB/GADD45G/MAP2K1/TRIB1/GADD45A/RGS2/PTPN1/GADD45B/UCHL1/THBS1/DUSP5/FGF18/APOE | 30 |
| BP | GO:0002237 | response to molecule of bacterial origin | 30/473 | 345/17653 | 1.62E-08 | 6.09E-06 | 4.44E-06 | ZFP36/S100A8/JAK2/HCK/MGST1/NFKBIA/CEBPB/CD14/HAVCR2/IRAK3/LTBR/TNFRSF1A/HMGB2/SERPINE1/CCL2/LY96/CXCL2/TIMP4/SNCA/PDE4D/TRIB1/GJA1/ALPL/FOS/CTSG/LITAF/IL6/JUNB/HAMP/PENK | 30 |
| BP | GO:0060047 | heart contraction | 26/473 | 275/17653 | 2.77E-08 | 9.61E-06 | 7.01E-06 | MYH6/JAK2/SCN2B/CYP2J2/ACE2/PDE5A/ATP1A2/ATP1A1/TNNT1/ATP2A2/KCNA5/ITPR3/PDE4D/KCNJ2/SGCG/GJA1/RGS2/ATP1B3/RYR2/CASQ1/HBEGF/CORIN/HEY2/TNNI3K/FGF12/NPPA | 26 |
| BP | GO:0003015 | heart process | 26/473 | 278/17653 | 3.45E-08 | 1.11E-05 | 8.12E-06 | MYH6/JAK2/SCN2B/CYP2J2/ACE2/PDE5A/ATP1A2/ATP1A1/TNNT1/ATP2A2/KCNA5/ITPR3/PDE4D/KCNJ2/SGCG/GJA1/RGS2/ATP1B3/RYR2/CASQ1/HBEGF/CORIN/HEY2/TNNI3K/FGF12/NPPA | 26 |
| BP | GO:0031589 | cell-substrate adhesion | 28/473 | 319/17653 | 4.02E-08 | 1.21E-05 | 8.83E-06 | LYVE1/JAK2/CORO1A/ITGB2/SMOC2/BCL6/S100A10/SORBS1/SFRP1/PHLDB2/ZYX/COL16A1/VWC2/ECM2/SERPINE1/PLAU/EMILIN1/RAC2/ADAMTS9/SPOCK1/APOA1/ID1/ITGA5/CD44/CYR61/THBS1/THY1/POSTN | 28 |
| BP | GO:0032103 | positive regulation of response to external stimulus | 26/473 | 289/17653 | 7.56E-08 | 2.13E-05 | 1.56E-05 | S100A9/FCER1G/S100A8/JAK2/NFKBIA/CYBA/VAMP8/HAVCR2/OSMR/TNFRSF1A/PLA2G2A/SERPINE1/AIF1/THBS4/RAC2/S100A12/PDGFRB/LY96/CXCL2/SNCA/LDLR/IL1RL1/IL6/THBS1/FGF18/NPPA | 26 |
| BP | GO:0072503 | cellular divalent inorganic cation homeostasis | 34/473 | 457/17653 | 8.51E-08 | 2.17E-05 | 1.58E-05 | S100A9/MT1M/S1PR3/FPR1/MT1X/S100A8/JAK2/CORO1A/MT1A/MT2A/CYBA/DDIT3/GNA15/PLCE1/C1QTNF1/EDNRA/ATP1A2/GPR4/GRM1/ATP2A2/KCNA5/ITPR3/PDGFRA/SNCA/PDE4D/GJA1/MT1G/RYR2/CASQ1/SLC30A2/APP/MT1E/THY1/APOE | 34 |
| BP | GO:0060048 | cardiac muscle contraction | 17/473 | 132/17653 | 8.97E-08 | 2.17E-05 | 1.58E-05 | MYH6/SCN2B/ACE2/PDE5A/ATP1A2/ATP1A1/TNNT1/ATP2A2/KCNA5/PDE4D/KCNJ2/GJA1/RGS2/RYR2/TNNI3K/FGF12/NPPA | 17 |
| BP | GO:0006936 | muscle contraction | 29/473 | 352/17653 | 9.13E-08 | 2.17E-05 | 1.58E-05 | MYH6/SCN2B/LMOD2/ACE2/SORBS1/PDE5A/PLCE1/EDNRA/ENO1/ATP1A2/ATP1A1/TNNT1/ATP2A2/KCNA5/PDE4D/SSPN/KCNJ2/GJA1/RGS2/TPM3/RYR2/CASQ1/SULF1/MYL7/TNNI3K/CNN1/MYOT/FGF12/NPPA | 29 |
| BP | GO:0061041 | regulation of wound healing | 17/473 | 134/17653 | 1.12E-07 | 2.52E-05 | 1.84E-05 | S100A9/PRKCD/FCER1G/PROS1/PLEK/C1QTNF1/PHLDB2/SERPINE1/PLAU/PRKCQ/PDGFRA/GJA1/SOX15/ANXA2/HBEGF/THBS1/APOE | 17 |
| BP | GO:0032970 | regulation of actin filament-based process | 30/473 | 377/17653 | 1.17E-07 | 2.52E-05 | 1.84E-05 | CDC42EP4/PRKCD/FES/CORO1A/ARPC1B/HCK/WAS/HCLS1/LMOD2/S100A10/PLEK/SFRP1/PHLDB2/ATP1A2/ARPC3/ATP1A1/SYNPO2L/RAC2/ATP2A2/PDGFRB/ARPC5L/ARHGDIA/PDGFRA/PDE4D/KCNJ2/APOA1/ID1/RYR2/BST2/PROX1 | 30 |
| BP | GO:0030193 | regulation of blood coagulation | 13/473 | 78/17653 | 1.40E-07 | 2.75E-05 | 2.01E-05 | S100A9/PRKCD/FCER1G/PROS1/PLEK/C1QTNF1/SERPINE1/PLAU/PRKCQ/PDGFRA/ANXA2/THBS1/APOE | 13 |
| BP | GO:1900046 | regulation of hemostasis | 13/473 | 78/17653 | 1.40E-07 | 2.75E-05 | 2.01E-05 | S100A9/PRKCD/FCER1G/PROS1/PLEK/C1QTNF1/SERPINE1/PLAU/PRKCQ/PDGFRA/ANXA2/THBS1/APOE | 13 |
| BP | GO:0055119 | relaxation of cardiac muscle | 7/473 | 17/17653 | 1.46E-07 | 2.75E-05 | 2.01E-05 | PDE5A/ATP1A2/ATP1A1/ATP2A2/PDE4D/KCNJ2/RGS2 | 7 |
| BP | GO:0008016 | regulation of heart contraction | 23/473 | 243/17653 | 1.73E-07 | 3.12E-05 | 2.28E-05 | MYH6/JAK2/SCN2B/CYP2J2/ACE2/PDE5A/ATP1A2/ATP1A1/ATP2A2/KCNA5/ITPR3/PDE4D/KCNJ2/GJA1/RGS2/ATP1B3/RYR2/CASQ1/HBEGF/CORIN/HEY2/TNNI3K/NPPA | 23 |
| BP | GO:0050729 | positive regulation of inflammatory response | 16/473 | 124/17653 | 2.09E-07 | 3.33E-05 | 2.43E-05 | S100A9/FCER1G/S100A8/JAK2/NFKBIA/VAMP8/OSMR/TNFRSF1A/PLA2G2A/SERPINE1/S100A12/SNCA/LDLR/IL1RL1/IL6/NPPA | 16 |
| BP | GO:0072507 | divalent inorganic cation homeostasis | 34/473 | 475/17653 | 2.13E-07 | 3.33E-05 | 2.43E-05 | S100A9/MT1M/S1PR3/FPR1/MT1X/S100A8/JAK2/CORO1A/MT1A/MT2A/CYBA/DDIT3/GNA15/PLCE1/C1QTNF1/EDNRA/ATP1A2/GPR4/GRM1/ATP2A2/KCNA5/ITPR3/PDGFRA/SNCA/PDE4D/GJA1/MT1G/RYR2/CASQ1/SLC30A2/APP/MT1E/THY1/APOE | 34 |
| BP | GO:0034341 | response to interferon-gamma | 20/473 | 191/17653 | 2.18E-07 | 3.33E-05 | 2.43E-05 | CDC42EP4/PRKCD/JAK2/IFITM2/HCK/WAS/IFITM3/MT2A/IFI30/SEC61A1/ZYX/AIF1/SOCS1/CCL2/SNCA/HLA-DRB4/BST2/IFITM1/IRF6/CD44 | 20 |
| BP | GO:0009612 | response to mechanical stimulus | 21/473 | 209/17653 | 2.19E-07 | 3.33E-05 | 2.43E-05 | BTG2/NFKBIA/CYBA/LTBR/SLC2A1/ATP1A2/TNFRSF1A/ATP1A1/PKD1L2/KCNA5/KCNJ2/GADD45A/GJA1/FOS/RYR2/JUNB/TNC/SLC38A2/THBS1/POSTN/NPPA | 21 |
| BP | GO:0031102 | neuron projection regeneration | 11/473 | 56/17653 | 2.28E-07 | 3.33E-05 | 2.43E-05 | JAK2/RGMA/KLF4/MAP2K1/MATN2/PRRX1/SPP1/APOA1/TNC/THY1/APOE | 11 |
| BP | GO:0050878 | regulation of body fluid levels | 35/473 | 499/17653 | 2.29E-07 | 3.33E-05 | 2.43E-05 | S100A9/PRKCD/FCER1G/JAK2/PROS1/WAS/F13A1/CYBA/HEG1/VAMP8/GNA15/PLEK/C1QTNF1/SERPINA5/CCND1/SERPINE1/PLAU/CD59/ADCY3/CEL/ACTB/PRKCQ/RAC2/TSPAN32/CLIC1/ITPR3/PDGFRA/NPR3/GJA1/ANXA2/NME1/SOCS2/IL6/THBS1/APOE | 35 |
| BP | GO:1902905 | positive regulation of supramolecular fiber organization | 20/473 | 195/17653 | 3.05E-07 | 4.30E-05 | 3.14E-05 | CDC42EP4/FES/CORO1A/ARPC1B/HCK/WAS/LMOD2/S100A10/PLEK/SFRP1/ARPC3/SYNPO2L/RAC2/ARPC5L/APOA1/RGS2/ID1/PROX1/APP/APOE | 20 |
| BP | GO:0050818 | regulation of coagulation | 13/473 | 84/17653 | 3.44E-07 | 4.71E-05 | 3.44E-05 | S100A9/PRKCD/FCER1G/PROS1/PLEK/C1QTNF1/SERPINE1/PLAU/PRKCQ/PDGFRA/ANXA2/THBS1/APOE | 13 |
| BP | GO:1903035 | negative regulation of response to wounding | 13/473 | 85/17653 | 3.97E-07 | 5.27E-05 | 3.84E-05 | PRKCD/PROS1/C1QTNF1/PHLDB2/RGMA/SERPINE1/PLAU/PDGFRA/SPP1/GJA1/ANXA2/THBS1/APOE | 13 |
| BP | GO:0031349 | positive regulation of defense response | 33/473 | 466/17653 | 4.11E-07 | 5.30E-05 | 3.87E-05 | S100A9/PRKCD/FCER1G/S100A8/JAK2/HCK/ITGB2/NFKBIA/CYBA/CD14/VAMP8/HAVCR2/CD209/OSMR/IRAK3/MARCO/TNFRSF1A/HMGB2/PLA2G2A/SERPINE1/FCN1/IFI16/S100A12/LY96/SNCA/PVR/LDLR/GJA1/CTSK/IL1RL1/IL6/PENK/NPPA | 33 |
| BP | GO:0006882 | cellular zinc ion homeostasis | 9/473 | 38/17653 | 5.39E-07 | 6.75E-05 | 4.93E-05 | S100A9/MT1M/MT1X/S100A8/MT1A/MT2A/MT1G/SLC30A2/MT1E | 9 |
| BP | GO:0090257 | regulation of muscle system process | 22/473 | 242/17653 | 6.41E-07 | 7.81E-05 | 5.70E-05 | ACE2/PDE5A/PLCE1/ERRFI1/ENO1/ATP1A2/ATP1A1/FBXO32/AIF1/TNNT1/KLF4/ATP2A2/LMCD1/PDE4D/KCNJ2/RGS2/RYR2/CASQ1/HAMP/TNNI3K/CNN1/NPPA | 22 |
| BP | GO:1903779 | regulation of cardiac conduction | 12/473 | 75/17653 | 6.73E-07 | 7.99E-05 | 5.83E-05 | ACE2/ATP1A2/ATP1A1/ATP2A2/ITPR3/PDE4D/ATP1B3/RYR2/CASQ1/CORIN/TNNI3K/NPPA | 12 |
| BP | GO:0050727 | regulation of inflammatory response | 30/473 | 411/17653 | 7.45E-07 | 8.53E-05 | 6.22E-05 | S100A9/PRKCD/C1R/FCER1G/S100A8/JAK2/PROS1/HCK/BCL6/NFKBIA/C1QC/C1QB/VAMP8/ACE2/OSMR/TNFRSF1A/ZYX/PLA2G2A/SERPINE1/CD59/KLF4/S100A12/SNCA/LDLR/APOA1/IL1RL1/IL6/APOE/C6/NPPA | 30 |
| BP | GO:0031032 | actomyosin structure organization | 19/473 | 188/17653 | 7.56E-07 | 8.53E-05 | 6.22E-05 | MYH6/WAS/LMOD2/S100A10/SORBS1/SFRP1/PHLDB2/FRMD5/ZYX/TNNT1/FRMD3/PDGFRB/PDGFRA/APOA1/MYOZ3/MYOZ2/CASQ1/PROX1/CNN1 | 19 |
| BP | GO:0055069 | zinc ion homeostasis | 9/473 | 40/17653 | 8.62E-07 | 9.48E-05 | 6.92E-05 | S100A9/MT1M/MT1X/S100A8/MT1A/MT2A/MT1G/SLC30A2/MT1E | 9 |
| BP | GO:0071496 | cellular response to external stimulus | 25/473 | 307/17653 | 8.83E-07 | 9.48E-05 | 6.92E-05 | PIM1/CYBA/SFRP1/LTBR/PLEKHF1/SLC2A1/ATP1A2/TNFRSF1A/ATP1A1/GSDMD/CYBB/AIF1/PDK4/IFI16/KCNJ2/GADD45A/GJA1/FOS/CDKN1A/ATF3/TNC/SLC38A2/PENK/POSTN/NPPA | 25 |
| BP | GO:0010810 | regulation of cell-substrate adhesion | 19/473 | 191/17653 | 9.63E-07 | 9.90E-05 | 7.22E-05 | JAK2/SMOC2/BCL6/S100A10/SFRP1/PHLDB2/COL16A1/VWC2/ECM2/SERPINE1/PLAU/RAC2/SPOCK1/APOA1/ITGA5/CYR61/THBS1/THY1/POSTN | 19 |
| BP | GO:0006941 | striated muscle contraction | 18/473 | 173/17653 | 9.65E-07 | 9.90E-05 | 7.22E-05 | MYH6/SCN2B/ACE2/PDE5A/ATP1A2/ATP1A1/TNNT1/ATP2A2/KCNA5/PDE4D/KCNJ2/GJA1/RGS2/RYR2/CASQ1/TNNI3K/FGF12/NPPA | 18 |
| BP | GO:2000379 | positive regulation of reactive oxygen species metabolic process | 13/473 | 92/17653 | 1.01E-06 | 0.0001014 | 7.40E-05 | PRKCD/JAK2/ITGB2/CYBA/ACE2/AIF1/KLF4/PDGFRB/SNCA/GADD45A/KLF2/CDKN1A/THBS1 | 13 |
| BP | GO:0060326 | cell chemotaxis | 23/473 | 273/17653 | 1.35E-06 | 0.0001326 | 9.67E-05 | S100A9/PRKCD/FPR1/FCER1G/S100A8/CORO1A/ITGB2/HMGB2/SERPINE1/PRKCQ/AIF1/THBS4/RAC2/S100A12/PDGFRB/CCL2/CXCL2/PDGFRA/PDE4D/HBEGF/IL6/THBS1/FGF18 | 23 |
| BP | GO:0010273 | detoxification of copper ion | 6/473 | 15/17653 | 1.46E-06 | 0.0001358 | 9.91E-05 | MT1M/MT1X/MT1A/MT2A/MT1G/MT1E | 6 |
| BP | GO:1990169 | stress response to copper ion | 6/473 | 15/17653 | 1.46E-06 | 0.0001358 | 9.91E-05 | MT1M/MT1X/MT1A/MT2A/MT1G/MT1E | 6 |
| BP | GO:0061337 | cardiac conduction | 16/473 | 143/17653 | 1.47E-06 | 0.0001358 | 9.91E-05 | SCN2B/ACE2/ATP1A2/ATP1A1/ATP2A2/KCNA5/ITPR3/PDE4D/KCNJ2/GJA1/ATP1B3/RYR2/CASQ1/CORIN/TNNI3K/NPPA | 16 |
| BP | GO:0002683 | negative regulation of immune system process | 30/473 | 426/17653 | 1.56E-06 | 0.0001405 | 0.0001025 | ZFP36/FCER1G/MYC/MAFB/BCL6/NFKBIA/C1QC/CEBPB/CD14/HAVCR2/PDE5A/IRAK3/SFRP1/HTRA1/CD59/IFI16/GPNMB/TSPAN32/CCL2/LY96/LRRC32/TRIB1/LDLR/APOA1/BST2/IL1RL1/NME1/IFIT1/THBS1/THY1 | 30 |
| BP | GO:0043500 | muscle adaptation | 14/473 | 111/17653 | 1.59E-06 | 0.0001405 | 0.0001025 | MYH6/CYBA/PDE5A/ERRFI1/GATM/AIF1/TNNT1/KLF4/ATP2A2/LMCD1/RGS2/HAMP/HEY2/NPPA | 14 |
| BP | GO:0007229 | integrin-mediated signaling pathway | 13/473 | 97/17653 | 1.87E-06 | 0.0001622 | 0.0001184 | FCER1G/HCK/ITGB2/PLEK/FGR/TYROBP/ZYX/COL16A1/TSPAN32/APOA1/ITGA5/TIMP1/THY1 | 13 |
| BP | GO:1901654 | response to ketone | 18/473 | 183/17653 | 2.18E-06 | 0.0001834 | 0.0001338 | CYBA/DDIT4/DUSP1/SFRP1/CCND1/ERRFI1/CYBB/FBXO32/ADCY3/KLF4/GABRB1/TGFBR3/SPP1/FOS/KLF2/CDKN1A/NME1/THBS1 | 18 |
| BP | GO:0046916 | cellular transition metal ion homeostasis | 14/473 | 114/17653 | 2.20E-06 | 0.0001834 | 0.0001338 | S100A9/MT1M/MYC/MT1X/S100A8/MT1A/MT2A/HEPH/MT1G/SLC30A2/APP/MT1E/HAMP/TFRC | 14 |
| BP | GO:0001819 | positive regulation of cytokine production | 29/473 | 411/17653 | 2.25E-06 | 0.0001848 | 0.0001348 | FCER1G/JAK2/IL18R1/CYBA/HEG1/CEBPB/CD14/MBP/HAVCR2/DDIT3/FGR/BCL3/GSDMD/CYBB/LUM/HMGB2/SERPINE1/PRKCQ/FCN1/IFI16/LY96/PDE4D/IL1RL1/IL6/SULF1/THBS1/CYP1B1/POSTN/EGR1 | 29 |
| BP | GO:0033673 | negative regulation of kinase activity | 23/473 | 283/17653 | 2.50E-06 | 0.0002014 | 0.0001469 | PRKCD/PIK3IP1/MVP/MIDN/DUSP1/IRAK3/SFRP1/DUSP26/RGS4/ERRFI1/SOCS1/WARS/TRIB1/GADD45A/RGS2/CDKN1A/SOCS2/PTPN1/GADD45B/UCHL1/DUSP5/THY1/APOE | 23 |
| BP | GO:0046677 | response to antibiotic | 25/473 | 326/17653 | 2.63E-06 | 0.0002079 | 0.0001517 | PRKCD/S100A8/JAK2/STAT3/CD14/DUSP1/CCND1/CYBB/ADCY3/LDHA/KLF4/KCNA5/ID3/PDGFRB/OXCT1/ID1/ALPL/KLF2/CA3/IL6/HAMP/TNC/PENK/CYP1B1/EGR1 | 25 |
| BP | GO:0045926 | negative regulation of growth | 21/473 | 244/17653 | 2.78E-06 | 0.0002159 | 0.0001575 | MT1M/MT1X/MT1A/BCL6/MT2A/TSPYL2/FRZB/SEMA4B/DCUN1D3/SFRP1/ENO1/SPP1/GJA1/WWC1/MT1G/BST2/CDKN1A/CTSG/SOCS2/MT1E/NPPA | 21 |
| BP | GO:0030099 | myeloid cell differentiation | 28/473 | 394/17653 | 2.94E-06 | 0.000222 | 0.000162 | ZFP36/MYC/MAFB/JAK2/FES/STAT3/HCLS1/BCL6/NFKBIA/C1QC/CEBPB/SFRP1/LTBR/TYROBP/HMGB2/PRKCQ/IFI16/TGFBR3/TRIB1/MT1G/FOS/KLF2/ANXA2/NME1/APP/JUNB/THBS1/TFRC | 28 |
| BP | GO:0071280 | cellular response to copper ion | 7/473 | 25/17653 | 3.00E-06 | 0.000222 | 0.000162 | MT1M/MT1X/MT1A/MT2A/SNCA/MT1G/MT1E | 7 |
| BP | GO:0032640 | tumor necrosis factor production | 14/473 | 117/17653 | 3.00E-06 | 0.000222 | 0.000162 | ZFP36/FCER1G/JAK2/CYBA/CD14/HAVCR2/IRAK3/BCL3/ERRFI1/CYBB/GPNMB/LY96/APP/THBS1 | 14 |
| BP | GO:0061045 | negative regulation of wound healing | 11/473 | 72/17653 | 3.15E-06 | 0.0002292 | 0.0001672 | PRKCD/PROS1/C1QTNF1/PHLDB2/SERPINE1/PLAU/PDGFRA/GJA1/ANXA2/THBS1/APOE | 11 |
| BP | GO:0051348 | negative regulation of transferase activity | 24/473 | 309/17653 | 3.34E-06 | 0.0002394 | 0.0001747 | ZFP36/PRKCD/PIK3IP1/MVP/MIDN/DUSP1/IRAK3/SFRP1/DUSP26/RGS4/ERRFI1/SOCS1/WARS/TRIB1/GADD45A/RGS2/CDKN1A/SOCS2/PTPN1/GADD45B/UCHL1/DUSP5/THY1/APOE | 24 |
| BP | GO:0061687 | detoxification of inorganic compound | 6/473 | 17/17653 | 3.45E-06 | 0.0002396 | 0.0001748 | MT1M/MT1X/MT1A/MT2A/MT1G/MT1E | 6 |
| BP | GO:0097501 | stress response to metal ion | 6/473 | 17/17653 | 3.45E-06 | 0.0002396 | 0.0001748 | MT1M/MT1X/MT1A/MT2A/MT1G/MT1E | 6 |
| BP | GO:1903522 | regulation of blood circulation | 23/473 | 290/17653 | 3.77E-06 | 0.0002579 | 0.0001882 | MYH6/JAK2/SCN2B/CYP2J2/ACE2/PDE5A/ATP1A2/ATP1A1/ATP2A2/KCNA5/ITPR3/PDE4D/KCNJ2/GJA1/RGS2/ATP1B3/RYR2/CASQ1/HBEGF/CORIN/HEY2/TNNI3K/NPPA | 23 |
| BP | GO:0043901 | negative regulation of multi-organism process | 17/473 | 172/17653 | 3.91E-06 | 0.0002633 | 0.0001921 | ZFP36/FCN3/IFITM2/IFITM3/HAVCR2/HTRA1/ISG20/FCN1/IFI16/TRIB1/BST2/IFITM1/ANXA2/PROX1/CTSG/TIMP1/IFIT1 | 17 |
| BP | GO:0018108 | peptidyl-tyrosine phosphorylation | 27/473 | 379/17653 | 4.22E-06 | 0.0002803 | 0.0002045 | PRKCD/SLA/JAK2/FES/DYRK1B/STAT3/HCK/ITGB2/HCLS1/MVP/FGR/SFRP1/ERRFI1/TNFRSF1A/THBS4/PDGFRB/SOCS1/PDGFRA/MAP2K1/ITGA5/HBEGF/IL6/APP/PTPN1/CD44/FGF18/THY1 | 27 |
| BP | GO:0009991 | response to extracellular stimulus | 32/473 | 495/17653 | 4.33E-06 | 0.0002835 | 0.0002069 | ZFP36/PIM1/CYBA/DDIT3/SFRP1/CCND1/PLEKHF1/SLC2A1/GSDMD/CYBB/CHSY1/LDHA/AIF1/PDK4/IFI16/SGIP1/OXCT1/LDLR/SPP1/APOA1/ALPL/FOS/CDKN1A/PROX1/ATF3/LIPG/HAMP/TNC/SLC38A2/PENK/POSTN/APOE | 32 |
| BP | GO:1902903 | regulation of supramolecular fiber organization | 25/473 | 336/17653 | 4.49E-06 | 0.0002895 | 0.0002113 | CDC42EP4/PRKCD/FES/CORO1A/ARPC1B/HCK/WAS/HCLS1/LMOD2/S100A10/PLEK/SFRP1/PHLDB2/ARPC3/SYNPO2L/RAC2/ARPC5L/SNCA/LDLR/APOA1/RGS2/ID1/PROX1/APP/APOE | 25 |
| BP | GO:0042326 | negative regulation of phosphorylation | 31/473 | 473/17653 | 4.65E-06 | 0.0002952 | 0.0002154 | PRKCD/PIK3IP1/MYC/STAT3/MVP/MIDN/DDIT4/DUSP1/IRAK3/SFRP1/DUSP26/RGS4/ERRFI1/KLF4/SOCS1/WARS/SNCA/PDE4D/TRIB1/GADD45A/RGS2/FAM129A/CDKN1A/ATF3/SOCS2/PTPN1/GADD45B/UCHL1/DUSP5/THY1/APOE | 31 |
| BP | GO:0045823 | positive regulation of heart contraction | 8/473 | 37/17653 | 4.88E-06 | 0.0002981 | 0.0002175 | ACE2/ATP1A1/ATP2A2/PDE4D/RGS2/RYR2/HEY2/NPPA | 8 |
| BP | GO:0071276 | cellular response to cadmium ion | 8/473 | 37/17653 | 4.88E-06 | 0.0002981 | 0.0002175 | MT1M/MT1X/MT1A/MT2A/CYBB/MT1G/FOS/MT1E | 8 |
| BP | GO:0018212 | peptidyl-tyrosine modification | 27/473 | 382/17653 | 4.89E-06 | 0.0002981 | 0.0002175 | PRKCD/SLA/JAK2/FES/DYRK1B/STAT3/HCK/ITGB2/HCLS1/MVP/FGR/SFRP1/ERRFI1/TNFRSF1A/THBS4/PDGFRB/SOCS1/PDGFRA/MAP2K1/ITGA5/HBEGF/IL6/APP/PTPN1/CD44/FGF18/THY1 | 27 |
| BP | GO:0071706 | tumor necrosis factor superfamily cytokine production | 14/473 | 123/17653 | 5.44E-06 | 0.0003273 | 0.0002388 | ZFP36/FCER1G/JAK2/CYBA/CD14/HAVCR2/IRAK3/BCL3/ERRFI1/CYBB/GPNMB/LY96/APP/THBS1 | 14 |
| BP | GO:0050867 | positive regulation of cell activation | 26/473 | 363/17653 | 5.77E-06 | 0.0003397 | 0.0002479 | FCER1G/JAK2/CORO1A/ITGB2/BCL6/VAMP8/HAVCR2/CD209/MAP3K8/PLEK/FGR/PRKCQ/AIF1/PDGFRB/SOCS1/CCL2/HLA-DRB4/SOX15/CDKN1A/IL1RL1/IL6/APP/THBS1/TFRC/THY1/NPPA | 26 |
| BP | GO:0007596 | blood coagulation | 25/473 | 341/17653 | 5.82E-06 | 0.0003397 | 0.0002479 | S100A9/PRKCD/FCER1G/JAK2/PROS1/WAS/F13A1/GNA15/PLEK/C1QTNF1/SERPINA5/SERPINE1/PLAU/CD59/ACTB/PRKCQ/RAC2/TSPAN32/CLIC1/ITPR3/PDGFRA/ANXA2/IL6/THBS1/APOE | 25 |
| BP | GO:0055076 | transition metal ion homeostasis | 15/473 | 141/17653 | 5.87E-06 | 0.0003397 | 0.0002479 | S100A9/MT1M/MYC/MT1X/S100A8/MT1A/MT2A/HEPH/MT1G/PICALM/SLC30A2/APP/MT1E/HAMP/TFRC | 15 |
| BP | GO:0006942 | regulation of striated muscle contraction | 12/473 | 92/17653 | 6.20E-06 | 0.0003538 | 0.0002582 | ACE2/PDE5A/ATP1A2/ATP1A1/ATP2A2/PDE4D/KCNJ2/RGS2/RYR2/CASQ1/TNNI3K/NPPA | 12 |
| BP | GO:0051495 | positive regulation of cytoskeleton organization | 19/473 | 217/17653 | 6.43E-06 | 0.0003626 | 0.0002646 | CDC42EP4/FES/CORO1A/ARPC1B/HCK/WAS/HCLS1/LMOD2/S100A10/PLEK/SFRP1/ARPC3/SYNPO2L/RAC2/ARPC5L/APOA1/RGS2/ID1/PROX1 | 19 |
| BP | GO:0007015 | actin filament organization | 27/473 | 388/17653 | 6.51E-06 | 0.0003628 | 0.0002648 | CDC42EP4/PRKCD/JAK2/CORO1A/SORBS2/ARPC1B/HCK/WAS/LCP1/HCLS1/LMOD2/S100A10/SORBS1/PLEK/SFRP1/PHLDB2/ARPC3/ZYX/SYNPO2L/AIF1/RAC2/ARPC5L/CAP1/APOA1/ID1/TPM3/PROX1 | 27 |
| BP | GO:0055117 | regulation of cardiac muscle contraction | 11/473 | 78/17653 | 7.01E-06 | 0.0003813 | 0.0002783 | ACE2/PDE5A/ATP1A2/ATP1A1/ATP2A2/PDE4D/KCNJ2/RGS2/RYR2/TNNI3K/NPPA | 11 |
| BP | GO:0071260 | cellular response to mechanical stimulus | 11/473 | 78/17653 | 7.01E-06 | 0.0003813 | 0.0002783 | CYBA/LTBR/SLC2A1/ATP1A2/TNFRSF1A/ATP1A1/KCNJ2/GADD45A/GJA1/SLC38A2/NPPA | 11 |
| BP | GO:0030195 | negative regulation of blood coagulation | 9/473 | 51/17653 | 7.38E-06 | 0.0003919 | 0.000286 | PRKCD/PROS1/C1QTNF1/SERPINE1/PLAU/PDGFRA/ANXA2/THBS1/APOE | 9 |
| BP | GO:1900047 | negative regulation of hemostasis | 9/473 | 51/17653 | 7.38E-06 | 0.0003919 | 0.000286 | PRKCD/PROS1/C1QTNF1/SERPINE1/PLAU/PDGFRA/ANXA2/THBS1/APOE | 9 |
| BP | GO:0007599 | hemostasis | 25/473 | 346/17653 | 7.50E-06 | 0.0003935 | 0.0002872 | S100A9/PRKCD/FCER1G/JAK2/PROS1/WAS/F13A1/GNA15/PLEK/C1QTNF1/SERPINA5/SERPINE1/PLAU/CD59/ACTB/PRKCQ/RAC2/TSPAN32/CLIC1/ITPR3/PDGFRA/ANXA2/IL6/THBS1/APOE | 25 |
| BP | GO:0050817 | coagulation | 25/473 | 347/17653 | 7.89E-06 | 0.000409 | 0.0002984 | S100A9/PRKCD/FCER1G/JAK2/PROS1/WAS/F13A1/GNA15/PLEK/C1QTNF1/SERPINA5/SERPINE1/PLAU/CD59/ACTB/PRKCQ/RAC2/TSPAN32/CLIC1/ITPR3/PDGFRA/ANXA2/IL6/THBS1/APOE | 25 |
| BP | GO:0006469 | negative regulation of protein kinase activity | 21/473 | 263/17653 | 8.95E-06 | 0.0004591 | 0.000335 | PRKCD/MVP/DUSP1/IRAK3/SFRP1/DUSP26/RGS4/ERRFI1/SOCS1/WARS/TRIB1/GADD45A/RGS2/CDKN1A/SOCS2/PTPN1/GADD45B/UCHL1/DUSP5/THY1/APOE | 21 |
| BP | GO:0046688 | response to copper ion | 8/473 | 40/17653 | 9.06E-06 | 0.0004592 | 0.0003351 | MT1M/MT1X/MT1A/MT2A/S100A13/SNCA/MT1G/MT1E | 8 |
| BP | GO:0006937 | regulation of muscle contraction | 16/473 | 165/17653 | 9.57E-06 | 0.0004799 | 0.0003502 | ACE2/PDE5A/PLCE1/ENO1/ATP1A2/ATP1A1/TNNT1/ATP2A2/PDE4D/KCNJ2/RGS2/RYR2/CASQ1/TNNI3K/CNN1/NPPA | 16 |
| BP | GO:0032956 | regulation of actin cytoskeleton organization | 24/473 | 331/17653 | 1.08E-05 | 0.0005332 | 0.0003891 | CDC42EP4/PRKCD/FES/CORO1A/ARPC1B/HCK/WAS/HCLS1/LMOD2/S100A10/PLEK/SFRP1/PHLDB2/ARPC3/SYNPO2L/RAC2/PDGFRB/ARPC5L/ARHGDIA/PDGFRA/APOA1/ID1/BST2/PROX1 | 24 |
| BP | GO:0032102 | negative regulation of response to external stimulus | 23/473 | 310/17653 | 1.13E-05 | 0.0005432 | 0.0003964 | PRKCD/PROS1/HAVCR2/SEMA4B/C1QTNF1/PHLDB2/TNFRSF1A/HTRA1/RGMA/SERPINE1/PLAU/AIF1/KLF4/CCL2/PDGFRA/TRIB1/SPP1/APOA1/GJA1/ANXA2/IFIT1/THBS1/APOE | 23 |
| BP | GO:0071214 | cellular response to abiotic stimulus | 23/473 | 310/17653 | 1.13E-05 | 0.0005432 | 0.0003964 | MYC/CYBA/CCND2/OSR1/SFRP1/LTBR/ERRFI1/SLC2A1/ATP1A2/TNFRSF1A/ATP1A1/GRM1/IFI16/KCNJ2/GADD45A/GJA1/CDKN1A/MFAP4/HAMP/SLC38A2/GUCA1C/EGR1/NPPA | 23 |
| BP | GO:0104004 | cellular response to environmental stimulus | 23/473 | 310/17653 | 1.13E-05 | 0.0005432 | 0.0003964 | MYC/CYBA/CCND2/OSR1/SFRP1/LTBR/ERRFI1/SLC2A1/ATP1A2/TNFRSF1A/ATP1A1/GRM1/IFI16/KCNJ2/GADD45A/GJA1/CDKN1A/MFAP4/HAMP/SLC38A2/GUCA1C/EGR1/NPPA | 23 |
| BP | GO:0032680 | regulation of tumor necrosis factor production | 13/473 | 114/17653 | 1.15E-05 | 0.0005462 | 0.0003986 | ZFP36/FCER1G/JAK2/CYBA/CD14/HAVCR2/IRAK3/BCL3/ERRFI1/CYBB/GPNMB/LY96/THBS1 | 13 |
| BP | GO:0010038 | response to metal ion | 24/473 | 334/17653 | 1.25E-05 | 0.0005831 | 0.0004255 | MT1M/MT1X/S100A8/MT1A/MT2A/CD14/ALOX5AP/DUSP1/CCND1/CYBB/S100A13/ITPR3/SNCA/AQP9/MT1G/FOS/RYR2/SLC30A2/APP/JUNB/MT1E/HAMP/THBS1/PENK | 24 |
| BP | GO:0048678 | response to axon injury | 10/473 | 68/17653 | 1.25E-05 | 0.0005831 | 0.0004255 | JAK2/RANGAP1/RGMA/AIF1/KLF4/MAP2K1/MATN2/SPP1/APOA1/TNC | 10 |
| BP | GO:0007159 | leukocyte cell-cell adhesion | 24/473 | 335/17653 | 1.31E-05 | 0.0006047 | 0.0004412 | S100A9/S100A8/CORO1A/ITGB2/BCL6/CEBPB/HAVCR2/CD209/MAP3K8/PDE5A/PRKCQ/AIF1/KLF4/RAC2/GPNMB/SOCS1/CCL2/LRRC32/HLA-DRB4/ITGA5/IL6/CD44/TFRC/THY1 | 24 |
| BP | GO:0007160 | cell-matrix adhesion | 18/473 | 209/17653 | 1.41E-05 | 0.0006412 | 0.000468 | LYVE1/ITGB2/BCL6/S100A10/SORBS1/SFRP1/PHLDB2/ZYX/COL16A1/ECM2/SERPINE1/PLAU/EMILIN1/ADAMTS9/CD44/THBS1/THY1/POSTN | 18 |
| BP | GO:0030856 | regulation of epithelial cell differentiation | 15/473 | 152/17653 | 1.47E-05 | 0.0006579 | 0.0004801 | ZFP36/S1PR3/CEBPB/FRZB/OSR1/CCND1/ERRFI1/TNFRSF1A/SERPINE1/APOLD1/PROM1/SFRP4/ID1/CTSK/HEY2 | 15 |
| BP | GO:0016049 | cell growth | 30/473 | 477/17653 | 1.47E-05 | 0.0006579 | 0.0004801 | S100A9/S100A8/SORBS2/BCL6/CYBA/TSPYL2/FRZB/SEMA4B/PLCE1/DCUN1D3/SFRP1/ENO1/HTRA1/FAM107A/PRKCQ/SPOCK1/IGFBP4/SPP1/GJA1/SH3GL2/BST2/CDKN1A/SOCS2/HBEGF/APP/HAMP/CYR61/POSTN/APOE/NPPA | 30 |
| BP | GO:0002573 | myeloid leukocyte differentiation | 17/473 | 191/17653 | 1.58E-05 | 0.0007003 | 0.0005111 | MYC/MAFB/HCLS1/C1QC/CEBPB/SFRP1/LTBR/TYROBP/IFI16/TRIB1/MT1G/FOS/ANXA2/NME1/APP/JUNB/TFRC | 17 |
| BP | GO:0010043 | response to zinc ion | 9/473 | 56/17653 | 1.63E-05 | 0.0007083 | 0.0005169 | MT1M/MT1X/S100A8/MT1A/MT2A/MT1G/SLC30A2/MT1E/HAMP | 9 |
| BP | GO:0050819 | negative regulation of coagulation | 9/473 | 56/17653 | 1.63E-05 | 0.0007083 | 0.0005169 | PRKCD/PROS1/C1QTNF1/SERPINE1/PLAU/PDGFRA/ANXA2/THBS1/APOE | 9 |
| BP | GO:1903555 | regulation of tumor necrosis factor superfamily cytokine production | 13/473 | 118/17653 | 1.68E-05 | 0.0007199 | 0.0005253 | ZFP36/FCER1G/JAK2/CYBA/CD14/HAVCR2/IRAK3/BCL3/ERRFI1/CYBB/GPNMB/LY96/THBS1 | 13 |
| BP | GO:2000377 | regulation of reactive oxygen species metabolic process | 16/473 | 173/17653 | 1.74E-05 | 0.0007406 | 0.0005405 | PRKCD/JAK2/STAT3/ITGB2/CYBA/ACE2/AIF1/KLF4/RAC2/PDGFRB/SNCA/GADD45A/KLF2/CDKN1A/THBS1/CYP1B1 | 16 |
| BP | GO:0010543 | regulation of platelet activation | 7/473 | 32/17653 | 1.79E-05 | 0.0007459 | 0.0005443 | PRKCD/FCER1G/PLEK/C1QTNF1/PRKCQ/PDGFRA/APOE | 7 |
| BP | GO:0090075 | relaxation of muscle | 7/473 | 32/17653 | 1.79E-05 | 0.0007459 | 0.0005443 | PDE5A/ATP1A2/ATP1A1/ATP2A2/PDE4D/KCNJ2/RGS2 | 7 |
| BP | GO:0014896 | muscle hypertrophy | 12/473 | 102/17653 | 1.81E-05 | 0.0007466 | 0.0005448 | MYH6/SORBS2/CYBA/PDE5A/ERRFI1/ATP2A2/LMCD1/RGS2/RYR2/HAMP/HEY2/NPPA | 12 |
| BP | GO:0071294 | cellular response to zinc ion | 6/473 | 22/17653 | 1.86E-05 | 0.0007466 | 0.0005448 | MT1M/MT1X/MT1A/MT2A/MT1G/MT1E | 6 |
| BP | GO:0002521 | leukocyte differentiation | 30/473 | 483/17653 | 1.87E-05 | 0.0007466 | 0.0005448 | FCER1G/MYC/MAFB/CMTM7/STAT3/HCLS1/IL18R1/BCL6/C1QC/DOCK2/LRRC8A/CEBPB/SFRP1/BCL3/LTBR/TYROBP/KLF6/IFI16/SOX4/SOCS1/TRIB1/MT1G/FOS/ANXA2/NME1/IL6/APP/JUNB/TFRC/EGR1 | 30 |
| BP | GO:0001933 | negative regulation of protein phosphorylation | 28/473 | 435/17653 | 1.87E-05 | 0.0007466 | 0.0005448 | PRKCD/MYC/MVP/DDIT4/DUSP1/IRAK3/SFRP1/DUSP26/RGS4/ERRFI1/KLF4/SOCS1/WARS/SNCA/PDE4D/TRIB1/GADD45A/RGS2/FAM129A/CDKN1A/ATF3/SOCS2/PTPN1/GADD45B/UCHL1/DUSP5/THY1/APOE | 28 |
| BP | GO:0071222 | cellular response to lipopolysaccharide | 16/473 | 174/17653 | 1.87E-05 | 0.0007466 | 0.0005448 | ZFP36/HCK/NFKBIA/CEBPB/CD14/HAVCR2/HMGB2/SERPINE1/CCL2/LY96/PDE4D/TRIB1/CTSG/LITAF/IL6/HAMP | 16 |
| BP | GO:0007162 | negative regulation of cell adhesion | 20/473 | 255/17653 | 1.89E-05 | 0.0007499 | 0.0005472 | PRKCD/JAK2/BCL6/CEBPB/MBP/HAVCR2/PDE5A/C1QTNF1/PHLDB2/SERPINE1/KLF4/GPNMB/ARHGDIA/SPOCK1/LRRC32/APOA1/TNC/THBS1/CYP1B1/POSTN | 20 |
| BP | GO:0072593 | reactive oxygen species metabolic process | 20/473 | 256/17653 | 2.01E-05 | 0.0007868 | 0.0005741 | PRKCD/JAK2/STAT3/ITGB2/CYBA/ACE2/DDIT4/CYBB/AIF1/PDK4/KLF4/RAC2/PDGFRB/SNCA/GADD45A/KLF2/CDKN1A/CYR61/THBS1/CYP1B1 | 20 |
| BP | GO:0042110 | T cell activation | 29/473 | 463/17653 | 2.21E-05 | 0.0008579 | 0.0006261 | FCER1G/MAFB/CORO1A/STAT3/WAS/LCP1/IL18R1/BCL6/DOCK2/CEBPB/HAVCR2/CD209/MAP3K8/PDE5A/BCL3/DDOST/PRKCQ/AIF1/RAC2/SOX4/GPNMB/SOCS1/CCL2/LRRC32/HLA-DRB4/IL6/TFRC/THY1/EGR1 | 29 |
| BP | GO:0045785 | positive regulation of cell adhesion | 26/473 | 392/17653 | 2.23E-05 | 0.0008586 | 0.0006266 | JAK2/CORO1A/SMOC2/BCL6/S100A10/HAVCR2/CD209/MAP3K8/SFRP1/DUSP26/FRMD5/COL16A1/VWC2/ECM2/PRKCQ/AIF1/SOCS1/CCL2/HLA-DRB4/APOA1/ITGA5/IL6/CD44/CYR61/TFRC/THY1 | 26 |
| BP | GO:0055001 | muscle cell development | 16/473 | 177/17653 | 2.31E-05 | 0.0008843 | 0.0006453 | MYH6/SORBS2/LMOD2/TNNT1/ATP2A2/PDGFRB/PDGFRA/MYOZ3/SGCG/MYOZ2/CASQ1/PROX1/HAMP/HEY2/UCHL1/NPPA | 16 |
| BP | GO:0031667 | response to nutrient levels | 29/473 | 465/17653 | 2.39E-05 | 0.0009054 | 0.0006607 | ZFP36/PIM1/CYBA/DDIT3/SFRP1/CCND1/PLEKHF1/SLC2A1/CYBB/CHSY1/LDHA/PDK4/IFI16/SGIP1/OXCT1/LDLR/SPP1/APOA1/ALPL/CDKN1A/PROX1/ATF3/LIPG/HAMP/TNC/SLC38A2/PENK/POSTN/APOE | 29 |
| BP | GO:0061448 | connective tissue development | 19/473 | 240/17653 | 2.68E-05 | 0.0010047 | 0.0007332 | DYRK1B/CHRDL2/FRZB/OSR1/SERPINH1/GPR4/CHSY1/LUM/PDGFRB/OXCT1/PRRX1/TIMP1/SULF1/CD44/CYR61/MUSTN1/COMP/FGF18/EGR1 | 19 |
| BP | GO:0035637 | multicellular organismal signaling | 17/473 | 199/17653 | 2.69E-05 | 0.0010047 | 0.0007332 | SCN2B/ACE2/ATP1A2/ATP1A1/ATP2A2/KCNA5/ITPR3/PDE4D/KCNJ2/GJA1/ATP1B3/RYR2/CASQ1/CORIN/TNNI3K/FGF12/NPPA | 17 |
| BP | GO:0001501 | skeletal system development | 30/473 | 493/17653 | 2.75E-05 | 0.0010144 | 0.0007402 | TIPARP/CHRDL2/FRZB/OSR1/SLC39A1/SFRP1/TYROBP/TEAD4/SERPINH1/RDH10/CHSY1/LUM/EXTL1/SOX4/PDGFRA/SFRP4/IGFBP4/NPR3/PRRX1/ALPL/ANXA2/TIMP1/SULF1/CD44/CYR61/MUSTN1/COMP/AEBP1/FGF18/POSTN | 30 |
| BP | GO:0001558 | regulation of cell growth | 26/473 | 397/17653 | 2.77E-05 | 0.0010144 | 0.0007402 | S100A9/S100A8/BCL6/CYBA/TSPYL2/FRZB/SEMA4B/PLCE1/DCUN1D3/SFRP1/ENO1/HTRA1/FAM107A/PRKCQ/SPOCK1/IGFBP4/SPP1/GJA1/BST2/CDKN1A/SOCS2/HBEGF/HAMP/CYR61/APOE/NPPA | 26 |
| BP | GO:0071219 | cellular response to molecule of bacterial origin | 16/473 | 182/17653 | 3.26E-05 | 0.001186 | 0.0008655 | ZFP36/HCK/NFKBIA/CEBPB/CD14/HAVCR2/HMGB2/SERPINE1/CCL2/LY96/PDE4D/TRIB1/CTSG/LITAF/IL6/HAMP | 16 |
| BP | GO:0071902 | positive regulation of protein serine/threonine kinase activity | 24/473 | 355/17653 | 3.38E-05 | 0.0012206 | 0.0008907 | MAP3K6/FPR1/JAK2/FGD2/CCND2/MAP3K8/PDE5A/PLCE1/CCND1/GRM1/MDFIC/TPD52L1/S100A12/PDGFRB/GADD45G/SNCA/MAP2K1/GADD45A/PROX1/PTPN1/GADD45B/THBS1/DUSP5/FGF18 | 24 |
| BP | GO:0071216 | cellular response to biotic stimulus | 17/473 | 204/17653 | 3.70E-05 | 0.0013248 | 0.0009668 | ZFP36/HCK/NFKBIA/CEBPB/CD14/HAVCR2/DDIT3/HMGB2/SERPINE1/CCL2/LY96/PDE4D/TRIB1/CTSG/LITAF/IL6/HAMP | 17 |
| BP | GO:0031100 | animal organ regeneration | 10/473 | 77/17653 | 3.82E-05 | 0.0013554 | 0.0009891 | LCP1/NFKBIA/NNMT/CEBPB/CCND1/TGFBR3/APOA1/CDKN1A/HAMP/PTPN3 | 10 |
| BP | GO:0043406 | positive regulation of MAP kinase activity | 20/473 | 268/17653 | 3.86E-05 | 0.0013598 | 0.0009923 | MAP3K6/FPR1/JAK2/FGD2/MAP3K8/PDE5A/PLCE1/GRM1/MDFIC/TPD52L1/S100A12/PDGFRB/GADD45G/MAP2K1/GADD45A/PTPN1/GADD45B/THBS1/DUSP5/FGF18 | 20 |
| BP | GO:0070252 | actin-mediated cell contraction | 12/473 | 110/17653 | 3.89E-05 | 0.0013598 | 0.0009923 | MYH6/SCN2B/ATP1A2/ATP1A1/TNNT1/KCNA5/PDE4D/KCNJ2/GJA1/TPM3/RYR2/FGF12 | 12 |
| BP | GO:0002576 | platelet degranulation | 13/473 | 128/17653 | 4.00E-05 | 0.0013886 | 0.0010133 | SERPINA3/FCER1G/PROS1/F13A1/SRGN/PLEK/SERPINE1/CFD/LHFPL2/APOA1/TIMP1/APP/THBS1 | 13 |
| BP | GO:0043407 | negative regulation of MAP kinase activity | 10/473 | 78/17653 | 4.27E-05 | 0.0014717 | 0.001074 | PRKCD/DUSP1/IRAK3/SFRP1/RGS4/RGS2/PTPN1/UCHL1/DUSP5/APOE | 10 |
| BP | GO:0086003 | cardiac muscle cell contraction | 9/473 | 63/17653 | 4.32E-05 | 0.0014771 | 0.0010779 | SCN2B/ATP1A2/ATP1A1/KCNA5/PDE4D/KCNJ2/GJA1/RYR2/FGF12 | 9 |
| BP | GO:0043502 | regulation of muscle adaptation | 11/473 | 95/17653 | 4.68E-05 | 0.0015757 | 0.0011499 | PDE5A/ERRFI1/FBXO32/AIF1/TNNT1/KLF4/ATP2A2/LMCD1/RGS2/CASQ1/HAMP | 11 |
| BP | GO:0048525 | negative regulation of viral process | 11/473 | 95/17653 | 4.68E-05 | 0.0015757 | 0.0011499 | ZFP36/FCN3/IFITM2/IFITM3/ISG20/FCN1/IFI16/BST2/IFITM1/PROX1/IFIT1 | 11 |
| BP | GO:1903901 | negative regulation of viral life cycle | 10/473 | 79/17653 | 4.78E-05 | 0.0015966 | 0.0011651 | FCN3/IFITM2/IFITM3/ISG20/FCN1/IFI16/BST2/IFITM1/PROX1/IFIT1 | 10 |
| BP | GO:0007204 | positive regulation of cytosolic calcium ion concentration | 21/473 | 295/17653 | 4.97E-05 | 0.0016471 | 0.001202 | S1PR3/FPR1/JAK2/CORO1A/CYBA/DDIT3/GNA15/PLCE1/C1QTNF1/EDNRA/ATP1A2/GPR4/GRM1/ITPR3/PDGFRA/SNCA/PDE4D/GJA1/RYR2/CASQ1/THY1 | 21 |
| BP | GO:0034113 | heterotypic cell-cell adhesion | 8/473 | 50/17653 | 5.00E-05 | 0.0016471 | 0.001202 | ITGB2/MBP/KLF4/GLDN/APOA1/ITGA5/CD44/THY1 | 8 |
| BP | GO:0015711 | organic anion transport | 28/473 | 460/17653 | 5.05E-05 | 0.0016527 | 0.001206 | PRKCD/SLCO4A1/SLCO2B1/MFSD10/OSR1/ACE2/SLC16A9/PLTP/SLC7A1/SLC2A1/ATP1A2/PLA2G2A/SLC7A5/SNCA/AQP9/CPT1B/SLC16A3/LDLR/APOA1/GJA1/TNFAIP8L3/TMEM30B/CA3/SLC2A3/SLC38A2/THBS1/SLCO2A1/APOE | 28 |
| BP | GO:0045807 | positive regulation of endocytosis | 13/473 | 131/17653 | 5.10E-05 | 0.0016541 | 0.0012071 | FCER1G/CYBA/DOCK2/CD14/SERPINE1/SGIP1/CCL2/SNCA/SFRP4/LDLR/PICALM/ANXA2/APOE | 13 |
| BP | GO:0051047 | positive regulation of secretion | 25/473 | 388/17653 | 5.13E-05 | 0.0016541 | 0.0012071 | S100A9/FCER1G/S100A8/JAK2/ITGB2/CYBA/CD14/MBP/VAMP8/HAVCR2/FGR/C1QTNF1/GSDMD/HGS/FCN1/SOX4/SNCA/OXCT1/SPP1/GJA1/IL1RL1/IL6/RAB15/POSTN/NPPA | 25 |
| BP | GO:0033002 | muscle cell proliferation | 17/473 | 211/17653 | 5.66E-05 | 0.0018116 | 0.001322 | JAK2/STAT3/PIM1/CYBA/AIF1/KLF4/PDGFRB/TGFBR3/NAMPT/NPR3/TRIB1/GJA1/HBEGF/IL6/HEY2/THBS1/CNN1 | 17 |
| BP | GO:0060627 | regulation of vesicle-mediated transport | 29/473 | 490/17653 | 6.15E-05 | 0.001953 | 0.0014252 | FCER1G/FES/CORO1A/HCK/ITGB2/CYBA/DOCK2/CD14/VAMP8/FGR/HGS/SERPINE1/RAC2/SGIP1/ATP2A2/CCL2/SNCA/SFRP4/MAP2K1/LDLR/PICALM/SH3GL2/STXBP6/ANXA2/PTPN1/RABGAP1L/RAB15/APOE/NPPA | 29 |
| BP | GO:0003300 | cardiac muscle hypertrophy | 11/473 | 98/17653 | 6.25E-05 | 0.0019716 | 0.0014388 | MYH6/SORBS2/PDE5A/ERRFI1/ATP2A2/LMCD1/RGS2/RYR2/HAMP/HEY2/NPPA | 11 |
| BP | GO:0030048 | actin filament-based movement | 13/473 | 134/17653 | 6.46E-05 | 0.0020246 | 0.0014775 | MYH6/SCN2B/WAS/ATP1A2/ATP1A1/TNNT1/KCNA5/PDE4D/KCNJ2/GJA1/TPM3/RYR2/FGF12 | 13 |
| BP | GO:0045822 | negative regulation of heart contraction | 6/473 | 27/17653 | 6.57E-05 | 0.0020455 | 0.0014927 | JAK2/PDE5A/ATP1A2/ATP1A1/ATP2A2/PDE4D | 6 |
| BP | GO:0043409 | negative regulation of MAPK cascade | 15/473 | 173/17653 | 6.70E-05 | 0.0020696 | 0.0015103 | PRKCD/MYC/DUSP1/IRAK3/SFRP1/DUSP26/RGS4/ERRFI1/KLF4/RGS2/ATF3/PTPN1/UCHL1/DUSP5/APOE | 15 |
| BP | GO:0051480 | regulation of cytosolic calcium ion concentration | 22/473 | 325/17653 | 7.00E-05 | 0.00215 | 0.001569 | S1PR3/FPR1/JAK2/CORO1A/CYBA/DDIT3/GNA15/PLCE1/C1QTNF1/EDNRA/ATP1A2/GPR4/GRM1/KCNA5/ITPR3/PDGFRA/SNCA/PDE4D/GJA1/RYR2/CASQ1/THY1 | 22 |
| BP | GO:0060537 | muscle tissue development | 25/473 | 396/17653 | 7.13E-05 | 0.0021736 | 0.0015862 | MYH6/TIPARP/SORBS2/PIM1/BTG2/HEG1/OSR1/SVIL/PDGFRB/ADAMTS9/PDGFRA/TGFBR3/HLF/SGCG/GJA1/SOX15/FOS/RYR2/CASQ1/PROX1/ATF3/HAMP/HEY2/EGR1/NPPA | 25 |
| BP | GO:0050730 | regulation of peptidyl-tyrosine phosphorylation | 18/473 | 237/17653 | 7.45E-05 | 0.0022505 | 0.0016423 | PRKCD/JAK2/STAT3/ITGB2/HCLS1/MVP/SFRP1/ERRFI1/TNFRSF1A/THBS4/SOCS1/ITGA5/HBEGF/IL6/APP/PTPN1/CD44/THY1 | 18 |
| BP | GO:0014897 | striated muscle hypertrophy | 11/473 | 100/17653 | 7.53E-05 | 0.0022505 | 0.0016423 | MYH6/SORBS2/PDE5A/ERRFI1/ATP2A2/LMCD1/RGS2/RYR2/HAMP/HEY2/NPPA | 11 |
| BP | GO:0034765 | regulation of ion transmembrane transport | 27/473 | 446/17653 | 7.53E-05 | 0.0022505 | 0.0016423 | SCN2B/CORO1A/CYBA/CAPN1/OSR1/ATP1A2/CYBB/KCNK12/HSPA2/KCNA5/CLIC1/SNCA/CLIC6/PDE4D/KCNJ2/GJA1/ATP1B3/RYR2/CASQ1/APP/HAMP/KLHL24/PTPN3/THBS1/THY1/FGF12/NPPA | 27 |
| BP | GO:0072006 | nephron development | 13/473 | 137/17653 | 8.12E-05 | 0.0024113 | 0.0017596 | MYC/OSR1/IRX2/PLCE1/SEC61A1/GPR4/PDGFRB/PROM1/PDGFRA/KLHL3/IRX3/SULF1/EGR1 | 13 |
| BP | GO:0046718 | viral entry into host cell | 12/473 | 119/17653 | 8.44E-05 | 0.0024896 | 0.0018168 | FCN3/IFITM2/IFITM3/VAMP8/CD209/ACE2/FCN1/PVR/LDLR/ITGA5/IFITM1/TFRC | 12 |
| BP | GO:0030239 | myofibril assembly | 9/473 | 69/17653 | 8.98E-05 | 0.0026306 | 0.0019197 | MYH6/LMOD2/TNNT1/PDGFRB/PDGFRA/MYOZ3/MYOZ2/CASQ1/PROX1 | 9 |
| BP | GO:0030168 | platelet activation | 14/473 | 158/17653 | 9.22E-05 | 0.0026826 | 0.0019577 | PRKCD/FCER1G/GNA15/PLEK/C1QTNF1/ACTB/PRKCQ/RAC2/TSPAN32/CLIC1/ITPR3/PDGFRA/IL6/APOE | 14 |
| BP | GO:0072203 | cell proliferation involved in metanephros development | 4/473 | 10/17653 | 9.40E-05 | 0.0027189 | 0.0019841 | MYC/OSR1/PDGFRB/EGR1 | 4 |
| BP | GO:0001822 | kidney development | 19/473 | 264/17653 | 9.73E-05 | 0.0027479 | 0.0020053 | MYC/TIPARP/OSR1/IRX2/PLCE1/SFRP1/SEC61A1/RDH10/GPR4/SOX4/ID3/PDGFRB/PROM1/PDGFRA/KLHL3/PROX1/IRX3/SULF1/EGR1 | 19 |
| BP | GO:1903524 | positive regulation of blood circulation | 9/473 | 70/17653 | 0.0001006 | 0.0027479 | 0.0020053 | ACE2/ATP1A1/ATP2A2/PDE4D/GJA1/RGS2/RYR2/HEY2/NPPA | 9 |
| BP | GO:0033006 | regulation of mast cell activation involved in immune response | 6/473 | 29/17653 | 0.0001008 | 0.0027479 | 0.0020053 | FCER1G/FES/VAMP8/FGR/RAC2/NPPA | 6 |
| BP | GO:0043304 | regulation of mast cell degranulation | 6/473 | 29/17653 | 0.0001008 | 0.0027479 | 0.0020053 | FCER1G/FES/VAMP8/FGR/RAC2/NPPA | 6 |
| BP | GO:0055075 | potassium ion homeostasis | 6/473 | 29/17653 | 0.0001008 | 0.0027479 | 0.0020053 | SLC12A9/ATP1A2/ATP1A1/KCNA5/KCNJ2/ATP1B3 | 6 |
| BP | GO:0070570 | regulation of neuron projection regeneration | 6/473 | 29/17653 | 0.0001008 | 0.0027479 | 0.0020053 | RGMA/KLF4/MAP2K1/PRRX1/SPP1/THY1 | 6 |
| BP | GO:0032370 | positive regulation of lipid transport | 8/473 | 55/17653 | 0.0001009 | 0.0027479 | 0.0020053 | PRKCD/NFKBIA/PLTP/C1QTNF1/SPP1/ANXA2/LIPG/APOE | 8 |
| BP | GO:0045071 | negative regulation of viral genome replication | 8/473 | 55/17653 | 0.0001009 | 0.0027479 | 0.0020053 | IFITM2/IFITM3/ISG20/IFI16/BST2/IFITM1/PROX1/IFIT1 | 8 |
| BP | GO:0070527 | platelet aggregation | 8/473 | 55/17653 | 0.0001009 | 0.0027479 | 0.0020053 | PRKCD/PLEK/C1QTNF1/ACTB/PRKCQ/TSPAN32/CLIC1/PDGFRA | 8 |
| BP | GO:0071229 | cellular response to acid chemical | 16/473 | 200/17653 | 0.0001011 | 0.0027479 | 0.0020053 | CYBA/CEBPB/OSR1/SFRP1/CYBB/COL16A1/PDK4/KLF4/ID3/SOCS1/LDLR/KLF2/NME1/HAMP/TNC/EGR1 | 16 |
| BP | GO:0002697 | regulation of immune effector process | 25/473 | 405/17653 | 0.0001019 | 0.0027524 | 0.0020086 | C1R/FCER1G/FES/PROS1/WAS/ITGB2/BCL6/C1QC/C1QB/VAMP8/HAVCR2/IRAK3/FGR/HTRA1/CD59/RAC2/TSPAN32/PVR/APOA1/BST2/IL6/IFIT1/TFRC/C6/NPPA | 25 |
| BP | GO:0086001 | cardiac muscle cell action potential | 9/473 | 71/17653 | 0.0001125 | 0.0030226 | 0.0022057 | SCN2B/ATP1A2/ATP1A1/ATP2A2/KCNA5/KCNJ2/GJA1/RYR2/FGF12 | 9 |
| BP | GO:0046597 | negative regulation of viral entry into host cell | 5/473 | 19/17653 | 0.0001152 | 0.0030574 | 0.0022311 | FCN3/IFITM2/IFITM3/FCN1/IFITM1 | 5 |
| BP | GO:0097242 | amyloid-beta clearance | 5/473 | 19/17653 | 0.0001152 | 0.0030574 | 0.0022311 | ITGB2/MARCO/LDLR/PICALM/APOE | 5 |
| BP | GO:0071621 | granulocyte chemotaxis | 11/473 | 105/17653 | 0.0001175 | 0.0031 | 0.0022622 | S100A9/FCER1G/S100A8/ITGB2/THBS4/RAC2/S100A12/CCL2/CXCL2/PDE4D/THBS1 | 11 |
| BP | GO:0055002 | striated muscle cell development | 14/473 | 162/17653 | 0.0001205 | 0.003132 | 0.0022856 | MYH6/SORBS2/LMOD2/TNNT1/PDGFRB/PDGFRA/MYOZ3/MYOZ2/CASQ1/PROX1/HAMP/HEY2/UCHL1/NPPA | 14 |
| BP | GO:0051235 | maintenance of location | 21/473 | 314/17653 | 0.0001207 | 0.003132 | 0.0022856 | S100A9/S100A8/CORO1A/RANGAP1/NFKBIA/CYBA/SRGN/DDIT3/BCL3/ATP1A2/SOX4/ITPR3/SNCA/PDE4D/APOA1/RYR2/CASQ1/SLC30A2/IL6/THY1/APOE | 21 |
| BP | GO:0030593 | neutrophil chemotaxis | 10/473 | 88/17653 | 0.0001208 | 0.003132 | 0.0022856 | S100A9/FCER1G/S100A8/ITGB2/THBS4/RAC2/S100A12/CCL2/CXCL2/PDE4D | 10 |
| BP | GO:0010959 | regulation of metal ion transport | 23/473 | 363/17653 | 0.0001306 | 0.0033493 | 0.0024441 | SCN2B/CORO1A/CYBA/OSR1/ATP1A2/ATP1A1/HSPA2/KCNA5/PDGFRB/CCL2/SNCA/PDE4D/KCNJ2/GJA1/ATP1B3/RYR2/CASQ1/HAMP/KLHL24/PTPN3/THY1/FGF12/NPPA | 23 |
| BP | GO:0045576 | mast cell activation | 8/473 | 57/17653 | 0.0001307 | 0.0033493 | 0.0024441 | FCER1G/FES/VAMP8/FGR/S100A13/RAC2/S100A12/NPPA | 8 |
| BP | GO:0043300 | regulation of leukocyte degranulation | 7/473 | 43/17653 | 0.0001325 | 0.0033493 | 0.0024441 | FCER1G/FES/ITGB2/VAMP8/FGR/RAC2/NPPA | 7 |
| BP | GO:0060337 | type I interferon signaling pathway | 10/473 | 89/17653 | 0.0001329 | 0.0033493 | 0.0024441 | IFITM2/IFITM3/ISG20/IFIT2/BST2/IFITM1/IRF6/PTPN1/IFIT1/EGR1 | 10 |
| BP | GO:0071357 | cellular response to type I interferon | 10/473 | 89/17653 | 0.0001329 | 0.0033493 | 0.0024441 | IFITM2/IFITM3/ISG20/IFIT2/BST2/IFITM1/IRF6/PTPN1/IFIT1/EGR1 | 10 |
| BP | GO:0001649 | osteoblast differentiation | 16/473 | 205/17653 | 0.0001349 | 0.0033555 | 0.0024487 | CEBPB/SFRP1/GPNMB/ID3/CLIC1/FASN/SPP1/GJA1/ID1/ALPL/IFITM1/IL6/JUNB/CYR61/TNC/PENK | 16 |
| BP | GO:0035690 | cellular response to drug | 22/473 | 340/17653 | 0.0001349 | 0.0033555 | 0.0024487 | PRKCD/MYC/MT2A/CYBA/GNA15/DDIT4/SFRP1/ERRFI1/CYBB/FBXO32/ADCY3/ACTB/AIF1/KLF4/ID1/RYR2/KLF2/NME1/IL6/TFRC/CYP1B1/EGR1 | 22 |
| BP | GO:0022407 | regulation of cell-cell adhesion | 24/473 | 388/17653 | 0.0001353 | 0.0033555 | 0.0024487 | PRKCD/JAK2/CORO1A/BCL6/CEBPB/MBP/HAVCR2/CD209/MAP3K8/PDE5A/C1QTNF1/PRKCQ/AIF1/KLF4/GPNMB/SOCS1/CCL2/LRRC32/HLA-DRB4/APOA1/IL6/CD44/TFRC/THY1 | 24 |
| BP | GO:0010811 | positive regulation of cell-substrate adhesion | 11/473 | 107/17653 | 0.0001393 | 0.003434 | 0.002506 | JAK2/SMOC2/S100A10/SFRP1/COL16A1/VWC2/ECM2/APOA1/ITGA5/CYR61/THY1 | 11 |
| BP | GO:0052548 | regulation of endopeptidase activity | 25/473 | 414/17653 | 0.0001437 | 0.0035231 | 0.002571 | SERPINA3/S100A9/MYC/S100A8/JAK2/PROS1/STAT3/MBP/SERPINA5/SERPINH1/SERPINE1/KLF4/IFI16/SPOCK1/TIMP4/SNCA/PICALM/SERPINB1/COL6A3/BST2/TIMP1/APP/CD44/CYR61/THBS1 | 25 |
| BP | GO:0003209 | cardiac atrium morphogenesis | 6/473 | 31/17653 | 0.0001493 | 0.0036054 | 0.0026311 | MYH6/HEG1/SOX4/PROX1/CYR61/HEY2 | 6 |
| BP | GO:0032373 | positive regulation of sterol transport | 5/473 | 20/17653 | 0.0001502 | 0.0036054 | 0.0026311 | NFKBIA/PLTP/ANXA2/LIPG/APOE | 5 |
| BP | GO:0032376 | positive regulation of cholesterol transport | 5/473 | 20/17653 | 0.0001502 | 0.0036054 | 0.0026311 | NFKBIA/PLTP/ANXA2/LIPG/APOE | 5 |
| BP | GO:0035455 | response to interferon-alpha | 5/473 | 20/17653 | 0.0001502 | 0.0036054 | 0.0026311 | IFITM2/IFITM3/IFIT2/BST2/IFITM1 | 5 |
| BP | GO:0043900 | regulation of multi-organism process | 24/473 | 391/17653 | 0.0001521 | 0.0036303 | 0.0026492 | ZFP36/FCN3/IFITM2/IFITM3/CYBA/HAVCR2/PDE5A/HTRA1/MDFIC/ISG20/FCN1/IFI16/TSPAN32/LHFPL2/LY96/TRIB1/BST2/IFITM1/ANXA2/PROX1/CTSG/TIMP1/IFIT1/APOE | 24 |
| BP | GO:0010951 | negative regulation of endopeptidase activity | 18/473 | 251/17653 | 0.0001543 | 0.003652 | 0.002665 | SERPINA3/PROS1/SERPINA5/SERPINH1/SERPINE1/KLF4/IFI16/SPOCK1/TIMP4/SNCA/PICALM/SERPINB1/COL6A3/BST2/TIMP1/APP/CD44/THBS1 | 18 |
| BP | GO:0030100 | regulation of endocytosis | 17/473 | 229/17653 | 0.0001546 | 0.003652 | 0.002665 | FCER1G/HCK/CYBA/DOCK2/CD14/FGR/SERPINE1/SGIP1/CCL2/SNCA/SFRP4/LDLR/PICALM/SH3GL2/ANXA2/PTPN1/APOE | 17 |
| BP | GO:0046890 | regulation of lipid biosynthetic process | 14/473 | 167/17653 | 0.0001663 | 0.0039071 | 0.0028512 | PRKCD/SORBS1/TNFRSF1A/ATP1A1/PDK4/TM7SF2/FASN/LDLR/APOA1/SCD/PROX1/CYR61/APOE/EGR1 | 14 |
| BP | GO:0002703 | regulation of leukocyte mediated immunity | 14/473 | 168/17653 | 0.000177 | 0.0040561 | 0.0029599 | FCER1G/FES/WAS/ITGB2/BCL6/VAMP8/HAVCR2/FGR/RAC2/PVR/BST2/IL6/TFRC/NPPA | 14 |
| BP | GO:0002279 | mast cell activation involved in immune response | 7/473 | 45/17653 | 0.0001782 | 0.0040561 | 0.0029599 | FCER1G/FES/VAMP8/FGR/S100A13/RAC2/NPPA | 7 |
| BP | GO:0002886 | regulation of myeloid leukocyte mediated immunity | 7/473 | 45/17653 | 0.0001782 | 0.0040561 | 0.0029599 | FCER1G/FES/ITGB2/VAMP8/FGR/RAC2/NPPA | 7 |
| BP | GO:0031103 | axon regeneration | 7/473 | 45/17653 | 0.0001782 | 0.0040561 | 0.0029599 | JAK2/RGMA/KLF4/MAP2K1/SPP1/APOA1/TNC | 7 |
| BP | GO:0043303 | mast cell degranulation | 7/473 | 45/17653 | 0.0001782 | 0.0040561 | 0.0029599 | FCER1G/FES/VAMP8/FGR/S100A13/RAC2/NPPA | 7 |
| BP | GO:0110053 | regulation of actin filament organization | 18/473 | 254/17653 | 0.0001789 | 0.0040561 | 0.0029599 | CDC42EP4/PRKCD/CORO1A/ARPC1B/HCK/WAS/HCLS1/LMOD2/S100A10/PLEK/SFRP1/PHLDB2/ARPC3/SYNPO2L/RAC2/ARPC5L/APOA1/ID1 | 18 |
| BP | GO:0006874 | cellular calcium ion homeostasis | 25/473 | 420/17653 | 0.0001794 | 0.0040561 | 0.0029599 | S1PR3/FPR1/JAK2/CORO1A/CYBA/DDIT3/GNA15/PLCE1/C1QTNF1/EDNRA/ATP1A2/GPR4/GRM1/ATP2A2/KCNA5/ITPR3/PDGFRA/SNCA/PDE4D/GJA1/RYR2/CASQ1/APP/THY1/APOE | 25 |
| BP | GO:0001503 | ossification | 23/473 | 371/17653 | 0.0001798 | 0.0040561 | 0.0029599 | CHRDL2/CEBPB/SRGN/OSR1/SFRP1/CHSY1/GPNMB/OMD/ID3/CLIC1/FASN/SPP1/GJA1/ID1/ALPL/CTSK/IFITM1/IL6/JUNB/CYR61/TNC/FGF18/PENK | 23 |
| BP | GO:1901342 | regulation of vasculature development | 24/473 | 396/17653 | 0.000184 | 0.0041311 | 0.0030147 | STAT3/ITGB2/SFRP1/CYBB/GPR4/HGS/PLK2/SERPINE1/KLF4/THBS4/GPNMB/ADAMTS9/WARS/ID1/ITGA5/KLF2/IL6/SULF1/HEY2/THBS1/FGF18/CYP1B1/EGR1/C6 | 24 |
| BP | GO:0034114 | regulation of heterotypic cell-cell adhesion | 5/473 | 21/17653 | 0.0001929 | 0.0042865 | 0.0031281 | MBP/KLF4/APOA1/CD44/THY1 | 5 |
| BP | GO:0043302 | positive regulation of leukocyte degranulation | 5/473 | 21/17653 | 0.0001929 | 0.0042865 | 0.0031281 | FCER1G/ITGB2/VAMP8/FGR/NPPA | 5 |
| BP | GO:0051098 | regulation of binding | 22/473 | 349/17653 | 0.0001956 | 0.0043262 | 0.003157 | PRKCD/JAK2/NFKBIA/S100A10/DDIT3/BCL3/HMGB2/PLK2/ACTB/KLF4/IFI16/ID3/TGFBR3/ID1/IFIT2/ANXA2/NME1/APP/HEY2/IFIT1/XIRP1/APOE | 22 |
| BP | GO:1904062 | regulation of cation transmembrane transport | 20/473 | 302/17653 | 0.0001984 | 0.0043456 | 0.0031712 | SCN2B/CORO1A/CYBA/CAPN1/OSR1/ATP1A2/HSPA2/SNCA/PDE4D/KCNJ2/ATP1B3/RYR2/CASQ1/APP/HAMP/KLHL24/PTPN3/THY1/FGF12/NPPA | 20 |
| BP | GO:0007517 | muscle organ development | 24/473 | 398/17653 | 0.0001984 | 0.0043456 | 0.0031712 | MYH6/PIM1/BTG2/HEG1/SVIL/TEAD4/FZD2/ID3/TGFBR3/HLF/SGCG/GJA1/SOX15/FOS/RYR2/COL6A3/CASQ1/PROX1/ATF3/HBEGF/HAMP/HEY2/AEBP1/EGR1 | 24 |
| BP | GO:0072001 | renal system development | 19/473 | 280/17653 | 0.0002089 | 0.0045339 | 0.0033086 | MYC/TIPARP/OSR1/IRX2/PLCE1/SFRP1/SEC61A1/RDH10/GPR4/SOX4/ID3/PDGFRB/PROM1/PDGFRA/KLHL3/PROX1/IRX3/SULF1/EGR1 | 19 |
| BP | GO:0034340 | response to type I interferon | 10/473 | 94/17653 | 0.0002098 | 0.0045339 | 0.0033086 | IFITM2/IFITM3/ISG20/IFIT2/BST2/IFITM1/IRF6/PTPN1/IFIT1/EGR1 | 10 |
| BP | GO:0002696 | positive regulation of leukocyte activation | 22/473 | 351/17653 | 0.000212 | 0.0045339 | 0.0033086 | FCER1G/CORO1A/ITGB2/BCL6/VAMP8/HAVCR2/CD209/MAP3K8/FGR/PRKCQ/AIF1/SOCS1/CCL2/HLA-DRB4/CDKN1A/IL1RL1/IL6/APP/THBS1/TFRC/THY1/NPPA | 22 |
| BP | GO:0032835 | glomerulus development | 8/473 | 61/17653 | 0.0002121 | 0.0045339 | 0.0033086 | OSR1/PLCE1/GPR4/PDGFRB/PROM1/PDGFRA/SULF1/EGR1 | 8 |
| BP | GO:0070486 | leukocyte aggregation | 4/473 | 12/17653 | 0.0002123 | 0.0045339 | 0.0033086 | S100A9/S100A8/RAC2/CD44 | 4 |
| BP | GO:0071346 | cellular response to interferon-gamma | 14/473 | 171/17653 | 0.000213 | 0.0045339 | 0.0033086 | CDC42EP4/PRKCD/JAK2/HCK/WAS/MT2A/IFI30/ZYX/AIF1/SOCS1/CCL2/HLA-DRB4/IRF6/CD44 | 14 |
| BP | GO:0042035 | regulation of cytokine biosynthetic process | 10/473 | 95/17653 | 0.0002289 | 0.0048154 | 0.003514 | ZFP36/CEBPB/BCL3/ERRFI1/CYBB/PRKCQ/KLF4/IL6/THBS1/EGR1 | 10 |
| BP | GO:0001101 | response to acid chemical | 21/473 | 329/17653 | 0.0002295 | 0.0048154 | 0.003514 | CYBA/CEBPB/OSR1/DUSP1/SFRP1/CYBB/COL16A1/PDK4/KLF4/GLRB/ID3/PDGFRB/SOCS1/TGFBR3/LDLR/GJA1/KLF2/NME1/HAMP/TNC/EGR1 | 21 |
| BP | GO:0006959 | humoral immune response | 21/473 | 329/17653 | 0.0002295 | 0.0048154 | 0.003514 | S100A9/FCN3/C1R/S100A8/PROS1/C1QC/C1QB/MASP1/VSIG4/BCL3/PLA2G2A/CD59/FCN1/CFD/S100A12/CCL2/BST2/CTSG/IL6/LYZ/C6 | 21 |
| BP | GO:0031668 | cellular response to extracellular stimulus | 17/473 | 237/17653 | 0.0002328 | 0.0048623 | 0.0035483 | PIM1/CYBA/SFRP1/PLEKHF1/SLC2A1/GSDMD/CYBB/AIF1/PDK4/IFI16/FOS/CDKN1A/ATF3/TNC/SLC38A2/PENK/POSTN | 17 |
| BP | GO:0002448 | mast cell mediated immunity | 7/473 | 47/17653 | 0.0002359 | 0.0049045 | 0.0035791 | FCER1G/FES/VAMP8/FGR/S100A13/RAC2/NPPA | 7 |
| BP | GO:0006909 | phagocytosis | 21/473 | 330/17653 | 0.0002391 | 0.0049486 | 0.0036112 | FCN3/PRKCD/FCER1G/CORO1A/ARPC1B/HCK/WAS/ITGB2/CYBA/DOCK2/CD14/FCGR2A/FGR/MARCO/ARPC3/ACTB/AIF1/FCN1/RAC2/CCL2/THBS1 | 21 |
| BP | GO:0002888 | positive regulation of myeloid leukocyte mediated immunity | 5/473 | 22/17653 | 0.0002441 | 0.0050194 | 0.0036629 | FCER1G/ITGB2/VAMP8/FGR/NPPA | 5 |
| BP | GO:0014706 | striated muscle tissue development | 23/473 | 379/17653 | 0.0002447 | 0.0050194 | 0.0036629 | MYH6/SORBS2/PIM1/BTG2/HEG1/SVIL/PDGFRB/ADAMTS9/PDGFRA/TGFBR3/HLF/SGCG/GJA1/SOX15/FOS/RYR2/CASQ1/PROX1/ATF3/HAMP/HEY2/EGR1/NPPA | 23 |
| BP | GO:0055074 | calcium ion homeostasis | 25/473 | 431/17653 | 0.000266 | 0.0054299 | 0.0039624 | S1PR3/FPR1/JAK2/CORO1A/CYBA/DDIT3/GNA15/PLCE1/C1QTNF1/EDNRA/ATP1A2/GPR4/GRM1/ATP2A2/KCNA5/ITPR3/PDGFRA/SNCA/PDE4D/GJA1/RYR2/CASQ1/APP/THY1/APOE | 25 |
| BP | GO:0086009 | membrane repolarization | 7/473 | 48/17653 | 0.0002699 | 0.0054847 | 0.0040025 | ATP1A2/ATP1A1/KCNA5/KCNJ2/GJA1/ATP1B3/NPPA | 7 |
| BP | GO:0002027 | regulation of heart rate | 10/473 | 97/17653 | 0.0002717 | 0.0054976 | 0.0040119 | MYH6/SCN2B/ATP2A2/KCNA5/PDE4D/KCNJ2/RYR2/HEY2/TNNI3K/NPPA | 10 |
| BP | GO:0009615 | response to virus | 20/473 | 310/17653 | 0.0002796 | 0.0055406 | 0.0040432 | FCN3/IFITM2/IFITM3/IVNS1ABP/DDIT4/IRAK3/FGR/BCL3/ENO1/HTRA1/RNASE2/ISG20/IFI16/TSPAN32/IFIT2/BST2/IFITM1/IL6/IFIT1/PENK | 20 |
| BP | GO:0030260 | entry into host cell | 12/473 | 135/17653 | 0.00028 | 0.0055406 | 0.0040432 | FCN3/IFITM2/IFITM3/VAMP8/CD209/ACE2/FCN1/PVR/LDLR/ITGA5/IFITM1/TFRC | 12 |
| BP | GO:0044409 | entry into host | 12/473 | 135/17653 | 0.00028 | 0.0055406 | 0.0040432 | FCN3/IFITM2/IFITM3/VAMP8/CD209/ACE2/FCN1/PVR/LDLR/ITGA5/IFITM1/TFRC | 12 |
| BP | GO:0051806 | entry into cell of other organism involved in symbiotic interaction | 12/473 | 135/17653 | 0.00028 | 0.0055406 | 0.0040432 | FCN3/IFITM2/IFITM3/VAMP8/CD209/ACE2/FCN1/PVR/LDLR/ITGA5/IFITM1/TFRC | 12 |
| BP | GO:0051828 | entry into other organism involved in symbiotic interaction | 12/473 | 135/17653 | 0.00028 | 0.0055406 | 0.0040432 | FCN3/IFITM2/IFITM3/VAMP8/CD209/ACE2/FCN1/PVR/LDLR/ITGA5/IFITM1/TFRC | 12 |
| BP | GO:0051101 | regulation of DNA binding | 11/473 | 116/17653 | 0.0002849 | 0.0056135 | 0.0040964 | JAK2/NFKBIA/DDIT3/BCL3/HMGB2/KLF4/IFI16/ID3/ID1/NME1/HEY2 | 11 |
| BP | GO:0010466 | negative regulation of peptidase activity | 18/473 | 264/17653 | 0.0002874 | 0.0056372 | 0.0041138 | SERPINA3/PROS1/SERPINA5/SERPINH1/SERPINE1/KLF4/IFI16/SPOCK1/TIMP4/SNCA/PICALM/SERPINB1/COL6A3/BST2/TIMP1/APP/CD44/THBS1 | 18 |
| BP | GO:0050673 | epithelial cell proliferation | 24/473 | 409/17653 | 0.0002966 | 0.0057479 | 0.0041945 | ZFP36/MYC/STAT3/CYBA/CEBPB/OSR1/SFRP1/CCND1/ERRFI1/HTRA1/HMGB2/THBS4/MAP2K1/TGFBR3/IGFBP4/APOA1/GJA1/ID1/PROX1/NME1/IRF6/SULF1/THBS1/APOE | 24 |
| BP | GO:0046686 | response to cadmium ion | 8/473 | 64/17653 | 0.0002972 | 0.0057479 | 0.0041945 | MT1M/MT1X/MT1A/MT2A/CYBB/MT1G/FOS/MT1E | 8 |
| BP | GO:0033008 | positive regulation of mast cell activation involved in immune response | 4/473 | 13/17653 | 0.0003002 | 0.0057479 | 0.0041945 | FCER1G/VAMP8/FGR/NPPA | 4 |
| BP | GO:0043306 | positive regulation of mast cell degranulation | 4/473 | 13/17653 | 0.0003002 | 0.0057479 | 0.0041945 | FCER1G/VAMP8/FGR/NPPA | 4 |
| BP | GO:0003230 | cardiac atrium development | 6/473 | 35/17653 | 0.0003006 | 0.0057479 | 0.0041945 | MYH6/HEG1/SOX4/PROX1/CYR61/HEY2 | 6 |
| BP | GO:1903523 | negative regulation of blood circulation | 6/473 | 35/17653 | 0.0003006 | 0.0057479 | 0.0041945 | JAK2/PDE5A/ATP1A2/ATP1A1/ATP2A2/PDE4D | 6 |
| BP | GO:0097529 | myeloid leukocyte migration | 14/473 | 177/17653 | 0.0003043 | 0.00576 | 0.0042034 | S100A9/FCER1G/S100A8/ITGB2/SERPINE1/AIF1/THBS4/RAC2/S100A12/CCL2/CXCL2/PDE4D/IL6/THBS1 | 14 |
| BP | GO:0072012 | glomerulus vasculature development | 5/473 | 23/17653 | 0.0003051 | 0.00576 | 0.0042034 | OSR1/GPR4/PDGFRB/PDGFRA/EGR1 | 5 |
| BP | GO:0086064 | cell communication by electrical coupling involved in cardiac conduction | 5/473 | 23/17653 | 0.0003051 | 0.00576 | 0.0042034 | ATP1A2/ATP1A1/PDE4D/GJA1/RYR2 | 5 |
| BP | GO:0032602 | chemokine production | 9/473 | 81/17653 | 0.0003114 | 0.0058545 | 0.0042723 | S100A9/S100A8/MBP/HAVCR2/KLF4/IL1RL1/IL6/POSTN/EGR1 | 9 |
| BP | GO:1903532 | positive regulation of secretion by cell | 22/473 | 361/17653 | 0.0003133 | 0.0058657 | 0.0042805 | FCER1G/JAK2/ITGB2/CD14/MBP/VAMP8/HAVCR2/FGR/C1QTNF1/GSDMD/HGS/FCN1/SOX4/SNCA/OXCT1/SPP1/GJA1/IL1RL1/IL6/RAB15/POSTN/NPPA | 22 |
| BP | GO:1990266 | neutrophil migration | 10/473 | 99/17653 | 0.000321 | 0.0059845 | 0.0043672 | S100A9/FCER1G/S100A8/ITGB2/THBS4/RAC2/S100A12/CCL2/CXCL2/PDE4D | 10 |
| BP | GO:0045637 | regulation of myeloid cell differentiation | 17/473 | 244/17653 | 0.0003274 | 0.006079 | 0.0044362 | ZFP36/MYC/MAFB/FES/STAT3/HCLS1/NFKBIA/C1QC/CEBPB/SFRP1/TYROBP/HMGB2/PRKCQ/TRIB1/FOS/NME1/THBS1 | 17 |
| BP | GO:0042116 | macrophage activation | 8/473 | 65/17653 | 0.0003311 | 0.0061233 | 0.0044685 | HAVCR2/TYROBP/AIF1/SNCA/LDLR/IL1RL1/APP/THBS1 | 8 |
| BP | GO:0032612 | interleukin-1 production | 9/473 | 82/17653 | 0.0003417 | 0.0062666 | 0.004573 | S1PR3/JAK2/HAVCR2/ERRFI1/GSDMD/S100A13/IFI16/APOA1/EGR1 | 9 |
| BP | GO:0055006 | cardiac cell development | 9/473 | 82/17653 | 0.0003417 | 0.0062666 | 0.004573 | MYH6/SORBS2/PDGFRB/PDGFRA/TGFBR3/PROX1/HAMP/HEY2/NPPA | 9 |
| BP | GO:0033280 | response to vitamin D | 6/473 | 36/17653 | 0.0003527 | 0.0064427 | 0.0047016 | PIM1/SFRP1/SPP1/ALPL/TNC/PENK | 6 |
| BP | GO:0097530 | granulocyte migration | 11/473 | 119/17653 | 0.0003557 | 0.0064427 | 0.0047016 | S100A9/FCER1G/S100A8/ITGB2/THBS4/RAC2/S100A12/CCL2/CXCL2/PDE4D/THBS1 | 11 |
| BP | GO:0051271 | negative regulation of cellular component movement | 21/473 | 340/17653 | 0.0003569 | 0.0064427 | 0.0047016 | STAT3/WAS/SEMA4B/SFRP1/PHLDB2/FRMD5/SERPINE1/AIF1/KLF4/CCL2/ADAMTS9/TGFBR3/TRIB1/BST2/IFITM1/TIMP1/SULF1/THBS1/THY1/CYP1B1/APOE | 21 |
| BP | GO:1901652 | response to peptide | 27/473 | 491/17653 | 0.0003573 | 0.0064427 | 0.0047016 | PRKCD/JAK2/STAT3/RANGAP1/BTG2/NFKBIA/CYBA/SORBS1/ERRFI1/SLC2A1/CYBB/ADCY3/PRKCQ/PDK4/KLF4/SOCS1/TIMP4/TGFBR3/GJA1/ID1/KLF2/SOCS2/TIMP1/APP/PTPN1/EGR1/NPPA | 27 |
| BP | GO:0001655 | urogenital system development | 20/473 | 316/17653 | 0.0003584 | 0.0064427 | 0.0047016 | MYC/TIPARP/OSR1/IRX2/PLCE1/SFRP1/SEC61A1/RDH10/GPR4/SOX4/ID3/PDGFRB/PROM1/PDGFRA/KLHL3/PROX1/IRX3/SULF1/TNC/EGR1 | 20 |
| BP | GO:0042742 | defense response to bacterium | 19/473 | 293/17653 | 0.0003701 | 0.0066105 | 0.004824 | S100A9/PRKCD/FCER1G/S100A8/CYBA/CEBPB/HAVCR2/FGR/BCL3/TNFRSF1A/GSDMD/HMGB2/PLA2G2A/SERPINE1/S100A12/CTSG/IL6/HAMP/LYZ | 19 |
| BP | GO:0030595 | leukocyte chemotaxis | 15/473 | 202/17653 | 0.0003707 | 0.0066105 | 0.004824 | S100A9/FCER1G/S100A8/CORO1A/ITGB2/SERPINE1/AIF1/THBS4/RAC2/S100A12/CCL2/CXCL2/PDE4D/IL6/THBS1 | 15 |
| BP | GO:0052547 | regulation of peptidase activity | 25/473 | 441/17653 | 0.0003747 | 0.0066118 | 0.004825 | SERPINA3/S100A9/MYC/S100A8/JAK2/PROS1/STAT3/MBP/SERPINA5/SERPINH1/SERPINE1/KLF4/IFI16/SPOCK1/TIMP4/SNCA/PICALM/SERPINB1/COL6A3/BST2/TIMP1/APP/CD44/CYR61/THBS1 | 25 |
| BP | GO:0010460 | positive regulation of heart rate | 5/473 | 24/17653 | 0.000377 | 0.0066118 | 0.004825 | ATP2A2/PDE4D/RYR2/HEY2/NPPA | 5 |
| BP | GO:0060419 | heart growth | 10/473 | 101/17653 | 0.0003775 | 0.0066118 | 0.004825 | MYH6/SORBS2/PIM1/HEG1/TGFBR3/GJA1/PROX1/HAMP/HEY2/NPPA | 10 |
| BP | GO:0019216 | regulation of lipid metabolic process | 22/473 | 366/17653 | 0.0003783 | 0.0066118 | 0.004825 | SERPINA3/PRKCD/PIK3IP1/SORBS1/FGR/TNFRSF1A/ATP1A1/PDK4/KLF4/TM7SF2/PDGFRB/PDGFRA/SNCA/FASN/LDLR/APOA1/TNFAIP8L3/SCD/PROX1/CYR61/APOE/EGR1 | 22 |
| BP | GO:0045765 | regulation of angiogenesis | 22/473 | 366/17653 | 0.0003783 | 0.0066118 | 0.004825 | STAT3/ITGB2/SFRP1/CYBB/GPR4/HGS/PLK2/SERPINE1/KLF4/THBS4/GPNMB/ADAMTS9/WARS/ID1/ITGA5/KLF2/IL6/SULF1/THBS1/FGF18/CYP1B1/C6 | 22 |
| BP | GO:0051090 | regulation of DNA binding transcription factor activity | 24/473 | 416/17653 | 0.0003795 | 0.0066118 | 0.004825 | S100A9/S100A8/JAK2/PIM1/HCK/ITGB2/NFKBIA/HAVCR2/DDIT3/IRAK3/FZD2/PRKCQ/KLF4/S100A12/ID3/SFRP4/TRIB1/ID1/FOS/PROX1/IL6/APP/BEX1/CYP1B1 | 24 |
| BP | GO:0031960 | response to corticosteroid | 13/473 | 160/17653 | 0.0003822 | 0.0066329 | 0.0048403 | ZFP36/CYBA/DDIT4/DUSP1/CCND1/ERRFI1/CYBB/FBXO32/AIF1/ALPL/FOS/CDKN1A/IL6 | 13 |
| BP | GO:0008154 | actin polymerization or depolymerization | 15/473 | 203/17653 | 0.0003906 | 0.0067532 | 0.0049281 | CDC42EP4/PRKCD/JAK2/CORO1A/ARPC1B/HCK/WAS/HCLS1/LMOD2/PLEK/ARPC3/AIF1/RAC2/ARPC5L/CAP1 | 15 |
| BP | GO:0006869 | lipid transport | 21/473 | 343/17653 | 0.0004009 | 0.0069041 | 0.0050383 | PRKCD/SLCO2B1/NFKBIA/PLTP/C1QTNF1/SERPINA5/PLA2G2A/CEL/APOLD1/AQP9/CPT1B/LDLR/SPP1/APOA1/TNFAIP8L3/TMEM30B/ANXA2/LIPG/THBS1/SLCO2A1/APOE | 21 |
| BP | GO:0032760 | positive regulation of tumor necrosis factor production | 8/473 | 67/17653 | 0.0004084 | 0.0069547 | 0.0050752 | FCER1G/JAK2/CYBA/CD14/HAVCR2/CYBB/LY96/THBS1 | 8 |
| BP | GO:0071479 | cellular response to ionizing radiation | 8/473 | 67/17653 | 0.0004084 | 0.0069547 | 0.0050752 | CYBA/CCND2/SFRP1/IFI16/GADD45A/CDKN1A/HAMP/EGR1 | 8 |
| BP | GO:0030007 | cellular potassium ion homeostasis | 4/473 | 14/17653 | 0.0004114 | 0.0069547 | 0.0050752 | ATP1A2/ATP1A1/KCNJ2/ATP1B3 | 4 |
| BP | GO:0071867 | response to monoamine | 6/473 | 37/17653 | 0.0004115 | 0.0069547 | 0.0050752 | SNCA/PDE4D/ID1/RYR2/APP/PENK | 6 |
| BP | GO:0071869 | response to catecholamine | 6/473 | 37/17653 | 0.0004115 | 0.0069547 | 0.0050752 | SNCA/PDE4D/ID1/RYR2/APP/PENK | 6 |
| BP | GO:0048008 | platelet-derived growth factor receptor signaling pathway | 7/473 | 52/17653 | 0.0004472 | 0.0075027 | 0.0054751 | JAK2/TIPARP/HGS/PDGFRB/PDGFRA/SNCA/PTPN1 | 7 |
| BP | GO:0034103 | regulation of tissue remodeling | 9/473 | 85/17653 | 0.0004473 | 0.0075027 | 0.0054751 | SFRP1/TMBIM1/PDK4/THBS4/GPNMB/SPP1/GJA1/IL6/TFRC | 9 |
| BP | GO:0051216 | cartilage development | 14/473 | 184/17653 | 0.0004513 | 0.007541 | 0.0055031 | CHRDL2/FRZB/OSR1/SERPINH1/CHSY1/LUM/PRRX1/TIMP1/SULF1/CD44/CYR61/MUSTN1/COMP/FGF18 | 14 |
| BP | GO:0007568 | aging | 19/473 | 298/17653 | 0.0004562 | 0.0075955 | 0.0055428 | PRKCD/CTSC/STAT3/ITGB2/BCL6/PLK2/SERPINE1/PDGFRB/SNCA/MAP2K1/PDE4D/PICALM/FOS/CDKN1A/LITAF/TIMP1/APP/HAMP/PENK | 19 |
| BP | GO:0061437 | renal system vasculature development | 5/473 | 25/17653 | 0.0004609 | 0.007618 | 0.0055593 | OSR1/GPR4/PDGFRB/PDGFRA/EGR1 | 5 |
| BP | GO:0061440 | kidney vasculature development | 5/473 | 25/17653 | 0.0004609 | 0.007618 | 0.0055593 | OSR1/GPR4/PDGFRB/PDGFRA/EGR1 | 5 |
| BP | GO:0048660 | regulation of smooth muscle cell proliferation | 12/473 | 143/17653 | 0.0004746 | 0.007815 | 0.005703 | JAK2/CYBA/AIF1/KLF4/PDGFRB/NAMPT/NPR3/TRIB1/HBEGF/IL6/THBS1/CNN1 | 12 |
| BP | GO:1903409 | reactive oxygen species biosynthetic process | 10/473 | 104/17653 | 0.0004776 | 0.0078361 | 0.0057184 | JAK2/STAT3/ITGB2/CYBA/CYBB/AIF1/KLF4/SNCA/KLF2/CYP1B1 | 10 |
| BP | GO:0007584 | response to nutrient | 15/473 | 207/17653 | 0.00048 | 0.0078476 | 0.0057268 | PIM1/CYBA/SFRP1/CCND1/CYBB/LDHA/OXCT1/SPP1/APOA1/ALPL/LIPG/HAMP/TNC/PENK/POSTN | 15 |
| BP | GO:0043392 | negative regulation of DNA binding | 7/473 | 53/17653 | 0.0005036 | 0.0081304 | 0.0059332 | JAK2/NFKBIA/DDIT3/IFI16/ID3/ID1/HEY2 | 7 |
| BP | GO:0046068 | cGMP metabolic process | 7/473 | 53/17653 | 0.0005036 | 0.0081304 | 0.0059332 | PDE5A/FZD2/ADCY3/THBS1/GUCA1C/APOE/NPPA | 7 |
| BP | GO:0086002 | cardiac muscle cell action potential involved in contraction | 7/473 | 53/17653 | 0.0005036 | 0.0081304 | 0.0059332 | SCN2B/ATP1A1/KCNA5/KCNJ2/GJA1/RYR2/FGF12 | 7 |
| BP | GO:0051017 | actin filament bundle assembly | 12/473 | 144/17653 | 0.0005055 | 0.0081304 | 0.0059332 | WAS/LCP1/S100A10/SORBS1/PLEK/SFRP1/PHLDB2/ZYX/SYNPO2L/AIF1/APOA1/ID1 | 12 |
| BP | GO:0045834 | positive regulation of lipid metabolic process | 11/473 | 124/17653 | 0.0005063 | 0.0081304 | 0.0059332 | PRKCD/SORBS1/FGR/TNFRSF1A/PDGFRB/PDGFRA/LDLR/APOA1/TNFAIP8L3/CYR61/APOE | 11 |
| BP | GO:0042089 | cytokine biosynthetic process | 10/473 | 105/17653 | 0.0005155 | 0.0082481 | 0.0060191 | ZFP36/CEBPB/BCL3/ERRFI1/CYBB/PRKCQ/KLF4/IL6/THBS1/EGR1 | 10 |
| BP | GO:1903037 | regulation of leukocyte cell-cell adhesion | 19/473 | 302/17653 | 0.0005371 | 0.0085636 | 0.0062493 | CORO1A/BCL6/CEBPB/HAVCR2/CD209/MAP3K8/PDE5A/PRKCQ/AIF1/KLF4/GPNMB/SOCS1/CCL2/LRRC32/HLA-DRB4/IL6/CD44/TFRC/THY1 | 19 |
| BP | GO:0032409 | regulation of transporter activity | 17/473 | 255/17653 | 0.0005432 | 0.0086301 | 0.0062978 | PRKCD/SCN2B/CAPN1/OSR1/ATP1A2/HSPA2/SNCA/PDE4D/GJA1/ATP1B3/RYR2/CASQ1/APP/KLHL24/PTPN3/FGF12/NPPA | 17 |
| BP | GO:1903557 | positive regulation of tumor necrosis factor superfamily cytokine production | 8/473 | 70/17653 | 0.0005512 | 0.0087087 | 0.0063552 | FCER1G/JAK2/CYBA/CD14/HAVCR2/CYBB/LY96/THBS1 | 8 |
| BP | GO:0033003 | regulation of mast cell activation | 6/473 | 39/17653 | 0.000552 | 0.0087087 | 0.0063552 | FCER1G/FES/VAMP8/FGR/RAC2/NPPA | 6 |
| BP | GO:0001952 | regulation of cell-matrix adhesion | 10/473 | 106/17653 | 0.0005559 | 0.0087087 | 0.0063552 | BCL6/S100A10/SFRP1/PHLDB2/COL16A1/SERPINE1/PLAU/THBS1/THY1/POSTN | 10 |
| BP | GO:0042107 | cytokine metabolic process | 10/473 | 106/17653 | 0.0005559 | 0.0087087 | 0.0063552 | ZFP36/CEBPB/BCL3/ERRFI1/CYBB/PRKCQ/KLF4/IL6/THBS1/EGR1 | 10 |
| BP | GO:0010876 | lipid localization | 22/473 | 377/17653 | 0.0005644 | 0.008797 | 0.0064196 | PRKCD/SLCO2B1/NFKBIA/PLTP/C1QTNF1/SERPINA5/PLA2G2A/CEL/APOLD1/AQP9/CPT1B/LDLR/SPP1/APOA1/TNFAIP8L3/TMEM30B/ANXA2/IL6/LIPG/THBS1/SLCO2A1/APOE | 22 |
| BP | GO:0003229 | ventricular cardiac muscle tissue development | 7/473 | 54/17653 | 0.0005654 | 0.008797 | 0.0064196 | MYH6/HEG1/ADAMTS9/TGFBR3/RYR2/PROX1/HEY2 | 7 |
| BP | GO:0048659 | smooth muscle cell proliferation | 12/473 | 146/17653 | 0.0005723 | 0.0088733 | 0.0064753 | JAK2/CYBA/AIF1/KLF4/PDGFRB/NAMPT/NPR3/TRIB1/HBEGF/IL6/THBS1/CNN1 | 12 |
| BP | GO:0070371 | ERK1 and ERK2 cascade | 20/473 | 328/17653 | 0.0005757 | 0.0088954 | 0.0064914 | MYC/HAVCR2/DUSP1/MARCO/DUSP26/ERRFI1/PLA2G2A/KLF4/GPNMB/PDGFRB/CCL2/PDGFRA/MAP2K1/TNFAIP8L3/ATF3/APP/PTPN1/CD44/CYR61/FGF18 | 20 |
| BP | GO:0001656 | metanephros development | 9/473 | 89/17653 | 0.0006285 | 0.0096462 | 0.0070393 | MYC/OSR1/IRX2/RDH10/ID3/PDGFRB/PDGFRA/IRX3/EGR1 | 9 |
| BP | GO:0060333 | interferon-gamma-mediated signaling pathway | 9/473 | 89/17653 | 0.0006285 | 0.0096462 | 0.0070393 | PRKCD/JAK2/HCK/MT2A/IFI30/SOCS1/HLA-DRB4/IRF6/CD44 | 9 |
| BP | GO:0086065 | cell communication involved in cardiac conduction | 7/473 | 55/17653 | 0.0006332 | 0.0096462 | 0.0070393 | ATP1A2/ATP1A1/KCNA5/PDE4D/GJA1/RYR2/TNNI3K | 7 |
| BP | GO:0043030 | regulation of macrophage activation | 6/473 | 40/17653 | 0.000635 | 0.0096462 | 0.0070393 | HAVCR2/SNCA/LDLR/IL1RL1/APP/THBS1 | 6 |
| BP | GO:0086004 | regulation of cardiac muscle cell contraction | 6/473 | 40/17653 | 0.000635 | 0.0096462 | 0.0070393 | ATP1A2/ATP1A1/ATP2A2/PDE4D/KCNJ2/RYR2 | 6 |
| BP | GO:0061572 | actin filament bundle organization | 12/473 | 148/17653 | 0.0006464 | 0.0097869 | 0.007142 | WAS/LCP1/S100A10/SORBS1/PLEK/SFRP1/PHLDB2/ZYX/SYNPO2L/AIF1/APOA1/ID1 | 12 |
| BP | GO:0097305 | response to alcohol | 14/473 | 191/17653 | 0.0006549 | 0.0098831 | 0.0072122 | S100A8/STAT3/CD14/SFRP1/CCND1/CYBB/KLF4/TGFBR3/OXCT1/KLF2/CA3/HAMP/TNC/PENK | 14 |
| BP | GO:0032652 | regulation of interleukin-1 production | 8/473 | 72/17653 | 0.0006671 | 0.0099793 | 0.0072824 | S1PR3/JAK2/HAVCR2/ERRFI1/GSDMD/IFI16/APOA1/EGR1 | 8 |
| BP | GO:0062012 | regulation of small molecule metabolic process | 26/473 | 485/17653 | 0.0006677 | 0.0099793 | 0.0072824 | STAT3/SORBS1/MIDN/PDE5A/DDIT4/PLEK/C1QTNF1/ENO1/FZD2/PDK4/TM7SF2/SNCA/PDE4D/FASN/CAP1/IGFBP4/RAMP1/NPR3/LDLR/APOA1/SCD/PROX1/THBS1/GUCA1C/APOE/EGR1 | 26 |
| BP | GO:0002026 | regulation of the force of heart contraction | 5/473 | 27/17653 | 0.0006702 | 0.0099793 | 0.0072824 | MYH6/ATP1A2/ATP1A1/ATP2A2/RYR2 | 5 |
| BP | GO:0042730 | fibrinolysis | 5/473 | 27/17653 | 0.0006702 | 0.0099793 | 0.0072824 | PROS1/SERPINE1/PLAU/ANXA2/THBS1 | 5 |
| BP | GO:0033273 | response to vitamin | 9/473 | 90/17653 | 0.0006822 | 0.0101248 | 0.0073886 | PIM1/SFRP1/CCND1/SPP1/ALPL/HAMP/TNC/PENK/POSTN | 9 |
| BP | GO:0042129 | regulation of T cell proliferation | 12/473 | 149/17653 | 0.0006864 | 0.0101536 | 0.0074096 | CORO1A/CEBPB/HAVCR2/CD209/PDE5A/PRKCQ/AIF1/RAC2/GPNMB/LRRC32/IL6/TFRC | 12 |
| BP | GO:0010927 | cellular component assembly involved in morphogenesis | 10/473 | 109/17653 | 0.000693 | 0.0101846 | 0.0074322 | MYH6/LMOD2/PHLDB2/TNNT1/PDGFRB/PDGFRA/MYOZ3/MYOZ2/CASQ1/PROX1 | 10 |
| BP | GO:0032368 | regulation of lipid transport | 10/473 | 109/17653 | 0.000693 | 0.0101846 | 0.0074322 | PRKCD/NFKBIA/PLTP/C1QTNF1/SPP1/APOA1/ANXA2/LIPG/THBS1/APOE | 10 |
| BP | GO:0002526 | acute inflammatory response | 15/473 | 215/17653 | 0.0007122 | 0.0103666 | 0.0075651 | SERPINA3/CD163/C1R/FCER1G/S100A8/PROS1/STAT3/C1QC/C1QB/CEBPB/OSMR/CD59/IL6/HAMP/C6 | 15 |
| BP | GO:0007379 | segment specification | 4/473 | 16/17653 | 0.0007168 | 0.0103666 | 0.0075651 | MAFB/OSR1/IRX2/IRX3 | 4 |
| BP | GO:0045989 | positive regulation of striated muscle contraction | 4/473 | 16/17653 | 0.0007168 | 0.0103666 | 0.0075651 | ACE2/ATP1A1/RGS2/NPPA | 4 |
| BP | GO:0071871 | response to epinephrine | 4/473 | 16/17653 | 0.0007168 | 0.0103666 | 0.0075651 | SNCA/PDE4D/RYR2/PENK | 4 |
| BP | GO:0072224 | metanephric glomerulus development | 4/473 | 16/17653 | 0.0007168 | 0.0103666 | 0.0075651 | OSR1/PDGFRB/PDGFRA/EGR1 | 4 |
| BP | GO:0032273 | positive regulation of protein polymerization | 11/473 | 130/17653 | 0.0007542 | 0.0108716 | 0.0079336 | CDC42EP4/FES/CORO1A/ARPC1B/HCK/WAS/LMOD2/ARPC3/RAC2/ARPC5L/RGS2 | 11 |
| BP | GO:0035265 | organ growth | 13/473 | 172/17653 | 0.0007597 | 0.0108813 | 0.0079406 | MYH6/SORBS2/PIM1/HEG1/PDGFRB/TGFBR3/GJA1/WWC1/PROX1/HAMP/HEY2/COMP/NPPA | 13 |
| BP | GO:0043112 | receptor metabolic process | 13/473 | 172/17653 | 0.0007597 | 0.0108813 | 0.0079406 | FCER1G/ITGB2/CAPN1/ACE2/SNCA/SFRP4/RAMP1/PICALM/SH3GL2/ANXA2/PTPN1/TFRC/APOE | 13 |
| BP | GO:0070372 | regulation of ERK1 and ERK2 cascade | 19/473 | 311/17653 | 0.0007658 | 0.0108998 | 0.0079541 | HAVCR2/DUSP1/MARCO/DUSP26/ERRFI1/PLA2G2A/KLF4/GPNMB/PDGFRB/CCL2/PDGFRA/MAP2K1/TNFAIP8L3/ATF3/APP/PTPN1/CD44/CYR61/FGF18 | 19 |
| BP | GO:2000146 | negative regulation of cell motility | 19/473 | 311/17653 | 0.0007658 | 0.0108998 | 0.0079541 | STAT3/WAS/SFRP1/PHLDB2/FRMD5/SERPINE1/AIF1/KLF4/CCL2/ADAMTS9/TRIB1/BST2/IFITM1/TIMP1/SULF1/THBS1/THY1/CYP1B1/APOE | 19 |
| BP | GO:0010644 | cell communication by electrical coupling | 5/473 | 28/17653 | 0.0007981 | 0.0111323 | 0.0081238 | ATP1A2/ATP1A1/PDE4D/GJA1/RYR2 | 5 |
| BP | GO:0030823 | regulation of cGMP metabolic process | 5/473 | 28/17653 | 0.0007981 | 0.0111323 | 0.0081238 | PDE5A/FZD2/THBS1/GUCA1C/APOE | 5 |
| BP | GO:0034368 | protein-lipid complex remodeling | 5/473 | 28/17653 | 0.0007981 | 0.0111323 | 0.0081238 | PLTP/PLA2G2A/APOA1/LIPG/APOE | 5 |
| BP | GO:0034369 | plasma lipoprotein particle remodeling | 5/473 | 28/17653 | 0.0007981 | 0.0111323 | 0.0081238 | PLTP/PLA2G2A/APOA1/LIPG/APOE | 5 |
| BP | GO:0007589 | body fluid secretion | 9/473 | 92/17653 | 0.0008006 | 0.0111323 | 0.0081238 | CYBA/VAMP8/CCND1/CEL/NPR3/GJA1/ANXA2/NME1/SOCS2 | 9 |
| BP | GO:0034109 | homotypic cell-cell adhesion | 8/473 | 74/17653 | 0.0008019 | 0.0111323 | 0.0081238 | PRKCD/PLEK/C1QTNF1/ACTB/PRKCQ/TSPAN32/CLIC1/PDGFRA | 8 |
| BP | GO:1903725 | regulation of phospholipid metabolic process | 8/473 | 74/17653 | 0.0008019 | 0.0111323 | 0.0081238 | PRKCD/PIK3IP1/FGR/KLF4/PDGFRB/PDGFRA/LDLR/TNFAIP8L3 | 8 |
| BP | GO:0050866 | negative regulation of cell activation | 13/473 | 173/17653 | 0.0008019 | 0.0111323 | 0.0081238 | PRKCD/BCL6/CEBPB/HAVCR2/PDE5A/C1QTNF1/SFRP1/GPNMB/TSPAN32/PDGFRA/LRRC32/LDLR/APOE | 13 |
| BP | GO:0006509 | membrane protein ectodomain proteolysis | 6/473 | 42/17653 | 0.0008295 | 0.0114804 | 0.0083778 | PRKCQ/TIMP4/SPPL2A/TIMP1/PTPN3/APOE | 6 |
| BP | GO:0050663 | cytokine secretion | 14/473 | 196/17653 | 0.0008441 | 0.0116377 | 0.0084926 | CD14/MBP/SRGN/HAVCR2/FGR/GSDMD/S100A13/FCN1/S100A12/SOCS1/LRRC32/APOA1/IL1RL1/POSTN | 14 |
| BP | GO:0042098 | T cell proliferation | 13/473 | 174/17653 | 0.000846 | 0.0116377 | 0.0084926 | CORO1A/DOCK2/CEBPB/HAVCR2/CD209/PDE5A/PRKCQ/AIF1/RAC2/GPNMB/LRRC32/IL6/TFRC | 13 |
| BP | GO:0051209 | release of sequestered calcium ion into cytosol | 10/473 | 112/17653 | 0.0008568 | 0.0117149 | 0.008549 | CORO1A/CYBA/DDIT3/ATP1A2/ITPR3/SNCA/PDE4D/RYR2/CASQ1/THY1 | 10 |
| BP | GO:0051283 | negative regulation of sequestering of calcium ion | 10/473 | 112/17653 | 0.0008568 | 0.0117149 | 0.008549 | CORO1A/CYBA/DDIT3/ATP1A2/ITPR3/SNCA/PDE4D/RYR2/CASQ1/THY1 | 10 |
| BP | GO:0097193 | intrinsic apoptotic signaling pathway | 18/473 | 290/17653 | 0.0008714 | 0.0118471 | 0.0086454 | S100A9/PRKCD/S100A8/JAK2/CEBPB/IVNS1ABP/DDIT3/DDIT4/BCL3/PLEKHF1/ENO1/TNFRSF1A/IFI16/MLLT11/CDKN1A/PTPN1/CD44/CYP1B1 | 18 |
| BP | GO:0010517 | regulation of phospholipase activity | 7/473 | 58/17653 | 0.0008757 | 0.0118471 | 0.0086454 | GNA15/EDNRA/PDGFRB/PDGFRA/SNCA/RGS2/CYR61 | 7 |
| BP | GO:0032651 | regulation of interleukin-1 beta production | 7/473 | 58/17653 | 0.0008757 | 0.0118471 | 0.0086454 | S1PR3/JAK2/ERRFI1/GSDMD/IFI16/APOA1/EGR1 | 7 |
| BP | GO:1905954 | positive regulation of lipid localization | 8/473 | 75/17653 | 0.000877 | 0.0118471 | 0.0086454 | PRKCD/NFKBIA/PLTP/C1QTNF1/SPP1/ANXA2/LIPG/APOE | 8 |
| BP | GO:0010642 | negative regulation of platelet-derived growth factor receptor signaling pathway | 4/473 | 17/17653 | 0.0009177 | 0.0122695 | 0.0089537 | HGS/PDGFRB/PDGFRA/SNCA | 4 |
| BP | GO:0034375 | high-density lipoprotein particle remodeling | 4/473 | 17/17653 | 0.0009177 | 0.0122695 | 0.0089537 | PLTP/APOA1/LIPG/APOE | 4 |
| BP | GO:0051282 | regulation of sequestering of calcium ion | 10/473 | 113/17653 | 0.000918 | 0.0122695 | 0.0089537 | CORO1A/CYBA/DDIT3/ATP1A2/ITPR3/SNCA/PDE4D/RYR2/CASQ1/THY1 | 10 |
| BP | GO:0019058 | viral life cycle | 19/473 | 316/17653 | 0.0009258 | 0.0122695 | 0.0089537 | FCN3/IFITM2/IFITM3/VAMP8/CD209/ACE2/ISG20/FCN1/IFI16/CCL2/PVR/LDLR/ITGA5/BST2/IFITM1/PROX1/IFIT1/TFRC/APOE | 19 |
| BP | GO:0035966 | response to topologically incorrect protein | 14/473 | 198/17653 | 0.0009317 | 0.0122695 | 0.0089537 | TSPYL2/DDIT3/CCND1/SEC61A1/SERPINH1/EXTL1/HSPA2/THBS4/CCL2/HSPA6/ATF3/PTPN1/SDF2L1/THBS1 | 14 |
| BP | GO:0030041 | actin filament polymerization | 13/473 | 176/17653 | 0.0009404 | 0.0122695 | 0.0089537 | CDC42EP4/PRKCD/JAK2/CORO1A/ARPC1B/HCK/WAS/HCLS1/LMOD2/ARPC3/AIF1/RAC2/ARPC5L | 13 |
| BP | GO:2001235 | positive regulation of apoptotic signaling pathway | 13/473 | 176/17653 | 0.0009404 | 0.0122695 | 0.0089537 | S100A9/PRKCD/S100A8/JAK2/CTSC/DDIT3/SFRP1/LTBR/PLEKHF1/TPD52L1/MLLT11/ATF3/THBS1 | 13 |
| BP | GO:0050900 | leukocyte migration | 25/473 | 470/17653 | 0.0009405 | 0.0122695 | 0.0089537 | S100A9/FPR1/FCER1G/S100A8/PROS1/CORO1A/HCK/ITGB2/SERPINE1/AIF1/THBS4/RAC2/S100A12/SLC7A5/CCL2/CXCL2/SLC16A3/PDE4D/ITGA5/ATP1B3/IL6/APP/CD44/THBS1/THY1 | 25 |
| BP | GO:0043277 | apoptotic cell clearance | 6/473 | 43/17653 | 0.0009425 | 0.0122695 | 0.0089537 | FCN3/MARCO/FCN1/RAC2/CCL2/THBS1 | 6 |
| BP | GO:0034367 | protein-containing complex remodeling | 5/473 | 29/17653 | 0.0009433 | 0.0122695 | 0.0089537 | PLTP/PLA2G2A/APOA1/LIPG/APOE | 5 |
| BP | GO:0042036 | negative regulation of cytokine biosynthetic process | 5/473 | 29/17653 | 0.0009433 | 0.0122695 | 0.0089537 | ZFP36/BCL3/ERRFI1/KLF4/IL6 | 5 |
| BP | GO:0071295 | cellular response to vitamin | 5/473 | 29/17653 | 0.0009433 | 0.0122695 | 0.0089537 | PIM1/SFRP1/TNC/PENK/POSTN | 5 |
| BP | GO:0048738 | cardiac muscle tissue development | 15/473 | 221/17653 | 0.0009436 | 0.0122695 | 0.0089537 | MYH6/SORBS2/PIM1/HEG1/PDGFRB/ADAMTS9/PDGFRA/TGFBR3/SGCG/GJA1/RYR2/PROX1/HAMP/HEY2/NPPA | 15 |
| BP | GO:0055013 | cardiac muscle cell development | 8/473 | 76/17653 | 0.0009576 | 0.0124164 | 0.0090609 | MYH6/SORBS2/PDGFRB/PDGFRA/PROX1/HAMP/HEY2/NPPA | 8 |
| BP | GO:1902305 | regulation of sodium ion transmembrane transport | 7/473 | 59/17653 | 0.0009711 | 0.0125542 | 0.0091614 | SCN2B/OSR1/ATP1A2/ATP1B3/KLHL24/PTPN3/FGF12 | 7 |
| BP | GO:0001818 | negative regulation of cytokine production | 16/473 | 245/17653 | 0.0009753 | 0.0125732 | 0.0091753 | ZFP36/BCL6/SRGN/HAVCR2/IRAK3/BCL3/ERRFI1/KLF4/GPNMB/LRRC32/APOA1/KLF2/BST2/IL1RL1/IL6/THBS1 | 16 |
| BP | GO:0002761 | regulation of myeloid leukocyte differentiation | 10/473 | 114/17653 | 0.0009828 | 0.0126331 | 0.009219 | MYC/MAFB/HCLS1/C1QC/CEBPB/SFRP1/TYROBP/TRIB1/FOS/NME1 | 10 |
| BP | GO:0043903 | regulation of symbiosis, encompassing mutualism through parasitism | 15/473 | 222/17653 | 0.0009878 | 0.0126612 | 0.0092395 | ZFP36/FCN3/IFITM2/IFITM3/MDFIC/ISG20/FCN1/IFI16/BST2/IFITM1/ANXA2/PROX1/CTSG/IFIT1/APOE | 15 |
| BP | GO:0008217 | regulation of blood pressure | 13/473 | 177/17653 | 0.0009909 | 0.012665 | 0.0092423 | MYH6/CYBA/ACE2/EDNRA/ATP1A2/ATP1A1/PDE4D/NPR3/GJA1/CTSG/CORIN/POSTN/NPPA | 13 |
| BP | GO:0032386 | regulation of intracellular transport | 22/473 | 395/17653 | 0.0010414 | 0.0132709 | 0.0096845 | PRKCD/FCER1G/JAK2/FES/RANGAP1/LCP1/VAMP8/FGR/RNASE2/MDFIC/RAC2/SOX4/ATP2A2/XPO4/MAP2K1/WWC1/TMEM30B/CTSK/ANXA2/CDKN1A/PTPN1/NPPA | 22 |
| BP | GO:0051279 | regulation of release of sequestered calcium ion into cytosol | 8/473 | 77/17653 | 0.0010441 | 0.0132709 | 0.0096845 | CORO1A/CYBA/ATP1A2/SNCA/PDE4D/RYR2/CASQ1/THY1 | 8 |
| BP | GO:0019079 | viral genome replication | 10/473 | 115/17653 | 0.0010512 | 0.0133231 | 0.0097225 | IFITM2/IFITM3/CD209/ISG20/IFI16/CCL2/BST2/IFITM1/PROX1/IFIT1 | 10 |
| BP | GO:0050670 | regulation of lymphocyte proliferation | 14/473 | 201/17653 | 0.0010774 | 0.0136166 | 0.0099367 | CORO1A/BCL6/CEBPB/HAVCR2/CD209/PDE5A/PRKCQ/AIF1/RAC2/GPNMB/LRRC32/CDKN1A/IL6/TFRC | 14 |
| BP | GO:0006986 | response to unfolded protein | 13/473 | 179/17653 | 0.0010985 | 0.013845 | 0.0101034 | TSPYL2/DDIT3/CCND1/SEC61A1/SERPINH1/EXTL1/HSPA2/THBS4/CCL2/HSPA6/ATF3/PTPN1/THBS1 | 13 |
| BP | GO:0046596 | regulation of viral entry into host cell | 5/473 | 30/17653 | 0.0011073 | 0.0138788 | 0.010128 | FCN3/IFITM2/IFITM3/FCN1/IFITM1 | 5 |
| BP | GO:0051385 | response to mineralocorticoid | 5/473 | 30/17653 | 0.0011073 | 0.0138788 | 0.010128 | CYBA/CCND1/CYBB/FOS/CDKN1A | 5 |
| BP | GO:0051208 | sequestering of calcium ion | 10/473 | 116/17653 | 0.0011235 | 0.0140421 | 0.0102473 | CORO1A/CYBA/DDIT3/ATP1A2/ITPR3/SNCA/PDE4D/RYR2/CASQ1/THY1 | 10 |
| BP | GO:0032944 | regulation of mononuclear cell proliferation | 14/473 | 202/17653 | 0.00113 | 0.0140844 | 0.0102781 | CORO1A/BCL6/CEBPB/HAVCR2/CD209/PDE5A/PRKCQ/AIF1/RAC2/GPNMB/LRRC32/CDKN1A/IL6/TFRC | 14 |
| BP | GO:0051100 | negative regulation of binding | 12/473 | 158/17653 | 0.0011485 | 0.0142011 | 0.0103632 | PRKCD/JAK2/NFKBIA/DDIT3/ACTB/IFI16/ID3/ID1/IFIT2/HEY2/IFIT1/XIRP1 | 12 |
| BP | GO:0010544 | negative regulation of platelet activation | 4/473 | 18/17653 | 0.0011551 | 0.0142011 | 0.0103632 | PRKCD/C1QTNF1/PDGFRA/APOE | 4 |
| BP | GO:0032026 | response to magnesium ion | 4/473 | 18/17653 | 0.0011551 | 0.0142011 | 0.0103632 | CD14/CCND1/SNCA/THBS1 | 4 |
| BP | GO:0033005 | positive regulation of mast cell activation | 4/473 | 18/17653 | 0.0011551 | 0.0142011 | 0.0103632 | FCER1G/VAMP8/FGR/NPPA | 4 |
| BP | GO:0033194 | response to hydroperoxide | 4/473 | 18/17653 | 0.0011551 | 0.0142011 | 0.0103632 | PRKCD/JAK2/MGST1/AIF1 | 4 |
| BP | GO:0031670 | cellular response to nutrient | 7/473 | 61/17653 | 0.0011862 | 0.0145436 | 0.0106132 | PIM1/CYBA/SFRP1/CYBB/TNC/PENK/POSTN | 7 |
| BP | GO:0043551 | regulation of phosphatidylinositol 3-kinase activity | 6/473 | 45/17653 | 0.0012037 | 0.0146394 | 0.0106831 | PIK3IP1/FGR/KLF4/PDGFRB/PDGFRA/TNFAIP8L3 | 6 |
| BP | GO:0046850 | regulation of bone remodeling | 6/473 | 45/17653 | 0.0012037 | 0.0146394 | 0.0106831 | SFRP1/PDK4/SPP1/GJA1/IL6/TFRC | 6 |
| BP | GO:1903115 | regulation of actin filament-based movement | 6/473 | 45/17653 | 0.0012037 | 0.0146394 | 0.0106831 | ATP1A2/ATP1A1/ATP2A2/PDE4D/KCNJ2/RYR2 | 6 |
| BP | GO:0022408 | negative regulation of cell-cell adhesion | 12/473 | 159/17653 | 0.0012129 | 0.0147116 | 0.0107358 | PRKCD/JAK2/BCL6/CEBPB/MBP/HAVCR2/PDE5A/C1QTNF1/KLF4/GPNMB/LRRC32/APOA1 | 12 |
| BP | GO:1905952 | regulation of lipid localization | 11/473 | 138/17653 | 0.0012339 | 0.0149262 | 0.0108924 | PRKCD/NFKBIA/PLTP/C1QTNF1/SPP1/APOA1/ANXA2/IL6/LIPG/THBS1/APOE | 11 |
| BP | GO:0045088 | regulation of innate immune response | 23/473 | 427/17653 | 0.0012599 | 0.0152002 | 0.0110923 | S100A9/PRKCD/FCER1G/S100A8/JAK2/HCK/ITGB2/NFKBIA/CYBA/CD14/HAVCR2/CD209/IRAK3/FGR/MARCO/HMGB2/FCN1/IFI16/SOCS1/LY96/PVR/CTSK/PTPN1 | 23 |
| BP | GO:0015850 | organic hydroxy compound transport | 15/473 | 228/17653 | 0.001291 | 0.0153764 | 0.0112209 | FCER1G/SLCO2B1/NFKBIA/PLTP/C1QTNF1/CEL/SNCA/AQP9/SLC16A3/LDLR/SPP1/APOA1/ANXA2/LIPG/APOE | 15 |
| BP | GO:0044319 | wound healing, spreading of cells | 5/473 | 31/17653 | 0.0012916 | 0.0153764 | 0.0112209 | PHLDB2/ITGA5/HBEGF/CD44/CYR61 | 5 |
| BP | GO:0046627 | negative regulation of insulin receptor signaling pathway | 5/473 | 31/17653 | 0.0012916 | 0.0153764 | 0.0112209 | PRKCD/PRKCQ/SOCS1/SOCS2/PTPN1 | 5 |
| BP | GO:0090505 | epiboly involved in wound healing | 5/473 | 31/17653 | 0.0012916 | 0.0153764 | 0.0112209 | PHLDB2/ITGA5/HBEGF/CD44/CYR61 | 5 |
| BP | GO:1901890 | positive regulation of cell junction assembly | 5/473 | 31/17653 | 0.0012916 | 0.0153764 | 0.0112209 | S100A10/ACE2/SFRP1/COL16A1/THY1 | 5 |
| BP | GO:0051258 | protein polymerization | 17/473 | 276/17653 | 0.0013044 | 0.0154879 | 0.0113023 | CDC42EP4/PRKCD/JAK2/FES/CORO1A/ARPC1B/HCK/WAS/HCLS1/LMOD2/ARPC3/AIF1/RAC2/ARPC5L/SNCA/RGS2/CASQ1 | 17 |
| BP | GO:0002262 | myeloid cell homeostasis | 11/473 | 139/17653 | 0.0013085 | 0.0154956 | 0.0113079 | ZFP36/FCER1G/MAFB/JAK2/STAT3/HCLS1/BCL6/HMGB2/TGFBR3/KLF2/IL6 | 11 |
| BP | GO:0002791 | regulation of peptide secretion | 24/473 | 455/17653 | 0.0013229 | 0.0156249 | 0.0114023 | S100A9/S100A8/JAK2/CD14/MBP/SRGN/HAVCR2/MIDN/FGR/SFRP1/SLC2A1/GSDMD/FCN1/SOX4/KCNA5/SOCS1/ITPR3/LRRC32/OXCT1/APOA1/GJA1/IL1RL1/IL6/POSTN | 24 |
| BP | GO:0010721 | negative regulation of cell development | 18/473 | 301/17653 | 0.0013286 | 0.0156516 | 0.0114217 | S1PR3/STAT3/FRZB/SEMA4B/RGMA/PLK2/TRAK2/ID3/ARHGDIA/SPOCK1/LDLR/SPP1/ID1/APP/IRX3/THY1/POSTN/APOE | 18 |
| BP | GO:0002762 | negative regulation of myeloid leukocyte differentiation | 6/473 | 46/17653 | 0.0013535 | 0.0159033 | 0.0116054 | MYC/MAFB/C1QC/SFRP1/TRIB1/NME1 | 6 |
| BP | GO:0045861 | negative regulation of proteolysis | 20/473 | 352/17653 | 0.0013676 | 0.0160274 | 0.011696 | SERPINA3/PROS1/SERPINA5/SERPINH1/SERPINE1/CD59/KLF4/IFI16/SPOCK1/TIMP4/SNCA/PICALM/SERPINB1/COL6A3/BST2/TIMP1/APP/CD44/PTPN3/THBS1 | 20 |
| BP | GO:0048732 | gland development | 23/473 | 430/17653 | 0.0013807 | 0.0161394 | 0.0117778 | MAFB/JAK2/NFKBIA/CEBPB/FRZB/CAPN1/SERPINA5/SFRP1/CCND1/MAP2K1/TGFBR3/FASN/APOA1/GJA1/ALDH1A3/PROX1/NME1/SOCS2/IRF6/HAMP/SULF1/TNC/PTPN3 | 23 |
| BP | GO:0070661 | leukocyte proliferation | 17/473 | 278/17653 | 0.0014099 | 0.0164035 | 0.0119705 | PRKCD/CORO1A/BCL6/DOCK2/CEBPB/HAVCR2/CD209/PDE5A/PRKCQ/AIF1/RAC2/GPNMB/LRRC32/NPR3/CDKN1A/IL6/TFRC | 17 |
| BP | GO:0040013 | negative regulation of locomotion | 20/473 | 353/17653 | 0.0014147 | 0.0164035 | 0.0119705 | STAT3/WAS/SEMA4B/SFRP1/PHLDB2/FRMD5/SERPINE1/AIF1/KLF4/CCL2/ADAMTS9/TRIB1/BST2/IFITM1/TIMP1/SULF1/THBS1/THY1/CYP1B1/APOE | 20 |
| BP | GO:0030509 | BMP signaling pathway | 12/473 | 162/17653 | 0.0014243 | 0.0164035 | 0.0119705 | MYH6/SFRP1/HTRA1/VWC2/RGMA/SFRP4/TGFBR3/ID1/RYR2/SULF1/CYR61/EGR1 | 12 |
| BP | GO:1901568 | fatty acid derivative metabolic process | 12/473 | 162/17653 | 0.0014243 | 0.0164035 | 0.0119705 | ALOX5/MGST1/ALOX5AP/CYP2J2/TBXAS1/TNFRSF1A/FASN/OXCT1/TYRP1/PLA2G4C/SCD/CYP1B1 | 12 |
| BP | GO:0035994 | response to muscle stretch | 4/473 | 19/17653 | 0.0014324 | 0.0164035 | 0.0119705 | NFKBIA/FOS/RYR2/NPPA | 4 |
| BP | GO:0042533 | tumor necrosis factor biosynthetic process | 4/473 | 19/17653 | 0.0014324 | 0.0164035 | 0.0119705 | BCL3/ERRFI1/CYBB/THBS1 | 4 |
| BP | GO:0042534 | regulation of tumor necrosis factor biosynthetic process | 4/473 | 19/17653 | 0.0014324 | 0.0164035 | 0.0119705 | BCL3/ERRFI1/CYBB/THBS1 | 4 |
| BP | GO:0072111 | cell proliferation involved in kidney development | 4/473 | 19/17653 | 0.0014324 | 0.0164035 | 0.0119705 | MYC/OSR1/PDGFRB/EGR1 | 4 |
| BP | GO:0030838 | positive regulation of actin filament polymerization | 9/473 | 100/17653 | 0.0014532 | 0.0165734 | 0.0120944 | CDC42EP4/CORO1A/ARPC1B/HCK/WAS/LMOD2/ARPC3/RAC2/ARPC5L | 9 |
| BP | GO:1901888 | regulation of cell junction assembly | 8/473 | 81/17653 | 0.0014546 | 0.0165734 | 0.0120944 | S100A10/ACE2/SFRP1/PHLDB2/COL16A1/GJA1/THBS1/THY1 | 8 |
| BP | GO:0030278 | regulation of ossification | 13/473 | 185/17653 | 0.0014818 | 0.0168405 | 0.0122893 | CEBPB/SRGN/OSR1/SFRP1/CHSY1/OMD/ID3/CLIC1/GJA1/ID1/IFITM1/IL6/CYR61 | 13 |
| BP | GO:0031669 | cellular response to nutrient levels | 14/473 | 208/17653 | 0.0014931 | 0.0168918 | 0.0123268 | PIM1/CYBA/SFRP1/PLEKHF1/SLC2A1/CYBB/PDK4/IFI16/CDKN1A/ATF3/TNC/SLC38A2/PENK/POSTN | 14 |
| BP | GO:0090504 | epiboly | 5/473 | 32/17653 | 0.0014975 | 0.0168918 | 0.0123268 | PHLDB2/ITGA5/HBEGF/CD44/CYR61 | 5 |
| BP | GO:1904037 | positive regulation of epithelial cell apoptotic process | 5/473 | 32/17653 | 0.0014975 | 0.0168918 | 0.0123268 | JAK2/CD248/SFRP4/IL6/THBS1 | 5 |
| BP | GO:0050732 | negative regulation of peptidyl-tyrosine phosphorylation | 6/473 | 47/17653 | 0.001517 | 0.0170694 | 0.0124564 | PRKCD/MVP/SFRP1/ERRFI1/SOCS1/THY1 | 6 |
| BP | GO:0006898 | receptor-mediated endocytosis | 19/473 | 330/17653 | 0.0015344 | 0.0172224 | 0.012568 | CD163/FCER1G/ITGB2/CD14/MASP1/MARCO/SERPINE1/SGIP1/SNCA/SFRP4/CAP1/RAMP1/LDLR/APOA1/PICALM/SH3GL2/ANXA2/TFRC/APOE | 19 |
| BP | GO:0071901 | negative regulation of protein serine/threonine kinase activity | 11/473 | 142/17653 | 0.0015546 | 0.0173618 | 0.0126698 | PRKCD/DUSP1/IRAK3/SFRP1/RGS4/RGS2/CDKN1A/PTPN1/UCHL1/DUSP5/APOE | 11 |
| BP | GO:0014066 | regulation of phosphatidylinositol 3-kinase signaling | 9/473 | 101/17653 | 0.0015584 | 0.0173618 | 0.0126698 | PIK3IP1/JAK2/HCLS1/FGR/HCST/KLF4/IER3/PDGFRB/PDGFRA | 9 |
| BP | GO:0031532 | actin cytoskeleton reorganization | 9/473 | 101/17653 | 0.0015584 | 0.0173618 | 0.0126698 | S100A9/FES/HCK/HCLS1/PLEK/RAC2/ARHGDIA/PDGFRA/PTPN1 | 9 |
| BP | GO:0046849 | bone remodeling | 8/473 | 82/17653 | 0.0015749 | 0.0174816 | 0.0127572 | SFRP1/PDK4/RAC2/SPP1/GJA1/CTSK/IL6/TFRC | 8 |
| BP | GO:0046889 | positive regulation of lipid biosynthetic process | 7/473 | 64/17653 | 0.0015769 | 0.0174816 | 0.0127572 | PRKCD/SORBS1/TNFRSF1A/LDLR/APOA1/CYR61/APOE | 7 |
| BP | GO:0051384 | response to glucocorticoid | 11/473 | 143/17653 | 0.0016446 | 0.0181874 | 0.0132723 | ZFP36/DDIT4/DUSP1/CCND1/ERRFI1/FBXO32/AIF1/ALPL/FOS/CDKN1A/IL6 | 11 |
| BP | GO:0006979 | response to oxidative stress | 23/473 | 436/17653 | 0.0016524 | 0.0182285 | 0.0133022 | PRKCD/JAK2/MGST1/CYBA/DUSP1/CYBB/LDHA/AIF1/KLF4/ATP2A2/KCNA5/PDGFRB/PDGFRA/SNCA/FOS/KLF2/CA3/ACOX2/IL6/APP/PENK/CYP1B1/APOE | 23 |
| BP | GO:0006953 | acute-phase response | 6/473 | 48/17653 | 0.0016952 | 0.0185648 | 0.0135477 | SERPINA3/CD163/STAT3/CEBPB/IL6/HAMP | 6 |
| BP | GO:0055010 | ventricular cardiac muscle tissue morphogenesis | 6/473 | 48/17653 | 0.0016952 | 0.0185648 | 0.0135477 | MYH6/HEG1/TGFBR3/RYR2/PROX1/HEY2 | 6 |
| BP | GO:1903307 | positive regulation of regulated secretory pathway | 6/473 | 48/17653 | 0.0016952 | 0.0185648 | 0.0135477 | FCER1G/ITGB2/VAMP8/FGR/RAB15/NPPA | 6 |
| BP | GO:0032271 | regulation of protein polymerization | 14/473 | 211/17653 | 0.0017084 | 0.0186639 | 0.01362 | CDC42EP4/PRKCD/FES/CORO1A/ARPC1B/HCK/WAS/HCLS1/LMOD2/ARPC3/RAC2/ARPC5L/SNCA/RGS2 | 14 |
| BP | GO:0090066 | regulation of anatomical structure size | 25/473 | 491/17653 | 0.0017197 | 0.0186819 | 0.0136331 | CDC42EP4/PRKCD/CORO1A/ARPC1B/HCK/WAS/HCLS1/LMOD2/LRRC8A/SLC12A9/ACE2/SEMA4B/PLEK/EDNRA/ATP1A2/ARPC3/RAC2/KCNA5/ARPC5L/SPP1/GJA1/RGS2/PICALM/APOE/NPPA | 25 |
| BP | GO:0003299 | muscle hypertrophy in response to stress | 5/473 | 33/17653 | 0.0017266 | 0.0186819 | 0.0136331 | MYH6/ERRFI1/ATP2A2/HEY2/NPPA | 5 |
| BP | GO:0014887 | cardiac muscle adaptation | 5/473 | 33/17653 | 0.0017266 | 0.0186819 | 0.0136331 | MYH6/ERRFI1/ATP2A2/HEY2/NPPA | 5 |
| BP | GO:0014898 | cardiac muscle hypertrophy in response to stress | 5/473 | 33/17653 | 0.0017266 | 0.0186819 | 0.0136331 | MYH6/ERRFI1/ATP2A2/HEY2/NPPA | 5 |
| BP | GO:0003158 | endothelium development | 10/473 | 123/17653 | 0.0017514 | 0.0188766 | 0.0137752 | S1PR3/HEG1/TNFRSF1A/APOLD1/SOX18/PDE4D/GJA1/ID1/PROX1/HEY2 | 10 |
| BP | GO:0071305 | cellular response to vitamin D | 4/473 | 20/17653 | 0.0017529 | 0.0188766 | 0.0137752 | PIM1/SFRP1/TNC/PENK | 4 |
| BP | GO:0070663 | regulation of leukocyte proliferation | 14/473 | 212/17653 | 0.0017856 | 0.0191541 | 0.0139777 | CORO1A/BCL6/CEBPB/HAVCR2/CD209/PDE5A/PRKCQ/AIF1/RAC2/GPNMB/LRRC32/CDKN1A/IL6/TFRC | 14 |
| BP | GO:0033559 | unsaturated fatty acid metabolic process | 9/473 | 103/17653 | 0.0017872 | 0.0191541 | 0.0139777 | ALOX5/MGST1/ALOX5AP/CYP2J2/TBXAS1/TNFRSF1A/PLA2G4C/SCD/CYP1B1 | 9 |
| BP | GO:0046651 | lymphocyte proliferation | 16/473 | 260/17653 | 0.0018061 | 0.0193103 | 0.0140917 | PRKCD/CORO1A/BCL6/DOCK2/CEBPB/HAVCR2/CD209/PDE5A/PRKCQ/AIF1/RAC2/GPNMB/LRRC32/CDKN1A/IL6/TFRC | 16 |
| BP | GO:0060191 | regulation of lipase activity | 8/473 | 84/17653 | 0.0018391 | 0.019571 | 0.0142819 | GNA15/EDNRA/PDGFRB/PDGFRA/SNCA/APOA1/RGS2/CYR61 | 8 |
| BP | GO:1903426 | regulation of reactive oxygen species biosynthetic process | 8/473 | 84/17653 | 0.0018391 | 0.019571 | 0.0142819 | JAK2/STAT3/ITGB2/CYBA/AIF1/KLF4/SNCA/KLF2 | 8 |
| BP | GO:0031345 | negative regulation of cell projection organization | 12/473 | 167/17653 | 0.0018437 | 0.0195737 | 0.0142839 | PRKCD/SEMA4B/RGMA/PLK2/TRAK2/ARHGDIA/SPOCK1/SPP1/ID1/MAP4/THY1/APOE | 12 |
| BP | GO:0046330 | positive regulation of JNK cascade | 10/473 | 124/17653 | 0.0018606 | 0.0197063 | 0.0143807 | MAP3K6/FGD2/LTBR/MDFIC/TPD52L1/GADD45G/GADD45A/APP/PTPN1/GADD45B | 10 |
| BP | GO:0015914 | phospholipid transport | 7/473 | 66/17653 | 0.0018885 | 0.0199118 | 0.0145306 | PRKCD/PLTP/LDLR/APOA1/TNFAIP8L3/TMEM30B/APOE | 7 |
| BP | GO:2000649 | regulation of sodium ion transmembrane transporter activity | 6/473 | 49/17653 | 0.0018888 | 0.0199118 | 0.0145306 | SCN2B/OSR1/ATP1A2/KLHL24/PTPN3/FGF12 | 6 |
| BP | GO:0030799 | regulation of cyclic nucleotide metabolic process | 9/473 | 104/17653 | 0.0019113 | 0.0201018 | 0.0146693 | PDE5A/FZD2/PDE4D/CAP1/RAMP1/NPR3/THBS1/GUCA1C/APOE | 9 |
| BP | GO:0032943 | mononuclear cell proliferation | 16/473 | 262/17653 | 0.0019521 | 0.0204389 | 0.0149152 | PRKCD/CORO1A/BCL6/DOCK2/CEBPB/HAVCR2/CD209/PDE5A/PRKCQ/AIF1/RAC2/GPNMB/LRRC32/CDKN1A/IL6/TFRC | 16 |
| BP | GO:0071868 | cellular response to monoamine stimulus | 5/473 | 34/17653 | 0.0019803 | 0.0204389 | 0.0149152 | SNCA/PDE4D/ID1/RYR2/APP | 5 |
| BP | GO:0071870 | cellular response to catecholamine stimulus | 5/473 | 34/17653 | 0.0019803 | 0.0204389 | 0.0149152 | SNCA/PDE4D/ID1/RYR2/APP | 5 |
| BP | GO:0097421 | liver regeneration | 5/473 | 34/17653 | 0.0019803 | 0.0204389 | 0.0149152 | NFKBIA/CEBPB/CCND1/HAMP/PTPN3 | 5 |
| BP | GO:1900077 | negative regulation of cellular response to insulin stimulus | 5/473 | 34/17653 | 0.0019803 | 0.0204389 | 0.0149152 | PRKCD/PRKCQ/SOCS1/SOCS2/PTPN1 | 5 |
| BP | GO:0002028 | regulation of sodium ion transport | 8/473 | 85/17653 | 0.0019837 | 0.0204389 | 0.0149152 | SCN2B/OSR1/ATP1A2/ATP1A1/ATP1B3/KLHL24/PTPN3/FGF12 | 8 |
| BP | GO:0030301 | cholesterol transport | 8/473 | 85/17653 | 0.0019837 | 0.0204389 | 0.0149152 | NFKBIA/PLTP/CEL/LDLR/APOA1/ANXA2/LIPG/APOE | 8 |
| BP | GO:0034115 | negative regulation of heterotypic cell-cell adhesion | 3/473 | 10/17653 | 0.0019932 | 0.0204389 | 0.0149152 | MBP/KLF4/APOA1 | 3 |
| BP | GO:0070587 | regulation of cell-cell adhesion involved in gastrulation | 3/473 | 10/17653 | 0.0019932 | 0.0204389 | 0.0149152 | MBP/KLF4/APOA1 | 3 |
| BP | GO:1900221 | regulation of amyloid-beta clearance | 3/473 | 10/17653 | 0.0019932 | 0.0204389 | 0.0149152 | LDLR/PICALM/APOE | 3 |
| BP | GO:2000341 | regulation of chemokine (C-X-C motif) ligand 2 production | 3/473 | 10/17653 | 0.0019932 | 0.0204389 | 0.0149152 | MBP/KLF4/POSTN | 3 |
| BP | GO:1901615 | organic hydroxy compound metabolic process | 25/473 | 497/17653 | 0.0020252 | 0.0207206 | 0.0151208 | GALK1/INPP1/PLCE1/PLEK/PLTP/CRYM/RDH10/DIO2/LDHA/CEL/TM7SF2/SNCA/RDH5/FASN/TYRP1/LDLR/APOA1/ALDH1A3/SCD/PROX1/ACOX2/APP/CYP1B1/APOE/ADH1A | 25 |
| BP | GO:0043433 | negative regulation of DNA binding transcription factor activity | 12/473 | 169/17653 | 0.0020376 | 0.0207524 | 0.0151441 | PIM1/NFKBIA/HAVCR2/DDIT3/IRAK3/KLF4/ID3/SFRP4/TRIB1/ID1/PROX1/CYP1B1 | 12 |
| BP | GO:0048771 | tissue remodeling | 12/473 | 169/17653 | 0.0020376 | 0.0207524 | 0.0151441 | CAPN1/SFRP1/TMBIM1/PDK4/THBS4/RAC2/GPNMB/SPP1/GJA1/CTSK/IL6/TFRC | 12 |
| BP | GO:0045446 | endothelial cell differentiation | 9/473 | 105/17653 | 0.0020421 | 0.0207524 | 0.0151441 | S1PR3/HEG1/TNFRSF1A/APOLD1/SOX18/PDE4D/ID1/PROX1/HEY2 | 9 |
| BP | GO:0009187 | cyclic nucleotide metabolic process | 11/473 | 147/17653 | 0.0020482 | 0.0207592 | 0.015149 | PDE5A/FZD2/ADCY3/PDE4D/CAP1/RAMP1/NPR3/THBS1/GUCA1C/APOE/NPPA | 11 |
| BP | GO:0003281 | ventricular septum development | 7/473 | 67/17653 | 0.0020612 | 0.0207592 | 0.015149 | HEG1/FZD2/SOX4/TGFBR3/PROX1/CYR61/HEY2 | 7 |
| BP | GO:0032418 | lysosome localization | 7/473 | 67/17653 | 0.0020612 | 0.0207592 | 0.015149 | FCER1G/FES/VAMP8/FGR/S100A13/RAC2/NPPA | 7 |
| BP | GO:0032611 | interleukin-1 beta production | 7/473 | 67/17653 | 0.0020612 | 0.0207592 | 0.015149 | S1PR3/JAK2/ERRFI1/GSDMD/IFI16/APOA1/EGR1 | 7 |
| BP | GO:0048545 | response to steroid hormone | 21/473 | 391/17653 | 0.0020889 | 0.0209909 | 0.0153181 | ZFP36/JAK2/CYBA/DDIT4/DUSP1/SFRP1/CCND1/ERRFI1/ATP1A2/ATP1A1/CYBB/HMGB2/FBXO32/AIF1/GABRB1/SPP1/ALPL/FOS/CDKN1A/IL6/THBS1 | 21 |
| BP | GO:0002460 | adaptive immune response based on somatic recombination of immune receptors built from immunoglobulin superfamily domains | 18/473 | 314/17653 | 0.0021174 | 0.0211814 | 0.0154571 | PRKCD/C1R/FCER1G/CTSC/STAT3/WAS/IL18R1/BCL6/C1QC/C1QB/HAVCR2/BCL3/PRKCQ/PVR/IL1RL1/IL6/TFRC/C6 | 18 |
| BP | GO:0036499 | PERK-mediated unfolded protein response | 4/473 | 21/17653 | 0.00212 | 0.0211814 | 0.0154571 | DDIT3/CCL2/ATF3/PTPN1 | 4 |
| BP | GO:0007611 | learning or memory | 15/473 | 240/17653 | 0.0021354 | 0.0211814 | 0.0154571 | BTG2/CEBPB/CCND2/ATP1A2/GATM/PLK2/ADCY3/ITPR3/NPTX2/LDLR/ITGA5/PICALM/FOS/APP/APOE | 15 |
| BP | GO:0022898 | regulation of transmembrane transporter activity | 15/473 | 240/17653 | 0.0021354 | 0.0211814 | 0.0154571 | SCN2B/CAPN1/OSR1/ATP1A2/HSPA2/PDE4D/GJA1/ATP1B3/RYR2/CASQ1/APP/KLHL24/PTPN3/FGF12/NPPA | 15 |
| BP | GO:0048661 | positive regulation of smooth muscle cell proliferation | 8/473 | 86/17653 | 0.0021371 | 0.0211814 | 0.0154571 | JAK2/CYBA/AIF1/PDGFRB/NAMPT/HBEGF/IL6/THBS1 | 8 |
| BP | GO:1901655 | cellular response to ketone | 8/473 | 86/17653 | 0.0021371 | 0.0211814 | 0.0154571 | DDIT4/SFRP1/ERRFI1/FBXO32/ADCY3/KLF4/SPP1/KLF2 | 8 |
| BP | GO:0030308 | negative regulation of cell growth | 12/473 | 170/17653 | 0.0021407 | 0.0211814 | 0.0154571 | BCL6/TSPYL2/FRZB/SEMA4B/DCUN1D3/SFRP1/ENO1/SPP1/GJA1/BST2/CDKN1A/NPPA | 12 |
| BP | GO:1900542 | regulation of purine nucleotide metabolic process | 13/473 | 194/17653 | 0.0022598 | 0.0221705 | 0.0161789 | STAT3/PDE5A/DDIT4/ENO1/FZD2/SNCA/PDE4D/CAP1/RAMP1/NPR3/THBS1/GUCA1C/APOE | 13 |
| BP | GO:0010165 | response to X-ray | 5/473 | 35/17653 | 0.0022603 | 0.0221705 | 0.0161789 | CCND2/SFRP1/CCND1/CDKN1A/HAMP | 5 |
| BP | GO:0035909 | aorta morphogenesis | 5/473 | 35/17653 | 0.0022603 | 0.0221705 | 0.0161789 | SOX4/PDGFRB/ADAMTS9/PROX1/HEY2 | 5 |
| BP | GO:2000249 | regulation of actin cytoskeleton reorganization | 5/473 | 35/17653 | 0.0022603 | 0.0221705 | 0.0161789 | FES/HCK/HCLS1/ARHGDIA/PDGFRA | 5 |
| BP | GO:0030218 | erythrocyte differentiation | 9/473 | 107/17653 | 0.0023252 | 0.0225669 | 0.0164682 | ZFP36/MAFB/JAK2/STAT3/HCLS1/BCL6/HMGB2/TGFBR3/KLF2 | 9 |
| BP | GO:0031638 | zymogen activation | 6/473 | 51/17653 | 0.0023257 | 0.0225669 | 0.0164682 | C1R/ENO1/SERPINE1/PLAU/IFI16/THBS1 | 6 |
| BP | GO:0032371 | regulation of sterol transport | 6/473 | 51/17653 | 0.0023257 | 0.0225669 | 0.0164682 | NFKBIA/PLTP/APOA1/ANXA2/LIPG/APOE | 6 |
| BP | GO:0032374 | regulation of cholesterol transport | 6/473 | 51/17653 | 0.0023257 | 0.0225669 | 0.0164682 | NFKBIA/PLTP/APOA1/ANXA2/LIPG/APOE | 6 |
| BP | GO:0045010 | actin nucleation | 6/473 | 51/17653 | 0.0023257 | 0.0225669 | 0.0164682 | CORO1A/ARPC1B/WAS/LMOD2/ARPC3/ARPC5L | 6 |
| BP | GO:0097553 | calcium ion transmembrane import into cytosol | 10/473 | 128/17653 | 0.0023537 | 0.0227638 | 0.0166119 | CORO1A/CYBA/DDIT3/ATP1A2/ITPR3/SNCA/PDE4D/RYR2/CASQ1/THY1 | 10 |
| BP | GO:0050707 | regulation of cytokine secretion | 12/473 | 172/17653 | 0.0023596 | 0.0227638 | 0.0166119 | CD14/MBP/SRGN/HAVCR2/FGR/GSDMD/FCN1/SOCS1/LRRC32/APOA1/IL1RL1/POSTN | 12 |
| BP | GO:0051403 | stress-activated MAPK cascade | 16/473 | 267/17653 | 0.0023611 | 0.0227638 | 0.0166119 | ZFP36/MAP3K6/MYC/FGD2/MAP3K8/SFRP1/LTBR/MDFIC/TPD52L1/GADD45G/MAP2K1/TRIB1/GADD45A/APP/PTPN1/GADD45B | 16 |
| BP | GO:0010212 | response to ionizing radiation | 11/473 | 150/17653 | 0.002401 | 0.0230493 | 0.0168202 | MYC/CYBA/CCND2/DCUN1D3/SFRP1/CCND1/IFI16/GADD45A/CDKN1A/HAMP/EGR1 | 11 |
| BP | GO:0035051 | cardiocyte differentiation | 11/473 | 150/17653 | 0.002401 | 0.0230493 | 0.0168202 | MYH6/SORBS2/PRICKLE1/PDGFRB/SOX18/PDGFRA/TGFBR3/PROX1/HAMP/HEY2/NPPA | 11 |
| BP | GO:0042445 | hormone metabolic process | 14/473 | 219/17653 | 0.0024117 | 0.0231029 | 0.0168593 | TIPARP/ACE2/CRYM/ATP1A1/RDH10/DIO2/RDH5/SPP1/APOA1/ALDH1A3/CTSG/CORIN/CYP1B1/EGR1 | 14 |
| BP | GO:0050792 | regulation of viral process | 13/473 | 196/17653 | 0.0024718 | 0.0235295 | 0.0171706 | ZFP36/FCN3/IFITM2/IFITM3/MDFIC/ISG20/FCN1/IFI16/BST2/IFITM1/PROX1/IFIT1/APOE | 13 |
| BP | GO:0045069 | regulation of viral genome replication | 8/473 | 88/17653 | 0.0024718 | 0.0235295 | 0.0171706 | IFITM2/IFITM3/ISG20/IFI16/BST2/IFITM1/PROX1/IFIT1 | 8 |
| BP | GO:0045638 | negative regulation of myeloid cell differentiation | 8/473 | 88/17653 | 0.0024718 | 0.0235295 | 0.0171706 | ZFP36/MYC/MAFB/NFKBIA/C1QC/SFRP1/TRIB1/NME1 | 8 |
| BP | GO:0019218 | regulation of steroid metabolic process | 9/473 | 108/17653 | 0.002478 | 0.0235387 | 0.0171774 | ATP1A1/TM7SF2/FASN/LDLR/APOA1/SCD/PROX1/APOE/EGR1 | 9 |
| BP | GO:0035904 | aorta development | 6/473 | 52/17653 | 0.0025707 | 0.0243679 | 0.0177825 | PRICKLE1/SOX4/PDGFRB/ADAMTS9/PROX1/HEY2 | 6 |
| BP | GO:0007259 | JAK-STAT cascade | 12/473 | 174/17653 | 0.0025964 | 0.0244569 | 0.0178474 | JAK2/STAT3/HCLS1/BCL3/TNFRSF1A/HGS/SOCS1/CCL2/SOCS2/IL6/PTPN1/CYP1B1 | 12 |
| BP | GO:0071772 | response to BMP | 12/473 | 174/17653 | 0.0025964 | 0.0244569 | 0.0178474 | MYH6/SFRP1/HTRA1/VWC2/RGMA/SFRP4/TGFBR3/ID1/RYR2/SULF1/CYR61/EGR1 | 12 |
| BP | GO:0071773 | cellular response to BMP stimulus | 12/473 | 174/17653 | 0.0025964 | 0.0244569 | 0.0178474 | MYH6/SFRP1/HTRA1/VWC2/RGMA/SFRP4/TGFBR3/ID1/RYR2/SULF1/CYR61/EGR1 | 12 |
| BP | GO:0007044 | cell-substrate junction assembly | 8/473 | 89/17653 | 0.0026539 | 0.0248888 | 0.0181626 | S100A10/SORBS1/SFRP1/PHLDB2/COL16A1/ITGA5/THBS1/THY1 | 8 |
| BP | GO:0007193 | adenylate cyclase-inhibiting G-protein coupled receptor signaling pathway | 8/473 | 89/17653 | 0.0026539 | 0.0248888 | 0.0181626 | S1PR3/MARCO/EDNRA/GRM1/ADCY3/RGS1/NPR3/RGS2 | 8 |
| BP | GO:0071774 | response to fibroblast growth factor | 11/473 | 152/17653 | 0.0026623 | 0.0248888 | 0.0181626 | ZFP36/SFRP1/CCL2/SNCA/SULF1/CD44/TNC/THBS1/FGF18/POSTN/FGF12 | 11 |
| BP | GO:0050708 | regulation of protein secretion | 22/473 | 426/17653 | 0.0026803 | 0.0248888 | 0.0181626 | JAK2/CD14/MBP/SRGN/HAVCR2/MIDN/FGR/SFRP1/SLC2A1/GSDMD/FCN1/SOX4/KCNA5/SOCS1/ITPR3/LRRC32/OXCT1/APOA1/GJA1/IL1RL1/IL6/POSTN | 22 |
| BP | GO:0050863 | regulation of T cell activation | 18/473 | 321/17653 | 0.0026849 | 0.0248888 | 0.0181626 | CORO1A/BCL6/CEBPB/HAVCR2/CD209/MAP3K8/PDE5A/PRKCQ/AIF1/RAC2/GPNMB/SOCS1/CCL2/LRRC32/HLA-DRB4/IL6/TFRC/THY1 | 18 |
| BP | GO:0002523 | leukocyte migration involved in inflammatory response | 3/473 | 11/17653 | 0.0026864 | 0.0248888 | 0.0181626 | S100A9/S100A8/ITGB2 | 3 |
| BP | GO:0070586 | cell-cell adhesion involved in gastrulation | 3/473 | 11/17653 | 0.0026864 | 0.0248888 | 0.0181626 | MBP/KLF4/APOA1 | 3 |
| BP | GO:1990440 | positive regulation of transcription from RNA polymerase II promoter in response to endoplasmic reticulum stress | 3/473 | 11/17653 | 0.0026864 | 0.0248888 | 0.0181626 | CEBPB/DDIT3/ATF3 | 3 |
| BP | GO:2000045 | regulation of G1/S transition of mitotic cell cycle | 12/473 | 175/17653 | 0.0027218 | 0.0251171 | 0.0183292 | BTG2/CCND2/DCUN1D3/CCND1/PLK2/AIF1/KLF4/SOX4/GPNMB/KCNA5/GADD45A/CDKN1A | 12 |
| BP | GO:0030336 | negative regulation of cell migration | 17/473 | 296/17653 | 0.0027277 | 0.0251171 | 0.0183292 | STAT3/SFRP1/PHLDB2/SERPINE1/AIF1/KLF4/CCL2/ADAMTS9/TRIB1/BST2/IFITM1/TIMP1/SULF1/THBS1/THY1/CYP1B1/APOE | 17 |
| BP | GO:0072330 | monocarboxylic acid biosynthetic process | 17/473 | 296/17653 | 0.0027277 | 0.0251171 | 0.0183292 | GALK1/ALOX5/STAT3/MGST1/ALOX5AP/DDIT4/ENO1/TBXAS1/RDH10/LDHA/PDK4/FASN/APOA1/ALDH1A3/SCD/PROX1/ACOX2 | 17 |
| BP | GO:0043434 | response to peptide hormone | 22/473 | 427/17653 | 0.0027575 | 0.0253395 | 0.0184915 | PRKCD/JAK2/STAT3/RANGAP1/BTG2/CYBA/SORBS1/ERRFI1/SLC2A1/CYBB/ADCY3/PRKCQ/PDK4/SOCS1/TIMP4/TGFBR3/GJA1/SOCS2/TIMP1/PTPN1/EGR1/NPPA | 22 |
| BP | GO:0045471 | response to ethanol | 10/473 | 131/17653 | 0.0027884 | 0.0255721 | 0.0186612 | S100A8/STAT3/CD14/CCND1/CYBB/OXCT1/CA3/HAMP/TNC/PENK | 10 |
| BP | GO:0043550 | regulation of lipid kinase activity | 6/473 | 53/17653 | 0.0028346 | 0.0257362 | 0.018781 | PIK3IP1/FGR/KLF4/PDGFRB/PDGFRA/TNFAIP8L3 | 6 |
| BP | GO:0071398 | cellular response to fatty acid | 6/473 | 53/17653 | 0.0028346 | 0.0257362 | 0.018781 | SFRP1/PDK4/ID3/LDLR/NME1/TNC | 6 |
| BP | GO:1903428 | positive regulation of reactive oxygen species biosynthetic process | 6/473 | 53/17653 | 0.0028346 | 0.0257362 | 0.018781 | JAK2/ITGB2/CYBA/AIF1/KLF4/KLF2 | 6 |
| BP | GO:0003073 | regulation of systemic arterial blood pressure | 8/473 | 90/17653 | 0.0028463 | 0.0257362 | 0.018781 | CYBA/ACE2/PDE4D/GJA1/CTSG/CORIN/POSTN/NPPA | 8 |
| BP | GO:0032231 | regulation of actin filament bundle assembly | 8/473 | 90/17653 | 0.0028463 | 0.0257362 | 0.018781 | WAS/S100A10/PLEK/SFRP1/PHLDB2/SYNPO2L/APOA1/ID1 | 8 |
| BP | GO:0060840 | artery development | 8/473 | 90/17653 | 0.0028463 | 0.0257362 | 0.018781 | PRICKLE1/SOX4/PDGFRB/ADAMTS9/PRRX1/PROX1/HEY2/APOE | 8 |
| BP | GO:0097006 | regulation of plasma lipoprotein particle levels | 8/473 | 90/17653 | 0.0028463 | 0.0257362 | 0.018781 | PLTP/PLA2G2A/LPCAT3/LDLR/APOA1/ANXA2/LIPG/APOE | 8 |
| BP | GO:0097696 | STAT cascade | 12/473 | 176/17653 | 0.0028522 | 0.0257382 | 0.0187824 | JAK2/STAT3/HCLS1/BCL3/TNFRSF1A/HGS/SOCS1/CCL2/SOCS2/IL6/PTPN1/CYP1B1 | 12 |
| BP | GO:0001953 | negative regulation of cell-matrix adhesion | 5/473 | 37/17653 | 0.0029049 | 0.0261615 | 0.0190914 | BCL6/PHLDB2/SERPINE1/THBS1/POSTN | 5 |
| BP | GO:0051346 | negative regulation of hydrolase activity | 23/473 | 456/17653 | 0.002912 | 0.0261732 | 0.0190999 | SERPINA3/PROS1/SERPINA5/PLEKHF1/SERPINH1/SERPINE1/KLF4/IFI16/PPP1R14B/SPOCK1/TIMP4/SNCA/APOA1/RGS2/PICALM/SERPINB1/COL6A3/BST2/TIMP1/APP/CD44/IFIT1/THBS1 | 23 |
| BP | GO:0051607 | defense response to virus | 14/473 | 224/17653 | 0.0029616 | 0.026485 | 0.0193274 | FCN3/IFITM2/IFITM3/DDIT4/HTRA1/RNASE2/ISG20/IFI16/TSPAN32/IFIT2/BST2/IFITM1/IL6/IFIT1 | 14 |
| BP | GO:1903039 | positive regulation of leukocyte cell-cell adhesion | 14/473 | 224/17653 | 0.0029616 | 0.026485 | 0.0193274 | CORO1A/BCL6/HAVCR2/CD209/MAP3K8/PRKCQ/AIF1/SOCS1/CCL2/HLA-DRB4/IL6/CD44/TFRC/THY1 | 14 |
| BP | GO:0006066 | alcohol metabolic process | 18/473 | 324/17653 | 0.0029643 | 0.026485 | 0.0193274 | GALK1/INPP1/PLCE1/PLEK/RDH10/CEL/TM7SF2/SNCA/RDH5/FASN/LDLR/APOA1/ALDH1A3/SCD/APP/CYP1B1/APOE/ADH1A | 18 |
| BP | GO:0045862 | positive regulation of proteolysis | 19/473 | 350/17653 | 0.0029703 | 0.0264866 | 0.0193286 | S100A9/MYC/S100A8/JAK2/CTSC/STAT3/MBP/PRICKLE1/NKD2/ENO1/PLK2/IFI16/SNCA/TRIB1/PICALM/APP/CYR61/APOE/C6 | 19 |
| BP | GO:0042594 | response to starvation | 12/473 | 177/17653 | 0.0029876 | 0.0264969 | 0.0193361 | ZFP36/DDIT3/SFRP1/PLEKHF1/SLC2A1/PDK4/IFI16/OXCT1/CDKN1A/ATF3/HAMP/SLC38A2 | 12 |
| BP | GO:0035456 | response to interferon-beta | 4/473 | 23/17653 | 0.0030067 | 0.0264969 | 0.0193361 | IFITM2/IFITM3/BST2/IFITM1 | 4 |
| BP | GO:0043032 | positive regulation of macrophage activation | 4/473 | 23/17653 | 0.0030067 | 0.0264969 | 0.0193361 | HAVCR2/IL1RL1/APP/THBS1 | 4 |
| BP | GO:0051043 | regulation of membrane protein ectodomain proteolysis | 4/473 | 23/17653 | 0.0030067 | 0.0264969 | 0.0193361 | TIMP4/TIMP1/PTPN3/APOE | 4 |
| BP | GO:0060396 | growth hormone receptor signaling pathway | 4/473 | 23/17653 | 0.0030067 | 0.0264969 | 0.0193361 | JAK2/STAT3/SOCS2/PTPN1 | 4 |
| BP | GO:0140115 | export across plasma membrane | 4/473 | 23/17653 | 0.0030067 | 0.0264969 | 0.0193361 | ATP1A2/ATP1A1/ATP1B3/NPPA | 4 |
| BP | GO:0030316 | osteoclast differentiation | 8/473 | 91/17653 | 0.0030493 | 0.0268198 | 0.0195718 | MAFB/CEBPB/SFRP1/TYROBP/FOS/ANXA2/JUNB/TFRC | 8 |
| BP | GO:0006140 | regulation of nucleotide metabolic process | 13/473 | 201/17653 | 0.003074 | 0.0269842 | 0.0196917 | STAT3/PDE5A/DDIT4/ENO1/FZD2/SNCA/PDE4D/CAP1/RAMP1/NPR3/THBS1/GUCA1C/APOE | 13 |
| BP | GO:0048844 | artery morphogenesis | 7/473 | 72/17653 | 0.003115 | 0.0270524 | 0.0197415 | SOX4/PDGFRB/ADAMTS9/PRRX1/PROX1/HEY2/APOE | 7 |
| BP | GO:0001885 | endothelial cell development | 6/473 | 54/17653 | 0.0031183 | 0.0270524 | 0.0197415 | S1PR3/HEG1/TNFRSF1A/SOX18/PDE4D/ID1 | 6 |
| BP | GO:0031663 | lipopolysaccharide-mediated signaling pathway | 6/473 | 54/17653 | 0.0031183 | 0.0270524 | 0.0197415 | HCK/NFKBIA/CD14/CCL2/LY96/TRIB1 | 6 |
| BP | GO:0051893 | regulation of focal adhesion assembly | 6/473 | 54/17653 | 0.0031183 | 0.0270524 | 0.0197415 | S100A10/SFRP1/PHLDB2/COL16A1/THBS1/THY1 | 6 |
| BP | GO:0090109 | regulation of cell-substrate junction assembly | 6/473 | 54/17653 | 0.0031183 | 0.0270524 | 0.0197415 | S100A10/SFRP1/PHLDB2/COL16A1/THBS1/THY1 | 6 |
| BP | GO:0090303 | positive regulation of wound healing | 6/473 | 54/17653 | 0.0031183 | 0.0270524 | 0.0197415 | S100A9/PLEK/SERPINE1/SOX15/HBEGF/THBS1 | 6 |
| BP | GO:0030335 | positive regulation of cell migration | 24/473 | 486/17653 | 0.0031243 | 0.0270524 | 0.0197415 | JAK2/CORO1A/SEMA4B/FGR/PLK2/SERPINE1/PLAU/AIF1/THBS4/RAC2/GPNMB/PDGFRB/CXCL2/PDGFRA/ITGA5/PROX1/HBEGF/IL6/APP/CYR61/THBS1/FGF18/THY1/POSTN | 24 |
| BP | GO:0070482 | response to oxygen levels | 20/473 | 378/17653 | 0.0031297 | 0.0270524 | 0.0197415 | MYC/CYBA/DDIT4/SFRP1/EDNRA/ENO1/CYBB/PLAU/LDHA/APOLD1/KCNA5/PDGFRB/TGFBR3/RYR2/CDKN1A/THBS1/PENK/POSTN/EGR1/NPPA | 20 |
| BP | GO:0002218 | activation of innate immune response | 17/473 | 301/17653 | 0.0032374 | 0.0279296 | 0.0203817 | S100A9/PRKCD/FCER1G/S100A8/HCK/ITGB2/NFKBIA/CYBA/CD14/HAVCR2/CD209/IRAK3/MARCO/FCN1/IFI16/LY96/CTSK | 17 |
| BP | GO:0071248 | cellular response to metal ion | 11/473 | 156/17653 | 0.0032539 | 0.0279409 | 0.0203899 | MT1M/MT1X/MT1A/MT2A/ALOX5AP/CYBB/SNCA/MT1G/FOS/JUNB/MT1E | 11 |
| BP | GO:0045089 | positive regulation of innate immune response | 19/473 | 353/17653 | 0.0032609 | 0.0279409 | 0.0203899 | S100A9/PRKCD/FCER1G/S100A8/HCK/ITGB2/NFKBIA/CYBA/CD14/HAVCR2/CD209/IRAK3/MARCO/HMGB2/FCN1/IFI16/LY96/PVR/CTSK | 19 |
| BP | GO:0015918 | sterol transport | 8/473 | 92/17653 | 0.0032635 | 0.0279409 | 0.0203899 | NFKBIA/PLTP/CEL/LDLR/APOA1/ANXA2/LIPG/APOE | 8 |
| BP | GO:0050830 | defense response to Gram-positive bacterium | 8/473 | 92/17653 | 0.0032635 | 0.0279409 | 0.0203899 | HAVCR2/FGR/GSDMD/HMGB2/PLA2G2A/CTSG/IL6/LYZ | 8 |
| BP | GO:0070296 | sarcoplasmic reticulum calcium ion transport | 5/473 | 38/17653 | 0.0032726 | 0.0279663 | 0.0204084 | ATP1A2/ATP2A2/PDE4D/RYR2/CASQ1 | 5 |
| BP | GO:0045665 | negative regulation of neuron differentiation | 13/473 | 203/17653 | 0.0033461 | 0.0285401 | 0.0208271 | SEMA4B/RGMA/PLK2/TRAK2/ID3/ARHGDIA/SPOCK1/SPP1/ID1/APP/IRX3/THY1/APOE | 13 |
| BP | GO:0003208 | cardiac ventricle morphogenesis | 7/473 | 73/17653 | 0.0033677 | 0.0285622 | 0.0208432 | MYH6/HEG1/SOX4/TGFBR3/RYR2/PROX1/HEY2 | 7 |
| BP | GO:0007045 | cell-substrate adherens junction assembly | 7/473 | 73/17653 | 0.0033677 | 0.0285622 | 0.0208432 | S100A10/SORBS1/SFRP1/PHLDB2/COL16A1/THBS1/THY1 | 7 |
| BP | GO:0048041 | focal adhesion assembly | 7/473 | 73/17653 | 0.0033677 | 0.0285622 | 0.0208432 | S100A10/SORBS1/SFRP1/PHLDB2/COL16A1/THBS1/THY1 | 7 |
| BP | GO:0060401 | cytosolic calcium ion transport | 11/473 | 157/17653 | 0.0034172 | 0.0289189 | 0.0211035 | CORO1A/CYBA/DDIT3/ATP1A2/ATP2A2/ITPR3/SNCA/PDE4D/RYR2/CASQ1/THY1 | 11 |
| BP | GO:0032722 | positive regulation of chemokine production | 6/473 | 55/17653 | 0.0034226 | 0.0289189 | 0.0211035 | MBP/HAVCR2/IL1RL1/IL6/POSTN/EGR1 | 6 |
| BP | GO:0002224 | toll-like receptor signaling pathway | 10/473 | 135/17653 | 0.0034654 | 0.028981 | 0.0211489 | S100A9/S100A8/ITGB2/NFKBIA/CYBA/CD14/HAVCR2/IRAK3/LY96/CTSK | 10 |
| BP | GO:0016485 | protein processing | 18/473 | 329/17653 | 0.0034838 | 0.028981 | 0.0211489 | C1R/PROS1/C1QC/C1QB/SRGN/ACE2/NKD2/ENO1/SERPINE1/PLAU/CD59/IFI16/SPPL2A/CTSG/CORIN/AEBP1/THBS1/C6 | 18 |
| BP | GO:0015748 | organophosphate ester transport | 8/473 | 93/17653 | 0.0034892 | 0.028981 | 0.0211489 | PRKCD/PLTP/LDLR/APOA1/GJA1/TNFAIP8L3/TMEM30B/APOE | 8 |
| BP | GO:0031623 | receptor internalization | 8/473 | 93/17653 | 0.0034892 | 0.028981 | 0.0211489 | FCER1G/ITGB2/SNCA/SFRP4/RAMP1/PICALM/SH3GL2/TFRC | 8 |
| BP | GO:0001867 | complement activation, lectin pathway | 3/473 | 12/17653 | 0.003511 | 0.028981 | 0.0211489 | FCN3/MASP1/FCN1 | 3 |
| BP | GO:0010755 | regulation of plasminogen activation | 3/473 | 12/17653 | 0.003511 | 0.028981 | 0.0211489 | ENO1/SERPINE1/THBS1 | 3 |
| BP | GO:0032119 | sequestering of zinc ion | 3/473 | 12/17653 | 0.003511 | 0.028981 | 0.0211489 | S100A9/S100A8/SLC30A2 | 3 |
| BP | GO:0034384 | high-density lipoprotein particle clearance | 3/473 | 12/17653 | 0.003511 | 0.028981 | 0.0211489 | APOA1/LIPG/APOE | 3 |
| BP | GO:0042340 | keratan sulfate catabolic process | 3/473 | 12/17653 | 0.003511 | 0.028981 | 0.0211489 | LUM/OMD/FMOD | 3 |
| BP | GO:0050665 | hydrogen peroxide biosynthetic process | 3/473 | 12/17653 | 0.003511 | 0.028981 | 0.0211489 | STAT3/CYBA/CYBB | 3 |
| BP | GO:0072567 | chemokine (C-X-C motif) ligand 2 production | 3/473 | 12/17653 | 0.003511 | 0.028981 | 0.0211489 | MBP/KLF4/POSTN | 3 |
| BP | GO:0090197 | positive regulation of chemokine secretion | 3/473 | 12/17653 | 0.003511 | 0.028981 | 0.0211489 | MBP/IL1RL1/POSTN | 3 |
| BP | GO:0002719 | negative regulation of cytokine production involved in immune response | 4/473 | 24/17653 | 0.0035327 | 0.028981 | 0.0211489 | BCL6/IRAK3/APOA1/BST2 | 4 |
| BP | GO:0010640 | regulation of platelet-derived growth factor receptor signaling pathway | 4/473 | 24/17653 | 0.0035327 | 0.028981 | 0.0211489 | HGS/PDGFRB/PDGFRA/SNCA | 4 |
| BP | GO:0051894 | positive regulation of focal adhesion assembly | 4/473 | 24/17653 | 0.0035327 | 0.028981 | 0.0211489 | S100A10/SFRP1/COL16A1/THY1 | 4 |
| BP | GO:0071378 | cellular response to growth hormone stimulus | 4/473 | 24/17653 | 0.0035327 | 0.028981 | 0.0211489 | JAK2/STAT3/SOCS2/PTPN1 | 4 |
| BP | GO:0045667 | regulation of osteoblast differentiation | 9/473 | 114/17653 | 0.0035698 | 0.0292268 | 0.0213283 | CEBPB/SFRP1/ID3/CLIC1/GJA1/ID1/IFITM1/IL6/CYR61 | 9 |
| BP | GO:0002221 | pattern recognition receptor signaling pathway | 12/473 | 181/17653 | 0.0035821 | 0.0292268 | 0.0213283 | S100A9/S100A8/ITGB2/NFKBIA/CYBA/CD14/HAVCR2/IRAK3/MARCO/FCN1/LY96/CTSK | 12 |
| BP | GO:0008064 | regulation of actin polymerization or depolymerization | 12/473 | 181/17653 | 0.0035821 | 0.0292268 | 0.0213283 | CDC42EP4/PRKCD/CORO1A/ARPC1B/HCK/WAS/HCLS1/LMOD2/PLEK/ARPC3/RAC2/ARPC5L | 12 |
| BP | GO:2001233 | regulation of apoptotic signaling pathway | 20/473 | 383/17653 | 0.0036271 | 0.0295409 | 0.0215575 | S100A9/PRKCD/S100A8/JAK2/CTSC/IVNS1ABP/DDIT3/SFRP1/LTBR/PLEKHF1/ENO1/TMBIM1/HMGB2/SERPINE1/TPD52L1/MLLT11/ATF3/PTPN1/CD44/THBS1 | 20 |
| BP | GO:0032642 | regulation of chemokine production | 7/473 | 74/17653 | 0.0036358 | 0.0295579 | 0.0215698 | MBP/HAVCR2/KLF4/IL1RL1/IL6/POSTN/EGR1 | 7 |
| BP | GO:0032941 | secretion by tissue | 5/473 | 39/17653 | 0.0036727 | 0.0297511 | 0.0217109 | CYBA/VAMP8/CEL/NPR3/GJA1 | 5 |
| BP | GO:0099622 | cardiac muscle cell membrane repolarization | 5/473 | 39/17653 | 0.0036727 | 0.0297511 | 0.0217109 | ATP1A1/KCNA5/KCNJ2/GJA1/NPPA | 5 |
| BP | GO:0050678 | regulation of epithelial cell proliferation | 19/473 | 357/17653 | 0.0036848 | 0.0297955 | 0.0217433 | ZFP36/MYC/STAT3/CYBA/OSR1/SFRP1/CCND1/ERRFI1/HTRA1/HMGB2/THBS4/TGFBR3/GJA1/ID1/PROX1/NME1/SULF1/THBS1/APOE | 19 |
| BP | GO:0022617 | extracellular matrix disassembly | 8/473 | 94/17653 | 0.0037267 | 0.0300267 | 0.021912 | LCP1/CAPN1/HTRA1/SPP1/CTSK/CTSG/TIMP1/CD44 | 8 |
| BP | GO:0055017 | cardiac muscle tissue growth | 8/473 | 94/17653 | 0.0037267 | 0.0300267 | 0.021912 | SORBS2/PIM1/HEG1/TGFBR3/GJA1/HAMP/HEY2/NPPA | 8 |
| BP | GO:0030832 | regulation of actin filament length | 12/473 | 182/17653 | 0.0037447 | 0.0300407 | 0.0219222 | CDC42EP4/PRKCD/CORO1A/ARPC1B/HCK/WAS/HCLS1/LMOD2/PLEK/ARPC3/RAC2/ARPC5L | 12 |
| BP | GO:0072376 | protein activation cascade | 12/473 | 182/17653 | 0.0037447 | 0.0300407 | 0.0219222 | FCN3/C1R/PROS1/F13A1/C1QC/C1QB/MASP1/VSIG4/CD59/FCN1/CFD/C6 | 12 |
| BP | GO:0014888 | striated muscle adaptation | 6/473 | 56/17653 | 0.0037484 | 0.0300407 | 0.0219222 | MYH6/ERRFI1/TNNT1/ATP2A2/HEY2/NPPA | 6 |
| BP | GO:0006956 | complement activation | 11/473 | 159/17653 | 0.0037634 | 0.0301071 | 0.0219706 | FCN3/C1R/PROS1/C1QC/C1QB/MASP1/VSIG4/CD59/FCN1/CFD/C6 | 11 |
| BP | GO:0034101 | erythrocyte homeostasis | 9/473 | 115/17653 | 0.0037837 | 0.0302158 | 0.02205 | ZFP36/MAFB/JAK2/STAT3/HCLS1/BCL6/HMGB2/TGFBR3/KLF2 | 9 |
| BP | GO:1903900 | regulation of viral life cycle | 10/473 | 137/17653 | 0.0038496 | 0.0306881 | 0.0223946 | FCN3/IFITM2/IFITM3/ISG20/FCN1/IFI16/BST2/IFITM1/PROX1/IFIT1 | 10 |
| BP | GO:0060420 | regulation of heart growth | 7/473 | 75/17653 | 0.0039198 | 0.0311922 | 0.0227625 | MYH6/PIM1/TGFBR3/GJA1/PROX1/HAMP/HEY2 | 7 |
| BP | GO:0022604 | regulation of cell morphogenesis | 23/473 | 468/17653 | 0.004001 | 0.0317776 | 0.0231897 | CDC42EP4/FES/CHN1/CORO1A/HCK/WAS/ITGB2/FGD2/S100A10/SEMA4B/FGR/S100A13/TRAK2/CCL2/ARHGDIA/MAP2K1/SPP1/APOA1/ID1/CD44/THY1/POSTN/APOE | 23 |
| BP | GO:2000134 | negative regulation of G1/S transition of mitotic cell cycle | 9/473 | 116/17653 | 0.0040074 | 0.0317776 | 0.0231897 | BTG2/DCUN1D3/CCND1/PLK2/KLF4/SOX4/GPNMB/GADD45A/CDKN1A | 9 |
| BP | GO:0010977 | negative regulation of neuron projection development | 10/473 | 138/17653 | 0.004054 | 0.0319785 | 0.0233363 | SEMA4B/RGMA/PLK2/TRAK2/ARHGDIA/SPOCK1/SPP1/ID1/THY1/APOE | 10 |
| BP | GO:0032233 | positive regulation of actin filament bundle assembly | 6/473 | 57/17653 | 0.0040967 | 0.0319785 | 0.0233363 | S100A10/PLEK/SFRP1/SYNPO2L/APOA1/ID1 | 6 |
| BP | GO:0045453 | bone resorption | 6/473 | 57/17653 | 0.0040967 | 0.0319785 | 0.0233363 | PDK4/RAC2/SPP1/CTSK/IL6/TFRC | 6 |
| BP | GO:1903391 | regulation of adherens junction organization | 6/473 | 57/17653 | 0.0040967 | 0.0319785 | 0.0233363 | S100A10/SFRP1/PHLDB2/COL16A1/THBS1/THY1 | 6 |
| BP | GO:0050832 | defense response to fungus | 5/473 | 40/17653 | 0.0041067 | 0.0319785 | 0.0233363 | S100A9/S100A8/S100A12/CTSG/HAMP | 5 |
| BP | GO:0060412 | ventricular septum morphogenesis | 5/473 | 40/17653 | 0.0041067 | 0.0319785 | 0.0233363 | FZD2/SOX4/TGFBR3/PROX1/HEY2 | 5 |
| BP | GO:0001774 | microglial cell activation | 4/473 | 25/17653 | 0.0041178 | 0.0319785 | 0.0233363 | AIF1/SNCA/LDLR/APP | 4 |
| BP | GO:0007263 | nitric oxide mediated signal transduction | 4/473 | 25/17653 | 0.0041178 | 0.0319785 | 0.0233363 | RASD1/FPR1/THBS1/APOE | 4 |
| BP | GO:0048679 | regulation of axon regeneration | 4/473 | 25/17653 | 0.0041178 | 0.0319785 | 0.0233363 | RGMA/KLF4/MAP2K1/SPP1 | 4 |
| BP | GO:0055094 | response to lipoprotein particle | 4/473 | 25/17653 | 0.0041178 | 0.0319785 | 0.0233363 | FCER1G/ITGB2/LDLR/APOE | 4 |
| BP | GO:1903319 | positive regulation of protein maturation | 4/473 | 25/17653 | 0.0041178 | 0.0319785 | 0.0233363 | NKD2/ENO1/SOX4/C6 | 4 |
| BP | GO:1905048 | regulation of metallopeptidase activity | 4/473 | 25/17653 | 0.0041178 | 0.0319785 | 0.0233363 | STAT3/MBP/PICALM/TIMP1 | 4 |
| BP | GO:0048017 | inositol lipid-mediated signaling | 11/473 | 161/17653 | 0.0041371 | 0.032073 | 0.0234053 | PIK3IP1/JAK2/HCLS1/FGR/HCST/KLF4/IER3/PDGFRB/PDGFRA/NPR3/TNFAIP8L3 | 11 |
| BP | GO:0002758 | innate immune response-activating signal transduction | 16/473 | 283/17653 | 0.0041766 | 0.0323235 | 0.0235881 | S100A9/PRKCD/FCER1G/S100A8/HCK/ITGB2/NFKBIA/CYBA/CD14/HAVCR2/CD209/IRAK3/MARCO/FCN1/LY96/CTSK | 16 |
| BP | GO:1904035 | regulation of epithelial cell apoptotic process | 7/473 | 76/17653 | 0.0042203 | 0.0326059 | 0.0237942 | ZFP36/JAK2/CD248/SERPINE1/SFRP4/IL6/THBS1 | 7 |
| BP | GO:0002286 | T cell activation involved in immune response | 8/473 | 96/17653 | 0.0042392 | 0.0326963 | 0.0238602 | FCER1G/STAT3/LCP1/IL18R1/BCL6/HAVCR2/BCL3/IL6 | 8 |
| BP | GO:0048705 | skeletal system morphogenesis | 13/473 | 209/17653 | 0.0042821 | 0.0329707 | 0.0240603 | TIPARP/OSR1/SLC39A1/SFRP1/SERPINH1/RDH10/CHSY1/PDGFRA/SFRP4/PRRX1/ALPL/COMP/FGF18 | 13 |
| BP | GO:0030833 | regulation of actin filament polymerization | 11/473 | 162/17653 | 0.0043347 | 0.0333188 | 0.0243144 | CDC42EP4/PRKCD/CORO1A/ARPC1B/HCK/WAS/HCLS1/LMOD2/ARPC3/RAC2/ARPC5L | 11 |
| BP | GO:0032412 | regulation of ion transmembrane transporter activity | 14/473 | 234/17653 | 0.0043711 | 0.0334754 | 0.0244287 | SCN2B/CAPN1/OSR1/ATP1A2/HSPA2/PDE4D/ATP1B3/RYR2/CASQ1/APP/KLHL24/PTPN3/FGF12/NPPA | 14 |
| BP | GO:0002793 | positive regulation of peptide secretion | 15/473 | 259/17653 | 0.0043773 | 0.0334754 | 0.0244287 | S100A9/S100A8/JAK2/CD14/MBP/HAVCR2/FGR/GSDMD/FCN1/SOX4/OXCT1/GJA1/IL1RL1/IL6/POSTN | 15 |
| BP | GO:0050768 | negative regulation of neurogenesis | 15/473 | 259/17653 | 0.0043773 | 0.0334754 | 0.0244287 | STAT3/SEMA4B/RGMA/PLK2/TRAK2/ID3/ARHGDIA/SPOCK1/LDLR/SPP1/ID1/APP/IRX3/THY1/APOE | 15 |
| BP | GO:0051604 | protein maturation | 20/473 | 390/17653 | 0.0044328 | 0.0337592 | 0.0246358 | C1R/PROS1/C1QC/C1QB/SRGN/ACE2/NKD2/ENO1/SERPINH1/SERPINE1/PLAU/CD59/IFI16/SOX4/SPPL2A/CTSG/CORIN/AEBP1/THBS1/C6 | 20 |
| BP | GO:0017157 | regulation of exocytosis | 12/473 | 186/17653 | 0.0044557 | 0.0337592 | 0.0246358 | FCER1G/FES/ITGB2/VAMP8/FGR/HGS/RAC2/ATP2A2/SNCA/STXBP6/RAB15/NPPA | 12 |
| BP | GO:0010248 | establishment or maintenance of transmembrane electrochemical gradient | 3/473 | 13/17653 | 0.0044743 | 0.0337592 | 0.0246358 | ATP1A2/ATP1A1/ATP1B3 | 3 |
| BP | GO:0035791 | platelet-derived growth factor receptor-beta signaling pathway | 3/473 | 13/17653 | 0.0044743 | 0.0337592 | 0.0246358 | PDGFRB/PDGFRA/PTPN1 | 3 |
| BP | GO:0042574 | retinal metabolic process | 3/473 | 13/17653 | 0.0044743 | 0.0337592 | 0.0246358 | RDH10/ALDH1A3/CYP1B1 | 3 |
| BP | GO:0060452 | positive regulation of cardiac muscle contraction | 3/473 | 13/17653 | 0.0044743 | 0.0337592 | 0.0246358 | ACE2/RGS2/NPPA | 3 |
| BP | GO:0071481 | cellular response to X-ray | 3/473 | 13/17653 | 0.0044743 | 0.0337592 | 0.0246358 | CCND2/SFRP1/HAMP | 3 |
| BP | GO:0071872 | cellular response to epinephrine stimulus | 3/473 | 13/17653 | 0.0044743 | 0.0337592 | 0.0246358 | SNCA/PDE4D/RYR2 | 3 |
| BP | GO:0034250 | positive regulation of cellular amide metabolic process | 10/473 | 140/17653 | 0.0044883 | 0.0338086 | 0.0246718 | PRKCD/BCL3/TNFRSF1A/SOX4/PICALM/FAM129A/IL6/APP/CYR61/THBS1 | 10 |
| BP | GO:0010522 | regulation of calcium ion transport into cytosol | 8/473 | 97/17653 | 0.004515 | 0.0338399 | 0.0246947 | CORO1A/CYBA/ATP1A2/SNCA/PDE4D/RYR2/CASQ1/THY1 | 8 |
| BP | GO:0048010 | vascular endothelial growth factor receptor signaling pathway | 8/473 | 97/17653 | 0.004515 | 0.0338399 | 0.0246947 | CYBA/CYBB/HGS/ACTB/ITGA5/PTPN1/SULF1/FGF18 | 8 |
| BP | GO:0051591 | response to cAMP | 8/473 | 97/17653 | 0.004515 | 0.0338399 | 0.0246947 | DUSP1/LDHA/AQP9/FOS/NME1/APP/JUNB/PENK | 8 |
| BP | GO:0072210 | metanephric nephron development | 5/473 | 41/17653 | 0.0045761 | 0.0342409 | 0.0249873 | OSR1/IRX2/PDGFRB/PDGFRA/EGR1 | 5 |
| BP | GO:0051701 | interaction with host | 13/473 | 211/17653 | 0.0046373 | 0.0346416 | 0.0252797 | FCN3/IFITM2/IFITM3/VAMP8/CD209/ACE2/FCN1/PVR/LDLR/ITGA5/IFITM1/IFIT1/TFRC | 13 |
| BP | GO:0007565 | female pregnancy | 12/473 | 187/17653 | 0.0046493 | 0.0346741 | 0.0253034 | HAVCR2/SLC2A1/COL16A1/NAMPT/SPP1/GJA1/FOS/TIMP1/JUNB/CORIN/SLC38A2/NPPA | 12 |
| BP | GO:0001508 | action potential | 10/473 | 141/17653 | 0.0047188 | 0.0349588 | 0.0255112 | SCN2B/ATP1A2/ATP1A1/ATP2A2/KCNA5/KCNJ2/GJA1/RYR2/PTPN3/FGF12 | 10 |
| BP | GO:0098754 | detoxification | 9/473 | 119/17653 | 0.0047412 | 0.0349588 | 0.0255112 | S100A9/MT1M/MT1X/MT1A/MGST1/MT2A/MT1G/MT1E/APOE | 9 |
| BP | GO:0051170 | import into nucleus | 11/473 | 164/17653 | 0.0047524 | 0.0349588 | 0.0255112 | PRKCD/JAK2/STAT3/BCL6/NFKBIA/PRICKLE1/BCL3/SNRPB/NUP62CL/MDFIC/CDKN1A | 11 |
| BP | GO:0002063 | chondrocyte development | 4/473 | 26/17653 | 0.004765 | 0.0349588 | 0.0255112 | SERPINH1/CHSY1/SULF1/FGF18 | 4 |
| BP | GO:0030194 | positive regulation of blood coagulation | 4/473 | 26/17653 | 0.004765 | 0.0349588 | 0.0255112 | S100A9/PLEK/SERPINE1/THBS1 | 4 |
| BP | GO:0031639 | plasminogen activation | 4/473 | 26/17653 | 0.004765 | 0.0349588 | 0.0255112 | ENO1/SERPINE1/PLAU/THBS1 | 4 |
| BP | GO:0033687 | osteoblast proliferation | 4/473 | 26/17653 | 0.004765 | 0.0349588 | 0.0255112 | SFRP1/NPR3/JUNB/CYR61 | 4 |
| BP | GO:0045932 | negative regulation of muscle contraction | 4/473 | 26/17653 | 0.004765 | 0.0349588 | 0.0255112 | PDE5A/ATP1A2/TNNT1/RGS2 | 4 |
| BP | GO:1900048 | positive regulation of hemostasis | 4/473 | 26/17653 | 0.004765 | 0.0349588 | 0.0255112 | S100A9/PLEK/SERPINE1/THBS1 | 4 |
| BP | GO:1903393 | positive regulation of adherens junction organization | 4/473 | 26/17653 | 0.004765 | 0.0349588 | 0.0255112 | S100A10/SFRP1/COL16A1/THY1 | 4 |
| BP | GO:1902041 | regulation of extrinsic apoptotic signaling pathway via death domain receptors | 6/473 | 59/17653 | 0.0048643 | 0.0356295 | 0.0260006 | SFRP1/TMBIM1/HMGB2/SERPINE1/ATF3/THBS1 | 6 |
| BP | GO:0001570 | vasculogenesis | 7/473 | 78/17653 | 0.0048732 | 0.0356368 | 0.0260059 | TIPARP/HEG1/PDGFRB/SOX18/TGFBR3/JUNB/HEY2 | 7 |
| BP | GO:0002822 | regulation of adaptive immune response based on somatic recombination of immune receptors built from immunoglobulin superfamily domains | 9/473 | 120/17653 | 0.0050077 | 0.0363846 | 0.0265516 | FCER1G/WAS/BCL6/HAVCR2/PRKCQ/PVR/IL1RL1/IL6/TFRC | 9 |
| BP | GO:0010675 | regulation of cellular carbohydrate metabolic process | 9/473 | 120/17653 | 0.0050077 | 0.0363846 | 0.0265516 | STAT3/SORBS1/MIDN/DDIT4/PLEK/C1QTNF1/PDK4/SNCA/IGFBP4 | 9 |
| BP | GO:1903825 | organic acid transmembrane transport | 9/473 | 120/17653 | 0.0050077 | 0.0363846 | 0.0265516 | OSR1/SLC7A1/ATP1A2/SLC7A5/AQP9/CPT1B/SLC16A3/SLC38A2/THBS1 | 9 |
| BP | GO:1905039 | carboxylic acid transmembrane transport | 9/473 | 120/17653 | 0.0050077 | 0.0363846 | 0.0265516 | OSR1/SLC7A1/ATP1A2/SLC7A5/AQP9/CPT1B/SLC16A3/SLC38A2/THBS1 | 9 |
| BP | GO:0050870 | positive regulation of T cell activation | 13/473 | 213/17653 | 0.0050159 | 0.0363851 | 0.026552 | CORO1A/BCL6/HAVCR2/CD209/MAP3K8/PRKCQ/AIF1/SOCS1/CCL2/HLA-DRB4/IL6/TFRC/THY1 | 13 |
| BP | GO:0098656 | anion transmembrane transport | 15/473 | 263/17653 | 0.0050349 | 0.0364644 | 0.0266099 | LRRC8A/SLC12A9/OSR1/SLC7A1/ATP1A2/GLRB/SLC7A5/CLIC1/AQP9/CPT1B/GABRB1/CLIC6/SLC16A3/SLC38A2/THBS1 | 15 |
| BP | GO:0010107 | potassium ion import | 5/473 | 42/17653 | 0.0050824 | 0.0365737 | 0.0266896 | SLC12A9/ATP1A2/ATP1A1/KCNJ2/ATP1B3 | 5 |
| BP | GO:0045429 | positive regulation of nitric oxide biosynthetic process | 5/473 | 42/17653 | 0.0050824 | 0.0365737 | 0.0266896 | JAK2/ITGB2/AIF1/KLF4/KLF2 | 5 |
| BP | GO:0051602 | response to electrical stimulus | 5/473 | 42/17653 | 0.0050824 | 0.0365737 | 0.0266896 | BTG2/CD14/GRM1/S100A13/AIF1 | 5 |
| BP | GO:1904407 | positive regulation of nitric oxide metabolic process | 5/473 | 42/17653 | 0.0050824 | 0.0365737 | 0.0266896 | JAK2/ITGB2/AIF1/KLF4/KLF2 | 5 |
| BP | GO:1904019 | epithelial cell apoptotic process | 8/473 | 99/17653 | 0.0051077 | 0.0366973 | 0.0267798 | ZFP36/JAK2/CD248/SERPINE1/SFRP4/RYR2/IL6/THBS1 | 8 |
| BP | GO:0035967 | cellular response to topologically incorrect protein | 11/473 | 166/17653 | 0.0052015 | 0.0372973 | 0.0272177 | TSPYL2/DDIT3/CCND1/SEC61A1/EXTL1/HSPA2/CCL2/HSPA6/ATF3/PTPN1/SDF2L1 | 11 |
| BP | GO:0006606 | protein import into nucleus | 10/473 | 143/17653 | 0.0052077 | 0.0372973 | 0.0272177 | PRKCD/JAK2/STAT3/BCL6/NFKBIA/PRICKLE1/BCL3/NUP62CL/MDFIC/CDKN1A | 10 |
| BP | GO:0008344 | adult locomotory behavior | 7/473 | 79/17653 | 0.0052268 | 0.0373748 | 0.0272742 | CCND2/ATP1A2/GLRB/SNCA/APP/UCHL1/FGF12 | 7 |
| BP | GO:0010812 | negative regulation of cell-substrate adhesion | 6/473 | 60/17653 | 0.0052854 | 0.0375575 | 0.0274076 | BCL6/PHLDB2/SERPINE1/SPOCK1/THBS1/POSTN | 6 |
| BP | GO:0045669 | positive regulation of osteoblast differentiation | 6/473 | 60/17653 | 0.0052854 | 0.0375575 | 0.0274076 | CEBPB/CLIC1/GJA1/IFITM1/IL6/CYR61 | 6 |
| BP | GO:0003231 | cardiac ventricle development | 9/473 | 121/17653 | 0.0052857 | 0.0375575 | 0.0274076 | MYH6/HEG1/FZD2/SOX4/TGFBR3/RYR2/PROX1/CYR61/HEY2 | 9 |
| BP | GO:0051592 | response to calcium ion | 9/473 | 121/17653 | 0.0052857 | 0.0375575 | 0.0274076 | ALOX5AP/DUSP1/CCND1/ITPR3/FOS/RYR2/JUNB/THBS1/PENK | 9 |
| BP | GO:0002705 | positive regulation of leukocyte mediated immunity | 8/473 | 100/17653 | 0.0054255 | 0.0383711 | 0.0280013 | FCER1G/ITGB2/VAMP8/FGR/PVR/IL6/TFRC/NPPA | 8 |
| BP | GO:0030324 | lung development | 11/473 | 167/17653 | 0.0054383 | 0.0383711 | 0.0280013 | HEG1/ERRFI1/RDH10/PDGFRB/MAP2K1/ID1/KLF2/CRISPLD2/PROX1/TNC/FGF18 | 11 |
| BP | GO:0043270 | positive regulation of ion transport | 14/473 | 240/17653 | 0.00545 | 0.0383711 | 0.0280013 | PRKCD/VAMP8/HSPA2/PDGFRB/CCL2/SNCA/KCNJ2/ATP1B3/RYR2/CASQ1/THY1/APOE/FGF12/NPPA | 14 |
| BP | GO:1903169 | regulation of calcium ion transmembrane transport | 10/473 | 144/17653 | 0.0054666 | 0.0383711 | 0.0280013 | CORO1A/CYBA/ATP1A2/HSPA2/SNCA/PDE4D/RYR2/CASQ1/THY1/NPPA | 10 |
| BP | GO:0001773 | myeloid dendritic cell activation | 4/473 | 27/17653 | 0.0054771 | 0.0383711 | 0.0280013 | DOCK2/HAVCR2/LTBR/TSPAN32 | 4 |
| BP | GO:0050820 | positive regulation of coagulation | 4/473 | 27/17653 | 0.0054771 | 0.0383711 | 0.0280013 | S100A9/PLEK/SERPINE1/THBS1 | 4 |
| BP | GO:0071402 | cellular response to lipoprotein particle stimulus | 4/473 | 27/17653 | 0.0054771 | 0.0383711 | 0.0280013 | FCER1G/ITGB2/LDLR/APOE | 4 |
| BP | GO:1903305 | regulation of regulated secretory pathway | 9/473 | 122/17653 | 0.0055755 | 0.0383711 | 0.0280013 | FCER1G/FES/ITGB2/VAMP8/FGR/RAC2/ATP2A2/RAB15/NPPA | 9 |
| BP | GO:0010310 | regulation of hydrogen peroxide metabolic process | 3/473 | 14/17653 | 0.0055824 | 0.0383711 | 0.0280013 | STAT3/RAC2/SNCA | 3 |
| BP | GO:0010875 | positive regulation of cholesterol efflux | 3/473 | 14/17653 | 0.0055824 | 0.0383711 | 0.0280013 | NFKBIA/PLTP/APOE | 3 |
| BP | GO:0030299 | intestinal cholesterol absorption | 3/473 | 14/17653 | 0.0055824 | 0.0383711 | 0.0280013 | CEL/LDLR/APOA1 | 3 |
| BP | GO:0032930 | positive regulation of superoxide anion generation | 3/473 | 14/17653 | 0.0055824 | 0.0383711 | 0.0280013 | PRKCD/ITGB2/CYBA | 3 |
| BP | GO:0042159 | lipoprotein catabolic process | 3/473 | 14/17653 | 0.0055824 | 0.0383711 | 0.0280013 | LYPLAL1/LDLR/APOE | 3 |
| BP | GO:0051770 | positive regulation of nitric-oxide synthase biosynthetic process | 3/473 | 14/17653 | 0.0055824 | 0.0383711 | 0.0280013 | JAK2/CCL2/NAMPT | 3 |
| BP | GO:0072109 | glomerular mesangium development | 3/473 | 14/17653 | 0.0055824 | 0.0383711 | 0.0280013 | GPR4/PDGFRB/EGR1 | 3 |
| BP | GO:0072216 | positive regulation of metanephros development | 3/473 | 14/17653 | 0.0055824 | 0.0383711 | 0.0280013 | MYC/PDGFRB/EGR1 | 3 |
| BP | GO:0090153 | regulation of sphingolipid biosynthetic process | 3/473 | 14/17653 | 0.0055824 | 0.0383711 | 0.0280013 | PRKCD/TNFRSF1A/CYR61 | 3 |
| BP | GO:0090196 | regulation of chemokine secretion | 3/473 | 14/17653 | 0.0055824 | 0.0383711 | 0.0280013 | MBP/IL1RL1/POSTN | 3 |
| BP | GO:1905038 | regulation of membrane lipid metabolic process | 3/473 | 14/17653 | 0.0055824 | 0.0383711 | 0.0280013 | PRKCD/TNFRSF1A/CYR61 | 3 |
| BP | GO:2000303 | regulation of ceramide biosynthetic process | 3/473 | 14/17653 | 0.0055824 | 0.0383711 | 0.0280013 | PRKCD/TNFRSF1A/CYR61 | 3 |
| BP | GO:0046427 | positive regulation of JAK-STAT cascade | 7/473 | 80/17653 | 0.0055994 | 0.0383711 | 0.0280013 | JAK2/STAT3/HCLS1/TNFRSF1A/SOCS1/IL6/CYP1B1 | 7 |
| BP | GO:0006636 | unsaturated fatty acid biosynthetic process | 5/473 | 43/17653 | 0.0056271 | 0.0383711 | 0.0280013 | ALOX5/MGST1/ALOX5AP/TBXAS1/SCD | 5 |
| BP | GO:0010470 | regulation of gastrulation | 5/473 | 43/17653 | 0.0056271 | 0.0383711 | 0.0280013 | MBP/OSR1/PHLDB2/KLF4/APOA1 | 5 |
| BP | GO:0032732 | positive regulation of interleukin-1 production | 5/473 | 43/17653 | 0.0056271 | 0.0383711 | 0.0280013 | JAK2/HAVCR2/GSDMD/IFI16/EGR1 | 5 |
| BP | GO:0042771 | intrinsic apoptotic signaling pathway in response to DNA damage by p53 class mediator | 5/473 | 43/17653 | 0.0056271 | 0.0383711 | 0.0280013 | DDIT4/BCL3/IFI16/CDKN1A/CD44 | 5 |
| BP | GO:1903727 | positive regulation of phospholipid metabolic process | 5/473 | 43/17653 | 0.0056271 | 0.0383711 | 0.0280013 | PRKCD/FGR/PDGFRB/PDGFRA/TNFAIP8L3 | 5 |
| BP | GO:0032872 | regulation of stress-activated MAPK cascade | 13/473 | 216/17653 | 0.0056298 | 0.0383711 | 0.0280013 | MAP3K6/MYC/FGD2/SFRP1/LTBR/MDFIC/TPD52L1/GADD45G/MAP2K1/GADD45A/APP/PTPN1/GADD45B | 13 |
| BP | GO:0050679 | positive regulation of epithelial cell proliferation | 12/473 | 192/17653 | 0.0057208 | 0.0388563 | 0.0283553 | MYC/STAT3/CYBA/OSR1/SFRP1/CCND1/HTRA1/HMGB2/THBS4/ID1/PROX1/NME1 | 12 |
| BP | GO:0002292 | T cell differentiation involved in immune response | 6/473 | 61/17653 | 0.0057326 | 0.0388563 | 0.0283553 | FCER1G/STAT3/IL18R1/BCL6/BCL3/IL6 | 6 |
| BP | GO:0044344 | cellular response to fibroblast growth factor stimulus | 10/473 | 145/17653 | 0.0057354 | 0.0388563 | 0.0283553 | ZFP36/SFRP1/CCL2/SNCA/SULF1/CD44/THBS1/FGF18/POSTN/FGF12 | 10 |
| BP | GO:0060402 | calcium ion transport into cytosol | 10/473 | 145/17653 | 0.0057354 | 0.0388563 | 0.0283553 | CORO1A/CYBA/DDIT3/ATP1A2/ITPR3/SNCA/PDE4D/RYR2/CASQ1/THY1 | 10 |
| BP | GO:0001666 | response to hypoxia | 18/473 | 346/17653 | 0.0058452 | 0.0394956 | 0.0288219 | MYC/CYBA/DDIT4/SFRP1/EDNRA/ENO1/CYBB/PLAU/LDHA/APOLD1/KCNA5/TGFBR3/RYR2/THBS1/PENK/POSTN/EGR1/NPPA | 18 |
| BP | GO:0070302 | regulation of stress-activated protein kinase signaling cascade | 13/473 | 217/17653 | 0.0058473 | 0.0394956 | 0.0288219 | MAP3K6/MYC/FGD2/SFRP1/LTBR/MDFIC/TPD52L1/GADD45G/MAP2K1/GADD45A/APP/PTPN1/GADD45B | 13 |
| BP | GO:0002687 | positive regulation of leukocyte migration | 9/473 | 123/17653 | 0.0058774 | 0.0395801 | 0.0288836 | SERPINE1/AIF1/THBS4/RAC2/CXCL2/IL6/APP/THBS1/THY1 | 9 |
| BP | GO:1902807 | negative regulation of cell cycle G1/S phase transition | 9/473 | 123/17653 | 0.0058774 | 0.0395801 | 0.0288836 | BTG2/DCUN1D3/CCND1/PLK2/KLF4/SOX4/GPNMB/GADD45A/CDKN1A | 9 |
| BP | GO:0046328 | regulation of JNK cascade | 11/473 | 169/17653 | 0.0059373 | 0.0398646 | 0.0290912 | MAP3K6/FGD2/SFRP1/LTBR/MDFIC/TPD52L1/GADD45G/GADD45A/APP/PTPN1/GADD45B | 11 |
| BP | GO:1903317 | regulation of protein maturation | 11/473 | 169/17653 | 0.0059373 | 0.0398646 | 0.0290912 | C1R/PROS1/C1QC/C1QB/NKD2/ENO1/SERPINE1/CD59/SOX4/THBS1/C6 | 11 |
| BP | GO:0060541 | respiratory system development | 12/473 | 193/17653 | 0.005957 | 0.039878 | 0.029101 | HEG1/ERRFI1/RDH10/PDGFRB/MAP2K1/ID1/ALDH1A3/KLF2/CRISPLD2/PROX1/TNC/FGF18 | 12 |
| BP | GO:1902806 | regulation of cell cycle G1/S phase transition | 12/473 | 193/17653 | 0.005957 | 0.039878 | 0.029101 | BTG2/CCND2/DCUN1D3/CCND1/PLK2/AIF1/KLF4/SOX4/GPNMB/KCNA5/GADD45A/CDKN1A | 12 |
| BP | GO:0045921 | positive regulation of exocytosis | 7/473 | 81/17653 | 0.0059915 | 0.0399313 | 0.0291399 | FCER1G/ITGB2/VAMP8/FGR/HGS/RAB15/NPPA | 7 |
| BP | GO:0060415 | muscle tissue morphogenesis | 7/473 | 81/17653 | 0.0059915 | 0.0399313 | 0.0291399 | MYH6/HEG1/FZD2/TGFBR3/RYR2/PROX1/HEY2 | 7 |
| BP | GO:0070542 | response to fatty acid | 7/473 | 81/17653 | 0.0059915 | 0.0399313 | 0.0291399 | SFRP1/PDK4/ID3/TGFBR3/LDLR/NME1/TNC | 7 |
| BP | GO:0044706 | multi-multicellular organism process | 13/473 | 218/17653 | 0.0060715 | 0.0404048 | 0.0294854 | HAVCR2/SLC2A1/COL16A1/NAMPT/PLA2G4C/SPP1/GJA1/FOS/TIMP1/JUNB/CORIN/SLC38A2/NPPA | 13 |
| BP | GO:0003206 | cardiac chamber morphogenesis | 9/473 | 124/17653 | 0.0061917 | 0.0410839 | 0.029981 | MYH6/HEG1/FZD2/SOX4/TGFBR3/RYR2/PROX1/CYR61/HEY2 | 9 |
| BP | GO:0032963 | collagen metabolic process | 9/473 | 124/17653 | 0.0061917 | 0.0410839 | 0.029981 | ERRFI1/SERPINH1/COL6A2/PDGFRB/ID1/CTSK/COL6A3/MFAP4/IL6 | 9 |
| BP | GO:0060193 | positive regulation of lipase activity | 6/473 | 62/17653 | 0.0062068 | 0.0410958 | 0.0299896 | GNA15/EDNRA/PDGFRB/PDGFRA/APOA1/CYR61 | 6 |
| BP | GO:0006984 | ER-nucleus signaling pathway | 5/473 | 44/17653 | 0.0062117 | 0.0410958 | 0.0299896 | DDIT3/ATP2A2/CCL2/ATF3/PTPN1 | 5 |
| BP | GO:0048608 | reproductive structure development | 21/473 | 430/17653 | 0.0062328 | 0.0411747 | 0.0300472 | TIPARP/MGST1/CEBPB/OSR1/SERPINA5/SFRP1/CCND1/RDH10/HTRA1/HMGB2/PDGFRB/PDGFRA/MAP2K1/SPP1/GJA1/SOX15/JUNB/SULF1/CYR61/HEY2/TNC | 21 |
| BP | GO:0031098 | stress-activated protein kinase signaling cascade | 16/473 | 296/17653 | 0.0063816 | 0.0420963 | 0.0307198 | ZFP36/MAP3K6/MYC/FGD2/MAP3K8/SFRP1/LTBR/MDFIC/TPD52L1/GADD45G/MAP2K1/TRIB1/GADD45A/APP/PTPN1/GADD45B | 16 |
| BP | GO:0050810 | regulation of steroid biosynthetic process | 7/473 | 82/17653 | 0.0064037 | 0.0421186 | 0.0307361 | ATP1A1/TM7SF2/FASN/SCD/PROX1/APOE/EGR1 | 7 |
| BP | GO:1904894 | positive regulation of STAT cascade | 7/473 | 82/17653 | 0.0064037 | 0.0421186 | 0.0307361 | JAK2/STAT3/HCLS1/TNFRSF1A/SOCS1/IL6/CYP1B1 | 7 |
| BP | GO:0030323 | respiratory tube development | 11/473 | 171/17653 | 0.0064716 | 0.0425037 | 0.0310171 | HEG1/ERRFI1/RDH10/PDGFRB/MAP2K1/ID1/KLF2/CRISPLD2/PROX1/TNC/FGF18 | 11 |
| BP | GO:0032355 | response to estradiol | 9/473 | 125/17653 | 0.0065189 | 0.0427517 | 0.031198 | STAT3/ARPC1B/DUSP1/SFRP1/CCND1/PDGFRB/SOCS2/PENK/POSTN | 9 |
| BP | GO:0016331 | morphogenesis of embryonic epithelium | 10/473 | 148/17653 | 0.0066042 | 0.0432485 | 0.0315606 | OSR1/PRICKLE1/IRX2/SFRP1/RDH10/FZD2/SOX4/ALDH1A3/IRX3/SULF1 | 10 |
| BP | GO:0032677 | regulation of interleukin-8 production | 6/473 | 63/17653 | 0.0067089 | 0.0438423 | 0.0319939 | CD14/DDIT3/BCL3/SERPINE1/FCN1/KLF4 | 6 |
| BP | GO:0061458 | reproductive system development | 21/473 | 433/17653 | 0.0067267 | 0.0438423 | 0.0319939 | TIPARP/MGST1/CEBPB/OSR1/SERPINA5/SFRP1/CCND1/RDH10/HTRA1/HMGB2/PDGFRB/PDGFRA/MAP2K1/SPP1/GJA1/SOX15/JUNB/SULF1/CYR61/HEY2/TNC | 21 |
| BP | GO:0008625 | extrinsic apoptotic signaling pathway via death domain receptors | 7/473 | 83/17653 | 0.0068366 | 0.0438423 | 0.0319939 | SFRP1/TNFRSF1A/TMBIM1/HMGB2/SERPINE1/ATF3/THBS1 | 7 |
| BP | GO:0034333 | adherens junction assembly | 7/473 | 83/17653 | 0.0068366 | 0.0438423 | 0.0319939 | S100A10/SORBS1/SFRP1/PHLDB2/COL16A1/THBS1/THY1 | 7 |
| BP | GO:0044070 | regulation of anion transport | 7/473 | 83/17653 | 0.0068366 | 0.0438423 | 0.0319939 | PRKCD/OSR1/ATP1A2/SNCA/SFRP4/THBS1/APOE | 7 |
| BP | GO:0042743 | hydrogen peroxide metabolic process | 5/473 | 45/17653 | 0.0068377 | 0.0438423 | 0.0319939 | STAT3/CYBA/CYBB/RAC2/SNCA | 5 |
| BP | GO:0071827 | plasma lipoprotein particle organization | 5/473 | 45/17653 | 0.0068377 | 0.0438423 | 0.0319939 | PLTP/PLA2G2A/APOA1/LIPG/APOE | 5 |
| BP | GO:0030852 | regulation of granulocyte differentiation | 3/473 | 15/17653 | 0.0068406 | 0.0438423 | 0.0319939 | HCLS1/C1QC/TRIB1 | 3 |
| BP | GO:0036376 | sodium ion export across plasma membrane | 3/473 | 15/17653 | 0.0068406 | 0.0438423 | 0.0319939 | ATP1A2/ATP1A1/ATP1B3 | 3 |
| BP | GO:0060397 | JAK-STAT cascade involved in growth hormone signaling pathway | 3/473 | 15/17653 | 0.0068406 | 0.0438423 | 0.0319939 | JAK2/STAT3/PTPN1 | 3 |
| BP | GO:0070571 | negative regulation of neuron projection regeneration | 3/473 | 15/17653 | 0.0068406 | 0.0438423 | 0.0319939 | RGMA/SPP1/THY1 | 3 |
| BP | GO:0071850 | mitotic cell cycle arrest | 3/473 | 15/17653 | 0.0068406 | 0.0438423 | 0.0319939 | DUSP1/GADD45A/CDKN1A | 3 |
| BP | GO:0072075 | metanephric mesenchyme development | 3/473 | 15/17653 | 0.0068406 | 0.0438423 | 0.0319939 | MYC/OSR1/PDGFRB | 3 |
| BP | GO:0098856 | intestinal lipid absorption | 3/473 | 15/17653 | 0.0068406 | 0.0438423 | 0.0319939 | CEL/LDLR/APOA1 | 3 |
| BP | GO:1990000 | amyloid fibril formation | 3/473 | 15/17653 | 0.0068406 | 0.0438423 | 0.0319939 | LDLR/APP/APOE | 3 |
| BP | GO:0034620 | cellular response to unfolded protein | 10/473 | 149/17653 | 0.0069154 | 0.0442587 | 0.0322978 | TSPYL2/DDIT3/CCND1/SEC61A1/EXTL1/HSPA2/CCL2/HSPA6/ATF3/PTPN1 | 10 |
| BP | GO:0042554 | superoxide anion generation | 4/473 | 29/17653 | 0.0071072 | 0.0454215 | 0.0331463 | PRKCD/ITGB2/CYBA/CYBB | 4 |
| BP | GO:1902930 | regulation of alcohol biosynthetic process | 6/473 | 64/17653 | 0.0072398 | 0.0460453 | 0.0336015 | PLEK/TM7SF2/SNCA/FASN/SCD/APOE | 6 |
| BP | GO:1903036 | positive regulation of response to wounding | 6/473 | 64/17653 | 0.0072398 | 0.0460453 | 0.0336015 | S100A9/PLEK/SERPINE1/SOX15/HBEGF/THBS1 | 6 |
| BP | GO:0002698 | negative regulation of immune effector process | 8/473 | 105/17653 | 0.0072461 | 0.0460453 | 0.0336015 | BCL6/HAVCR2/IRAK3/HTRA1/CD59/APOA1/BST2/IFIT1 | 8 |
| BP | GO:0046620 | regulation of organ growth | 8/473 | 105/17653 | 0.0072461 | 0.0460453 | 0.0336015 | MYH6/PIM1/TGFBR3/GJA1/WWC1/PROX1/HAMP/HEY2 | 8 |
| BP | GO:0017038 | protein import | 12/473 | 198/17653 | 0.0072558 | 0.0460453 | 0.0336015 | PRKCD/JAK2/STAT3/BCL6/NFKBIA/PRICKLE1/TOMM40/BCL3/NUP62CL/MDFIC/CDKN1A/APOE | 12 |
| BP | GO:0036293 | response to decreased oxygen levels | 18/473 | 354/17653 | 0.00734 | 0.0464684 | 0.0339103 | MYC/CYBA/DDIT4/SFRP1/EDNRA/ENO1/CYBB/PLAU/LDHA/APOLD1/KCNA5/TGFBR3/RYR2/THBS1/PENK/POSTN/EGR1/NPPA | 18 |
| BP | GO:0071466 | cellular response to xenobiotic stimulus | 11/473 | 174/17653 | 0.0073431 | 0.0464684 | 0.0339103 | MGST1/CYP2J2/DDIT4/ERRFI1/FBXO32/AIF1/S100A12/RYR2/CMBL/CYP1B1/EGR1 | 11 |
| BP | GO:0000060 | protein import into nucleus, translocation | 5/473 | 46/17653 | 0.0075065 | 0.0473699 | 0.0345682 | JAK2/BCL6/NFKBIA/BCL3/CDKN1A | 5 |
| BP | GO:0030857 | negative regulation of epithelial cell differentiation | 5/473 | 46/17653 | 0.0075065 | 0.0473699 | 0.0345682 | S1PR3/FRZB/OSR1/CCND1/ID1 | 5 |
| BP | GO:0032147 | activation of protein kinase activity | 17/473 | 328/17653 | 0.0075355 | 0.0474512 | 0.0346275 | MAP3K6/PRKCD/FPR1/JAK2/MAP3K8/PLCE1/GRM1/ADCY3/MDFIC/SOCS1/GADD45G/MAP2K1/GADD45A/PTPN1/GADD45B/THBS1/DUSP5 | 17 |
| BP | GO:0007188 | adenylate cyclase-modulating G-protein coupled receptor signaling pathway | 12/473 | 199/17653 | 0.0075404 | 0.0474512 | 0.0346275 | S1PR3/FPR1/GNA15/MARCO/EDNRA/GRM1/ADCY3/RGS1/PDE4D/RAMP1/NPR3/RGS2 | 12 |
| BP | GO:0032874 | positive regulation of stress-activated MAPK cascade | 10/473 | 151/17653 | 0.0075719 | 0.0475825 | 0.0347233 | MAP3K6/FGD2/LTBR/MDFIC/TPD52L1/GADD45G/GADD45A/APP/PTPN1/GADD45B | 10 |
| BP | GO:0002699 | positive regulation of immune effector process | 11/473 | 175/17653 | 0.007653 | 0.0480258 | 0.0350468 | FCER1G/FES/ITGB2/VAMP8/FGR/RAC2/PVR/IL6/TFRC/C6/NPPA | 11 |
| BP | GO:0045778 | positive regulation of ossification | 7/473 | 85/17653 | 0.0077672 | 0.0486747 | 0.0355204 | CEBPB/OSR1/CLIC1/GJA1/IFITM1/IL6/CYR61 | 7 |
| BP | GO:0033619 | membrane protein proteolysis | 6/473 | 65/17653 | 0.0078004 | 0.0488144 | 0.0356223 | PRKCQ/TIMP4/SPPL2A/TIMP1/PTPN3/APOE | 6 |
| BP | GO:0007254 | JNK cascade | 12/473 | 200/17653 | 0.0078338 | 0.0489557 | 0.0357254 | MAP3K6/FGD2/SFRP1/LTBR/MDFIC/TPD52L1/GADD45G/TRIB1/GADD45A/APP/PTPN1/GADD45B | 12 |
| BP | GO:0070304 | positive regulation of stress-activated protein kinase signaling cascade | 10/473 | 152/17653 | 0.0079176 | 0.0494111 | 0.0360577 | MAP3K6/FGD2/LTBR/MDFIC/TPD52L1/GADD45G/GADD45A/APP/PTPN1/GADD45B | 10 |
| BP | GO:0050890 | cognition | 15/473 | 277/17653 | 0.007993 | 0.0498129 | 0.036351 | BTG2/CEBPB/CCND2/ATP1A2/GATM/PLK2/ADCY3/ITPR3/NPTX2/LDLR/ITGA5/PICALM/FOS/APP/APOE | 15 |
| BP | GO:0010880 | regulation of release of sequestered calcium ion into cytosol by sarcoplasmic reticulum | 4/473 | 30/17653 | 0.0080302 | 0.0498382 | 0.0363694 | ATP1A2/PDE4D/RYR2/CASQ1 | 4 |
| BP | GO:0042572 | retinol metabolic process | 4/473 | 30/17653 | 0.0080302 | 0.0498382 | 0.0363694 | RDH10/RDH5/ALDH1A3/CYP1B1 | 4 |
| BP | GO:0043552 | positive regulation of phosphatidylinositol 3-kinase activity | 4/473 | 30/17653 | 0.0080302 | 0.0498382 | 0.0363694 | FGR/PDGFRB/PDGFRA/TNFAIP8L3 | 4 |
| CC | GO:0031012 | extracellular matrix | 45/493 | 479/18698 | 1.45E-13 | 6.81E-11 | 5.36E-11 | S100A9/SMOC2/CD248/SFRP1/COL21A1/COL6A2/HTRA1/COL16A1/LUM/VWC2/ECM2/SERPINE1/EMILIN1/THBS4/OMD/LMCD1/ADAMTS9/SPOCK1/TIMP4/TGFBR3/FMOD/MATN2/SLITRK4/GLDN/APOA1/OLFML2A/ALPL/SERPINB1/COL6A3/ANXA2/CRISPLD2/MFAP4/IL1RL1/CTSG/TIMP1/AZGP1/CYR61/TNC/LAD1/COMP/AEBP1/THBS1/POSTN/APOE/NPPA | 45 |
| CC | GO:0060205 | cytoplasmic vesicle lumen | 32/493 | 338/18698 | 4.48E-10 | 6.09E-08 | 4.79E-08 | SERPINA3/S100A9/PRKCD/S100A8/PROS1/ALOX5/CTSC/F13A1/DOCK2/SRGN/MVP/TUBB/FGR/GSDMD/SERPINE1/RNASE2/FCN1/CFD/S100A11/S100A12/CAP1/APOA1/HSPA6/SERPINB1/ANXA2/CRISPLD2/CTSG/TIMP1/APP/LYZ/THBS1/APOE | 32 |
| CC | GO:0031983 | vesicle lumen | 32/493 | 339/18698 | 4.83E-10 | 6.09E-08 | 4.79E-08 | SERPINA3/S100A9/PRKCD/S100A8/PROS1/ALOX5/CTSC/F13A1/DOCK2/SRGN/MVP/TUBB/FGR/GSDMD/SERPINE1/RNASE2/FCN1/CFD/S100A11/S100A12/CAP1/APOA1/HSPA6/SERPINB1/ANXA2/CRISPLD2/CTSG/TIMP1/APP/LYZ/THBS1/APOE | 32 |
| CC | GO:0034774 | secretory granule lumen | 31/493 | 321/18698 | 5.18E-10 | 6.09E-08 | 4.79E-08 | SERPINA3/S100A9/PRKCD/S100A8/PROS1/ALOX5/CTSC/F13A1/DOCK2/SRGN/MVP/TUBB/FGR/GSDMD/SERPINE1/RNASE2/FCN1/CFD/S100A11/S100A12/CAP1/APOA1/HSPA6/SERPINB1/ANXA2/CRISPLD2/CTSG/TIMP1/APP/LYZ/THBS1 | 31 |
| CC | GO:0005578 | proteinaceous extracellular matrix | 33/493 | 375/18698 | 1.56E-09 | 1.47E-07 | 1.15E-07 | SMOC2/CD248/SFRP1/COL21A1/COL6A2/COL16A1/LUM/VWC2/ECM2/EMILIN1/THBS4/OMD/ADAMTS9/SPOCK1/TIMP4/TGFBR3/FMOD/MATN2/SLITRK4/GLDN/OLFML2A/ALPL/COL6A3/ANXA2/CRISPLD2/MFAP4/IL1RL1/TIMP1/CYR61/TNC/LAD1/COMP/POSTN | 33 |
| CC | GO:0015629 | actin cytoskeleton | 35/493 | 487/18698 | 8.88E-08 | 6.96E-06 | 5.47E-06 | CDC42EP4/MYH6/CORO1A/SORBS2/ARPC1B/HCK/WAS/LCP1/LMOD2/CYBA/IVNS1ABP/SORBS1/SVIL/FGR/SLC2A1/ARPC3/ZYX/SYNPO2L/STK38L/ACTB/AIF1/TNNT1/RAC2/ARPC5L/SNCA/MYH7B/CAP1/MYOZ3/MYOZ2/TPM3/CORIN/LAD1/MYL7/XIRP1/MYOT | 35 |
| CC | GO:0005925 | focal adhesion | 30/493 | 393/18698 | 2.10E-07 | 1.41E-05 | 1.11E-05 | JAK2/FES/SORBS2/ARPC1B/HCK/LCP1/CYBA/CAPN1/SORBS1/SVIL/PHLDB2/ARPC3/ZYX/FZD2/PLAU/CD59/ACTB/RAC2/PDGFRB/ARPC5L/MAP2K1/PVR/CAP1/GJA1/ITGA5/CD44/TNC/XIRP1/THY1/CNN1 | 30 |
| CC | GO:0005924 | cell-substrate adherens junction | 30/493 | 396/18698 | 2.47E-07 | 1.45E-05 | 1.14E-05 | JAK2/FES/SORBS2/ARPC1B/HCK/LCP1/CYBA/CAPN1/SORBS1/SVIL/PHLDB2/ARPC3/ZYX/FZD2/PLAU/CD59/ACTB/RAC2/PDGFRB/ARPC5L/MAP2K1/PVR/CAP1/GJA1/ITGA5/CD44/TNC/XIRP1/THY1/CNN1 | 30 |
| CC | GO:0030055 | cell-substrate junction | 30/493 | 401/18698 | 3.23E-07 | 1.69E-05 | 1.33E-05 | JAK2/FES/SORBS2/ARPC1B/HCK/LCP1/CYBA/CAPN1/SORBS1/SVIL/PHLDB2/ARPC3/ZYX/FZD2/PLAU/CD59/ACTB/RAC2/PDGFRB/ARPC5L/MAP2K1/PVR/CAP1/GJA1/ITGA5/CD44/TNC/XIRP1/THY1/CNN1 | 30 |
| CC | GO:0005581 | collagen trimer | 13/493 | 88/18698 | 5.03E-07 | 2.36E-05 | 1.86E-05 | FCN3/C1QC/C1QB/MARCO/C1QTNF1/COL21A1/COL6A2/COL16A1/LUM/EMILIN1/FCN1/GLDN/COL6A3 | 13 |
| CC | GO:0043292 | contractile fiber | 21/493 | 230/18698 | 8.40E-07 | 3.59E-05 | 2.82E-05 | MYH6/SORBS2/LMOD2/SVIL/SLC2A1/ENO1/SYNPO2L/FBXO32/TNNT1/KCNA5/TIMP4/MYH7B/LRRC39/MYOZ3/GJA1/MYOZ2/TPM3/RYR2/CASQ1/MYL7/MYOT | 21 |
| CC | GO:0030666 | endocytic vesicle membrane | 17/493 | 161/18698 | 1.27E-06 | 4.99E-05 | 3.92E-05 | CD163/CORO1A/CYBA/VAMP8/MARCO/FZD2/CYBB/RAC2/SGIP1/TAP1/TYRP1/HLA-DRB4/LDLR/PICALM/SH3GL2/HBEGF/APOE | 17 |
| CC | GO:0030669 | clathrin-coated endocytic vesicle membrane | 9/493 | 43/18698 | 1.45E-06 | 5.12E-05 | 4.02E-05 | FZD2/SGIP1/TYRP1/HLA-DRB4/LDLR/PICALM/SH3GL2/HBEGF/APOE | 9 |
| CC | GO:0030016 | myofibril | 20/493 | 219/18698 | 1.53E-06 | 5.12E-05 | 4.02E-05 | MYH6/SORBS2/LMOD2/SVIL/SLC2A1/ENO1/SYNPO2L/FBXO32/TNNT1/KCNA5/TIMP4/MYH7B/LRRC39/MYOZ3/MYOZ2/TPM3/RYR2/CASQ1/MYL7/MYOT | 20 |
| CC | GO:0031091 | platelet alpha granule | 12/493 | 90/18698 | 4.19E-06 | 0.0001237 | 9.73E-05 | SERPINA3/PROS1/F13A1/SRGN/SERPINA5/SERPINE1/CFD/LHFPL2/SNCA/TIMP1/APP/THBS1 | 12 |
| CC | GO:0044449 | contractile fiber part | 19/493 | 214/18698 | 4.21E-06 | 0.0001237 | 9.73E-05 | MYH6/SORBS2/LMOD2/SVIL/SLC2A1/ENO1/SYNPO2L/FBXO32/TNNT1/KCNA5/TIMP4/LRRC39/MYOZ3/MYOZ2/TPM3/RYR2/CASQ1/MYL7/MYOT | 19 |
| CC | GO:0030017 | sarcomere | 18/493 | 197/18698 | 5.03E-06 | 0.0001391 | 0.0001094 | MYH6/SORBS2/LMOD2/SLC2A1/ENO1/SYNPO2L/FBXO32/TNNT1/KCNA5/TIMP4/LRRC39/MYOZ3/MYOZ2/TPM3/RYR2/CASQ1/MYL7/MYOT | 18 |
| CC | GO:0045121 | membrane raft | 23/493 | 302/18698 | 5.76E-06 | 0.000143 | 0.0001124 | JAK2/HCK/ITGB2/CD14/S100A10/ACE2/SORBS1/TUBB/TUBA1B/SLC2A1/ATP1A2/TNFRSF1A/SERPINH1/ATP1A1/KCNA5/TUBA1A/GJA1/ATP1B3/BST2/ANXA2/APP/SULF1/THY1 | 23 |
| CC | GO:0030667 | secretory granule membrane | 23/493 | 303/18698 | 6.08E-06 | 0.000143 | 0.0001124 | FPR1/FCER1G/ITGB2/MGST1/CYBA/CD14/CD68/VAMP8/FCGR2A/SERPINA5/TYROBP/DDOST/CYBB/TMBIM1/PLAU/CD59/LHFPL2/ITPR3/SNCA/BST2/SLC2A3/CD44/RAB15 | 23 |
| CC | GO:0098857 | membrane microdomain | 23/493 | 303/18698 | 6.08E-06 | 0.000143 | 0.0001124 | JAK2/HCK/ITGB2/CD14/S100A10/ACE2/SORBS1/TUBB/TUBA1B/SLC2A1/ATP1A2/TNFRSF1A/SERPINH1/ATP1A1/KCNA5/TUBA1A/GJA1/ATP1B3/BST2/ANXA2/APP/SULF1/THY1 | 23 |
| CC | GO:0098589 | membrane region | 23/493 | 314/18698 | 1.09E-05 | 0.000244 | 0.0001918 | JAK2/HCK/ITGB2/CD14/S100A10/ACE2/SORBS1/TUBB/TUBA1B/SLC2A1/ATP1A2/TNFRSF1A/SERPINH1/ATP1A1/KCNA5/TUBA1A/GJA1/ATP1B3/BST2/ANXA2/APP/SULF1/THY1 | 23 |
| CC | GO:0005775 | vacuolar lumen | 16/493 | 172/18698 | 1.34E-05 | 0.0002858 | 0.0002247 | SERPINA3/PRKCD/CTSC/IFI30/TUBB/LUM/ASAH1/RNASE2/OMD/PDGFRB/CAP1/FMOD/CTSK/ANXA2/CTSG/LYZ | 16 |
| CC | GO:0005766 | primary lysosome | 15/493 | 155/18698 | 1.55E-05 | 0.000303 | 0.0002382 | SERPINA3/PRKCD/FPR1/CTSC/MGST1/CD68/VAMP8/TUBB/DDOST/RNASE2/CAP1/BST2/ANXA2/CTSG/LYZ | 15 |
| CC | GO:0042582 | azurophil granule | 15/493 | 155/18698 | 1.55E-05 | 0.000303 | 0.0002382 | SERPINA3/PRKCD/FPR1/CTSC/MGST1/CD68/VAMP8/TUBB/DDOST/RNASE2/CAP1/BST2/ANXA2/CTSG/LYZ | 15 |
| CC | GO:0030139 | endocytic vesicle | 21/493 | 290/18698 | 3.10E-05 | 0.0005826 | 0.000458 | CDC42EP4/CD163/CORO1A/WAS/CYBA/VAMP8/MARCO/ZYX/FZD2/CYBB/RAC2/SGIP1/TAP1/TYRP1/HLA-DRB4/LDLR/APOA1/PICALM/SH3GL2/HBEGF/APOE | 21 |
| CC | GO:0045334 | clathrin-coated endocytic vesicle | 9/493 | 62/18698 | 3.36E-05 | 0.0005932 | 0.0004663 | FZD2/SGIP1/TYRP1/HLA-DRB4/LDLR/PICALM/SH3GL2/HBEGF/APOE | 9 |
| CC | GO:0042383 | sarcolemma | 13/493 | 128/18698 | 3.41E-05 | 0.0005932 | 0.0004663 | SLC2A1/ATP1A2/COL6A2/ATP1A1/SSPN/KCNJ2/SGCG/RYR2/COL6A3/CASQ1/ANXA2/SLC38A2/MYOT | 13 |
| CC | GO:0043235 | receptor complex | 24/493 | 374/18698 | 6.07E-05 | 0.0010193 | 0.0008013 | ITGB2/IL13RA1/CD14/SORBS1/OSMR/TNFRSF1A/GRM1/VWC2/EMILIN1/TSPAN32/TM7SF2/PDGFRB/ITPR3/LY96/PDGFRA/GABRB1/TGFBR3/RAMP1/LDLR/ITGA5/IL6/APP/CD44/TFRC | 24 |
| CC | GO:0031093 | platelet alpha granule lumen | 9/493 | 67/18698 | 6.29E-05 | 0.0010202 | 0.000802 | SERPINA3/PROS1/F13A1/SRGN/SERPINE1/CFD/TIMP1/APP/THBS1 | 9 |
| CC | GO:0030665 | clathrin-coated vesicle membrane | 11/493 | 104/18698 | 9.38E-05 | 0.0014698 | 0.0011554 | VAMP8/FZD2/SGIP1/TYRP1/HLA-DRB4/LDLR/PICALM/SH3GL2/HBEGF/TFRC/APOE | 11 |
| CC | GO:0072562 | blood microparticle | 15/493 | 183/18698 | 0.0001065 | 0.0016139 | 0.0012687 | SERPINA3/FCN3/C1R/PROS1/F13A1/C1QC/C1QB/SLC2A1/ACTB/HSPA2/CLIC1/APOA1/HSPA6/TFRC/APOE | 15 |
| CC | GO:0005788 | endoplasmic reticulum lumen | 20/493 | 297/18698 | 0.0001294 | 0.0019001 | 0.0014937 | CTSC/COL21A1/SERPINH1/COL6A2/COL16A1/RDH5/IGFBP4/SPP1/APOA1/COL6A3/CASQ1/IL6/TIMP1/APP/SDF2L1/CYR61/TNC/THBS1/PENK/APOE | 20 |
| CC | GO:0005614 | interstitial matrix | 4/493 | 12/18698 | 0.0001997 | 0.0028443 | 0.002236 | SMOC2/VWC2/ECM2/TNC | 4 |
| CC | GO:0098562 | cytoplasmic side of membrane | 15/493 | 199/18698 | 0.0002678 | 0.0037023 | 0.0029104 | SLA/JAK2/FES/HCK/GNA15/FGR/ERRFI1/ATP2A2/RGS1/NPTX2/SPPL2A/RGS2/LITAF/PTPN1/PTPN3 | 15 |
| CC | GO:0044420 | extracellular matrix component | 11/493 | 119/18698 | 0.0003114 | 0.0041488 | 0.0032614 | SMOC2/LUM/VWC2/EMILIN1/THBS4/MATN2/ANXA2/MFAP4/TIMP1/TNC/LAD1 | 11 |
| CC | GO:0014704 | intercalated disc | 7/493 | 50/18698 | 0.0003178 | 0.0041488 | 0.0032614 | SLC2A1/ATP1A2/ATP1A1/KCNA5/KCNJ2/GJA1/HAMP | 7 |
| CC | GO:0005884 | actin filament | 10/493 | 101/18698 | 0.0003335 | 0.0042362 | 0.0033301 | CORO1A/HCK/WAS/LCP1/LMOD2/ARPC3/AIF1/RAC2/TPM3/XIRP1 | 10 |
| CC | GO:0043209 | myelin sheath | 13/493 | 164/18698 | 0.0004181 | 0.0051716 | 0.0040654 | MBP/TKT/TUBA1B/ATP1A2/ATP1A1/ACTB/HSPA2/ITPR3/TUBA1A/ANXA2/NME1/UCHL1/THY1 | 13 |
| CC | GO:0030018 | Z disc | 11/493 | 125/18698 | 0.0004757 | 0.0057327 | 0.0045066 | MYH6/SORBS2/SLC2A1/SYNPO2L/FBXO32/KCNA5/MYOZ3/MYOZ2/RYR2/CASQ1/MYOT | 11 |
| CC | GO:0001725 | stress fiber | 7/493 | 54/18698 | 0.0005146 | 0.0058996 | 0.0046378 | MYH6/LCP1/CYBA/SORBS1/ZYX/TPM3/XIRP1 | 7 |
| CC | GO:0097517 | contractile actin filament bundle | 7/493 | 54/18698 | 0.0005146 | 0.0058996 | 0.0046378 | MYH6/LCP1/CYBA/SORBS1/ZYX/TPM3/XIRP1 | 7 |
| CC | GO:0016528 | sarcoplasm | 8/493 | 71/18698 | 0.0005472 | 0.0061233 | 0.0048136 | RASD1/THBS4/ATP2A2/ITPR3/SPOCK1/RYR2/CASQ1/THBS1 | 8 |
| CC | GO:0005769 | early endosome | 20/493 | 334/18698 | 0.0005958 | 0.0065118 | 0.005119 | CORO1A/FGD2/VAMP8/HAVCR2/HGS/TRAK2/MAP2K1/ST8SIA2/LDLR/APOA1/GJA1/PICALM/SH3GL2/ANXA2/LITAF/LIPG/PTPN1/RABGAP1L/TFRC/APOE | 20 |
| CC | GO:0070821 | tertiary granule membrane | 8/493 | 73/18698 | 0.0006604 | 0.0069047 | 0.0054279 | FCER1G/ITGB2/CYBA/VAMP8/CYBB/PLAU/CD59/SLC2A3 | 8 |
| CC | GO:0035578 | azurophil granule lumen | 9/493 | 91/18698 | 0.0006611 | 0.0069047 | 0.0054279 | SERPINA3/PRKCD/CTSC/TUBB/RNASE2/CAP1/ANXA2/CTSG/LYZ | 9 |
| CC | GO:0009898 | cytoplasmic side of plasma membrane | 13/493 | 176/18698 | 0.0008151 | 0.0083285 | 0.0065472 | SLA/JAK2/FES/HCK/GNA15/FGR/ERRFI1/ATP2A2/RGS1/NPTX2/RGS2/LITAF/PTPN3 | 13 |
| CC | GO:0032432 | actin filament bundle | 7/493 | 60/18698 | 0.00098 | 0.0098001 | 0.007704 | MYH6/LCP1/CYBA/SORBS1/ZYX/TPM3/XIRP1 | 7 |
| CC | GO:0031674 | I band | 11/493 | 138/18698 | 0.0010878 | 0.0106514 | 0.0083732 | MYH6/SORBS2/SLC2A1/SYNPO2L/FBXO32/KCNA5/MYOZ3/MYOZ2/RYR2/CASQ1/MYOT | 11 |
| CC | GO:0016529 | sarcoplasmic reticulum | 7/493 | 62/18698 | 0.0011929 | 0.011442 | 0.0089948 | RASD1/THBS4/ATP2A2/ITPR3/RYR2/CASQ1/THBS1 | 7 |
| CC | GO:0101002 | ficolin-1-rich granule | 13/493 | 185/18698 | 0.0012885 | 0.0121119 | 0.0095214 | FPR1/FCER1G/ALOX5/ITGB2/CAPN1/MVP/GSDMD/ASAH1/FCN1/CFD/HSPA6/CRISPLD2/SLC2A3 | 13 |
| CC | GO:0070820 | tertiary granule | 12/493 | 164/18698 | 0.0013858 | 0.0127715 | 0.0100398 | FPR1/FCER1G/ITGB2/CYBA/VAMP8/GSDMD/CYBB/ASAH1/PLAU/CD59/LYZ/SLC2A3 | 12 |
| CC | GO:0005911 | cell-cell junction | 23/493 | 440/18698 | 0.0015231 | 0.0137662 | 0.0108218 | CDC42EP4/PRKCD/CORO1A/WAS/HEG1/SORBS1/CCND1/SLC2A1/ATP1A2/ATP1A1/ZYX/IGSF5/S100A11/KCNA5/PVR/KCNJ2/GJA1/ITGA5/STXBP6/ANXA2/APP/HAMP/KLHL24 | 23 |
| CC | GO:0042641 | actomyosin | 7/493 | 66/18698 | 0.0017262 | 0.0153081 | 0.0120339 | MYH6/LCP1/CYBA/SORBS1/ZYX/TPM3/XIRP1 | 7 |
| CC | GO:0045177 | apical part of cell | 20/493 | 366/18698 | 0.0018068 | 0.0157004 | 0.0123423 | SORBS2/MGST1/CYBA/OSMR/SLC2A1/ATP1A1/IGSF5/KCNA5/PDGFRB/SLC7A5/ITPR3/PROM1/PDE4D/LDLR/GJA1/BST2/APP/HAMP/CD44/THY1 | 20 |
| CC | GO:0005770 | late endosome | 15/493 | 240/18698 | 0.0018373 | 0.0157004 | 0.0123423 | IFITM3/VAMP8/DDIT3/HGS/FYCO1/MAP2K1/SPPL2A/HLA-DRB4/LDLR/GJA1/ANKRD13A/BST2/ANXA2/SLC30A2/LITAF | 15 |
| CC | GO:0019897 | extrinsic component of plasma membrane | 12/493 | 171/18698 | 0.0019748 | 0.0162838 | 0.012801 | SLA/JAK2/FES/HCK/S100A10/GNA15/FGR/ERRFI1/FCN1/ATP2A2/RGS1/ANXA2 | 12 |
| CC | GO:0030662 | coated vesicle membrane | 12/493 | 171/18698 | 0.0019748 | 0.0162838 | 0.012801 | VAMP8/FZD2/CD59/SGIP1/TYRP1/HLA-DRB4/LDLR/PICALM/SH3GL2/HBEGF/TFRC/APOE | 12 |
| CC | GO:0044291 | cell-cell contact zone | 7/493 | 68/18698 | 0.0020543 | 0.0166467 | 0.0130862 | SLC2A1/ATP1A2/ATP1A1/KCNA5/KCNJ2/GJA1/HAMP | 7 |
| CC | GO:0031234 | extrinsic component of cytoplasmic side of plasma membrane | 9/493 | 110/18698 | 0.0025269 | 0.0192723 | 0.0151502 | SLA/JAK2/FES/HCK/GNA15/FGR/ERRFI1/ATP2A2/RGS1 | 9 |
| CC | GO:0005885 | Arp2/3 protein complex | 3/493 | 11/18698 | 0.0025669 | 0.0192723 | 0.0151502 | ARPC1B/ARPC3/ARPC5L | 3 |
| CC | GO:0005890 | sodium:potassium-exchanging ATPase complex | 3/493 | 11/18698 | 0.0025669 | 0.0192723 | 0.0151502 | ATP1A2/ATP1A1/ATP1B3 | 3 |
| CC | GO:0031094 | platelet dense tubular network | 3/493 | 11/18698 | 0.0025669 | 0.0192723 | 0.0151502 | SERPINA5/ATP2A2/ITPR3 | 3 |
| CC | GO:0032587 | ruffle membrane | 8/493 | 90/18698 | 0.0025833 | 0.0192723 | 0.0151502 | LCP1/FGD2/PLEK/FGR/AIF1/WWC1/ITGA5/NME1 | 8 |
| CC | GO:0035579 | specific granule membrane | 8/493 | 91/18698 | 0.0027686 | 0.0203318 | 0.0159831 | ITGB2/CYBA/VAMP8/CYBB/TMBIM1/PLAU/CD59/SLC2A3 | 8 |
| CC | GO:0030136 | clathrin-coated vesicle | 12/493 | 179/18698 | 0.0028844 | 0.0205405 | 0.0161472 | VAMP8/FZD2/SGIP1/TMED9/TYRP1/HLA-DRB4/LDLR/PICALM/SH3GL2/HBEGF/TFRC/APOE | 12 |
| CC | GO:0098802 | plasma membrane receptor complex | 12/493 | 179/18698 | 0.0028844 | 0.0205405 | 0.0161472 | ITGB2/IL13RA1/SORBS1/OSMR/GRM1/VWC2/EMILIN1/TSPAN32/RAMP1/ITGA5/IL6/TFRC | 12 |
| CC | GO:0005604 | basement membrane | 8/493 | 92/18698 | 0.0029641 | 0.0207927 | 0.0163454 | SMOC2/VWC2/THBS4/MATN2/ANXA2/TIMP1/TNC/LAD1 | 8 |
| CC | GO:0042581 | specific granule | 11/493 | 160/18698 | 0.0035067 | 0.0242375 | 0.0190535 | ITGB2/CYBA/DOCK2/VAMP8/GSDMD/CYBB/TMBIM1/PLAU/CD59/LYZ/SLC2A3 | 11 |
| CC | GO:0009897 | external side of plasma membrane | 16/493 | 287/18698 | 0.004116 | 0.0277763 | 0.0218354 | CD163/FCER1G/ITGB2/HEG1/CD14/CD209/SERPINA5/CD59/FCN1/TGFBR3/LDLR/ITGA5/IL1RL1/THBS1/TFRC/THY1 | 16 |
| CC | GO:0035577 | azurophil granule membrane | 6/493 | 58/18698 | 0.0041369 | 0.0277763 | 0.0218354 | FPR1/MGST1/CD68/VAMP8/DDOST/BST2 | 6 |
| CC | GO:0044853 | plasma membrane raft | 8/493 | 98/18698 | 0.0043727 | 0.028946 | 0.0227548 | JAK2/HCK/ITGB2/SLC2A1/ATP1A2/ATP1A1/KCNA5/ATP1B3 | 8 |
| CC | GO:0005901 | caveola | 7/493 | 78/18698 | 0.004473 | 0.029199 | 0.0229537 | JAK2/HCK/SLC2A1/ATP1A2/ATP1A1/KCNA5/ATP1B3 | 7 |
| CC | GO:0005635 | nuclear envelope | 22/493 | 455/18698 | 0.0048646 | 0.0313202 | 0.0246212 | ALOX5/RANGAP1/ALOX5AP/CCND2/MVP/PRICKLE1/TUBB/CYBB/NUP62CL/RAC2/TM7SF2/CLIC1/XPO4/ITPR3/SNCA/GABRB1/SLC16A3/PDE4D/DPY19L2/PLA2G4C/SCRN1/APP | 22 |
| CC | GO:0034362 | low-density lipoprotein particle | 3/493 | 14/18698 | 0.005339 | 0.0339101 | 0.0266572 | LDLR/APOA1/APOE | 3 |
| CC | GO:0045335 | phagocytic vesicle | 9/493 | 124/18698 | 0.0056004 | 0.0346337 | 0.0272261 | CDC42EP4/CORO1A/WAS/CYBA/VAMP8/ZYX/CYBB/RAC2/TAP1 | 9 |
| CC | GO:1904813 | ficolin-1-rich granule lumen | 9/493 | 124/18698 | 0.0056004 | 0.0346337 | 0.0272261 | ALOX5/CAPN1/MVP/GSDMD/ASAH1/FCN1/CFD/HSPA6/CRISPLD2 | 9 |
| CC | GO:0005765 | lysosomal membrane | 18/493 | 355/18698 | 0.0064674 | 0.0389358 | 0.030608 | FPR1/IFITM3/MGST1/SLC15A3/LAPTM5/CD68/VAMP8/DDOST/PLEKHF1/TMBIM1/TMEM74/SPPL2A/HLA-DRB4/LDLR/BST2/ANXA2/SLC30A2/LITAF | 18 |
| CC | GO:0098852 | lytic vacuole membrane | 18/493 | 355/18698 | 0.0064674 | 0.0389358 | 0.030608 | FPR1/IFITM3/MGST1/SLC15A3/LAPTM5/CD68/VAMP8/DDOST/PLEKHF1/TMBIM1/TMEM74/SPPL2A/HLA-DRB4/LDLR/BST2/ANXA2/SLC30A2/LITAF | 18 |
| CC | GO:0090533 | cation-transporting ATPase complex | 3/493 | 15/18698 | 0.0065445 | 0.0389358 | 0.030608 | ATP1A2/ATP1A1/ATP1B3 | 3 |
| CC | GO:0030135 | coated vesicle | 15/493 | 276/18698 | 0.0067491 | 0.0396509 | 0.0311702 | CTSC/VAMP8/FZD2/CD59/SGIP1/TMED9/TYRP1/HLA-DRB4/LDLR/PICALM/SH3GL2/HBEGF/APP/TFRC/APOE | 15 |
| CC | GO:0042470 | melanosome | 8/493 | 106/18698 | 0.0069909 | 0.0400697 | 0.0314993 | SLC2A1/ATP1A1/GPNMB/FASN/TYRP1/ATP1B3/ANXA2/TFRC | 8 |
| CC | GO:0048770 | pigment granule | 8/493 | 106/18698 | 0.0069909 | 0.0400697 | 0.0314993 | SLC2A1/ATP1A1/GPNMB/FASN/TYRP1/ATP1B3/ANXA2/TFRC | 8 |
| CC | GO:0005913 | cell-cell adherens junction | 8/493 | 110/18698 | 0.0086765 | 0.0491319 | 0.0386232 | CDC42EP4/SORBS1/ZYX/S100A11/PVR/GJA1/STXBP6/ANXA2 | 8 |
| MF | GO:0050786 | RAGE receptor binding | 6/473 | 11/17548 | 1.53E-07 | 0.0001032 | 9.21E-05 | S100A9/FPR1/S100A8/HMGB2/S100A13/S100A12 | 6 |
| MF | GO:0008201 | heparin binding | 18/473 | 158/17548 | 2.72E-07 | 0.0001032 | 9.21E-05 | SMOC2/SERPINA5/SFRP1/ECM2/CEL/THBS4/GPNMB/TGFBR3/CRISPLD2/CTSG/HBEGF/APP/LIPG/CYR61/COMP/THBS1/POSTN/APOE | 18 |
| MF | GO:0005539 | glycosaminoglycan binding | 20/473 | 215/17548 | 1.58E-06 | 0.0004003 | 0.0003575 | LYVE1/SMOC2/SERPINA5/SFRP1/ECM2/CEL/THBS4/GPNMB/TGFBR3/CRISPLD2/CTSG/HBEGF/APP/LIPG/CD44/CYR61/COMP/THBS1/POSTN/APOE | 20 |
| MF | GO:0005200 | structural constituent of cytoskeleton | 13/473 | 107/17548 | 6.07E-06 | 0.0009381 | 0.0008379 | TUBA3E/TUBA3C/TUBA3D/SORBS2/ARPC1B/TUBB/TUBA1B/TUBA1C/FRMD5/ARPC3/ACTB/FRMD3/TUBA1A | 13 |
| MF | GO:1901681 | sulfur compound binding | 20/473 | 235/17548 | 6.18E-06 | 0.0009381 | 0.0008379 | SMOC2/MGST1/SERPINA5/SFRP1/ECM2/CEL/THBS4/GPNMB/TGFBR3/RYR2/CRISPLD2/CTSG/HBEGF/APP/LIPG/CYR61/COMP/THBS1/POSTN/APOE | 20 |
| MF | GO:0005178 | integrin binding | 12/473 | 118/17548 | 8.23E-05 | 0.0104046 | 0.0092928 | S1PR3/LCP1/FRMD5/COL16A1/ECM2/EMILIN1/THBS4/GPNMB/ITGA5/CYR61/THBS1/THY1 | 12 |
| MF | GO:0043548 | phosphatidylinositol 3-kinase binding | 6/473 | 29/17548 | 0.0001041 | 0.0112925 | 0.0100858 | PIK3IP1/JAK2/CORO1A/ATP1A1/HCST/PDGFRB | 6 |
| MF | GO:0050839 | cell adhesion molecule binding | 28/473 | 489/17548 | 0.0001584 | 0.0150285 | 0.0134226 | S1PR3/ITGB2/RANGAP1/LCP1/PHLDB2/FRMD5/ENO1/COL16A1/ECM2/LDHA/EMILIN1/THBS4/GPNMB/S100A11/CLIC1/PROM1/PVR/FASN/ITGA5/PICALM/STXBP6/ANXA2/PTPN1/CYR61/LAD1/THBS1/THY1/POSTN | 28 |
| MF | GO:0005518 | collagen binding | 8/473 | 63/17548 | 0.0002771 | 0.0233662 | 0.0208693 | C1QTNF1/SERPINH1/LUM/ECM2/CTSK/CD44/COMP/THBS1 | 8 |
| MF | GO:0008191 | metalloendopeptidase inhibitor activity | 4/473 | 15/17548 | 0.0005617 | 0.0426301 | 0.0380747 | SPOCK1/TIMP4/BST2/TIMP1 | 4 |
| MF | GO:0003779 | actin binding | 23/473 | 410/17548 | 0.0007937 | 0.045372 | 0.0405237 | MYH6/CORO1A/ARPC1B/WAS/LCP1/HCLS1/LMOD2/SORBS1/SVIL/ARPC3/SYNPO2L/STK38L/AIF1/ARPC5L/MYH7B/CAP1/KLHL3/MYOZ3/MYOZ2/TPM3/XIRP1/CNN1/MYOT | 23 |
| MF | GO:0005543 | phospholipid binding | 23/473 | 410/17548 | 0.0007937 | 0.045372 | 0.0405237 | FES/FGD2/PLEK/ADAP2/PLTP/SERPINA5/PLEKHF1/GSDMD/NUP62CL/PLA2G2A/SGIP1/ITPR3/SNCA/KCNJ2/PLA2G4C/APOA1/TNFAIP8L3/PICALM/STXBP6/ANXA2/THBS1/THY1/APOE | 23 |
| MF | GO:0004866 | endopeptidase inhibitor activity | 13/473 | 173/17548 | 0.0008466 | 0.045372 | 0.0405237 | SERPINA3/PROS1/SERPINA5/SERPINH1/SERPINE1/SPOCK1/TIMP4/SNCA/SERPINB1/COL6A3/BST2/TIMP1/APP | 13 |
| MF | GO:0008329 | signaling pattern recognition receptor activity | 4/473 | 17/17548 | 0.0009383 | 0.045372 | 0.0405237 | CD14/MARCO/FCN1/LY96 | 4 |
| MF | GO:0038187 | pattern recognition receptor activity | 4/473 | 17/17548 | 0.0009383 | 0.045372 | 0.0405237 | CD14/MARCO/FCN1/LY96 | 4 |
| MF | GO:0019838 | growth factor binding | 11/473 | 133/17548 | 0.0009565 | 0.045372 | 0.0405237 | NKD2/OSMR/DUSP1/HTRA1/S100A13/PDGFRB/PDGFRA/TGFBR3/IGFBP4/CYR61/THBS1 | 11 |

KEGG pathways

| ID | Description | GeneRatio | BgRatio | pvalue | p.adjust | qvalue | geneID | Count |
| --- | --- | --- | --- | --- | --- | --- | --- | --- |
| hsa04145 | Phagosome | 26/274 | 152/8063 | 5.98E-12 | 1.66E-09 | 1.13E-09 | 112714/7278/113457/715/11151/3689/1535/929/30835/203068/10376/2212/8685/84790/29927/1536/9146/60/7060/7846/6890/3126/3678/1311/7057/7037 | 26 |
| hsa04210 | Apoptosis | 19/274 | 136/8063 | 1.41E-07 | 1.96E-05 | 1.33E-05 | 112714/7278/113457/1075/4792/823/1649/10376/84790/7132/60/3710/10912/7846/5604/1647/2353/1513/4616 | 19 |
| hsa04610 | Complement and coagulation cascades | 14/274 | 85/8063 | 8.57E-07 | 7.94E-05 | 5.41E-05 | 715/5627/3689/2162/714/713/5648/11326/5104/5054/5328/966/1675/729 | 14 |
| hsa04540 | Gap junction | 14/274 | 88/8063 | 1.32E-06 | 9.20E-05 | 6.27E-05 | 112714/7278/113457/203068/10376/84790/2911/109/5159/3710/5156/7846/5604/2697 | 14 |
| hsa04978 | Mineral absorption | 10/274 | 59/8063 | 2.51E-05 | 0.0012179 | 0.0008301 | 4499/4501/4489/4502/477/476/9843/4495/483/4493 | 10 |
| hsa05216 | Thyroid cancer | 8/274 | 37/8063 | 2.63E-05 | 0.0012179 | 0.0008301 | 4609/595/10912/5604/1647/7170/1026/4616 | 8 |
| hsa04066 | HIF-1 signaling pathway | 13/274 | 109/8063 | 7.65E-05 | 0.0030365 | 0.0020695 | 6774/4055/6513/2023/1536/5054/3939/5604/1026/3569/7076/7037/4878 | 13 |
| hsa04068 | FoxO signaling pathway | 14/274 | 131/8063 | 0.0001359 | 0.0044147 | 0.0030089 | 6774/604/894/595/2911/10769/114907/10912/5604/1647/10365/1026/3569/4616 | 14 |
| hsa05020 | Prion disease | 22/274 | 273/8063 | 0.0001429 | 0.0044147 | 0.0030089 | 112714/7278/113457/5580/714/1535/713/1649/203068/10376/84790/1536/56901/3306/5880/3710/7846/3310/6262/3569/1958/729 | 22 |
| hsa05130 | Pathogenic Escherichia coli infection | 17/274 | 193/8063 | 0.0002924 | 0.0081279 | 0.0055397 | 112714/7278/113457/10095/3059/4792/203068/10376/2212/84790/7132/10094/60/81873/7846/2353/3569 | 17 |
| hsa04668 | TNF signaling pathway | 12/274 | 112/8063 | 0.0003977 | 0.0100522 | 0.0068512 | 8809/4792/1051/1326/602/7132/6347/2920/5604/2353/3569/3726 | 12 |
| hsa04510 | Focal adhesion | 17/274 | 201/8063 | 0.0004706 | 0.0109012 | 0.0074298 | 894/595/1292/7791/60/7060/5880/5159/5156/5604/6696/3678/1293/3371/1311/58498/7057 | 17 |
| hsa04933 | AGE-RAGE signaling pathway in diabetic complications | 11/274 | 100/8063 | 0.0005516 | 0.0117964 | 0.00804 | 5580/3717/6774/5292/51196/595/1536/5054/6347/3569/1958 | 11 |
| hsa05218 | Melanoma | 9/274 | 72/8063 | 0.0006903 | 0.0137082 | 0.009343 | 595/5159/10912/5156/5604/1647/1026/4616/8817 | 9 |
| hsa04064 | NF-kappa B signaling pathway | 11/274 | 104/8063 | 0.0007706 | 0.0139641 | 0.0095174 | 4792/929/4055/7132/5328/5588/10912/23643/2920/1647/4616 | 11 |
| hsa04919 | Thyroid hormone signaling pathway | 12/274 | 121/8063 | 0.0008037 | 0.0139641 | 0.0095174 | 4624/4609/51196/595/6513/477/476/1734/60/488/5604/483 | 12 |
| hsa05132 | Salmonella infection | 17/274 | 213/8063 | 0.0009103 | 0.0148869 | 0.0101463 | 4609/10095/4792/929/6281/7132/10094/79792/60/79443/81873/23643/5604/2353/302/3569/58498 | 17 |
| hsa05133 | Pertussis | 9/274 | 76/8063 | 0.0010267 | 0.0154705 | 0.0105441 | 715/3689/714/713/929/23643/3678/2353/3569 | 9 |
| hsa04931 | Insulin resistance | 11/274 | 108/8063 | 0.0010573 | 0.0154705 | 0.0105441 | 5580/6774/4792/9945/6513/7132/5507/5588/1375/3569/5770 | 11 |
| hsa04666 | Fc gamma R-mediated phagocytosis | 10/274 | 93/8063 | 0.0011687 | 0.0158386 | 0.010795 | 5580/10095/3055/7454/2212/10094/5880/81873/5604/8605 | 10 |
| hsa04630 | JAK-STAT signaling pathway | 14/274 | 162/8063 | 0.0011964 | 0.0158386 | 0.010795 | 4609/3717/6774/5292/3597/894/9180/595/5159/8651/5156/1026/8835/3569 | 14 |
| hsa05145 | Toxoplasmosis | 11/274 | 112/8063 | 0.001427 | 0.018032 | 0.0122899 | 3717/240/6774/4792/7132/3306/8651/23643/3126/3949/3310 | 11 |
| hsa05205 | Proteoglycans in cancer | 16/274 | 205/8063 | 0.0016187 | 0.0195656 | 0.0133351 | 4609/6774/3059/51196/595/2535/4060/5328/60/3710/5604/3678/1026/1839/960/7057 | 16 |
| hsa05202 | Transcriptional misregulation in cancer | 15/274 | 192/8063 | 0.0022255 | 0.0221268 | 0.0150807 | 4609/64332/604/1051/929/894/1649/5328/10912/8148/8842/1647/1026/3569/4616 | 15 |
| hsa04972 | Pancreatic secretion | 10/274 | 102/8063 | 0.0023584 | 0.0221268 | 0.0150807 | 477/476/5320/109/1056/488/3710/483/6262/278 | 10 |
| hsa04010 | MAPK signaling pathway | 20/274 | 294/8063 | 0.0023724 | 0.0221268 | 0.0150807 | 9064/4609/929/1649/1326/1843/7132/3306/5880/5159/10912/5156/5604/8605/1647/3310/2353/4616/1847/8817 | 20 |
| hsa05219 | Bladder cancer | 6/274 | 41/8063 | 0.0024083 | 0.0221268 | 0.0150807 | 4609/595/5604/1026/1839/7057 | 6 |
| hsa04917 | Prolactin signaling pathway | 8/274 | 70/8063 | 0.0024391 | 0.0221268 | 0.0150807 | 3717/6774/894/595/8651/5604/2353/8835 | 8 |
| hsa05230 | Central carbon metabolism in cancer | 8/274 | 70/8063 | 0.0024391 | 0.0221268 | 0.0150807 | 4609/6513/3939/5159/8140/5156/5604/9123 | 8 |
| hsa04218 | Cellular senescence | 13/274 | 156/8063 | 0.0024583 | 0.0221268 | 0.0150807 | 4609/894/823/595/5054/286826/3710/10912/5604/1647/1026/3569/4616 | 13 |
| hsa05210 | Colorectal cancer | 9/274 | 86/8063 | 0.0024674 | 0.0221268 | 0.0150807 | 4609/595/5880/10912/5604/1647/2353/1026/4616 | 9 |
| hsa04512 | ECM-receptor interaction | 9/274 | 88/8063 | 0.0028903 | 0.0251098 | 0.0171139 | 1292/7060/6696/3678/1293/960/3371/1311/7057 | 9 |
| hsa05134 | Legionellosis | 7/274 | 57/8063 | 0.0029927 | 0.0252115 | 0.0171832 | 3689/4792/929/3306/2920/3310/3569 | 7 |
| hsa05166 | Human T-cell leukemia virus 1 infection | 16/274 | 219/8063 | 0.0031665 | 0.0252762 | 0.0172272 | 7538/4609/3689/4792/894/4055/595/6513/7132/109/5604/3126/2353/1026/3569/1958 | 16 |
| hsa04115 | p53 signaling pathway | 8/274 | 73/8063 | 0.0031823 | 0.0252762 | 0.0172272 | 894/595/5054/10912/1647/1026/4616/7057 | 8 |
| hsa05213 | Endometrial cancer | 7/274 | 58/8063 | 0.003307 | 0.025537 | 0.017405 | 4609/595/10912/5604/1647/1026/4616 | 7 |
| hsa05214 | Glioma | 8/274 | 75/8063 | 0.003768 | 0.027833 | 0.0189698 | 595/5159/10912/5156/5604/1647/1026/4616 | 8 |
| hsa05416 | Viral myocarditis | 7/274 | 60/8063 | 0.0040103 | 0.027833 | 0.0189698 | 4624/3689/595/60/5880/3126/6445 | 7 |
| hsa05212 | Pancreatic cancer | 8/274 | 76/8063 | 0.0040906 | 0.027833 | 0.0189698 | 6774/595/5880/10912/5604/1647/1026/4616 | 8 |
| hsa05220 | Chronic myeloid leukemia | 8/274 | 76/8063 | 0.0040906 | 0.027833 | 0.0189698 | 4609/4792/595/10912/5604/1647/1026/4616 | 8 |
| hsa04380 | Osteoclast differentiation | 11/274 | 128/8063 | 0.0041049 | 0.027833 | 0.0189698 | 4792/1535/353514/2212/7305/7132/8651/5604/2353/1513/3726 | 11 |
| hsa05140 | Leishmaniasis | 8/274 | 77/8063 | 0.004434 | 0.0293486 | 0.0200029 | 3717/3689/4792/1535/2212/1536/3126/2353 | 8 |
| hsa05150 | Staphylococcus aureus infection | 9/274 | 96/8063 | 0.0051851 | 0.0335223 | 0.0228474 | 715/2357/3689/714/713/5648/2212/1675/3126 | 9 |
| hsa05167 | Kaposi sarcoma-associated herpesvirus infection | 14/274 | 193/8063 | 0.0059865 | 0.0377361 | 0.0257194 | 7538/4609/3717/6774/3055/4792/595/7132/3710/2920/5604/2353/1026/3569 | 14 |
| hsa04921 | Oxytocin signaling pathway | 12/274 | 154/8063 | 0.0061084 | 0.0377361 | 0.0257194 | 595/109/60/3710/5604/3759/8605/5997/2353/6262/1026/4878 | 12 |
| hsa05144 | Malaria | 6/274 | 50/8063 | 0.0065996 | 0.0398848 | 0.0271839 | 3689/7060/6347/3569/1311/7057 | 6 |
| hsa04913 | Ovarian steroidogenesis | 6/274 | 51/8063 | 0.0072719 | 0.0430123 | 0.0293155 | 240/1573/109/8605/3949/1545 | 6 |
| hsa05142 | Chagas disease | 9/274 | 102/8063 | 0.007683 | 0.0444973 | 0.0303276 | 4792/714/713/2769/7132/5054/6347/2353/3569 | 9 |
| hsa05223 | Non-small cell lung cancer | 7/274 | 68/8063 | 0.008003 | 0.0448639 | 0.0305774 | 6774/595/10912/5604/1647/1026/4616 | 7 |
| hsa04974 | Protein digestion and absorption | 9/274 | 103/8063 | 0.0081758 | 0.0448639 | 0.0305774 | 59272/81578/477/1292/476/1307/483/1293/54407 | 9 |
| hsa05131 | Shigellosis | 16/274 | 242/8063 | 0.0082304 | 0.0448639 | 0.0305774 | 5580/10095/3059/4792/929/823/51196/7132/10094/60/5588/81873/3710/3678/960/58498 | 16 |
| hsa04920 | Adipocytokine signaling pathway | 7/274 | 69/8063 | 0.0086554 | 0.0455939 | 0.031075 | 3717/6774/4792/6513/7132/5588/1375 | 7 |
| hsa04620 | Toll-like receptor signaling pathway | 9/274 | 104/8063 | 0.0086924 | 0.0455939 | 0.031075 | 4792/929/1326/23643/5604/6696/2353/1513/3569 | 9 |
| hsa05206 | MicroRNAs in cancer | 19/274 | 310/8063 | 0.0091817 | 0.0472688 | 0.0322165 | 4609/6774/5292/894/54541/6541/595/5328/6659/5159/8651/5156/5604/3678/1026/960/3371/7057/1545 | 19 |
| hsa04928 | Parathyroid hormone synthesis, secretion and action | 9/274 | 106/8063 | 0.0097994 | 0.0495314 | 0.0337587 | 9935/109/3710/5604/5144/2353/1026/1839/1958 | 9 |
