# Supplementary material for: Identification of Potential miRNA-mRNA Regulatory Network Contributing to Hypertrophic Cardiomyopathy (HCM)
Source: Front Cardiovasc Med. 2021 May 31;8:660372. doi: 10.3389/fcvm.2021.660372 (PMC8200816; doi:10.3389/fcvm.2021.660372)
Supplement: Supplementary Table 2 — Functional enrichment of targeted genes of 24 differentially expressed miRNAs (DEMs). [file Table_2.DOCX]

Table S2 Functional enrichment of targeted genes of 24 differentially expressed miRNAs (DEMs).

GO terms

| ONTOLOGY | ID | Description | GeneRatio | BgRatio | pvalue | p.adjust | qvalue | geneID | Count |
| --- | --- | --- | --- | --- | --- | --- | --- | --- | --- |
| BP | GO:0035265 | organ growth | 17/350 | 172/17653 | 5.92E-08 | 0.000144 | 0.000112 | BCL2/NOTCH1/PDGFRB/RARG/IGF1/TGFBR2/TBX20/FOXC1/SERP1/SORBS2/NLGN4X/BMPR1A/JARID2/ZFPM2/AKT1/PTEN/CARM1 | 17 |
| BP | GO:0003215 | cardiac right ventricle morphogenesis | 7/350 | 20/17653 | 7.04E-08 | 0.000144 | 0.000112 | NOTCH1/SOX4/TBX20/HAND2/BMPR1A/ZFPM2/PPP1R13L | 7 |
| BP | GO:0048638 | regulation of developmental growth | 22/350 | 323/17653 | 5.43E-07 | 0.0007397 | 0.0005751 | CDK4/BCL2/NOTCH1/MAPT/IGF1/TGFBR2/EZR/TBX20/FOXC1/RAI1/SERP1/CSF1/BMPR1A/DUSP10/JARID2/ZFPM2/AKT1/PTEN/CARM1/CRABP2/TRPV2/RND2 | 22 |
| BP | GO:0001649 | osteoblast differentiation | 17/350 | 205/17653 | 7.41E-07 | 0.0007579 | 0.0005893 | CDK6/SOX2/NOTCH1/SNAI1/IGF1/RUNX2/IL6R/HAND2/SATB2/IL6ST/BMP3/HNRNPC/ITGA11/BMPR1A/SND1/AKT1/SKI | 17 |
| BP | GO:0046620 | regulation of organ growth | 12/350 | 105/17653 | 1.14E-06 | 0.000934 | 0.0007262 | NOTCH1/IGF1/TGFBR2/TBX20/FOXC1/SERP1/BMPR1A/JARID2/ZFPM2/AKT1/PTEN/CARM1 | 12 |
| BP | GO:0001503 | ossification | 23/350 | 371/17653 | 1.53E-06 | 0.000989 | 0.000769 | BCL2/INPPL1/CDK6/SOX2/NOTCH1/SNAI1/IGF1/RUNX2/IL6R/ALOX15/FOXC1/HAND2/SATB2/IL6ST/BMP3/CSF1/HNRNPC/ITGA11/BMPR1A/SND1/AKT1/SKI/OMD | 23 |
| BP | GO:0003208 | cardiac ventricle morphogenesis | 10/350 | 73/17653 | 1.69E-06 | 0.000989 | 0.000769 | NOTCH1/SOX4/SMAD4/TGFBR2/TBX20/FOXC1/HAND2/BMPR1A/ZFPM2/PPP1R13L | 10 |
| BP | GO:0070482 | response to oxygen levels | 23/350 | 378/17653 | 2.10E-06 | 0.0010734 | 0.0008346 | MYB/CAV1/CDK4/BCL2/MYC/NOTCH1/PDGFRB/MDM4/SMAD4/TGFBR2/ITPR1/FAM162A/LDHA/UCP2/CYGB/CDKN1B/VASN/OPRD1/AKT1/PRKCB/PTEN/PKM/TM9SF4 | 23 |
| BP | GO:0003272 | endocardial cushion formation | 6/350 | 21/17653 | 2.46E-06 | 0.0011084 | 0.0008618 | NOTCH1/SNAI1/SMAD4/TGFBR2/TBX20/BMPR1A | 6 |
| BP | GO:0048639 | positive regulation of developmental growth | 15/350 | 178/17653 | 2.73E-06 | 0.0011084 | 0.0008618 | BCL2/NOTCH1/MAPT/IGF1/TGFBR2/EZR/TBX20/SERP1/CSF1/BMPR1A/ZFPM2/AKT1/CRABP2/TRPV2/RND2 | 15 |
| BP | GO:0072132 | mesenchyme morphogenesis | 8/350 | 46/17653 | 2.98E-06 | 0.0011084 | 0.0008618 | MYC/NOTCH1/SNAI1/SMAD4/TGFBR2/TBX20/FOXC1/BMPR1A | 8 |
| BP | GO:0031099 | regeneration | 15/350 | 186/17653 | 4.70E-06 | 0.0016032 | 0.0012465 | CDK4/BCL2/SOX2/NOTCH1/IGF1/TGFBR2/MAP2K1/THY1/UCP2/DUSP10/BIN3/PTEN/CCND1/AXL/PKM | 15 |
| BP | GO:0003171 | atrioventricular valve development | 6/350 | 24/17653 | 5.79E-06 | 0.0017793 | 0.0013834 | NOTCH1/SOX4/SMAD4/TGFBR2/TBX20/BMPR1A | 6 |
| BP | GO:0001666 | response to hypoxia | 21/350 | 346/17653 | 6.09E-06 | 0.0017793 | 0.0013834 | MYB/CAV1/BCL2/MYC/NOTCH1/MDM4/SMAD4/TGFBR2/ITPR1/FAM162A/LDHA/UCP2/CYGB/CDKN1B/VASN/OPRD1/AKT1/PRKCB/PTEN/PKM/TM9SF4 | 21 |
| BP | GO:0048008 | platelet-derived growth factor receptor signaling pathway | 8/350 | 52/17653 | 7.75E-06 | 0.0021137 | 0.0016434 | PDGFRA/PDGFRB/TXNIP/PTPRJ/BCAR1/PDGFB/PTEN/SNCA | 8 |
| BP | GO:0036293 | response to decreased oxygen levels | 21/350 | 354/17653 | 8.65E-06 | 0.002159 | 0.0016787 | MYB/CAV1/BCL2/MYC/NOTCH1/MDM4/SMAD4/TGFBR2/ITPR1/FAM162A/LDHA/UCP2/CYGB/CDKN1B/VASN/OPRD1/AKT1/PRKCB/PTEN/PKM/TM9SF4 | 21 |
| BP | GO:0048146 | positive regulation of fibroblast proliferation | 8/350 | 53/17653 | 8.97E-06 | 0.002159 | 0.0016787 | MYB/CDK4/MYC/CDK6/PDGFRA/PDGFRB/IGF1/PDGFB | 8 |
| BP | GO:0045785 | positive regulation of cell adhesion | 22/350 | 392/17653 | 1.26E-05 | 0.0027353 | 0.0021267 | MYB/CAV1/CDK6/ZAP70/SOX2/IGF1/TGFBR2/ALOX15/EFNB1/PTPRJ/CYTH3/THY1/IL6ST/PODXL/IL7R/CSF1/VAV3/DUSP10/TNFRSF13C/PLPP3/AKT1/MYO10 | 22 |
| BP | GO:0000302 | response to reactive oxygen species | 16/350 | 227/17653 | 1.27E-05 | 0.0027353 | 0.0021267 | MYB/MET/BCL2/MAPT/PDGFRA/PDGFRB/TXNIP/FOSL1/LDHA/UCP2/SESN2/TRA2B/AKT1/AXL/PXN/CBX8 | 16 |
| BP | GO:0010642 | negative regulation of platelet-derived growth factor receptor signaling pathway | 5/350 | 17/17653 | 1.51E-05 | 0.002839 | 0.0022074 | PDGFRA/PDGFRB/PTPRJ/PDGFB/SNCA | 5 |
| BP | GO:0001836 | release of cytochrome c from mitochondria | 8/350 | 57/17653 | 1.56E-05 | 0.002839 | 0.0022074 | BCL2/BMF/IGF1/FAM162A/MOAP1/APOPT1/AKT1/BCL2L1 | 8 |
| BP | GO:0022409 | positive regulation of cell-cell adhesion | 17/350 | 257/17653 | 1.57E-05 | 0.002839 | 0.0022074 | MYB/CAV1/ZAP70/SOX2/IGF1/TGFBR2/ALOX15/EFNB1/THY1/IL6ST/PODXL/IL7R/DUSP10/TNFRSF13C/PLPP3/AKT1/MYO10 | 17 |
| BP | GO:0048568 | embryonic organ development | 23/350 | 428/17653 | 1.61E-05 | 0.002839 | 0.0022074 | MYCN/NOTCH1/SNAI1/PDGFRA/RARG/RUNX2/TGFBR2/MAP2K1/TBX20/FOXC1/HAND2/EN1/USH1G/SATB2/TRA2B/PAX5/BMPR1A/CLIC5/MTHFD1/ZFPM2/PDGFB/AKT1/PPP1R13L | 23 |
| BP | GO:0055017 | cardiac muscle tissue growth | 10/350 | 94/17653 | 1.70E-05 | 0.002839 | 0.0022074 | NOTCH1/IGF1/TGFBR2/TBX20/FOXC1/SORBS2/BMPR1A/JARID2/ZFPM2/PTEN | 10 |
| BP | GO:0048732 | gland development | 23/350 | 430/17653 | 1.74E-05 | 0.002839 | 0.0022074 | AKT2/CAV1/MET/BCL2/NOTCH4/SOX2/NOTCH1/RARG/SMAD4/TGFBR2/CDH1/MAP2K1/FOXC1/HAND2/UCP2/CDKN1B/CSF1/BMPR1A/JARID2/AKT1/PTEN/CCND1/PKM | 23 |
| BP | GO:0003203 | endocardial cushion morphogenesis | 6/350 | 29/17653 | 1.88E-05 | 0.0029573 | 0.0022993 | NOTCH1/SNAI1/SMAD4/TGFBR2/TBX20/BMPR1A | 6 |
| BP | GO:0033002 | muscle cell proliferation | 15/350 | 211/17653 | 2.14E-05 | 0.0032439 | 0.0025221 | MYB/NOTCH1/PDGFRB/IGF1/TGFBR2/IL6R/TBX20/FOXC1/CDKN1B/BMPR1A/JARID2/ZFPM2/PDGFB/AKT1/PTEN | 15 |
| BP | GO:0014706 | striated muscle tissue development | 21/350 | 379/17653 | 2.41E-05 | 0.0033638 | 0.0026154 | CAV1/BCL2/NOTCH1/PDGFRA/PDGFRB/IGF1/SMAD4/TGFBR2/TBX20/FOXC1/FOXN2/MKL2/PAX5/SORBS2/BMPR1A/JARID2/ZFPM2/BIN3/PTEN/PPP1R13L/SKI | 21 |
| BP | GO:0008361 | regulation of cell size | 13/350 | 164/17653 | 2.43E-05 | 0.0033638 | 0.0026154 | CDK4/MAPT/RARG/IL7R/LRRC8A/VAV3/AKT1/PTEN/AKT1S1/CRABP2/SHANK3/TRPV2/RND2 | 13 |
| BP | GO:0061448 | connective tissue development | 16/350 | 240/17653 | 2.53E-05 | 0.0033638 | 0.0026154 | CDK4/MYCN/NOTCH1/SNAI1/PDGFRB/RARG/RUNX2/TGFBR2/HAND2/LNPK/SATB2/BMP3/CSF1/BMPR1A/PDGFB/CARM1 | 16 |
| BP | GO:0009749 | response to glucose | 14/350 | 189/17653 | 2.55E-05 | 0.0033638 | 0.0026154 | HNF4A/IGF1/SOX4/TXNIP/TGFBR2/LDHA/UCP2/SESN2/CDKN1B/TRA2B/SELENOS/PTEN/CDK16/RAB11B | 14 |
| BP | GO:0090199 | regulation of release of cytochrome c from mitochondria | 7/350 | 45/17653 | 2.69E-05 | 0.0034405 | 0.002675 | BMF/IGF1/FAM162A/MOAP1/APOPT1/AKT1/BCL2L1 | 7 |
| BP | GO:0003231 | cardiac ventricle development | 11/350 | 121/17653 | 2.91E-05 | 0.0036017 | 0.0028003 | NOTCH1/SOX4/DCTN5/SMAD4/TGFBR2/TBX20/FOXC1/HAND2/BMPR1A/ZFPM2/PPP1R13L | 11 |
| BP | GO:0060419 | heart growth | 10/350 | 101/17653 | 3.20E-05 | 0.0037593 | 0.0029229 | NOTCH1/IGF1/TGFBR2/TBX20/FOXC1/SORBS2/BMPR1A/JARID2/ZFPM2/PTEN | 10 |
| BP | GO:0009746 | response to hexose | 14/350 | 193/17653 | 3.22E-05 | 0.0037593 | 0.0029229 | HNF4A/IGF1/SOX4/TXNIP/TGFBR2/LDHA/UCP2/SESN2/CDKN1B/TRA2B/SELENOS/PTEN/CDK16/RAB11B | 14 |
| BP | GO:0009743 | response to carbohydrate | 15/350 | 220/17653 | 3.49E-05 | 0.0039607 | 0.0030795 | HNF4A/IGF1/SOX4/TXNIP/TGFBR2/LDHA/UCP2/SESN2/CDKN1B/TRA2B/SELENOS/PRKCB/PTEN/CDK16/RAB11B | 15 |
| BP | GO:0060038 | cardiac muscle cell proliferation | 8/350 | 64/17653 | 3.71E-05 | 0.0040992 | 0.0031871 | NOTCH1/TGFBR2/TBX20/FOXC1/BMPR1A/JARID2/ZFPM2/PTEN | 8 |
| BP | GO:0035107 | appendage morphogenesis | 12/350 | 149/17653 | 4.26E-05 | 0.0043594 | 0.0033894 | MYCN/NOTCH1/RARG/RUNX2/SOX4/SMAD4/HAND2/LNPK/EN1/BMPR1A/CRABP2/SKI | 12 |
| BP | GO:0035108 | limb morphogenesis | 12/350 | 149/17653 | 4.26E-05 | 0.0043594 | 0.0033894 | MYCN/NOTCH1/RARG/RUNX2/SOX4/SMAD4/HAND2/LNPK/EN1/BMPR1A/CRABP2/SKI | 12 |
| BP | GO:0034284 | response to monosaccharide | 14/350 | 198/17653 | 4.26E-05 | 0.0043594 | 0.0033894 | HNF4A/IGF1/SOX4/TXNIP/TGFBR2/LDHA/UCP2/SESN2/CDKN1B/TRA2B/SELENOS/PTEN/CDK16/RAB11B | 14 |
| BP | GO:0060537 | muscle tissue development | 21/350 | 396/17653 | 4.57E-05 | 0.0045424 | 0.0035318 | CAV1/BCL2/NOTCH1/PDGFRA/PDGFRB/IGF1/SMAD4/TGFBR2/TBX20/FOXC1/FOXN2/MKL2/PAX5/SORBS2/BMPR1A/JARID2/ZFPM2/BIN3/PTEN/PPP1R13L/SKI | 21 |
| BP | GO:0003181 | atrioventricular valve morphogenesis | 5/350 | 21/17653 | 4.66E-05 | 0.0045424 | 0.0035318 | NOTCH1/SOX4/SMAD4/TGFBR2/BMPR1A | 5 |
| BP | GO:0001501 | skeletal system development | 24/350 | 493/17653 | 5.20E-05 | 0.0047813 | 0.0037175 | INPPL1/MYCN/SNAI1/PDGFRA/RARG/IGF1/RUNX2/SOX4/TGFBR2/FOXC1/RAI1/HAND2/LNPK/FOXN3/EN1/SATB2/BMP3/SERP1/HOXA13/PAX5/BMPR1A/MTHFD1/CARM1/SKI | 24 |
| BP | GO:0030326 | embryonic limb morphogenesis | 11/350 | 129/17653 | 5.26E-05 | 0.0047813 | 0.0037175 | MYCN/NOTCH1/RARG/RUNX2/SMAD4/HAND2/LNPK/EN1/BMPR1A/CRABP2/SKI | 11 |
| BP | GO:0035113 | embryonic appendage morphogenesis | 11/350 | 129/17653 | 5.26E-05 | 0.0047813 | 0.0037175 | MYCN/NOTCH1/RARG/RUNX2/SMAD4/HAND2/LNPK/EN1/BMPR1A/CRABP2/SKI | 11 |
| BP | GO:0048145 | regulation of fibroblast proliferation | 9/350 | 87/17653 | 5.62E-05 | 0.0049999 | 0.0038875 | MYB/CDK4/MYC/CDK6/PDGFRA/PDGFRB/IGF1/PDGFB/SKI | 9 |
| BP | GO:0060485 | mesenchyme development | 16/350 | 257/17653 | 5.78E-05 | 0.005027 | 0.0039085 | BCL2/MYC/NOTCH1/SNAI1/PDGFRB/SMAD4/TGFBR2/TBX20/FOXC1/EFNB1/DAB2/HAND2/VASN/BMPR1A/ZFPM2/PTEN | 16 |
| BP | GO:0000079 | regulation of cyclin-dependent protein serine/threonine kinase activity | 9/350 | 88/17653 | 6.16E-05 | 0.0051386 | 0.0039953 | CDK4/CCNE2/CDK6/CDKN1B/PDGFB/AKT1/PTEN/CCND1/CDC37 | 9 |
| BP | GO:0048144 | fibroblast proliferation | 9/350 | 88/17653 | 6.16E-05 | 0.0051386 | 0.0039953 | MYB/CDK4/MYC/CDK6/PDGFRA/PDGFRB/IGF1/PDGFB/SKI | 9 |
| BP | GO:0055021 | regulation of cardiac muscle tissue growth | 8/350 | 69/17653 | 6.43E-05 | 0.0052585 | 0.0040885 | NOTCH1/IGF1/TGFBR2/TBX20/BMPR1A/JARID2/ZFPM2/PTEN | 8 |
| BP | GO:0003207 | cardiac chamber formation | 4/350 | 12/17653 | 6.63E-05 | 0.0053166 | 0.0041337 | NOTCH1/SOX4/TBX20/HAND2 | 4 |
| BP | GO:0003179 | heart valve morphogenesis | 6/350 | 36/17653 | 6.86E-05 | 0.0053939 | 0.0041938 | NOTCH1/SOX4/SMAD4/TGFBR2/TBX20/BMPR1A | 6 |
| BP | GO:0042770 | signal transduction in response to DNA damage | 11/350 | 134/17653 | 7.44E-05 | 0.0057452 | 0.0044669 | SNAI1/SOX4/MDM4/SPRED1/CNOT4/DYRK3/SESN2/CDKN1B/CHEK1/CARM1/FZR1 | 11 |
| BP | GO:0055024 | regulation of cardiac muscle tissue development | 9/350 | 91/17653 | 8.02E-05 | 0.0060722 | 0.0047212 | NOTCH1/IGF1/SMAD4/TGFBR2/TBX20/BMPR1A/JARID2/ZFPM2/PTEN | 9 |
| BP | GO:0010639 | negative regulation of organelle organization | 19/350 | 354/17653 | 8.95E-05 | 0.0065854 | 0.0051202 | MET/MAPT/IGF1/SMAD4/EVI5L/GMFB/HNRNPC/PAX5/CHEK1/JARID2/GNL3L/AKT1/SNCA/KANK2/TERF2/BCL2L1/MAP4/SHANK3/SKI | 19 |
| BP | GO:0060043 | regulation of cardiac muscle cell proliferation | 7/350 | 54/17653 | 9.02E-05 | 0.0065854 | 0.0051202 | NOTCH1/TGFBR2/TBX20/BMPR1A/JARID2/ZFPM2/PTEN | 7 |
| BP | GO:0010640 | regulation of platelet-derived growth factor receptor signaling pathway | 5/350 | 24/17653 | 9.28E-05 | 0.0066575 | 0.0051763 | PDGFRA/PDGFRB/PTPRJ/PDGFB/SNCA | 5 |
| BP | GO:1904029 | regulation of cyclin-dependent protein kinase activity | 9/350 | 93/17653 | 9.50E-05 | 0.0067016 | 0.0052105 | CDK4/CCNE2/CDK6/CDKN1B/PDGFB/AKT1/PTEN/CCND1/CDC37 | 9 |
| BP | GO:0003197 | endocardial cushion development | 6/350 | 39/17653 | 0.0001093 | 0.0075743 | 0.0058891 | NOTCH1/SNAI1/SMAD4/TGFBR2/TBX20/BMPR1A | 6 |
| BP | GO:0003148 | outflow tract septum morphogenesis | 5/350 | 25/17653 | 0.0001141 | 0.0077781 | 0.0060475 | SMAD4/TGFBR2/TBX20/BMPR1A/ZFPM2 | 5 |
| BP | GO:0060420 | regulation of heart growth | 8/350 | 75/17653 | 0.000117 | 0.0078457 | 0.0061001 | NOTCH1/IGF1/TGFBR2/TBX20/BMPR1A/JARID2/ZFPM2/PTEN | 8 |
| BP | GO:0003170 | heart valve development | 6/350 | 40/17653 | 0.0001264 | 0.0083396 | 0.0064841 | NOTCH1/SOX4/SMAD4/TGFBR2/TBX20/BMPR1A | 6 |
| BP | GO:0016202 | regulation of striated muscle tissue development | 11/350 | 143/17653 | 0.0001335 | 0.0086697 | 0.0067407 | BCL2/NOTCH1/IGF1/SMAD4/TGFBR2/TBX20/MKL2/BMPR1A/JARID2/ZFPM2/PTEN | 11 |
| BP | GO:0048738 | cardiac muscle tissue development | 14/350 | 221/17653 | 0.0001387 | 0.008866 | 0.0068934 | NOTCH1/PDGFRA/PDGFRB/IGF1/SMAD4/TGFBR2/TBX20/FOXC1/SORBS2/BMPR1A/JARID2/ZFPM2/PTEN/PPP1R13L | 14 |
| BP | GO:0014855 | striated muscle cell proliferation | 8/350 | 77/17653 | 0.000141 | 0.0088735 | 0.0068992 | NOTCH1/TGFBR2/TBX20/FOXC1/BMPR1A/JARID2/ZFPM2/PTEN | 8 |
| BP | GO:0007050 | cell cycle arrest | 15/350 | 250/17653 | 0.0001476 | 0.0091439 | 0.0071094 | AKT2/CDK4/MYC/CDK6/SOX2/NOTCH1/SOX4/MDM4/MAP2K1/CNOT4/CDKN1B/GAS1/RRAGD/CCND1/CARM1 | 15 |
| BP | GO:1901861 | regulation of muscle tissue development | 11/350 | 145/17653 | 0.0001511 | 0.0092216 | 0.0071699 | BCL2/NOTCH1/IGF1/SMAD4/TGFBR2/TBX20/MKL2/BMPR1A/JARID2/ZFPM2/PTEN | 11 |
| BP | GO:0010001 | glial cell differentiation | 13/350 | 197/17653 | 0.0001597 | 0.009603 | 0.0074664 | AKT2/CDK6/MYCN/SOX2/NOTCH1/MAPT/SOX4/MAP2K1/IL6ST/DUSP10/AKT1/PTEN/SKI | 13 |
| BP | GO:0048634 | regulation of muscle organ development | 11/350 | 147/17653 | 0.0001705 | 0.0101065 | 0.0078579 | BCL2/NOTCH1/IGF1/SMAD4/TGFBR2/TBX20/MKL2/BMPR1A/JARID2/ZFPM2/PTEN | 11 |
| BP | GO:0031657 | regulation of cyclin-dependent protein serine/threonine kinase activity involved in G1/S transition of mitotic cell cycle | 4/350 | 15/17653 | 0.0001744 | 0.0101705 | 0.0079076 | CDK4/CDK6/AKT1/PTEN | 4 |
| BP | GO:0006979 | response to oxidative stress | 21/350 | 436/17653 | 0.0001766 | 0.0101705 | 0.0079076 | MYB/MET/BCL2/MAPT/PDGFRA/PDGFRB/HSPA1B/TXNIP/FOSL1/LDHA/UCP2/CYGB/SESN2/TRA2B/SELENOS/AKT1/AXL/SNCA/PXN/CBX8/NFE2L1 | 21 |
| BP | GO:0003206 | cardiac chamber morphogenesis | 10/350 | 124/17653 | 0.0001822 | 0.010349 | 0.0080464 | NOTCH1/SOX4/SMAD4/TGFBR2/TBX20/FOXC1/HAND2/BMPR1A/ZFPM2/PPP1R13L | 10 |
| BP | GO:0001654 | eye development | 18/350 | 344/17653 | 0.0001872 | 0.010486 | 0.0081529 | CDK4/BCL2/SOX2/PDGFRA/PDGFRB/YY1/RARG/ZDHHC16/TGFBR2/FOXC1/THY1/CDKN1B/TGIF2/PPP1R13L/FKBP8/FZR1/RAB11FIP4/SKI | 18 |
| BP | GO:0051251 | positive regulation of lymphocyte activation | 17/350 | 315/17653 | 0.0001955 | 0.0105619 | 0.0082119 | MYB/CAV1/BCL2/NFATC2/ZAP70/UNG/IGF1/TGFBR2/EFNB1/THY1/IL6ST/IL7R/VAV3/DUSP10/TNFRSF13C/AKT1/AXL | 17 |
| BP | GO:0032835 | glomerulus development | 7/350 | 61/17653 | 0.0001973 | 0.0105619 | 0.0082119 | BCL2/NOTCH1/PDGFRA/PDGFRB/FOXC1/PODXL/PDGFB | 7 |
| BP | GO:0046622 | positive regulation of organ growth | 7/350 | 61/17653 | 0.0001973 | 0.0105619 | 0.0082119 | NOTCH1/IGF1/TBX20/SERP1/BMPR1A/ZFPM2/AKT1 | 7 |
| BP | GO:2000045 | regulation of G1/S transition of mitotic cell cycle | 12/350 | 175/17653 | 0.0001988 | 0.0105619 | 0.0082119 | CDK4/BCL2/CDK6/SOX4/MDM4/CNOT4/CDKN1B/AKT1/PTEN/CCND1/KANK2/CARM1 | 12 |
| BP | GO:0048736 | appendage development | 12/350 | 176/17653 | 0.0002097 | 0.0108549 | 0.0084398 | MYCN/NOTCH1/RARG/RUNX2/SOX4/SMAD4/HAND2/LNPK/EN1/BMPR1A/CRABP2/SKI | 12 |
| BP | GO:0060173 | limb development | 12/350 | 176/17653 | 0.0002097 | 0.0108549 | 0.0084398 | MYCN/NOTCH1/RARG/RUNX2/SOX4/SMAD4/HAND2/LNPK/EN1/BMPR1A/CRABP2/SKI | 12 |
| BP | GO:0042063 | gliogenesis | 15/350 | 260/17653 | 0.0002262 | 0.0114172 | 0.0088769 | AKT2/MYB/CDK6/MYCN/SOX2/NOTCH1/MAPT/SOX4/MAP2K1/IL6ST/CSF1/DUSP10/AKT1/PTEN/SKI | 15 |
| BP | GO:0045927 | positive regulation of growth | 15/350 | 260/17653 | 0.0002262 | 0.0114172 | 0.0088769 | BCL2/NOTCH1/MAPT/IGF1/TGFBR2/EZR/TBX20/SERP1/CSF1/BMPR1A/ZFPM2/AKT1/CRABP2/TRPV2/RND2 | 15 |
| BP | GO:0003198 | epithelial to mesenchymal transition involved in endocardial cushion formation | 4/350 | 16/17653 | 0.0002289 | 0.0114172 | 0.0088769 | NOTCH1/SNAI1/SMAD4/TGFBR2 | 4 |
| BP | GO:0042246 | tissue regeneration | 7/350 | 63/17653 | 0.0002419 | 0.0119182 | 0.0092665 | SOX2/NOTCH1/IGF1/TGFBR2/DUSP10/BIN3/PKM | 7 |
| BP | GO:0051881 | regulation of mitochondrial membrane potential | 7/350 | 64/17653 | 0.000267 | 0.0130008 | 0.0101082 | AKT2/BCL2/MAPT/UCP2/OPRD1/AKT1/BCL2L1 | 7 |
| BP | GO:0048705 | skeletal system morphogenesis | 13/350 | 209/17653 | 0.0002852 | 0.0137247 | 0.0106711 | INPPL1/MYCN/PDGFRA/RARG/RUNX2/TGFBR2/FOXC1/FOXN3/SATB2/PAX5/MTHFD1/CARM1/SKI | 13 |
| BP | GO:0022407 | regulation of cell-cell adhesion | 19/350 | 388/17653 | 0.0002903 | 0.0138043 | 0.0107329 | MYB/CAV1/ZAP70/SOX2/IGF1/TGFBR2/CDH1/ALOX15/PLA2G2D/EFNB1/THY1/IL6ST/PODXL/IL7R/DUSP10/TNFRSF13C/PLPP3/AKT1/MYO10 | 19 |
| BP | GO:0060977 | coronary vasculature morphogenesis | 4/350 | 17/17653 | 0.0002947 | 0.0138531 | 0.0107708 | NOTCH1/PDGFRB/SPRED1/HAND2 | 4 |
| BP | GO:0048661 | positive regulation of smooth muscle cell proliferation | 8/350 | 86/17653 | 0.0003048 | 0.0141661 | 0.0110143 | MYB/PDGFRB/IGF1/TGFBR2/IL6R/BMPR1A/PDGFB/AKT1 | 8 |
| BP | GO:0070371 | ERK1 and ERK2 cascade | 17/350 | 328/17653 | 0.0003138 | 0.0143185 | 0.0111327 | MET/MYC/NOTCH1/PDGFRA/PDGFRB/IGF1/CCL22/SPRED1/SMAD4/EZR/MAP2K1/ALOX15/HAND2/DUSP10/PDGFB/PTEN/AXL | 17 |
| BP | GO:0007568 | aging | 16/350 | 298/17653 | 0.0003174 | 0.0143185 | 0.0111327 | BCL2/CDK6/PDGFRB/TGFBR2/MAP2K1/UCP2/SERP1/PAX5/BMPR1A/CHEK1/AKT1/PTEN/SNCA/TERF2/CARM1/FZR1 | 16 |
| BP | GO:0003205 | cardiac chamber development | 11/350 | 158/17653 | 0.0003196 | 0.0143185 | 0.0111327 | NOTCH1/SOX4/DCTN5/SMAD4/TGFBR2/TBX20/FOXC1/HAND2/BMPR1A/ZFPM2/PPP1R13L | 11 |
| BP | GO:0032946 | positive regulation of mononuclear cell proliferation | 10/350 | 133/17653 | 0.0003221 | 0.0143185 | 0.0111327 | BCL2/NFATC2/ZAP70/IGF1/TGFBR2/EFNB1/IL6ST/CSF1/VAV3/TNFRSF13C | 10 |
| BP | GO:0010717 | regulation of epithelial to mesenchymal transition | 8/350 | 87/17653 | 0.00033 | 0.0144383 | 0.0112259 | NOTCH1/SNAI1/SMAD4/TGFBR2/FOXC1/DAB2/VASN/PTEN | 8 |
| BP | GO:0030278 | regulation of ossification | 12/350 | 185/17653 | 0.0003318 | 0.0144383 | 0.0112259 | BCL2/CDK6/NOTCH1/IGF1/RUNX2/IL6R/HAND2/IL6ST/CSF1/BMPR1A/SKI/OMD | 12 |
| BP | GO:0030330 | DNA damage response, signal transduction by p53 class mediator | 9/350 | 110/17653 | 0.0003419 | 0.0147199 | 0.0114448 | SNAI1/SOX4/MDM4/SPRED1/CNOT4/DYRK3/SESN2/CDKN1B/CARM1 | 9 |
| BP | GO:0007369 | gastrulation | 12/350 | 186/17653 | 0.0003485 | 0.0147214 | 0.011446 | SOX2/NANOG/HNF4A/SNAI1/SOX7/SMAD4/TGFBR2/TBX20/FOXC1/BMPR1A/ITGB3/TGIF2 | 12 |
| BP | GO:0010718 | positive regulation of epithelial to mesenchymal transition | 6/350 | 48/17653 | 0.0003538 | 0.0147214 | 0.011446 | NOTCH1/SNAI1/SMAD4/TGFBR2/FOXC1/DAB2 | 6 |
| BP | GO:0060349 | bone morphogenesis | 8/350 | 88/17653 | 0.0003568 | 0.0147214 | 0.011446 | INPPL1/RARG/RUNX2/TGFBR2/FOXC1/FOXN3/CARM1/SKI | 8 |
| BP | GO:0050867 | positive regulation of cell activation | 18/350 | 363/17653 | 0.0003592 | 0.0147214 | 0.011446 | MYB/CAV1/BCL2/NFATC2/ZAP70/UNG/PDGFRB/IGF1/TGFBR2/EFNB1/THY1/IL6ST/IL7R/VAV3/DUSP10/TNFRSF13C/AKT1/AXL | 18 |
| BP | GO:0010256 | endomembrane system organization | 20/350 | 427/17653 | 0.0003599 | 0.0147214 | 0.011446 | AKT2/CAV1/UBXN2B/MAP2K1/EHD3/SYNGR2/POM121C/SYT7/LNPK/UBL4A/VPS37B/SERP1/VPS37D/RAB30/TMEM170A/AKT1/PRKCB/PTEN/SEC61A1/REEP3 | 20 |
| BP | GO:0043276 | anoikis | 5/350 | 32/17653 | 0.000386 | 0.0156297 | 0.0121522 | CAV1/BCL2/NOTCH1/BMF/AKT1 | 5 |
| BP | GO:0007517 | muscle organ development | 19/350 | 398/17653 | 0.0003981 | 0.0159615 | 0.0124102 | CAV1/BCL2/NOTCH1/IGF1/SMAD4/TGFBR2/TBX20/FOXC1/FOXN2/SERP1/MKL2/ITGA11/PAX5/BMPR1A/JARID2/ZFPM2/BIN3/PTEN/SKI | 19 |
| BP | GO:0072006 | nephron development | 10/350 | 137/17653 | 0.0004083 | 0.0162113 | 0.0126044 | BCL2/MYC/NOTCH1/PDGFRA/PDGFRB/SMAD4/FOXC1/PODXL/PDGFB/SEC61A1 | 10 |
| BP | GO:0060411 | cardiac septum morphogenesis | 7/350 | 69/17653 | 0.0004262 | 0.0166373 | 0.0129356 | NOTCH1/SOX4/SMAD4/TGFBR2/TBX20/BMPR1A/ZFPM2 | 7 |
| BP | GO:0000082 | G1/S transition of mitotic cell cycle | 15/350 | 276/17653 | 0.0004271 | 0.0166373 | 0.0129356 | CDK4/CCNE2/BCL2/MYC/CDK6/SOX4/MDM4/CNOT4/CDKN1B/AKT1/PTEN/CCND1/KANK2/CARM1/RPA1 | 15 |
| BP | GO:0060350 | endochondral bone morphogenesis | 6/350 | 50/17653 | 0.0004432 | 0.0167941 | 0.0130575 | INPPL1/RARG/RUNX2/TGFBR2/FOXC1/CARM1 | 6 |
| BP | GO:0061971 | replacement bone morphogenesis | 6/350 | 50/17653 | 0.0004432 | 0.0167941 | 0.0130575 | INPPL1/RARG/RUNX2/TGFBR2/FOXC1/CARM1 | 6 |
| BP | GO:0045667 | regulation of osteoblast differentiation | 9/350 | 114/17653 | 0.0004454 | 0.0167941 | 0.0130575 | CDK6/NOTCH1/IGF1/RUNX2/IL6R/HAND2/IL6ST/BMPR1A/SKI | 9 |
| BP | GO:0032885 | regulation of polysaccharide biosynthetic process | 5/350 | 33/17653 | 0.0004476 | 0.0167941 | 0.0130575 | AKT2/IGF1/SELENOS/PDGFB/AKT1 | 5 |
| BP | GO:0001933 | negative regulation of protein phosphorylation | 20/350 | 435/17653 | 0.0004559 | 0.0169495 | 0.0131783 | CAV1/CDK4/MYC/CDK6/SPRED1/SMAD4/EZR/GMFB/PTPRJ/THY1/CDKN1B/DUSP10/PKIA/PLPP3/AKT1/PTEN/SNCA/AKT1S1/FKBP8/MLLT1 | 20 |
| BP | GO:0070665 | positive regulation of leukocyte proliferation | 10/350 | 140/17653 | 0.0004848 | 0.0177688 | 0.0138154 | BCL2/NFATC2/ZAP70/IGF1/TGFBR2/EFNB1/IL6ST/CSF1/VAV3/TNFRSF13C | 10 |
| BP | GO:1902806 | regulation of cell cycle G1/S phase transition | 12/350 | 193/17653 | 0.0004866 | 0.0177688 | 0.0138154 | CDK4/BCL2/CDK6/SOX4/MDM4/CNOT4/CDKN1B/AKT1/PTEN/CCND1/KANK2/CARM1 | 12 |
| BP | GO:2000134 | negative regulation of G1/S transition of mitotic cell cycle | 9/350 | 116/17653 | 0.0005061 | 0.0183191 | 0.0142432 | BCL2/SOX4/MDM4/CNOT4/CDKN1B/PTEN/CCND1/KANK2/CARM1 | 9 |
| BP | GO:0042326 | negative regulation of phosphorylation | 21/350 | 473/17653 | 0.0005225 | 0.0187472 | 0.0145761 | CAV1/CDK4/MYC/CDK6/MAPT/SPRED1/SMAD4/EZR/GMFB/PTPRJ/THY1/CDKN1B/DUSP10/PKIA/PLPP3/AKT1/PTEN/SNCA/AKT1S1/FKBP8/MLLT1 | 21 |
| BP | GO:0071901 | negative regulation of protein serine/threonine kinase activity | 10/350 | 142/17653 | 0.0005422 | 0.019282 | 0.0149918 | CAV1/CDK4/CDK6/SPRED1/PTPRJ/CDKN1B/DUSP10/PKIA/AKT1/PTEN | 10 |
| BP | GO:0033673 | negative regulation of kinase activity | 15/350 | 283/17653 | 0.0005546 | 0.0195532 | 0.0152028 | CAV1/CDK4/CDK6/MAPT/SPRED1/GMFB/PTPRJ/THY1/CDKN1B/DUSP10/PKIA/AKT1/PTEN/AKT1S1/MLLT1 | 15 |
| BP | GO:0035264 | multicellular organism growth | 10/350 | 143/17653 | 0.0005729 | 0.0200263 | 0.0155706 | CDK4/BCL2/RARG/IGF1/EZR/RAI1/EN1/CSF1/PPP1R13L/FKBP8 | 10 |
| BP | GO:2000826 | regulation of heart morphogenesis | 5/350 | 35/17653 | 0.0005927 | 0.0205421 | 0.0159716 | NOTCH1/SMAD4/TGFBR2/FOXC1/HAND2 | 5 |
| BP | GO:0010821 | regulation of mitochondrion organization | 13/350 | 226/17653 | 0.0006012 | 0.0205467 | 0.0159752 | BCL2/MAPT/BMF/IGF1/CDKL2/FAM162A/MOAP1/MIEF1/APOPT1/AKT1/CDC37/BCL2L1/PLAGL2 | 13 |
| BP | GO:0060562 | epithelial tube morphogenesis | 16/350 | 316/17653 | 0.0006028 | 0.0205467 | 0.0159752 | MET/BCL2/MYC/NOTCH4/MYCN/NOTCH1/RARG/SOX4/SMAD4/TGFBR2/TBX20/HAND2/PODXL/CSF1/MTHFD1/SKI | 16 |
| BP | GO:1901988 | negative regulation of cell cycle phase transition | 14/350 | 256/17653 | 0.0006195 | 0.0209402 | 0.0162811 | BCL2/SOX4/MDM4/CNOT4/FOXN3/OVOL1/CDKN1B/CHEK1/PTEN/CCND1/KANK2/CARM1/FZR1/MRNIP | 14 |
| BP | GO:0010948 | negative regulation of cell cycle process | 17/350 | 350/17653 | 0.0006563 | 0.0219878 | 0.0170956 | CDK4/BCL2/SOX4/MDM4/CBX3/CNOT4/FOXN3/OVOL1/CDKN1B/CHEK1/PTEN/CCND1/KANK2/TERF2/CARM1/FZR1/MRNIP | 17 |
| BP | GO:0045930 | negative regulation of mitotic cell cycle | 16/350 | 319/17653 | 0.0006672 | 0.0219878 | 0.0170956 | BCL2/SOX4/MDM4/CNOT4/FOXC1/FOXN3/OVOL1/CDKN1B/GAS1/CHEK1/PTEN/CCND1/KANK2/BCL2L1/CARM1/MRNIP | 16 |
| BP | GO:0003229 | ventricular cardiac muscle tissue development | 6/350 | 54/17653 | 0.000674 | 0.0219878 | 0.0170956 | NOTCH1/SMAD4/FOXC1/BMPR1A/ZFPM2/PPP1R13L | 6 |
| BP | GO:0035924 | cellular response to vascular endothelial growth factor stimulus | 6/350 | 54/17653 | 0.000674 | 0.0219878 | 0.0170956 | NOTCH1/PDGFRA/PDGFRB/FOXC1/GAS1/AKT1 | 6 |
| BP | GO:0002696 | positive regulation of leukocyte activation | 17/350 | 351/17653 | 0.0006775 | 0.0219878 | 0.0170956 | MYB/CAV1/BCL2/NFATC2/ZAP70/UNG/IGF1/TGFBR2/EFNB1/THY1/IL6ST/IL7R/VAV3/DUSP10/TNFRSF13C/AKT1/AXL | 17 |
| BP | GO:0048010 | vascular endothelial growth factor receptor signaling pathway | 8/350 | 97/17653 | 0.0006879 | 0.0219878 | 0.0170956 | HSP90AA1/FOXC1/VAV3/ITGB3/BCAR1/PRKCB/AXL/PXN | 8 |
| BP | GO:2000377 | regulation of reactive oxygen species metabolic process | 11/350 | 173/17653 | 0.0006881 | 0.0219878 | 0.0170956 | CAV1/BCL2/MYCN/MAPT/PDGFRB/TGFBR2/HSP90AA1/SELENOS/PDGFB/AKT1/SNCA | 11 |
| BP | GO:0003151 | outflow tract morphogenesis | 7/350 | 75/17653 | 0.0007085 | 0.022464 | 0.0174659 | SMAD4/TGFBR2/TBX20/FOXC1/HAND2/BMPR1A/ZFPM2 | 7 |
| BP | GO:0009408 | response to heat | 11/350 | 174/17653 | 0.0007218 | 0.0227103 | 0.0176574 | MAPT/IGF1/HSPA1B/HSP90AA1/POM121C/HSPA13/IER5/AKT1/AKT1S1/TRPV2/RPA1 | 11 |
| BP | GO:0048762 | mesenchymal cell differentiation | 12/350 | 202/17653 | 0.0007293 | 0.0227663 | 0.0177009 | BCL2/NOTCH1/SNAI1/SMAD4/TGFBR2/FOXC1/EFNB1/DAB2/HAND2/VASN/BMPR1A/PTEN | 12 |
| BP | GO:0032091 | negative regulation of protein binding | 8/350 | 98/17653 | 0.0007364 | 0.0227663 | 0.0177009 | CAV1/MET/DAB2/B2M/GNL3L/PDGFB/AKT1/CARM1 | 8 |
| BP | GO:0030335 | positive regulation of cell migration | 21/350 | 486/17653 | 0.0007403 | 0.0227663 | 0.0177009 | AKT2/MET/BCL2/NOTCH1/SNAI1/PDGFRA/PDGFRB/IGF1/TGFBR2/AGO2/IL6R/DAB2/FAM83H/THY1/PODXL/CSF1/ITGB3/BCAR1/PDGFB/PLPP3/AKT1 | 21 |
| BP | GO:0032881 | regulation of polysaccharide metabolic process | 5/350 | 37/17653 | 0.0007704 | 0.0234556 | 0.0182369 | AKT2/IGF1/SELENOS/PDGFB/AKT1 | 5 |
| BP | GO:1902807 | negative regulation of cell cycle G1/S phase transition | 9/350 | 123/17653 | 0.0007743 | 0.0234556 | 0.0182369 | BCL2/SOX4/MDM4/CNOT4/CDKN1B/PTEN/CCND1/KANK2/CARM1 | 9 |
| BP | GO:0034620 | cellular response to unfolded protein | 10/350 | 149/17653 | 0.000789 | 0.0234556 | 0.0182369 | HSPA1B/EDEM3/FICD/SELENOS/TATDN2/HSPA13/SERP1/CCND1/SEC61A1/TPP1 | 10 |
| BP | GO:0006469 | negative regulation of protein kinase activity | 14/350 | 263/17653 | 0.0008064 | 0.0234556 | 0.0182369 | CAV1/CDK4/CDK6/SPRED1/GMFB/PTPRJ/THY1/CDKN1B/DUSP10/PKIA/AKT1/PTEN/AKT1S1/MLLT1 | 14 |
| BP | GO:0051057 | positive regulation of small GTPase mediated signal transduction | 6/350 | 56/17653 | 0.0008197 | 0.0234556 | 0.0182369 | NOTCH1/PDGFRB/IGF1/SHOC2/CSF1/ADCYAP1R1 | 6 |
| BP | GO:0031100 | animal organ regeneration | 7/350 | 77/17653 | 0.00083 | 0.0234556 | 0.0182369 | CDK4/NOTCH1/TGFBR2/UCP2/CCND1/AXL/PKM | 7 |
| BP | GO:0003183 | mitral valve morphogenesis | 3/350 | 10/17653 | 0.0008361 | 0.0234556 | 0.0182369 | NOTCH1/SOX4/BMPR1A | 3 |
| BP | GO:0003211 | cardiac ventricle formation | 3/350 | 10/17653 | 0.0008361 | 0.0234556 | 0.0182369 | NOTCH1/SOX4/HAND2 | 3 |
| BP | GO:0045792 | negative regulation of cell size | 3/350 | 10/17653 | 0.0008361 | 0.0234556 | 0.0182369 | AKT1/PTEN/AKT1S1 | 3 |
| BP | GO:0072537 | fibroblast activation | 3/350 | 10/17653 | 0.0008361 | 0.0234556 | 0.0182369 | MYB/PDGFRB/CYGB | 3 |
| BP | GO:2000586 | regulation of platelet-derived growth factor receptor-beta signaling pathway | 3/350 | 10/17653 | 0.0008361 | 0.0234556 | 0.0182369 | PDGFRA/PDGFRB/PDGFB | 3 |
| BP | GO:0046677 | response to antibiotic | 16/350 | 326/17653 | 0.0008407 | 0.0234556 | 0.0182369 | MYB/MET/BCL2/PDGFRB/TXNIP/HSP90AA1/FOSL1/LDHA/CDKN1B/ADCYAP1R1/PDGFB/PTEN/CCND1/AXL/BCL2L1/CBX8 | 16 |
| BP | GO:0003279 | cardiac septum development | 8/350 | 100/17653 | 0.0008416 | 0.0234556 | 0.0182369 | NOTCH1/SOX4/DCTN5/SMAD4/TGFBR2/TBX20/BMPR1A/ZFPM2 | 8 |
| BP | GO:0032535 | regulation of cellular component size | 17/350 | 358/17653 | 0.0008432 | 0.0234556 | 0.0182369 | CDK4/MAPT/RARG/EZR/GMFB/ALOX15/IL7R/LRRC8A/VAV3/AKT1/PTEN/AKT1S1/KANK2/CRABP2/SHANK3/TRPV2/RND2 | 17 |
| BP | GO:0044843 | cell cycle G1/S phase transition | 15/350 | 295/17653 | 0.0008488 | 0.0234556 | 0.0182369 | CDK4/CCNE2/BCL2/MYC/CDK6/SOX4/MDM4/CNOT4/CDKN1B/AKT1/PTEN/CCND1/KANK2/CARM1/RPA1 | 15 |
| BP | GO:0014904 | myotube cell development | 5/350 | 38/17653 | 0.0008729 | 0.0237512 | 0.0184667 | BCL2/NFATC2/IGF1/BIN3/SKI | 5 |
| BP | GO:0061614 | pri-miRNA transcription by RNA polymerase II | 5/350 | 38/17653 | 0.0008729 | 0.0237512 | 0.0184667 | YY1/SMAD4/FOSL1/BMPR1A/PDGFB | 5 |
| BP | GO:0043255 | regulation of carbohydrate biosynthetic process | 7/350 | 78/17653 | 0.0008966 | 0.0237512 | 0.0184667 | AKT2/IGF1/SELENOS/ADCYAP1R1/PDGFB/AKT1/SNCA | 7 |
| BP | GO:0072401 | signal transduction involved in DNA integrity checkpoint | 7/350 | 78/17653 | 0.0008966 | 0.0237512 | 0.0184667 | SOX4/MDM4/CNOT4/CDKN1B/CHEK1/CARM1/FZR1 | 7 |
| BP | GO:0072422 | signal transduction involved in DNA damage checkpoint | 7/350 | 78/17653 | 0.0008966 | 0.0237512 | 0.0184667 | SOX4/MDM4/CNOT4/CDKN1B/CHEK1/CARM1/FZR1 | 7 |
| BP | GO:1900034 | regulation of cellular response to heat | 7/350 | 78/17653 | 0.0008966 | 0.0237512 | 0.0184667 | MAPT/HSPA1B/HSP90AA1/POM121C/IER5/AKT1S1/RPA1 | 7 |
| BP | GO:0009896 | positive regulation of catabolic process | 18/350 | 393/17653 | 0.0009072 | 0.0237512 | 0.0184667 | AKT2/CAV1/MTDH/IGF1/HSPA1B/AGO2/HSP90AA1/EZR/PABPC1/DAB2/SESN2/CDKN1B/CPEB3/AKT1/PTEN/SNCA/CDC37/FZR1 | 18 |
| BP | GO:0043010 | camera-type eye development | 15/350 | 297/17653 | 0.0009088 | 0.0237512 | 0.0184667 | CDK4/PDGFRA/PDGFRB/YY1/RARG/TGFBR2/FOXC1/THY1/CDKN1B/TGIF2/PPP1R13L/FKBP8/FZR1/RAB11FIP4/SKI | 15 |
| BP | GO:0006986 | response to unfolded protein | 11/350 | 179/17653 | 0.0009117 | 0.0237512 | 0.0184667 | HSPA1B/HSP90AA1/EDEM3/FICD/SELENOS/TATDN2/HSPA13/SERP1/CCND1/SEC61A1/TPP1 | 11 |
| BP | GO:0048754 | branching morphogenesis of an epithelial tube | 10/350 | 153/17653 | 0.0009673 | 0.0247276 | 0.0192258 | MET/BCL2/MYC/NOTCH4/MYCN/NOTCH1/SMAD4/TGFBR2/TBX20/CSF1 | 10 |
| BP | GO:0007492 | endoderm development | 7/350 | 79/17653 | 0.0009673 | 0.0247276 | 0.0192258 | SOX2/NANOG/NOTCH1/SOX7/SMAD4/TBX20/BMPR1A | 7 |
| BP | GO:0072395 | signal transduction involved in cell cycle checkpoint | 7/350 | 79/17653 | 0.0009673 | 0.0247276 | 0.0192258 | SOX4/MDM4/CNOT4/CDKN1B/CHEK1/CARM1/FZR1 | 7 |
| BP | GO:0032768 | regulation of monooxygenase activity | 6/350 | 58/17653 | 0.0009886 | 0.0250441 | 0.019472 | CAV1/HSP90AA1/CYGB/AKT1/SNCA/TERF2 | 6 |
| BP | GO:0072012 | glomerulus vasculature development | 4/350 | 23/17653 | 0.0009981 | 0.0250441 | 0.019472 | NOTCH1/PDGFRA/PDGFRB/PDGFB | 4 |
| BP | GO:1901522 | positive regulation of transcription from RNA polymerase II promoter involved in cellular response to chemical stimulus | 4/350 | 23/17653 | 0.0009981 | 0.0250441 | 0.019472 | NOTCH1/RUNX2/SMAD4/SESN2 | 4 |
| BP | GO:0048608 | reproductive structure development | 19/350 | 430/17653 | 0.0010086 | 0.0251535 | 0.019557 | BCL2/NOTCH1/SNAI1/PDGFRA/PDGFRB/RARG/SMAD4/FOSL1/MAP2K1/FOXC1/CDKN1B/CSDE1/ZFPM2/PDGFB/AKT1/PTEN/CCND1/AXL/BCL2L1 | 19 |
| BP | GO:0061458 | reproductive system development | 19/350 | 433/17653 | 0.001094 | 0.0270232 | 0.0210107 | BCL2/NOTCH1/SNAI1/PDGFRA/PDGFRB/RARG/SMAD4/FOSL1/MAP2K1/FOXC1/CDKN1B/CSDE1/ZFPM2/PDGFB/AKT1/PTEN/CCND1/AXL/BCL2L1 | 19 |
| BP | GO:0060412 | ventricular septum morphogenesis | 5/350 | 40/17653 | 0.0011079 | 0.0270232 | 0.0210107 | NOTCH1/SOX4/SMAD4/TGFBR2/ZFPM2 | 5 |
| BP | GO:0061647 | histone H3-K9 modification | 5/350 | 40/17653 | 0.0011079 | 0.0270232 | 0.0210107 | MYB/SMAD4/PAX5/CHEK1/JARID2 | 5 |
| BP | GO:0031331 | positive regulation of cellular catabolic process | 16/350 | 335/17653 | 0.001119 | 0.0270232 | 0.0210107 | AKT2/CAV1/MTDH/IGF1/HSPA1B/AGO2/HSP90AA1/EZR/PABPC1/DAB2/SESN2/CPEB3/AKT1/PTEN/SNCA/CDC37 | 16 |
| BP | GO:0003174 | mitral valve development | 3/350 | 11/17653 | 0.0011328 | 0.0270232 | 0.0210107 | NOTCH1/SOX4/BMPR1A | 3 |
| BP | GO:0060211 | regulation of nuclear-transcribed mRNA poly(A) tail shortening | 3/350 | 11/17653 | 0.0011328 | 0.0270232 | 0.0210107 | AGO2/PABPC1/CPEB3 | 3 |
| BP | GO:0060213 | positive regulation of nuclear-transcribed mRNA poly(A) tail shortening | 3/350 | 11/17653 | 0.0011328 | 0.0270232 | 0.0210107 | AGO2/PABPC1/CPEB3 | 3 |
| BP | GO:0051216 | cartilage development | 11/350 | 184/17653 | 0.0011412 | 0.0270232 | 0.0210107 | MYCN/SNAI1/RARG/RUNX2/TGFBR2/HAND2/LNPK/SATB2/BMP3/BMPR1A/CARM1 | 11 |
| BP | GO:0007178 | transmembrane receptor protein serine/threonine kinase signaling pathway | 16/350 | 336/17653 | 0.0011543 | 0.0270232 | 0.0210107 | CAV1/HNF4A/NOTCH1/RUNX2/SMAD4/TGFBR2/TBX20/DAB2/PEG10/BMP3/VASN/BMPR1A/TGIF2/PXN/FKBP8/SKI | 16 |
| BP | GO:0007548 | sex differentiation | 14/350 | 273/17653 | 0.0011544 | 0.0270232 | 0.0210107 | BCL2/HNF4A/PDGFRA/PDGFRB/SMAD4/CDKL2/FOXC1/CSDE1/BMPR1A/ADCYAP1R1/ZFPM2/CCND1/AXL/BCL2L1 | 14 |
| BP | GO:0050870 | positive regulation of T cell activation | 12/350 | 213/17653 | 0.0011563 | 0.0270232 | 0.0210107 | MYB/CAV1/ZAP70/IGF1/TGFBR2/EFNB1/THY1/IL6ST/IL7R/DUSP10/TNFRSF13C/AKT1 | 12 |
| BP | GO:0045926 | negative regulation of growth | 13/350 | 244/17653 | 0.0012174 | 0.0282905 | 0.021996 | BCL2/HNF4A/NOTCH1/HSPA1B/SMAD4/TGFBR2/RAI1/PTPRJ/SESN2/CDKN1B/DUSP10/JARID2/PTEN | 13 |
| BP | GO:0050671 | positive regulation of lymphocyte proliferation | 9/350 | 132/17653 | 0.0012787 | 0.0294392 | 0.0228892 | BCL2/NFATC2/ZAP70/IGF1/TGFBR2/EFNB1/IL6ST/VAV3/TNFRSF13C | 9 |
| BP | GO:0045844 | positive regulation of striated muscle tissue development | 7/350 | 83/17653 | 0.0012954 | 0.0294392 | 0.0228892 | BCL2/NOTCH1/IGF1/TBX20/MKL2/BMPR1A/ZFPM2 | 7 |
| BP | GO:0048636 | positive regulation of muscle organ development | 7/350 | 83/17653 | 0.0012954 | 0.0294392 | 0.0228892 | BCL2/NOTCH1/IGF1/TBX20/MKL2/BMPR1A/ZFPM2 | 7 |
| BP | GO:0000077 | DNA damage checkpoint | 10/350 | 159/17653 | 0.0012956 | 0.0294392 | 0.0228892 | SOX4/MDM4/CNOT4/FOXN3/CDKN1B/CHEK1/CCND1/CARM1/FZR1/MRNIP | 10 |
| BP | GO:0044773 | mitotic DNA damage checkpoint | 8/350 | 107/17653 | 0.0013083 | 0.0295635 | 0.0229858 | SOX4/MDM4/CNOT4/FOXN3/CDKN1B/CCND1/CARM1/MRNIP | 8 |
| BP | GO:0051348 | negative regulation of transferase activity | 15/350 | 309/17653 | 0.0013498 | 0.0303345 | 0.0235852 | CAV1/CDK4/CDK6/MAPT/SPRED1/GMFB/PTPRJ/THY1/CDKN1B/DUSP10/PKIA/AKT1/PTEN/AKT1S1/MLLT1 | 15 |
| BP | GO:0061437 | renal system vasculature development | 4/350 | 25/17653 | 0.001382 | 0.0305552 | 0.0237568 | NOTCH1/PDGFRA/PDGFRB/PDGFB | 4 |
| BP | GO:0061440 | kidney vasculature development | 4/350 | 25/17653 | 0.001382 | 0.0305552 | 0.0237568 | NOTCH1/PDGFRA/PDGFRB/PDGFB | 4 |
| BP | GO:0051928 | positive regulation of calcium ion transport | 8/350 | 108/17653 | 0.0013891 | 0.0305552 | 0.0237568 | CAV1/PDGFRB/EHD3/THY1/ADCYAP1R1/PDGFB/SNCA/TRPV2 | 8 |
| BP | GO:1901863 | positive regulation of muscle tissue development | 7/350 | 84/17653 | 0.0013896 | 0.0305552 | 0.0237568 | BCL2/NOTCH1/IGF1/TBX20/MKL2/BMPR1A/ZFPM2 | 7 |
| BP | GO:0070372 | regulation of ERK1 and ERK2 cascade | 15/350 | 311/17653 | 0.0014385 | 0.0314621 | 0.024462 | MET/NOTCH1/PDGFRA/PDGFRB/CCL22/SPRED1/SMAD4/EZR/MAP2K1/ALOX15/HAND2/DUSP10/PDGFB/PTEN/AXL | 15 |
| BP | GO:0051402 | neuron apoptotic process | 12/350 | 219/17653 | 0.0014655 | 0.0318824 | 0.0247888 | AKT2/MYB/BCL2/FAM162A/BARHL1/EN1/STAMBP/AXL/SNCA/AKT1S1/BCL2L1/SIGMAR1 | 12 |
| BP | GO:0031056 | regulation of histone modification | 9/350 | 136/17653 | 0.0015748 | 0.0337273 | 0.0262232 | MYB/SMAD4/PHF19/PAX5/CHEK1/JARID2/SNCA/SETD5/SKI | 9 |
| BP | GO:0006914 | autophagy | 20/350 | 482/17653 | 0.0015976 | 0.0337273 | 0.0262232 | MET/BCL2/MAPT/MTDH/BMF/UBXN2B/HSP90AA1/ITPR1/TRIM5/SESN2/VPS37B/RRAGD/ATG2A/VPS37D/TPCN2/AKT1/SNCA/CDC37/WDR45B/ATP6V1E1 | 20 |
| BP | GO:0061919 | process utilizing autophagic mechanism | 20/350 | 482/17653 | 0.0015976 | 0.0337273 | 0.0262232 | MET/BCL2/MAPT/MTDH/BMF/UBXN2B/HSP90AA1/ITPR1/TRIM5/SESN2/VPS37B/RRAGD/ATG2A/VPS37D/TPCN2/AKT1/SNCA/CDC37/WDR45B/ATP6V1E1 | 20 |
| BP | GO:0005979 | regulation of glycogen biosynthetic process | 4/350 | 26/17653 | 0.001608 | 0.0337273 | 0.0262232 | AKT2/IGF1/SELENOS/AKT1 | 4 |
| BP | GO:0010962 | regulation of glucan biosynthetic process | 4/350 | 26/17653 | 0.001608 | 0.0337273 | 0.0262232 | AKT2/IGF1/SELENOS/AKT1 | 4 |
| BP | GO:0031116 | positive regulation of microtubule polymerization | 4/350 | 26/17653 | 0.001608 | 0.0337273 | 0.0262232 | MET/MAPT/HSPA1B/CDKN1B | 4 |
| BP | GO:0090344 | negative regulation of cell aging | 4/350 | 26/17653 | 0.001608 | 0.0337273 | 0.0262232 | CDK6/PTEN/TERF2/FZR1 | 4 |
| BP | GO:0043491 | protein kinase B signaling | 13/350 | 252/17653 | 0.0016255 | 0.0339207 | 0.0263736 | MET/MTDH/PDGFRA/PDGFRB/IGF1/PTPRJ/SESN2/CDKN1B/PDGFB/AKT1/PTEN/AXL/AKT1S1 | 13 |
| BP | GO:0007569 | cell aging | 8/350 | 111/17653 | 0.0016556 | 0.0343727 | 0.026725 | BCL2/CDK6/MAP2K1/BMPR1A/CHEK1/PTEN/TERF2/FZR1 | 8 |
| BP | GO:0001655 | urogenital system development | 15/350 | 316/17653 | 0.0016817 | 0.0346846 | 0.0269675 | BCL2/MYC/NOTCH1/PDGFRA/PDGFRB/RARG/SOX4/SMAD4/FOXC1/OVOL1/CDKN1B/PODXL/PDGFB/PTEN/SEC61A1 | 15 |
| BP | GO:1903706 | regulation of hemopoiesis | 19/350 | 450/17653 | 0.0017042 | 0.0346846 | 0.0269675 | MYB/MYC/CDK6/NFATC2/ZAP70/NOTCH1/RARG/HSPA1B/TNRC6A/TGFBR2/FOXC1/KMT2D/B2M/ATXN1L/IL7R/CSF1/DUSP10/PRKCB/AXL | 19 |
| BP | GO:0048644 | muscle organ morphogenesis | 7/350 | 87/17653 | 0.0017045 | 0.0346846 | 0.0269675 | NOTCH1/SMAD4/TBX20/FOXC1/SERP1/BMPR1A/ZFPM2 | 7 |
| BP | GO:0060021 | roof of mouth development | 7/350 | 87/17653 | 0.0017045 | 0.0346846 | 0.0269675 | SNAI1/SMAD4/TGFBR2/HAND2/SATB2/BMPR1A/SKI | 7 |
| BP | GO:0031113 | regulation of microtubule polymerization | 5/350 | 44/17653 | 0.0017141 | 0.0347069 | 0.0269848 | MET/MAPT/HSPA1B/CDKN1B/SNCA | 5 |
| BP | GO:0042542 | response to hydrogen peroxide | 9/350 | 138/17653 | 0.0017422 | 0.0349577 | 0.0271798 | MYB/MET/BCL2/PDGFRB/TXNIP/FOSL1/LDHA/AXL/CBX8 | 9 |
| BP | GO:0032868 | response to insulin | 13/350 | 254/17653 | 0.0017436 | 0.0349577 | 0.0271798 | AKT2/CDK4/INPPL1/IGF1/UCP2/SESN2/SELENOS/BCAR1/AKT1/PRKCB/PTEN/PKM/ATP6V1E1 | 13 |
| BP | GO:1903039 | positive regulation of leukocyte cell-cell adhesion | 12/350 | 224/17653 | 0.0017727 | 0.0351645 | 0.0273406 | MYB/CAV1/ZAP70/IGF1/TGFBR2/EFNB1/THY1/IL6ST/IL7R/DUSP10/TNFRSF13C/AKT1 | 12 |
| BP | GO:0034599 | cellular response to oxidative stress | 14/350 | 286/17653 | 0.0017877 | 0.0351645 | 0.0273406 | MYB/MET/BCL2/MAPT/PDGFRA/HSPA1B/SESN2/SELENOS/AKT1/AXL/SNCA/PXN/CBX8/NFE2L1 | 14 |
| BP | GO:0035967 | cellular response to topologically incorrect protein | 10/350 | 166/17653 | 0.0017879 | 0.0351645 | 0.0273406 | HSPA1B/EDEM3/FICD/SELENOS/TATDN2/HSPA13/SERP1/CCND1/SEC61A1/TPP1 | 10 |
| BP | GO:0042733 | embryonic digit morphogenesis | 6/350 | 65/17653 | 0.0017969 | 0.0351645 | 0.0273406 | MYCN/NOTCH1/SMAD4/HAND2/LNPK/BMPR1A | 6 |
| BP | GO:1904888 | cranial skeletal system development | 6/350 | 65/17653 | 0.0017969 | 0.0351645 | 0.0273406 | PDGFRA/RUNX2/TGFBR2/FOXN3/PAX5/MTHFD1 | 6 |
| BP | GO:0090092 | regulation of transmembrane receptor protein serine/threonine kinase signaling pathway | 12/350 | 225/17653 | 0.0018401 | 0.0356044 | 0.0276826 | CAV1/NOTCH1/SMAD4/TGFBR2/TBX20/DAB2/PEG10/BMP3/VASN/BMPR1A/FKBP8/SKI | 12 |
| BP | GO:0044774 | mitotic DNA integrity checkpoint | 8/350 | 113/17653 | 0.0018546 | 0.0356044 | 0.0276826 | SOX4/MDM4/CNOT4/FOXN3/CDKN1B/CCND1/CARM1/MRNIP | 8 |
| BP | GO:0071156 | regulation of cell cycle arrest | 8/350 | 113/17653 | 0.0018546 | 0.0356044 | 0.0276826 | AKT2/CDK4/SOX4/MDM4/CNOT4/CDKN1B/CCND1/CARM1 | 8 |
| BP | GO:0042026 | protein refolding | 4/350 | 27/17653 | 0.0018585 | 0.0356044 | 0.0276826 | HSPA1B/HSP90AA1/B2M/HSPA13 | 4 |
| BP | GO:1901987 | regulation of cell cycle phase transition | 19/350 | 454/17653 | 0.0018838 | 0.0356044 | 0.0276826 | CDK4/BCL2/CDK6/SOX4/MDM4/HSP90AA1/CNOT4/FOXN3/OVOL1/CDKN1B/CHEK1/PKIA/AKT1/PTEN/CCND1/KANK2/CARM1/FZR1/MRNIP | 19 |
| BP | GO:0048701 | embryonic cranial skeleton morphogenesis | 5/350 | 45/17653 | 0.0018975 | 0.0356044 | 0.0276826 | PDGFRA/RUNX2/TGFBR2/PAX5/MTHFD1 | 5 |
| BP | GO:0055023 | positive regulation of cardiac muscle tissue growth | 5/350 | 45/17653 | 0.0018975 | 0.0356044 | 0.0276826 | NOTCH1/IGF1/TBX20/BMPR1A/ZFPM2 | 5 |
| BP | GO:0032530 | regulation of microvillus organization | 3/350 | 13/17653 | 0.0019064 | 0.0356044 | 0.0276826 | EZR/PODXL/FSCN1 | 3 |
| BP | GO:0035791 | platelet-derived growth factor receptor-beta signaling pathway | 3/350 | 13/17653 | 0.0019064 | 0.0356044 | 0.0276826 | PDGFRA/PDGFRB/PDGFB | 3 |
| BP | GO:0060391 | positive regulation of SMAD protein signal transduction | 3/350 | 13/17653 | 0.0019064 | 0.0356044 | 0.0276826 | SMAD4/DAB2/BMPR1A | 3 |
| BP | GO:2000241 | regulation of reproductive process | 9/350 | 140/17653 | 0.0019237 | 0.0357624 | 0.0278055 | NOTCH1/SNAI1/IGF1/AGO2/OVOL1/CDKN1B/CCDC36/ZFPM2/FZR1 | 9 |
| BP | GO:0060395 | SMAD protein signal transduction | 6/350 | 66/17653 | 0.0019442 | 0.0359817 | 0.027976 | HNF4A/SMAD4/DAB2/BMP3/BMPR1A/SKI | 6 |
| BP | GO:0090066 | regulation of anatomical structure size | 20/350 | 491/17653 | 0.0019836 | 0.0365455 | 0.0284143 | CAV1/CDK4/MAPT/RARG/EZR/GMFB/ALOX15/FOXC1/IL7R/LRRC8A/VAV3/KCNMB4/AKT1/PTEN/AKT1S1/KANK2/CRABP2/SHANK3/TRPV2/RND2 | 20 |
| BP | GO:0031570 | DNA integrity checkpoint | 10/350 | 169/17653 | 0.0020406 | 0.0373893 | 0.0290704 | SOX4/MDM4/CNOT4/FOXN3/CDKN1B/CHEK1/CCND1/CARM1/FZR1/MRNIP | 10 |
| BP | GO:0035966 | response to topologically incorrect protein | 11/350 | 198/17653 | 0.0020477 | 0.0373893 | 0.0290704 | HSPA1B/HSP90AA1/EDEM3/FICD/SELENOS/TATDN2/HSPA13/SERP1/CCND1/SEC61A1/TPP1 | 11 |
| BP | GO:0060840 | artery development | 7/350 | 90/17653 | 0.0020724 | 0.0376719 | 0.0292901 | NOTCH1/PDGFRB/SOX4/DCTN5/FOXC1/HAND2/BMPR1A | 7 |
| BP | GO:0003281 | ventricular septum development | 6/350 | 67/17653 | 0.0021005 | 0.0376806 | 0.0292969 | NOTCH1/SOX4/DCTN5/SMAD4/TGFBR2/ZFPM2 | 6 |
| BP | GO:0040014 | regulation of multicellular organism growth | 6/350 | 67/17653 | 0.0021005 | 0.0376806 | 0.0292969 | CDK4/BCL2/IGF1/EZR/RAI1/CSF1 | 6 |
| BP | GO:0051705 | multi-organism behavior | 6/350 | 67/17653 | 0.0021005 | 0.0376806 | 0.0292969 | ATXN1L/HAND2/EN1/NLGN4X/PTEN/SHANK3 | 6 |
| BP | GO:1903708 | positive regulation of hemopoiesis | 10/350 | 170/17653 | 0.002131 | 0.0377969 | 0.0293873 | MYB/ZAP70/HSPA1B/TGFBR2/FOXC1/ATXN1L/IL7R/CSF1/DUSP10/AXL | 10 |
| BP | GO:0002053 | positive regulation of mesenchymal cell proliferation | 4/350 | 28/17653 | 0.0021347 | 0.0377969 | 0.0293873 | MYC/MYCN/TGFBR2/BMPR1A | 4 |
| BP | GO:0061311 | cell surface receptor signaling pathway involved in heart development | 4/350 | 28/17653 | 0.0021347 | 0.0377969 | 0.0293873 | NOTCH1/SNAI1/HAND2/BMPR1A | 4 |
| BP | GO:0046660 | female sex differentiation | 8/350 | 116/17653 | 0.0021881 | 0.0385747 | 0.0299921 | BCL2/PDGFRA/SMAD4/FOXC1/ADCYAP1R1/ZFPM2/AXL/BCL2L1 | 8 |
| BP | GO:0006767 | water-soluble vitamin metabolic process | 7/350 | 91/17653 | 0.0022078 | 0.0385888 | 0.030003 | SLC5A6/SHMT1/PM20D2/AASDHPPT/PNPO/MTHFD1/PDXK | 7 |
| BP | GO:0009791 | post-embryonic development | 7/350 | 91/17653 | 0.0022078 | 0.0385888 | 0.030003 | BCL2/INPPL1/AGO2/ITPR1/SERP1/PPP1R13L/PLAGL2 | 7 |
| BP | GO:0048660 | regulation of smooth muscle cell proliferation | 9/350 | 143/17653 | 0.0022237 | 0.0387025 | 0.0300915 | MYB/PDGFRB/IGF1/TGFBR2/IL6R/CDKN1B/BMPR1A/PDGFB/AKT1 | 9 |
| BP | GO:0045913 | positive regulation of carbohydrate metabolic process | 6/350 | 68/17653 | 0.0022661 | 0.0391073 | 0.0304062 | AKT2/IGF1/ADCYAP1R1/PDGFB/AKT1/SNCA | 6 |
| BP | GO:0055008 | cardiac muscle tissue morphogenesis | 6/350 | 68/17653 | 0.0022661 | 0.0391073 | 0.0304062 | NOTCH1/SMAD4/TBX20/FOXC1/BMPR1A/ZFPM2 | 6 |
| BP | GO:0002064 | epithelial cell development | 11/350 | 201/17653 | 0.0023031 | 0.0391491 | 0.0304386 | MET/CDK6/NOTCH4/NOTCH1/RARG/EZR/PODXL/CLIC5/LRTOMT/PDGFB/TJP1 | 11 |
| BP | GO:0032869 | cellular response to insulin stimulus | 11/350 | 201/17653 | 0.0023031 | 0.0391491 | 0.0304386 | AKT2/CDK4/IGF1/UCP2/SELENOS/BCAR1/AKT1/PRKCB/PTEN/PKM/ATP6V1E1 | 11 |
| BP | GO:0050999 | regulation of nitric-oxide synthase activity | 5/350 | 47/17653 | 0.0023068 | 0.0391491 | 0.0304386 | CAV1/HSP90AA1/CYGB/AKT1/TERF2 | 5 |
| BP | GO:0060976 | coronary vasculature development | 5/350 | 47/17653 | 0.0023068 | 0.0391491 | 0.0304386 | NOTCH1/PDGFRB/DCTN5/SPRED1/HAND2 | 5 |
| BP | GO:2000379 | positive regulation of reactive oxygen species metabolic process | 7/350 | 92/17653 | 0.0023498 | 0.0394768 | 0.0306935 | MAPT/PDGFRB/TGFBR2/HSP90AA1/PDGFB/AKT1/SNCA | 7 |
| BP | GO:0003188 | heart valve formation | 3/350 | 14/17653 | 0.002391 | 0.0394768 | 0.0306935 | NOTCH1/SMAD4/TBX20 | 3 |
| BP | GO:0003214 | cardiac left ventricle morphogenesis | 3/350 | 14/17653 | 0.002391 | 0.0394768 | 0.0306935 | NOTCH1/SMAD4/TGFBR2 | 3 |
| BP | GO:0043217 | myelin maintenance | 3/350 | 14/17653 | 0.002391 | 0.0394768 | 0.0306935 | AKT2/AKT1/PTEN | 3 |
| BP | GO:0072109 | glomerular mesangium development | 3/350 | 14/17653 | 0.002391 | 0.0394768 | 0.0306935 | NOTCH1/PDGFRB/PDGFB | 3 |
| BP | GO:0072216 | positive regulation of metanephros development | 3/350 | 14/17653 | 0.002391 | 0.0394768 | 0.0306935 | MYC/PDGFRB/PDGFB | 3 |
| BP | GO:0032944 | regulation of mononuclear cell proliferation | 11/350 | 202/17653 | 0.0023937 | 0.0394768 | 0.0306935 | BCL2/NFATC2/ZAP70/IGF1/TGFBR2/PLA2G2D/EFNB1/IL6ST/CSF1/VAV3/TNFRSF13C | 11 |
| BP | GO:0031112 | positive regulation of microtubule polymerization or depolymerization | 4/350 | 29/17653 | 0.0024381 | 0.0397815 | 0.0309303 | MET/MAPT/HSPA1B/CDKN1B | 4 |
| BP | GO:0090200 | positive regulation of release of cytochrome c from mitochondria | 4/350 | 29/17653 | 0.0024381 | 0.0397815 | 0.0309303 | BMF/FAM162A/MOAP1/APOPT1 | 4 |
| BP | GO:0048708 | astrocyte differentiation | 6/350 | 69/17653 | 0.0024414 | 0.0397815 | 0.0309303 | CDK6/MYCN/NOTCH1/MAPT/MAP2K1/IL6ST | 6 |
| BP | GO:0014812 | muscle cell migration | 7/350 | 93/17653 | 0.0024989 | 0.0403967 | 0.0314087 | BCL2/PDGFRB/IGF1/BMPR1A/ITGB3/PDGFB/BIN3 | 7 |
| BP | GO:0048704 | embryonic skeletal system morphogenesis | 7/350 | 93/17653 | 0.0024989 | 0.0403967 | 0.0314087 | MYCN/PDGFRA/RUNX2/TGFBR2/SATB2/PAX5/MTHFD1 | 7 |
| BP | GO:0010463 | mesenchymal cell proliferation | 5/350 | 48/17653 | 0.0025339 | 0.0406422 | 0.0315995 | MYC/MYCN/TGFBR2/HAND2/BMPR1A | 5 |
| BP | GO:0055010 | ventricular cardiac muscle tissue morphogenesis | 5/350 | 48/17653 | 0.0025339 | 0.0406422 | 0.0315995 | NOTCH1/SMAD4/FOXC1/BMPR1A/ZFPM2 | 5 |
| BP | GO:0030098 | lymphocyte differentiation | 15/350 | 330/17653 | 0.002552 | 0.0407402 | 0.0316758 | MYB/BCL2/NFATC2/ZAP70/RUNX2/SOX4/TGFBR2/PLA2G2D/KLF6/B2M/IL7R/LRRC8A/DUSP10/AXL/ADGRG3 | 15 |
| BP | GO:0048659 | smooth muscle cell proliferation | 9/350 | 146/17653 | 0.00256 | 0.0407402 | 0.0316758 | MYB/PDGFRB/IGF1/TGFBR2/IL6R/CDKN1B/BMPR1A/PDGFB/AKT1 | 9 |
| BP | GO:0038034 | signal transduction in absence of ligand | 6/350 | 70/17653 | 0.0026266 | 0.0414778 | 0.0322492 | BCL2/HSPA1B/MKNK2/MOAP1/AKT1/BCL2L1 | 6 |
| BP | GO:0097192 | extrinsic apoptotic signaling pathway in absence of ligand | 6/350 | 70/17653 | 0.0026266 | 0.0414778 | 0.0322492 | BCL2/HSPA1B/MKNK2/MOAP1/AKT1/BCL2L1 | 6 |
| BP | GO:0021782 | glial cell development | 7/350 | 94/17653 | 0.0026551 | 0.041766 | 0.0324733 | AKT2/CDK6/MAPT/SOX4/AKT1/PTEN/SKI | 7 |
| BP | GO:0043552 | positive regulation of phosphatidylinositol 3-kinase activity | 4/350 | 30/17653 | 0.0027697 | 0.0430195 | 0.033448 | PDGFRA/PDGFRB/VAV3/PDGFB | 4 |
| BP | GO:0070873 | regulation of glycogen metabolic process | 4/350 | 30/17653 | 0.0027697 | 0.0430195 | 0.033448 | AKT2/IGF1/SELENOS/AKT1 | 4 |
| BP | GO:0046579 | positive regulation of Ras protein signal transduction | 5/350 | 49/17653 | 0.0027768 | 0.0430195 | 0.033448 | NOTCH1/PDGFRB/IGF1/SHOC2/CSF1 | 5 |
| BP | GO:0060421 | positive regulation of heart growth | 5/350 | 49/17653 | 0.0027768 | 0.0430195 | 0.033448 | NOTCH1/IGF1/TBX20/BMPR1A/ZFPM2 | 5 |
| BP | GO:0031571 | mitotic G1 DNA damage checkpoint | 6/350 | 71/17653 | 0.0028222 | 0.0432879 | 0.0336566 | SOX4/MDM4/CNOT4/CDKN1B/CCND1/CARM1 | 6 |
| BP | GO:0044819 | mitotic G1/S transition checkpoint | 6/350 | 71/17653 | 0.0028222 | 0.0432879 | 0.0336566 | SOX4/MDM4/CNOT4/CDKN1B/CCND1/CARM1 | 6 |
| BP | GO:0009266 | response to temperature stimulus | 12/350 | 237/17653 | 0.0028259 | 0.0432879 | 0.0336566 | MAPT/IGF1/HSPA1B/HSP90AA1/POM121C/UCP2/HSPA13/IER5/AKT1/AKT1S1/TRPV2/RPA1 | 12 |
| BP | GO:0010721 | negative regulation of cell development | 14/350 | 301/17653 | 0.0028533 | 0.0433228 | 0.0336838 | BCL2/MYCN/SOX2/NOTCH1/IGF1/SMAD4/B2M/THY1/BMPR1A/DUSP10/PTEN/ARHGDIA/CARM1/SKI | 14 |
| BP | GO:0055001 | muscle cell development | 10/350 | 177/17653 | 0.0028577 | 0.0433228 | 0.0336838 | BCL2/NFATC2/NOTCH1/PDGFRA/PDGFRB/IGF1/SMAD4/SORBS2/BIN3/SKI | 10 |
| BP | GO:0010038 | response to metal ion | 15/350 | 334/17653 | 0.0028599 | 0.0433228 | 0.0336838 | CAV1/CDK4/BCL2/MAPT/TXNIP/CDH1/ALOX15/B2M/NEUROD2/CDKN1B/KCNMB4/AKT1/PTEN/CCND1/SNCA | 15 |
| BP | GO:0045216 | cell-cell junction organization | 12/350 | 238/17653 | 0.0029244 | 0.0438016 | 0.034056 | CAV1/BCL2/MTDH/SNAI1/CDH1/PTPRJ/THY1/NLGN4X/PTEN/TJP1/FSCN1/PVR | 12 |
| BP | GO:0048872 | homeostasis of number of cells | 12/350 | 238/17653 | 0.0029244 | 0.0438016 | 0.034056 | BCL2/CDK6/HSPA1B/DYRK3/B2M/IL7R/CSF1/TNFRSF13C/MTHFD1/AKT1/AXL/NFE2L1 | 12 |
| BP | GO:0003417 | growth plate cartilage development | 3/350 | 15/17653 | 0.0029451 | 0.0438016 | 0.034056 | RARG/TGFBR2/CARM1 | 3 |
| BP | GO:0045725 | positive regulation of glycogen biosynthetic process | 3/350 | 15/17653 | 0.0029451 | 0.0438016 | 0.034056 | AKT2/IGF1/AKT1 | 3 |
| BP | GO:0072075 | metanephric mesenchyme development | 3/350 | 15/17653 | 0.0029451 | 0.0438016 | 0.034056 | MYC/PDGFRB/SMAD4 | 3 |
| BP | GO:0034330 | cell junction organization | 13/350 | 270/17653 | 0.0029678 | 0.04398 | 0.0341947 | CAV1/BCL2/MTDH/SNAI1/CDH1/PTPRJ/THY1/NLGN4X/ITGB3/PTEN/TJP1/FSCN1/PVR | 13 |
| BP | GO:0044783 | G1 DNA damage checkpoint | 6/350 | 72/17653 | 0.0030284 | 0.044555 | 0.0346418 | SOX4/MDM4/CNOT4/CDKN1B/CCND1/CARM1 | 6 |
| BP | GO:0048844 | artery morphogenesis | 6/350 | 72/17653 | 0.0030284 | 0.044555 | 0.0346418 | NOTCH1/PDGFRB/SOX4/FOXC1/HAND2/BMPR1A | 6 |
| BP | GO:0045736 | negative regulation of cyclin-dependent protein serine/threonine kinase activity | 4/350 | 31/17653 | 0.003131 | 0.0458995 | 0.0356871 | CDK4/CDK6/CDKN1B/PTEN | 4 |
| BP | GO:0072331 | signal transduction by p53 class mediator | 13/350 | 272/17653 | 0.003161 | 0.0461726 | 0.0358995 | SNAI1/SOX4/MDM4/SPRED1/CNOT4/DYRK3/SESN2/CDKN1B/CHEK1/AKT1/PPP1R13L/CARM1/RPA1 | 13 |
| BP | GO:0016049 | cell growth | 19/350 | 477/17653 | 0.0032558 | 0.0473893 | 0.0368455 | BCL2/HNF4A/MAPT/RARG/IGF1/HSPA1B/SMAD4/TGFBR2/PTPRJ/KMT2D/SESN2/CDKN1B/SORBS2/BCAR1/AKT1/BIN3/CRABP2/TRPV2/RND2 | 19 |
| BP | GO:0035176 | social behavior | 5/350 | 51/17653 | 0.0033124 | 0.0478721 | 0.0372209 | ATXN1L/EN1/NLGN4X/PTEN/SHANK3 | 5 |
| BP | GO:0051703 | intraspecies interaction between organisms | 5/350 | 51/17653 | 0.0033124 | 0.0478721 | 0.0372209 | ATXN1L/EN1/NLGN4X/PTEN/SHANK3 | 5 |
| BP | GO:0034976 | response to endoplasmic reticulum stress | 13/350 | 274/17653 | 0.0033642 | 0.0484492 | 0.0376695 | CAV1/BCL2/ITPR1/ALOX15/EDEM3/FICD/SESN2/SELENOS/TATDN2/SERP1/CCND1/SEC61A1/TPP1 | 13 |
| BP | GO:0070663 | regulation of leukocyte proliferation | 11/350 | 212/17653 | 0.0034689 | 0.0490239 | 0.0381164 | BCL2/NFATC2/ZAP70/IGF1/TGFBR2/PLA2G2D/EFNB1/IL6ST/CSF1/VAV3/TNFRSF13C | 11 |
| BP | GO:0014031 | mesenchymal cell development | 6/350 | 74/17653 | 0.0034745 | 0.0490239 | 0.0381164 | BCL2/NOTCH1/FOXC1/EFNB1/HAND2/BMPR1A | 6 |
| BP | GO:0043627 | response to estrogen | 6/350 | 74/17653 | 0.0034745 | 0.0490239 | 0.0381164 | CAV1/PDGFRB/TGFBR2/LDHA/KMT2D/CCND1 | 6 |
| BP | GO:0001704 | formation of primary germ layer | 8/350 | 125/17653 | 0.0034773 | 0.0490239 | 0.0381164 | SOX2/NANOG/SNAI1/SOX7/TBX20/FOXC1/BMPR1A/ITGB3 | 8 |
| BP | GO:0008637 | apoptotic mitochondrial changes | 8/350 | 125/17653 | 0.0034773 | 0.0490239 | 0.0381164 | BCL2/BMF/IGF1/FAM162A/MOAP1/APOPT1/AKT1/BCL2L1 | 8 |
| BP | GO:0035270 | endocrine system development | 8/350 | 125/17653 | 0.0034773 | 0.0490239 | 0.0381164 | CDK6/SOX2/SOX4/CDH1/IL6R/MAP2K1/BMPR1A/AKT1 | 8 |
| BP | GO:0043403 | skeletal muscle tissue regeneration | 4/350 | 32/17653 | 0.0035232 | 0.0490239 | 0.0381164 | IGF1/TGFBR2/BIN3/PKM | 4 |
| BP | GO:0060317 | cardiac epithelial to mesenchymal transition | 4/350 | 32/17653 | 0.0035232 | 0.0490239 | 0.0381164 | NOTCH1/SNAI1/SMAD4/TGFBR2 | 4 |
| BP | GO:0030033 | microvillus assembly | 3/350 | 16/17653 | 0.0035719 | 0.0490239 | 0.0381164 | EZR/PODXL/FSCN1 | 3 |
| BP | GO:0070875 | positive regulation of glycogen metabolic process | 3/350 | 16/17653 | 0.0035719 | 0.0490239 | 0.0381164 | AKT2/IGF1/AKT1 | 3 |
| BP | GO:0072224 | metanephric glomerulus development | 3/350 | 16/17653 | 0.0035719 | 0.0490239 | 0.0381164 | PDGFRA/PDGFRB/PDGFB | 3 |
| BP | GO:1900151 | regulation of nuclear-transcribed mRNA catabolic process, deadenylation-dependent decay | 3/350 | 16/17653 | 0.0035719 | 0.0490239 | 0.0381164 | AGO2/PABPC1/CPEB3 | 3 |
| BP | GO:1900153 | positive regulation of nuclear-transcribed mRNA catabolic process, deadenylation-dependent decay | 3/350 | 16/17653 | 0.0035719 | 0.0490239 | 0.0381164 | AGO2/PABPC1/CPEB3 | 3 |
| BP | GO:2000811 | negative regulation of anoikis | 3/350 | 16/17653 | 0.0035719 | 0.0490239 | 0.0381164 | CAV1/BCL2/NOTCH1 | 3 |
| BP | GO:0010676 | positive regulation of cellular carbohydrate metabolic process | 5/350 | 52/17653 | 0.0036064 | 0.049167 | 0.0382276 | AKT2/IGF1/ADCYAP1R1/AKT1/SNCA | 5 |
| BP | GO:0035904 | aorta development | 5/350 | 52/17653 | 0.0036064 | 0.049167 | 0.0382276 | NOTCH1/PDGFRB/SOX4/DCTN5/BMPR1A | 5 |
| BP | GO:0045787 | positive regulation of cell cycle | 16/350 | 377/17653 | 0.0036628 | 0.0497702 | 0.0386966 | CDK4/PDGFRB/IGF1/SOX4/MDM4/FOSL1/CNOT4/OVOL1/CDKN1B/CHEK1/PDGFB/AKT1/CCND1/USP22/CARM1/RAB11FIP4 | 16 |
| CC | GO:0043235 | receptor complex | 22/364 | 374/18698 | 4.59E-06 | 0.0020501 | 0.0017621 | MET/ZAP70/NOTCH1/PDGFRA/PDGFRB/TGFBR2/IL6R/IFNLR1/DAB2/B2M/GFRA1/IL6ST/ITGA11/BMPR1A/ADCYAP1R1/ITGB3/LIFR/GABRA3/GABBR2/GRM7/AXL/SHANK3 | 22 |
| CC | GO:0031252 | cell leading edge | 20/364 | 385/18698 | 7.23E-05 | 0.0115871 | 0.0099595 | AKT2/INPPL1/CDK6/MAPT/CDH1/HSP90AA1/EZR/PTPRJ/CYTH3/THY1/PODXL/OPRD1/SORBS2/ITGB3/BCAR1/MKLN1/GABRA3/PXN/FSCN1/MYO10 | 20 |
| CC | GO:0001726 | ruffle | 12/364 | 164/18698 | 9.13E-05 | 0.0115871 | 0.0099595 | AKT2/CDK6/HSP90AA1/EZR/PTPRJ/CYTH3/PODXL/ITGB3/BCAR1/MKLN1/FSCN1/MYO10 | 12 |
| CC | GO:0031965 | nuclear membrane | 17/364 | 304/18698 | 0.0001037 | 0.0115871 | 0.0099595 | CDK4/BCL2/MTDH/DCTN5/CBX3/ITPR1/TNPO2/POM121C/DDX19B/LRPPRC/TRA2B/DDX19A/ALG14/SNCA/BCL2L1/FZR1/SIGMAR1 | 17 |
| CC | GO:0098802 | plasma membrane receptor complex | 12/364 | 179/18698 | 0.0002086 | 0.0186446 | 0.0160256 | ZAP70/TGFBR2/IL6R/IFNLR1/DAB2/B2M/IL6ST/ITGA11/BMPR1A/ITGB3/GABBR2/SHANK3 | 12 |
| CC | GO:0005925 | focal adhesion | 19/364 | 393/18698 | 0.0002738 | 0.0192309 | 0.0165295 | CAV1/PDGFRB/HSPA1B/EZR/PABPC1/MAP2K1/EHD3/SLC4A2/DAB2/B2M/THY1/ITGA11/SORBS2/ITGB3/BCAR1/GIT1/PXN/PEAK3/PVR | 19 |
| CC | GO:0005924 | cell-substrate adherens junction | 19/364 | 396/18698 | 0.0003012 | 0.0192309 | 0.0165295 | CAV1/PDGFRB/HSPA1B/EZR/PABPC1/MAP2K1/EHD3/SLC4A2/DAB2/B2M/THY1/ITGA11/SORBS2/ITGB3/BCAR1/GIT1/PXN/PEAK3/PVR | 19 |
| CC | GO:0030055 | cell-substrate junction | 19/364 | 401/18698 | 0.000352 | 0.0196663 | 0.0169038 | CAV1/PDGFRB/HSPA1B/EZR/PABPC1/MAP2K1/EHD3/SLC4A2/DAB2/B2M/THY1/ITGA11/SORBS2/ITGB3/BCAR1/GIT1/PXN/PEAK3/PVR | 19 |
| MF | GO:0005161 | platelet-derived growth factor receptor binding | 6/348 | 15/17548 | 2.51E-07 | 0.0001612 | 0.0001444 | PDGFRA/PDGFRB/PTPRJ/ITGB3/PDGFB/PTEN | 6 |
| MF | GO:0000982 | transcription factor activity, RNA polymerase II proximal promoter sequence-specific DNA binding | 25/348 | 415/17548 | 9.03E-07 | 0.0002903 | 0.00026 | MYB/MYC/NFATC2/MYCN/SOX2/HNF4A/SNAI1/YY1/RUNX2/SOX4/ZBTB7A/SMAD4/FOSL1/TBX20/NFIC/KLF6/HAND2/SSBP2/BARHL1/EN1/OVOL1/SATB2/PAX5/ZFPM2/PLAGL2 | 25 |
| MF | GO:0001077 | transcriptional activator activity, RNA polymerase II proximal promoter sequence-specific DNA binding | 19/348 | 279/17548 | 3.27E-06 | 0.0005324 | 0.0004768 | MYB/MYC/NFATC2/MYCN/SOX2/HNF4A/RUNX2/SOX4/SMAD4/FOSL1/TBX20/NFIC/KLF6/HAND2/SSBP2/BARHL1/SATB2/PAX5/PLAGL2 | 19 |
| MF | GO:0001228 | transcriptional activator activity, RNA polymerase II transcription regulatory region sequence-specific DNA binding | 24/348 | 417/17548 | 3.31E-06 | 0.0005324 | 0.0004768 | MYB/E2F3/MYC/NFATC2/MYCN/SOX2/NANOG/HNF4A/RUNX2/SOX4/SMAD4/FOSL1/TBX20/FOXC1/NFIC/KLF6/HAND2/SSBP2/BARHL1/NEUROD2/SATB2/PAX5/PLAGL2/NFE2L1 | 24 |
| MF | GO:0004693 | cyclin-dependent protein serine/threonine kinase activity | 6/348 | 35/17548 | 5.82E-05 | 0.0073572 | 0.0065882 | CDK4/CDK6/CDKL2/CDKN1B/CCND1/CDK16 | 6 |
| MF | GO:0097472 | cyclin-dependent protein kinase activity | 6/348 | 36/17548 | 6.87E-05 | 0.0073572 | 0.0065882 | CDK4/CDK6/CDKL2/CDKN1B/CCND1/CDK16 | 6 |
| MF | GO:0000978 | RNA polymerase II proximal promoter sequence-specific DNA binding | 21/348 | 436/17548 | 0.0001769 | 0.0162477 | 0.0145494 | MYB/MYC/NFATC2/MYCN/HNF4A/YY1/RUNX2/ZBTB7A/SMAD4/FOSL1/TBX20/NFIC/KLF6/HAND2/SSBP2/NEUROD2/EN1/OVOL1/SATB2/HNRNPC/PAX5 | 21 |
| MF | GO:0000987 | proximal promoter sequence-specific DNA binding | 21/348 | 451/17548 | 0.0002795 | 0.021045 | 0.0188452 | MYB/MYC/NFATC2/MYCN/HNF4A/YY1/RUNX2/ZBTB7A/SMAD4/FOSL1/TBX20/NFIC/KLF6/HAND2/SSBP2/NEUROD2/EN1/OVOL1/SATB2/HNRNPC/PAX5 | 21 |
| MF | GO:0001190 | transcriptional activator activity, RNA polymerase II transcription factor binding | 7/348 | 65/17548 | 0.0002946 | 0.021045 | 0.0188452 | NOTCH1/SOX4/TBX20/MKL2/ZFPM2/CARM1/NFE2L1 | 7 |
| MF | GO:0001076 | transcription factor activity, RNA polymerase II transcription factor binding | 12/348 | 191/17548 | 0.0004438 | 0.0285359 | 0.0255531 | MYB/NOTCH1/YY1/RARG/SOX4/SMAD4/TBX20/MED4/MKL2/ZFPM2/CARM1/NFE2L1 | 12 |
| MF | GO:0019902 | phosphatase binding | 11/348 | 168/17548 | 0.0005393 | 0.0302002 | 0.0270434 | MET/BCL2/NFATC2/MAPT/SPRED1/SHOC2/CDKN1B/AKT1/PPP6R1/PTPA/PPP1R11 | 11 |
| MF | GO:0005057 | signal transducer activity, downstream of receptor | 11/348 | 169/17548 | 0.0005669 | 0.0302002 | 0.0270434 | MET/PDGFRA/PDGFRB/SMAD4/TGFBR2/MAP2K1/MAP3K9/CDKN1B/BMPR1A/MAP3K2/AXL | 11 |
| MF | GO:0019903 | protein phosphatase binding | 9/348 | 119/17548 | 0.0006106 | 0.0302002 | 0.0270434 | MET/BCL2/MAPT/SHOC2/CDKN1B/AKT1/PPP6R1/PTPA/PPP1R11 | 9 |

KEGG pathways

| ID | Description | GeneRatio | BgRatio | pvalue | p.adjust | qvalue | geneID | Count |
| --- | --- | --- | --- | --- | --- | --- | --- | --- |
| hsa05218 | Melanoma | 14/204 | 72/8063 | 2.32E-09 | 5.24E-07 | 3.76E-07 | 208/4233/1871/1019/1021/5156/5159/3479/999/5604/5155/207/5728/595 | 14 |
| hsa05214 | Glioma | 14/204 | 75/8063 | 4.06E-09 | 5.24E-07 | 3.76E-07 | 208/1871/1019/1021/5156/5159/3479/5604/91860/5155/207/5579/5728/595 | 14 |
| hsa01521 | EGFR tyrosine kinase inhibitor resistance | 14/204 | 79/8063 | 8.25E-09 | 7.09E-07 | 5.09E-07 | 208/4233/596/5156/5159/3479/3570/5604/5155/207/5579/5728/558/598 | 14 |
| hsa05215 | Prostate cancer | 14/204 | 97/8063 | 1.24E-07 | 7.54E-06 | 5.41E-06 | 208/1871/9134/596/5156/5159/3479/3320/5604/1027/5155/207/5728/595 | 14 |
| hsa05230 | Central carbon metabolism in cancer | 12/204 | 70/8063 | 1.46E-07 | 7.54E-06 | 5.41E-06 | 208/4233/4609/5156/5159/5604/8140/3939/441531/207/5728/5315 | 12 |
| hsa04218 | Cellular senescence | 17/204 | 156/8063 | 3.62E-07 | 1.37E-05 | 9.87E-06 | 208/1871/1019/9134/4609/1021/4773/7048/3708/5604/3105/91860/1111/207/5728/595/90550 | 17 |
| hsa05220 | Chronic myeloid leukemia | 12/204 | 76/8063 | 3.73E-07 | 1.37E-05 | 9.87E-06 | 208/1871/1019/4609/1021/4089/7048/5604/1027/207/595/598 | 12 |
| hsa04115 | p53 signaling pathway | 11/204 | 73/8063 | 1.87E-06 | 6.04E-05 | 4.34E-05 | 1019/9134/596/1021/3479/4194/83667/1111/5728/595/598 | 11 |
| hsa04151 | PI3K-Akt signaling pathway | 25/204 | 354/8063 | 2.83E-06 | 6.87E-05 | 4.93E-05 | 208/4602/4233/1019/9134/596/4609/1021/5156/5159/3479/3320/3570/5604/1027/3575/1435/22801/3690/5155/207/5728/595/11140/598 | 25 |
| hsa04510 | Focal adhesion | 18/204 | 201/8063 | 2.97E-06 | 6.87E-05 | 4.93E-05 | 208/857/4233/596/5156/5159/3479/5604/10451/22801/3690/9564/5155/207/5579/5728/595/5829 | 18 |
| hsa05222 | Small cell lung cancer | 12/204 | 92/8063 | 3.06E-06 | 6.87E-05 | 4.93E-05 | 208/1871/1019/9134/596/4609/1021/1027/207/5728/595/598 | 12 |
| hsa05206 | MicroRNAs in cancer | 23/204 | 310/8063 | 3.19E-06 | 6.87E-05 | 4.93E-05 | 4233/1871/9134/596/4609/1021/4855/4851/90427/5156/5159/6659/4194/7430/5604/1027/3690/23414/5155/5579/5728/595/6624 | 23 |
| hsa05163 | Human cytomegalovirus infection | 19/204 | 225/8063 | 3.74E-06 | 7.42E-05 | 5.33E-05 | 208/1871/1019/4609/1021/4773/5156/3570/3708/5604/3105/567/91860/3690/9564/207/5579/595/5829 | 19 |
| hsa01522 | Endocrine resistance | 12/204 | 98/8063 | 5.99E-06 | 0.0001104 | 7.93E-05 | 208/1871/1019/596/4855/4851/3479/5604/1027/207/595/10498 | 12 |
| hsa05166 | Human T-cell leukemia virus 1 infection | 18/204 | 219/8063 | 9.93E-06 | 0.0001708 | 0.0001227 | 208/1871/1019/9134/4609/4773/4089/7048/8061/5604/3105/567/1111/115650/207/5728/595/598 | 18 |
| hsa05212 | Pancreatic cancer | 10/204 | 76/8063 | 1.92E-05 | 0.0003096 | 0.0002223 | 208/1871/1019/1021/4089/7048/5604/207/595/598 | 10 |
| hsa05226 | Gastric cancer | 14/204 | 149/8063 | 2.26E-05 | 0.0003436 | 0.0002468 | 208/4233/1871/9134/596/4609/4089/7048/999/5604/1027/207/4583/595 | 14 |
| hsa05223 | Non-small cell lung cancer | 9/204 | 68/8063 | 4.82E-05 | 0.0006903 | 0.0004957 | 208/4233/1871/1019/1021/5604/207/5579/595 | 9 |
| hsa04630 | JAK-STAT signaling pathway | 14/204 | 162/8063 | 5.76E-05 | 0.0007823 | 0.0005618 | 208/596/4609/5156/5159/3570/163702/3572/3575/3977/5155/207/595/598 | 14 |
| hsa05225 | Hepatocellular carcinoma | 14/204 | 168/8063 | 8.57E-05 | 0.0011053 | 0.0007937 | 208/4233/1871/1019/4609/1021/4089/7048/5604/207/5579/5728/595/598 | 14 |
| hsa04066 | HIF-1 signaling pathway | 11/204 | 109/8063 | 9.11E-05 | 0.0011194 | 0.0008038 | 208/596/3479/3570/5604/3939/2872/1027/387712/207/5579 | 11 |
| hsa05205 | Proteoglycans in cancer | 15/204 | 205/8063 | 0.0002074 | 0.0024318 | 0.0017462 | 208/857/4233/4609/79923/3479/7430/3708/5604/10451/3690/207/5579/595/5829 | 15 |
| hsa04550 | Signaling pathways regulating pluripotency of stem cells | 12/204 | 143/8063 | 0.0002585 | 0.0028996 | 0.0020821 | 208/4609/6657/79923/3479/4089/5604/3572/657/3977/3720/207 | 12 |
| hsa04015 | Rap1 signaling pathway | 15/204 | 210/8063 | 0.0002697 | 0.0028996 | 0.0020821 | 208/4233/5156/5159/3479/999/5604/91860/1435/10451/3690/9564/5155/207/5579 | 15 |
| hsa05224 | Breast cancer | 12/204 | 147/8063 | 0.0003339 | 0.0034462 | 0.0024746 | 208/1871/1019/4609/1021/4855/4851/3479/5604/207/5728/595 | 12 |
| hsa05167 | Kaposi sarcoma-associated herpesvirus infection | 14/204 | 193/8063 | 0.0003691 | 0.0036626 | 0.0026301 | 208/1871/1019/4609/1021/4773/3708/5604/3105/3572/91860/5155/207/595 | 14 |
| hsa05219 | Bladder cancer | 6/204 | 41/8063 | 0.0005232 | 0.0049994 | 0.00359 | 1871/1019/4609/999/5604/595 | 6 |
| hsa05213 | Endometrial cancer | 7/204 | 58/8063 | 0.0006027 | 0.0055532 | 0.0039876 | 208/4609/999/5604/207/5728/595 | 7 |
| hsa04014 | Ras signaling pathway | 15/204 | 232/8063 | 0.0007744 | 0.0068895 | 0.0049472 | 208/4233/7535/5156/5159/3479/5604/26279/8036/91860/1435/5155/207/5579/598 | 15 |
| hsa04933 | AGE-RAGE signaling pathway in diabetic complications | 9/204 | 100/8063 | 0.0009259 | 0.0076484 | 0.0054921 | 208/1019/596/4089/7048/1027/207/5579/595 | 9 |
| hsa05170 | Human immunodeficiency virus 1 infection | 14/204 | 212/8063 | 0.0009421 | 0.0076484 | 0.0054921 | 208/596/4773/3708/5604/85363/3105/567/91860/1111/207/5579/5829/598 | 14 |
| hsa04919 | Thyroid hormone signaling pathway | 10/204 | 121/8063 | 0.0009486 | 0.0076484 | 0.0054921 | 208/4609/4855/4851/5604/29079/3690/207/5579/595 | 10 |
| hsa04110 | Cell cycle | 10/204 | 124/8063 | 0.0011458 | 0.0089582 | 0.0064327 | 1871/1019/9134/4609/1021/4089/1027/1111/595/51343 | 10 |
| hsa04010 | MAPK signaling pathway | 17/204 | 294/8063 | 0.0012262 | 0.0093047 | 0.0066815 | 208/4233/4609/4137/5156/5159/3479/3304/7048/5604/2872/1435/11221/10746/5155/207/5579 | 17 |
| hsa05210 | Colorectal cancer | 8/204 | 86/8063 | 0.0014319 | 0.0105551 | 0.0075793 | 208/596/4609/4089/7048/5604/207/595 | 8 |
| hsa04540 | Gap junction | 8/204 | 88/8063 | 0.0016616 | 0.011908 | 0.0085508 | 5156/5159/3708/5604/10746/5155/5579/7082 | 8 |
| hsa04068 | FoxO signaling pathway | 10/204 | 131/8063 | 0.0017382 | 0.0120142 | 0.0086271 | 208/3479/4089/7048/5604/1027/3575/207/5728/595 | 10 |
| hsa05165 | Human papillomavirus infection | 18/204 | 331/8063 | 0.0017695 | 0.0120142 | 0.0086271 | 208/1019/9134/1021/4855/4851/5159/5604/3105/1027/22801/3690/207/5728/595/5315/5829/529 | 18 |
| hsa04150 | mTOR signaling pathway | 11/204 | 155/8063 | 0.001866 | 0.0123443 | 0.0088641 | 208/3479/5604/8140/83667/58528/207/5579/5728/84335/529 | 11 |
| hsa04520 | Adherens junction | 7/204 | 71/8063 | 0.0020215 | 0.0130387 | 0.0093627 | 4233/6615/4089/7048/999/5795/7082 | 7 |
| hsa04666 | Fc gamma R-mediated phagocytosis | 8/204 | 93/8063 | 0.0023646 | 0.0148797 | 0.0106847 | 208/3636/5604/10451/8613/207/5579/4651 | 8 |
| hsa04140 | Autophagy - animal | 10/204 | 137/8063 | 0.0024246 | 0.0148939 | 0.0106949 | 208/596/3708/5604/58528/23130/207/5728/84335/598 | 10 |
| hsa05161 | Hepatitis B | 11/204 | 162/8063 | 0.002644 | 0.0158115 | 0.0113538 | 208/1871/9134/596/4609/4773/4089/7048/5604/207/5579 | 11 |
| hsa05162 | Measles | 10/204 | 139/8063 | 0.0026965 | 0.0158115 | 0.0113538 | 208/1019/9134/596/1021/3304/1027/207/595/598 | 10 |
| hsa05231 | Choline metabolism in cancer | 8/204 | 98/8063 | 0.0032823 | 0.0188186 | 0.0135132 | 208/5156/5159/5604/5155/8613/207/5579 | 8 |
| hsa04370 | VEGF signaling pathway | 6/204 | 59/8063 | 0.0035972 | 0.0201758 | 0.0144877 | 208/4773/5604/207/5579/5829 | 6 |
| hsa04144 | Endocytosis | 14/204 | 248/8063 | 0.0040697 | 0.0223401 | 0.0160418 | 857/5156/3304/7048/30845/3105/1601/9265/79720/10617/155382/28964/9230/84440 | 14 |
| hsa04662 | B cell receptor signaling pathway | 7/204 | 82/8063 | 0.0045774 | 0.0246038 | 0.0176673 | 208/3636/4773/5604/10451/207/5579 | 7 |
| hsa05169 | Epstein-Barr virus infection | 12/204 | 202/8063 | 0.0051224 | 0.0269711 | 0.0193672 | 208/1871/1019/9134/596/4609/1021/3105/567/1027/207/595 | 12 |
| hsa04929 | GnRH secretion | 6/204 | 64/8063 | 0.005394 | 0.027833 | 0.0199862 | 208/3708/5604/207/5579/9568 | 6 |
| hsa05203 | Viral carcinogenesis | 12/204 | 204/8063 | 0.0055361 | 0.028006 | 0.0201104 | 1019/9134/1021/3105/1027/3572/1111/8341/27044/595/5315/5829 | 12 |
| hsa04934 | Cushing syndrome | 10/204 | 155/8063 | 0.0058615 | 0.0290819 | 0.020883 | 1871/1019/9134/1021/3708/5604/8085/51655/1027/595 | 10 |
| hsa04915 | Estrogen signaling pathway | 9/204 | 138/8063 | 0.0082016 | 0.0399248 | 0.028669 | 208/596/3304/3320/3708/5604/91860/207/9568 | 9 |
| hsa05418 | Fluid shear stress and atherosclerosis | 9/204 | 139/8063 | 0.0085827 | 0.0410063 | 0.0294456 | 208/857/596/3320/91860/657/3690/5155/207 | 9 |
| hsa04071 | Sphingolipid signaling pathway | 8/204 | 119/8063 | 0.0104451 | 0.048997 | 0.0351835 | 208/596/166929/5604/4985/207/5579/5728 | 8 |
