# Supplementary material for: Identification of Potential miRNA-mRNA Regulatory Network Contributing to Hypertrophic Cardiomyopathy (HCM)
Source: Front Cardiovasc Med. 2021 May 31;8:660372. doi: 10.3389/fcvm.2021.660372 (PMC8200816; doi:10.3389/fcvm.2021.660372)
Supplement: Supplementary Table 3 — Overlapped GO terms and KEGG pathways in Supplementary Tables 1, 2. [file Table_3.DOCX]

Table S3 Overlapped GO terms and KEGG pathways in Table S1 and Table S2.

Overlapped GO terms

| ONTOLOGY | ID | Description |
| --- | --- | --- |
| BP | GO:0031099 | regeneration |
| BP | GO:2000379 | positive regulation of reactive oxygen species metabolic process |
| BP | GO:0033673 | negative regulation of kinase activity |
| BP | GO:0046677 | response to antibiotic |
| BP | GO:0045926 | negative regulation of growth |
| BP | GO:0051348 | negative regulation of transferase activity |
| BP | GO:0042326 | negative regulation of phosphorylation |
| BP | GO:0050867 | positive regulation of cell activation |
| BP | GO:0006469 | negative regulation of protein kinase activity |
| BP | GO:0010038 | response to metal ion |
| BP | GO:0016049 | cell growth |
| BP | GO:2000377 | regulation of reactive oxygen species metabolic process |
| BP | GO:0001933 | negative regulation of protein phosphorylation |
| BP | GO:0045785 | positive regulation of cell adhesion |
| BP | GO:0055001 | muscle cell development |
| BP | GO:0061448 | connective tissue development |
| BP | GO:0001501 | skeletal system development |
| BP | GO:0031100 | animal organ regeneration |
| BP | GO:0033002 | muscle cell proliferation |
| BP | GO:0060537 | muscle tissue development |
| BP | GO:0072006 | nephron development |
| BP | GO:0001649 | osteoblast differentiation |
| BP | GO:0022407 | regulation of cell-cell adhesion |
| BP | GO:0001503 | ossification |
| BP | GO:0007517 | muscle organ development |
| BP | GO:0002696 | positive regulation of leukocyte activation |
| BP | GO:0032835 | glomerulus development |
| BP | GO:0014706 | striated muscle tissue development |
| BP | GO:0072012 | glomerulus vasculature development |
| BP | GO:0001655 | urogenital system development |
| BP | GO:0060419 | heart growth |
| BP | GO:0048008 | platelet-derived growth factor receptor signaling pathway |
| BP | GO:0051216 | cartilage development |
| BP | GO:0007568 | aging |
| BP | GO:0061437 | renal system vasculature development |
| BP | GO:0061440 | kidney vasculature development |
| BP | GO:0048660 | regulation of smooth muscle cell proliferation |
| BP | GO:0003229 | ventricular cardiac muscle tissue development |
| BP | GO:0048659 | smooth muscle cell proliferation |
| BP | GO:0070371 | ERK1 and ERK2 cascade |
| BP | GO:0072224 | metanephric glomerulus development |
| BP | GO:0035265 | organ growth |
| BP | GO:0070372 | regulation of ERK1 and ERK2 cascade |
| BP | GO:0010642 | negative regulation of platelet-derived growth factor receptor signaling pathway |
| BP | GO:0035966 | response to topologically incorrect protein |
| BP | GO:0048738 | cardiac muscle tissue development |
| BP | GO:0006986 | response to unfolded protein |
| BP | GO:0032944 | regulation of mononuclear cell proliferation |
| BP | GO:0010721 | negative regulation of cell development |
| BP | GO:0048732 | gland development |
| BP | GO:0030278 | regulation of ossification |
| BP | GO:0071901 | negative regulation of protein serine/threonine kinase activity |
| BP | GO:0006979 | response to oxidative stress |
| BP | GO:0055010 | ventricular cardiac muscle tissue morphogenesis |
| BP | GO:0090066 | regulation of anatomical structure size |
| BP | GO:0070663 | regulation of leukocyte proliferation |
| BP | GO:0003281 | ventricular septum development |
| BP | GO:0048661 | positive regulation of smooth muscle cell proliferation |
| BP | GO:0035904 | aorta development |
| BP | GO:2000045 | regulation of G1/S transition of mitotic cell cycle |
| BP | GO:0060840 | artery development |
| BP | GO:1903039 | positive regulation of leukocyte cell-cell adhesion |
| BP | GO:0048844 | artery morphogenesis |
| BP | GO:0030335 | positive regulation of cell migration |
| BP | GO:0070482 | response to oxygen levels |
| BP | GO:0003208 | cardiac ventricle morphogenesis |
| BP | GO:0010640 | regulation of platelet-derived growth factor receptor signaling pathway |
| BP | GO:0045667 | regulation of osteoblast differentiation |
| BP | GO:0055017 | cardiac muscle tissue growth |
| BP | GO:0060420 | regulation of heart growth |
| BP | GO:2000134 | negative regulation of G1/S transition of mitotic cell cycle |
| BP | GO:0060412 | ventricular septum morphogenesis |
| BP | GO:0048705 | skeletal system morphogenesis |
| BP | GO:0035791 | platelet-derived growth factor receptor-beta signaling pathway |
| BP | GO:0048010 | vascular endothelial growth factor receptor signaling pathway |
| BP | GO:0050870 | positive regulation of T cell activation |
| BP | GO:0035967 | cellular response to topologically incorrect protein |
| BP | GO:0003231 | cardiac ventricle development |
| BP | GO:0072109 | glomerular mesangium development |
| BP | GO:0072216 | positive regulation of metanephros development |
| BP | GO:0001666 | response to hypoxia |
| BP | GO:1902807 | negative regulation of cell cycle G1/S phase transition |
| BP | GO:1902806 | regulation of cell cycle G1/S phase transition |
| BP | GO:0003206 | cardiac chamber morphogenesis |
| BP | GO:0048608 | reproductive structure development |
| BP | GO:0061458 | reproductive system development |
| BP | GO:0072075 | metanephric mesenchyme development |
| BP | GO:0034620 | cellular response to unfolded protein |
| BP | GO:0046620 | regulation of organ growth |
| BP | GO:0036293 | response to decreased oxygen levels |
| BP | GO:0043552 | positive regulation of phosphatidylinositol 3-kinase activity |
| CC | GO:0005925 | focal adhesion |
| CC | GO:0005924 | cell-substrate adherens junction |
| CC | GO:0030055 | cell-substrate junction |
| CC | GO:0043235 | receptor complex |
| CC | GO:0098802 | plasma membrane receptor complex |

Overlapped KEGG pathways

| ID | Description |
| --- | --- |
| hsa05218 | Melanoma |
| hsa05214 | Glioma |
| hsa05230 | Central carbon metabolism in cancer |
| hsa04218 | Cellular senescence |
| hsa05220 | Chronic myeloid leukemia |
| hsa04115 | p53 signaling pathway |
| hsa04510 | Focal adhesion |
| hsa05206 | MicroRNAs in cancer |
| hsa05166 | Human T-cell leukemia virus 1 infection |
| hsa05212 | Pancreatic cancer |
| hsa05223 | Non-small cell lung cancer |
| hsa04630 | JAK-STAT signaling pathway |
| hsa04066 | HIF-1 signaling pathway |
| hsa05205 | Proteoglycans in cancer |
| hsa05167 | Kaposi sarcoma-associated herpesvirus infection |
| hsa05219 | Bladder cancer |
| hsa05213 | Endometrial cancer |
| hsa04933 | AGE-RAGE signaling pathway in diabetic complications |
| hsa04919 | Thyroid hormone signaling pathway |
| hsa04010 | MAPK signaling pathway |
| hsa05210 | Colorectal cancer |
| hsa04540 | Gap junction |
| hsa04068 | FoxO signaling pathway |
| hsa04666 | Fc gamma R-mediated phagocytosis |
