# Supplementary material for: Identification of Potential miRNA-mRNA Regulatory Network Contributing to Hypertrophic Cardiomyopathy (HCM)
Source: Front Cardiovasc Med. 2021 May 31;8:660372. doi: 10.3389/fcvm.2021.660372 (PMC8200816; doi:10.3389/fcvm.2021.660372)
Supplement: Supplementary Table 4 — Negatively regulated miRNA-mRNA pairs of 24 DEMs and 20 overlapped mRNAs. [file Table_4.DOCX]

Table S4 Negatively regulated miRNA-mRNA pairs of 24 DEMs and 20 overlapped mRNAs.

| miRNA | mRNA | Correlation coefficient |
| --- | --- | --- |
| hsa-miR-452 | ARHGDIA | -0.271180096 |
| hsa-miR-371-3p | ARHGDIA | -0.118149359 |
| hsa-miR-34b | ARHGDIA | -0.124366393 |
| hsa-miR-373 | ARHGDIA | -0.242967556 |
| hsa-miR-452-1 | ARHGDIA | -0.234098275 |
| hsa-miR-184 | ARHGDIA | -0.146256434 |
| hsa-miR-34c-5p | ARHGDIA | -0.240046381 |
| hsa-miR-514 | ARHGDIA | -0.302454317 |
| hsa-miR-499-3p | ARHGDIA | -0.04190255 |
| hsa-miR-190b | ARHGDIA | -0.196640642 |
| hsa-miR-18a | CCND1 | -0.368108676 |
| hsa-miR-10a | CCND1 | -0.319560576 |
| hsa-miR-511 | CCND1 | -0.274904157 |
| hsa-miR-139-3p | CCND1 | -0.40903456 |
| hsa-miR-187 | CCND1 | -0.173258628 |
| hsa-miR-1247 | CCND1 | -0.18657607 |
| hsa-miR-92a-1 | CCND1 | -0.375982716 |
| hsa-miR-10a- | CCND1 | -0.315118527 |
| hsa-miR-21- | CCND1 | -0.171196111 |
| hsa-miR-1268 | CCND1 | -0.394315841 |
| hsa-miR-886-5p | CCND1 | -0.097396543 |
| hsa-miR-223 | CCND1 | -0.453389103 |
| hsa-miR-30c-1 | CCND1 | -0.109824655 |
| hsa-miR-144 | CCND1 | -0.305391134 |
| hsa-miR-452 | KLF6 | -0.265998108 |
| hsa-miR-371-3p | KLF6 | -0.148365891 |
| hsa-miR-34b | KLF6 | -0.207453316 |
| hsa-miR-373 | KLF6 | -0.218075213 |
| hsa-miR-452-1 | KLF6 | -0.205652913 |
| hsa-miR-184 | KLF6 | -0.205749024 |
| hsa-miR-34c-5p | KLF6 | -0.319035267 |
| hsa-miR-514 | KLF6 | -0.297237593 |
| hsa-miR-499-3p | KLF6 | -0.08571372 |
| hsa-miR-190b | KLF6 | -0.226850022 |
| hsa-miR-18a | KLHL24 | -0.289320865 |
| hsa-miR-10a | KLHL24 | -0.126441974 |
| hsa-miR-511 | KLHL24 | -0.181372879 |
| hsa-miR-139-3p | KLHL24 | -0.159707544 |
| hsa-miR-187 | KLHL24 | -0.12859202 |
| hsa-miR-1247 | KLHL24 | -0.060416515 |
| hsa-miR-92a-1 | KLHL24 | -0.361902118 |
| hsa-miR-10a- | KLHL24 | -0.190919143 |
| hsa-miR-21- | KLHL24 | -0.441774461 |
| hsa-miR-1268 | KLHL24 | -0.395666991 |
| hsa-miR-886-5p | KLHL24 | -0.339243452 |
| hsa-miR-223 | KLHL24 | -0.428373634 |
| hsa-miR-30c-1 | KLHL24 | -0.210693173 |
| hsa-miR-144 | KLHL24 | -0.043922192 |
| hsa-miR-452 | LDHA | -0.171998926 |
| hsa-miR-371-3p | LDHA | -0.097262254 |
| hsa-miR-34b | LDHA | -0.120634427 |
| hsa-miR-373 | LDHA | -0.189927244 |
| hsa-miR-452-1 | LDHA | -0.16869459 |
| hsa-miR-184 | LDHA | -0.180275644 |
| hsa-miR-34c-5p | LDHA | -0.193861559 |
| hsa-miR-514 | LDHA | -0.305303876 |
| hsa-miR-190b | LDHA | -0.232681595 |
| hsa-miR-452 | LRRC8A | -0.253913443 |
| hsa-miR-371-3p | LRRC8A | -0.152072938 |
| hsa-miR-34b | LRRC8A | -0.211998198 |
| hsa-miR-373 | LRRC8A | -0.231498645 |
| hsa-miR-452-1 | LRRC8A | -0.190092197 |
| hsa-miR-184 | LRRC8A | -0.262285879 |
| hsa-miR-34c-5p | LRRC8A | -0.284405934 |
| hsa-miR-514 | LRRC8A | -0.299027657 |
| hsa-miR-190b | LRRC8A | -0.21681617 |
| hsa-miR-452 | MAP2K1 | -0.172333119 |
| hsa-miR-371-3p | MAP2K1 | -0.224074041 |
| hsa-miR-34b | MAP2K1 | -0.255932739 |
| hsa-miR-373 | MAP2K1 | -0.253045922 |
| hsa-miR-452-1 | MAP2K1 | -0.091211459 |
| hsa-miR-184 | MAP2K1 | -0.279784413 |
| hsa-miR-34c-5p | MAP2K1 | -0.304161584 |
| hsa-miR-514 | MAP2K1 | -0.334258733 |
| hsa-miR-190b | MAP2K1 | -0.116400524 |
| hsa-miR-18a | MAP4 | -0.359026731 |
| hsa-miR-10a | MAP4 | -0.290640748 |
| hsa-miR-511 | MAP4 | -0.148114884 |
| hsa-miR-139-3p | MAP4 | -0.259379564 |
| hsa-miR-187 | MAP4 | -0.221397989 |
| hsa-miR-1247 | MAP4 | -0.154630195 |
| hsa-miR-92a-1 | MAP4 | -0.361311513 |
| hsa-miR-10a- | MAP4 | -0.206572187 |
| hsa-miR-21- | MAP4 | -0.219881073 |
| hsa-miR-1268 | MAP4 | -0.331584553 |
| hsa-miR-886-5p | MAP4 | -0.150309086 |
| hsa-miR-223 | MAP4 | -0.383350765 |
| hsa-miR-30c-1 | MAP4 | -0.145147711 |
| hsa-miR-144 | MAP4 | -0.178937072 |
| hsa-miR-452 | MYC | -0.216066534 |
| hsa-miR-371-3p | MYC | -0.25371985 |
| hsa-miR-34b | MYC | -0.225973356 |
| hsa-miR-373 | MYC | -0.375284821 |
| hsa-miR-452-1 | MYC | -0.227085583 |
| hsa-miR-184 | MYC | -0.283717728 |
| hsa-miR-34c-5p | MYC | -0.347437575 |
| hsa-miR-514 | MYC | -0.393315671 |
| hsa-miR-190b | MYC | -0.302254169 |
| hsa-miR-18a | OMD | -0.326079182 |
| hsa-miR-10a | OMD | -0.299938389 |
| hsa-miR-511 | OMD | -0.027215115 |
| hsa-miR-139-3p | OMD | -0.303612806 |
| hsa-miR-187 | OMD | -0.065860089 |
| hsa-miR-499-3p | OMD | -0.073456315 |
| hsa-miR-1247 | OMD | -0.133860798 |
| hsa-miR-92a-1 | OMD | -0.249007001 |
| hsa-miR-10a- | OMD | -0.294285028 |
| hsa-miR-21- | OMD | -0.207005349 |
| hsa-miR-1268 | OMD | -0.226281019 |
| hsa-miR-886-5p | OMD | -0.095219846 |
| hsa-miR-223 | OMD | -0.365010802 |
| hsa-miR-30c-1 | OMD | -0.146836499 |
| hsa-miR-144 | OMD | -0.277000677 |
| hsa-miR-452 | PDGFRA | -0.166940987 |
| hsa-miR-371-3p | PDGFRA | -0.201176936 |
| hsa-miR-34b | PDGFRA | -0.326946241 |
| hsa-miR-373 | PDGFRA | -0.312512452 |
| hsa-miR-452-1 | PDGFRA | -0.226205258 |
| hsa-miR-184 | PDGFRA | -0.175840623 |
| hsa-miR-34c-5p | PDGFRA | -0.380101204 |
| hsa-miR-514 | PDGFRA | -0.329136831 |
| hsa-miR-499-3p | PDGFRA | -0.129316194 |
| hsa-miR-190b | PDGFRA | -0.144046203 |
| hsa-miR-452 | PDGFRB | -0.313760332 |
| hsa-miR-34b | PDGFRB | -0.184697968 |
| hsa-miR-373 | PDGFRB | -0.087385147 |
| hsa-miR-452-1 | PDGFRB | -0.36050059 |
| hsa-miR-184 | PDGFRB | -0.136737081 |
| hsa-miR-34c-5p | PDGFRB | -0.310442024 |
| hsa-miR-514 | PDGFRB | -0.373952951 |
| hsa-miR-499-3p | PDGFRB | -0.151487149 |
| hsa-miR-190b | PDGFRB | -0.228885405 |
| hsa-miR-452 | PVR | -0.220820323 |
| hsa-miR-371-3p | PVR | -0.207831358 |
| hsa-miR-34b | PVR | -0.177645371 |
| hsa-miR-373 | PVR | -0.323530426 |
| hsa-miR-452-1 | PVR | -0.238127195 |
| hsa-miR-184 | PVR | -0.283886599 |
| hsa-miR-34c-5p | PVR | -0.300428937 |
| hsa-miR-514 | PVR | -0.264775453 |
| hsa-miR-499-3p | PVR | -0.011273452 |
| hsa-miR-1247 | PVR | -0.009292902 |
| hsa-miR-190b | PVR | -0.288803993 |
| hsa-miR-452 | RASD1 | -0.38564771 |
| hsa-miR-371-3p | RASD1 | -0.338773679 |
| hsa-miR-34b | RASD1 | -0.265595863 |
| hsa-miR-373 | RASD1 | -0.4702363 |
| hsa-miR-452-1 | RASD1 | -0.417199308 |
| hsa-miR-184 | RASD1 | -0.335916769 |
| hsa-miR-34c-5p | RASD1 | -0.431660596 |
| hsa-miR-514 | RASD1 | -0.494552767 |
| hsa-miR-499-3p | RASD1 | -0.158584511 |
| hsa-miR-190b | RASD1 | -0.25243363 |
| hsa-miR-452 | SEC61A1 | -0.260338354 |
| hsa-miR-371-3p | SEC61A1 | -0.185925778 |
| hsa-miR-34b | SEC61A1 | -0.242375708 |
| hsa-miR-373 | SEC61A1 | -0.253147919 |
| hsa-miR-452-1 | SEC61A1 | -0.164572499 |
| hsa-miR-184 | SEC61A1 | -0.350221863 |
| hsa-miR-34c-5p | SEC61A1 | -0.30097791 |
| hsa-miR-514 | SEC61A1 | -0.3781689 |
| hsa-miR-190b | SEC61A1 | -0.292455707 |
| hsa-miR-452 | SLC7A5 | -0.153056976 |
| hsa-miR-371-3p | SLC7A5 | -0.186256551 |
| hsa-miR-34b | SLC7A5 | -0.118335795 |
| hsa-miR-373 | SLC7A5 | -0.311691997 |
| hsa-miR-452-1 | SLC7A5 | -0.180703867 |
| hsa-miR-184 | SLC7A5 | -0.163328247 |
| hsa-miR-34c-5p | SLC7A5 | -0.220265745 |
| hsa-miR-514 | SLC7A5 | -0.299702777 |
| hsa-miR-190b | SLC7A5 | -0.104812401 |
| hsa-miR-18a | SNCA | -0.241396845 |
| hsa-miR-10a | SNCA | -0.170018335 |
| hsa-miR-511 | SNCA | -0.278460674 |
| hsa-miR-139-3p | SNCA | -0.276579455 |
| hsa-miR-187 | SNCA | -0.146860852 |
| hsa-miR-1247 | SNCA | -0.147170963 |
| hsa-miR-92a-1 | SNCA | -0.321650527 |
| hsa-miR-10a- | SNCA | -0.221600632 |
| hsa-miR-21- | SNCA | -0.094468768 |
| hsa-miR-1268 | SNCA | -0.27989384 |
| hsa-miR-886-5p | SNCA | -0.254606583 |
| hsa-miR-223 | SNCA | -0.293344507 |
| hsa-miR-30c-1 | SNCA | -0.225076855 |
| hsa-miR-144 | SNCA | -0.269909054 |
| hsa-miR-18a | SORBS2 | -0.425598829 |
| hsa-miR-10a | SORBS2 | -0.267271354 |
| hsa-miR-511 | SORBS2 | -0.40389347 |
| hsa-miR-139-3p | SORBS2 | -0.379157695 |
| hsa-miR-187 | SORBS2 | -0.253913101 |
| hsa-miR-1247 | SORBS2 | -0.230356398 |
| hsa-miR-92a-1 | SORBS2 | -0.500959966 |
| hsa-miR-10a- | SORBS2 | -0.366900294 |
| hsa-miR-21- | SORBS2 | -0.386694702 |
| hsa-miR-1268 | SORBS2 | -0.415095193 |
| hsa-miR-886-5p | SORBS2 | -0.236904597 |
| hsa-miR-223 | SORBS2 | -0.50543325 |
| hsa-miR-30c-1 | SORBS2 | -0.278682523 |
| hsa-miR-144 | SORBS2 | -0.265987758 |
| hsa-miR-18a | SOX4 | -0.245156401 |
| hsa-miR-10a | SOX4 | -0.265934384 |
| hsa-miR-511 | SOX4 | -0.274316262 |
| hsa-miR-139-3p | SOX4 | -0.357418856 |
| hsa-miR-187 | SOX4 | -0.18975051 |
| hsa-miR-1247 | SOX4 | -0.138657297 |
| hsa-miR-92a-1 | SOX4 | -0.259459275 |
| hsa-miR-10a- | SOX4 | -0.303297458 |
| hsa-miR-21- | SOX4 | -0.077117448 |
| hsa-miR-1268 | SOX4 | -0.154259344 |
| hsa-miR-886-5p | SOX4 | -0.152867262 |
| hsa-miR-223 | SOX4 | -0.287740958 |
| hsa-miR-30c-1 | SOX4 | -0.175600874 |
| hsa-miR-144 | SOX4 | -0.328064866 |
| hsa-miR-18a | THY1 | -0.017412937 |
| hsa-miR-10a | THY1 | -0.205626612 |
| hsa-miR-511 | THY1 | -0.068844947 |
| hsa-miR-139-3p | THY1 | -0.29175292 |
| hsa-miR-187 | THY1 | -0.095896042 |
| hsa-miR-499-3p | THY1 | -0.05785411 |
| hsa-miR-92a-1 | THY1 | -0.082941944 |
| hsa-miR-10a- | THY1 | -0.181550731 |
| hsa-miR-1268 | THY1 | -0.141462285 |
| hsa-miR-223 | THY1 | -0.094971915 |
| hsa-miR-30c-1 | THY1 | -0.048149161 |
| hsa-miR-144 | THY1 | -0.298308224 |
